# Supplementary material for: Functional Carbohydrate-Active Enzymes Acquired by Horizontal Gene Transfer from Plants in the Whitefly Bemisia tabaci
Source: Genome Biol Evol. 2025 Jan 25;17(2):evaf012. doi: 10.1093/gbe/evaf012 (PMC11800479; doi:10.1093/gbe/evaf012)

Supplementary Figure 1. Comparison of the number of HGT candidates found for B. tabaci MEAM1 with bacteria (A), fungi (B) or plant (C) as donors with data from the literature.

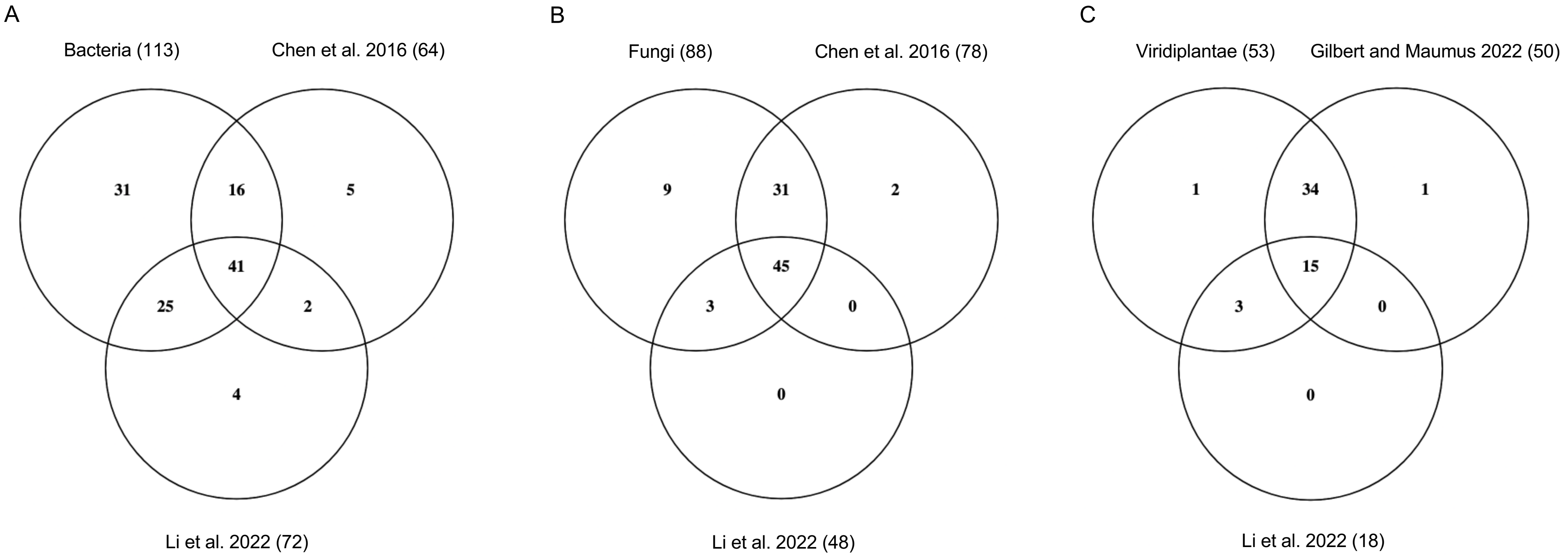

Supplementary Figure 2.
Phylogeny inferred for the HGT events corresponding to CAZymes in *B. tabaci* and *T. vaporariorum.* Gray circles indicate nodes with support values greater than or equal to 80% and 95% for SH-aLRT and UFboot, respectively.

Donor: Bacteria
BtaB53: GH32

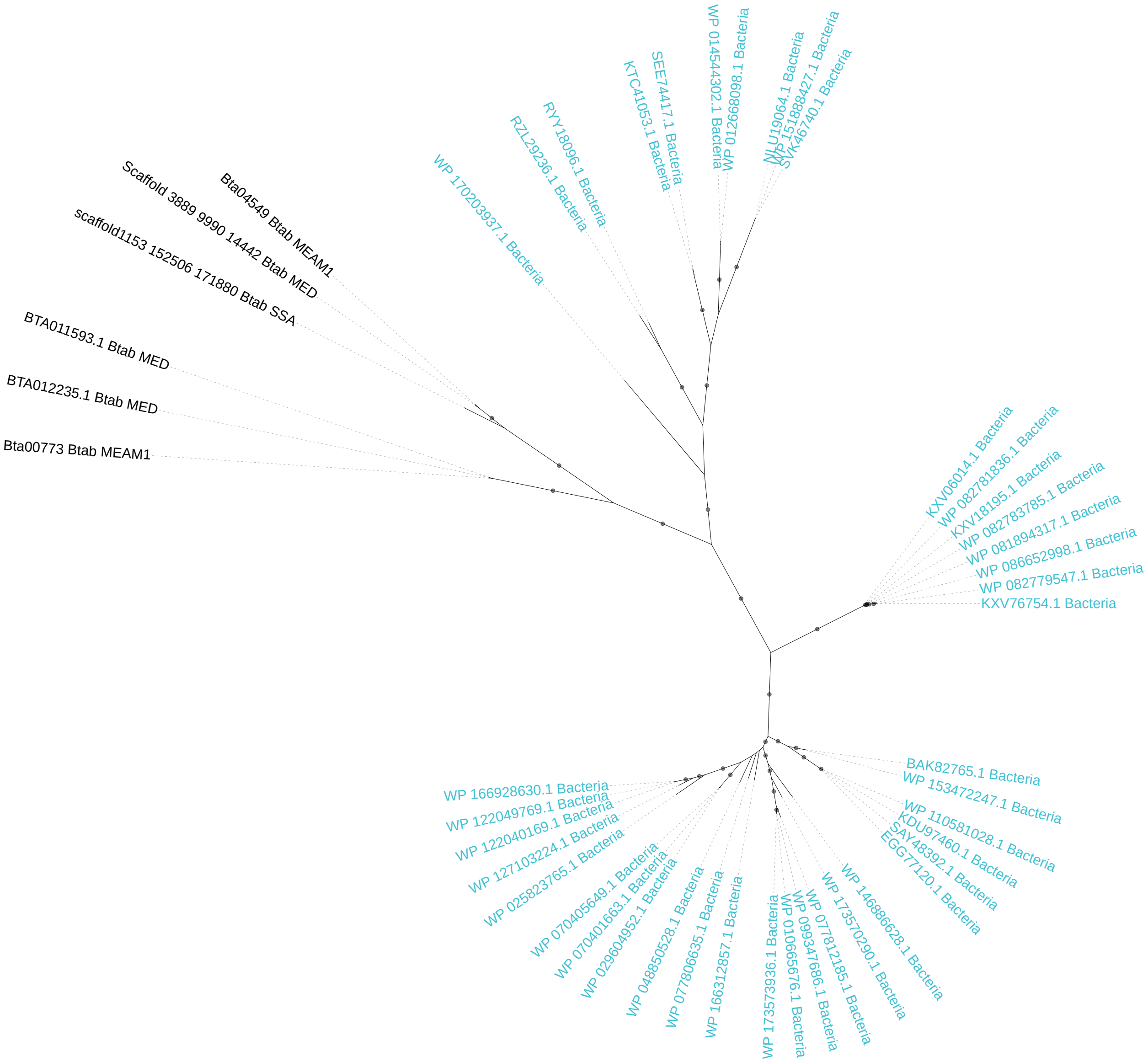

Donor: Bacteria
BtaB62: CBM50

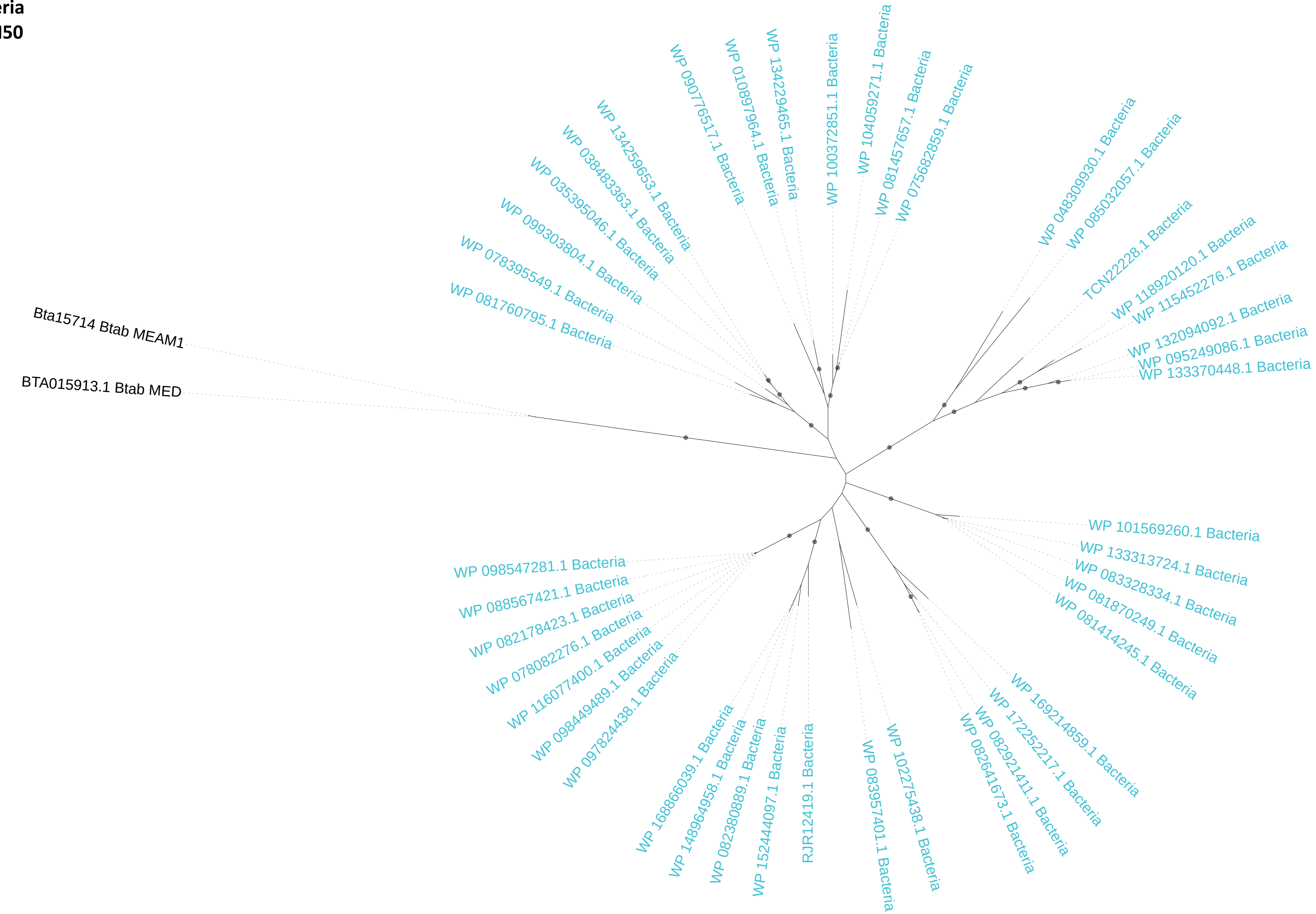

Donor: Bacteria
TvaB10: GH13

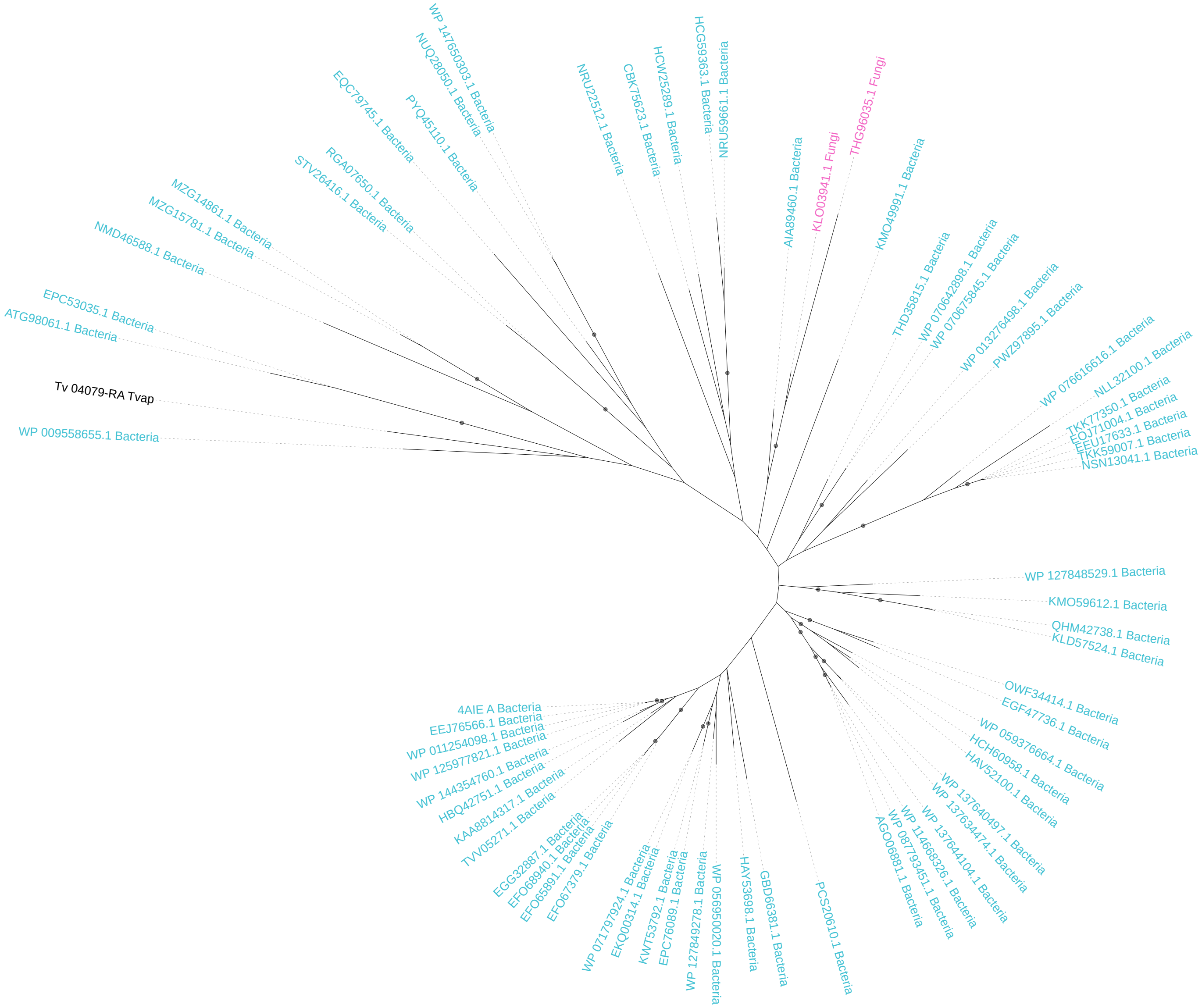

Donor: Fungi
BtaF06: GH49

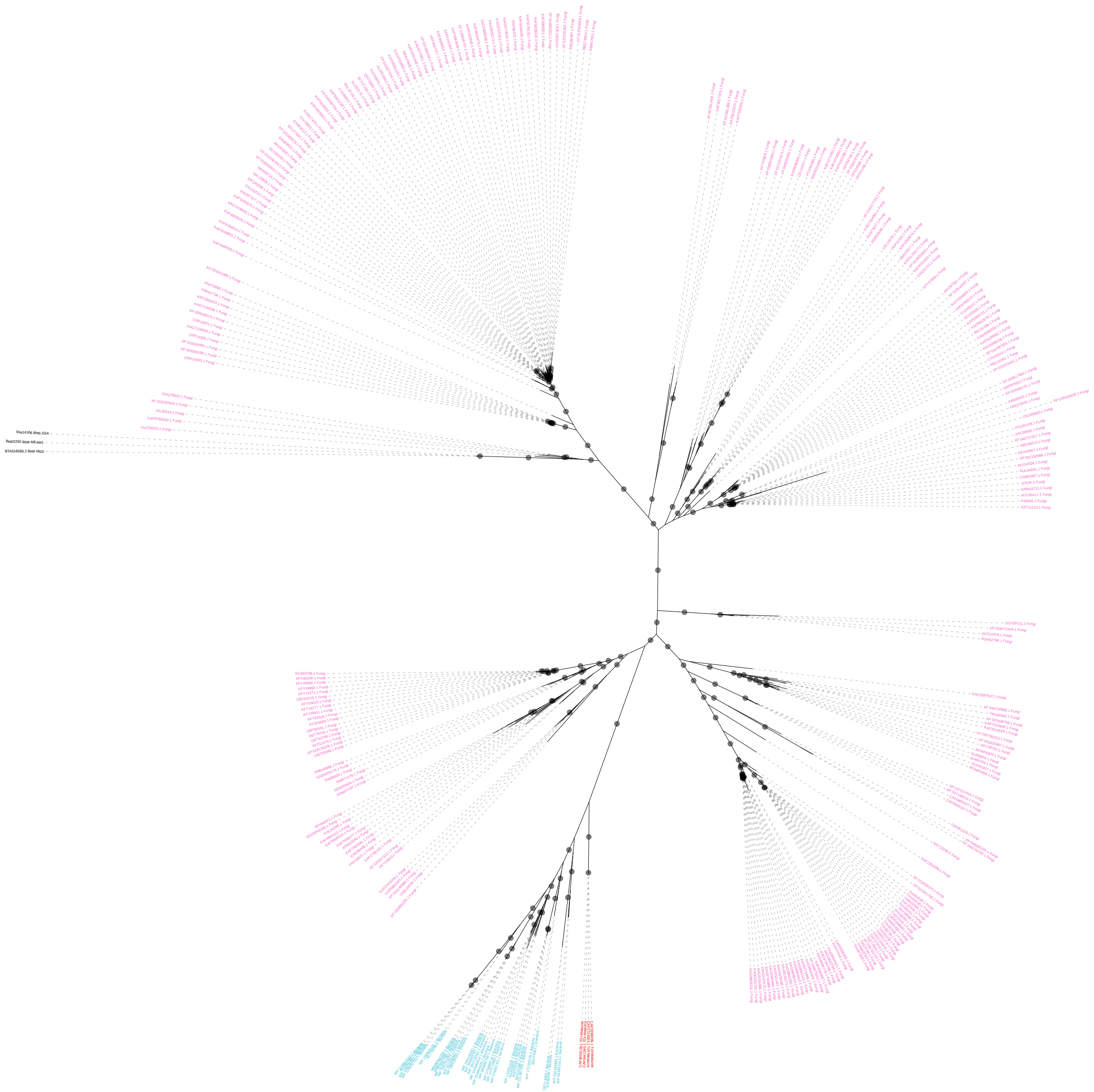

Donor: Fungi
BtaF15: GH71

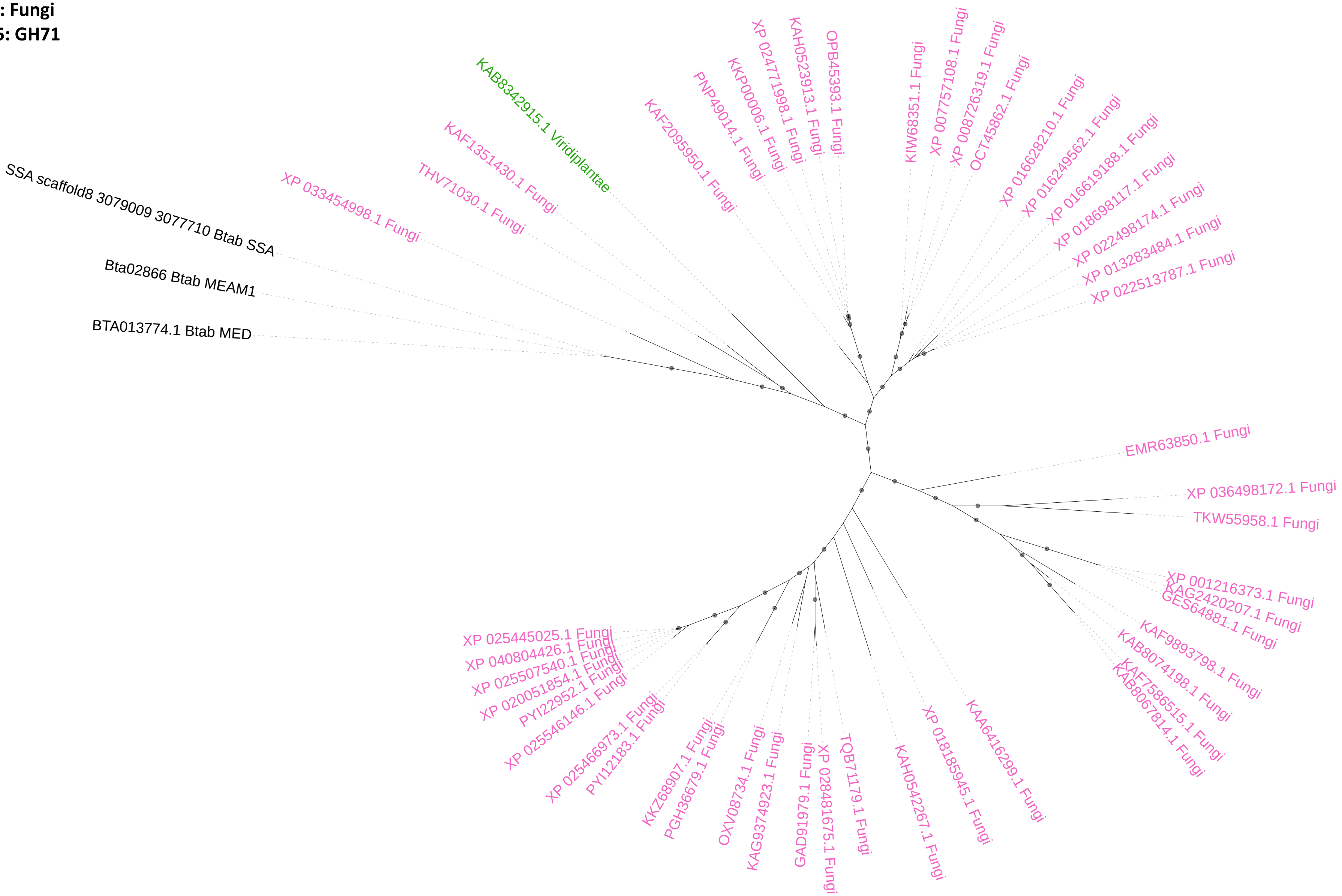

Donor: Fungi
BtaF18: CBM32-AA5_2

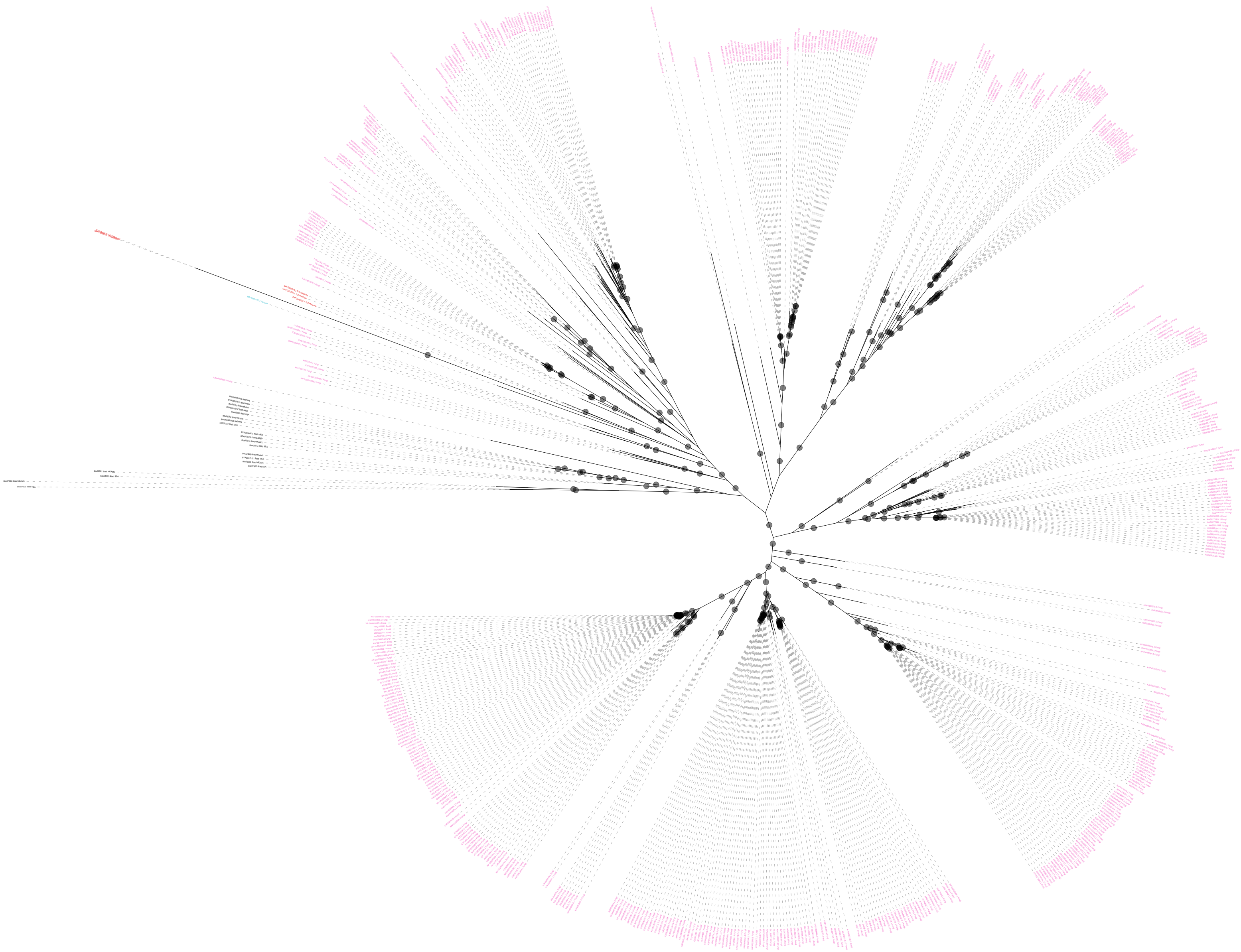

Donor: Fungi
BtaF20: GH30_3

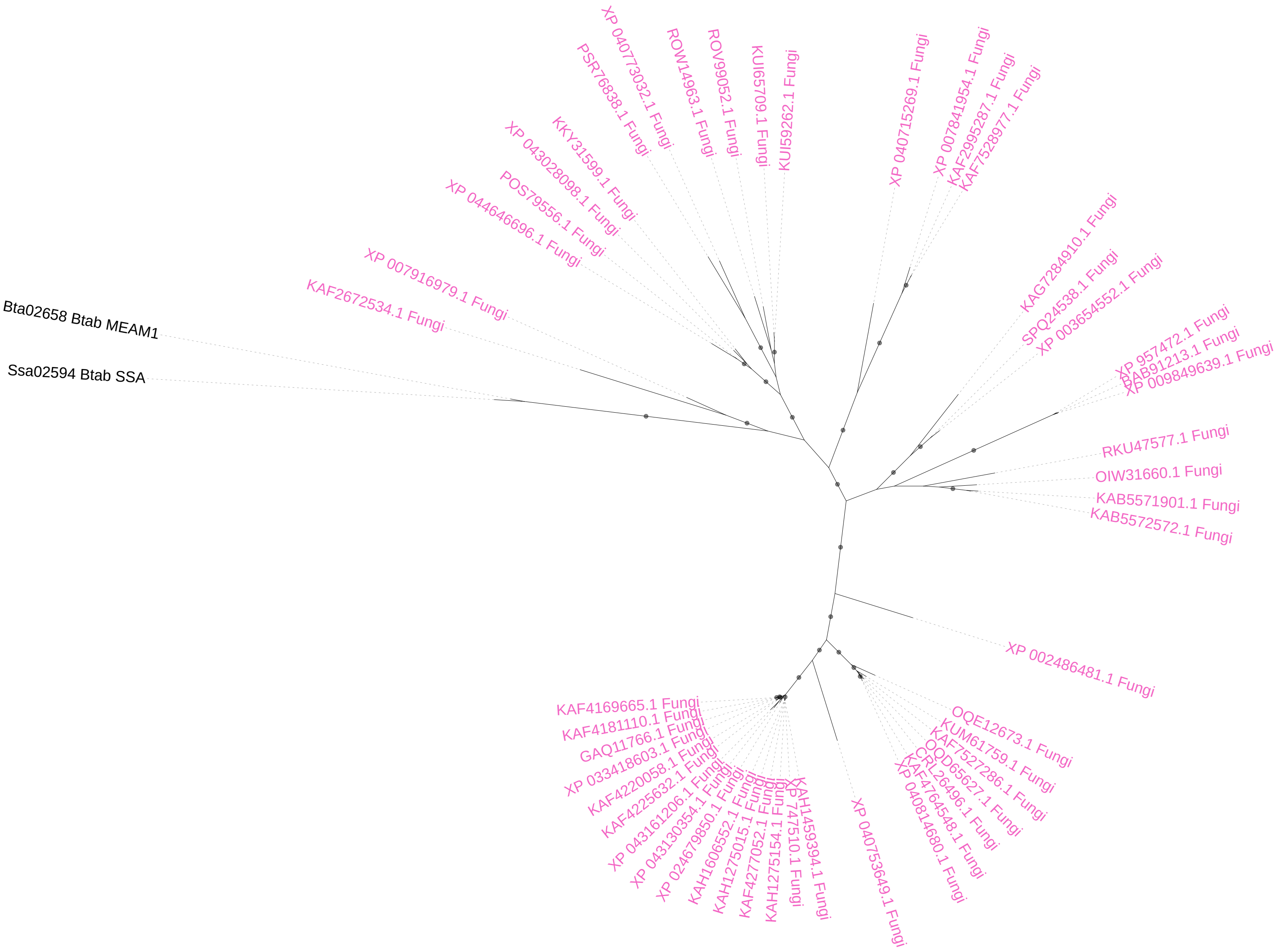

Donor: Viridiplantae
BtaV01_TvaV01: GH152

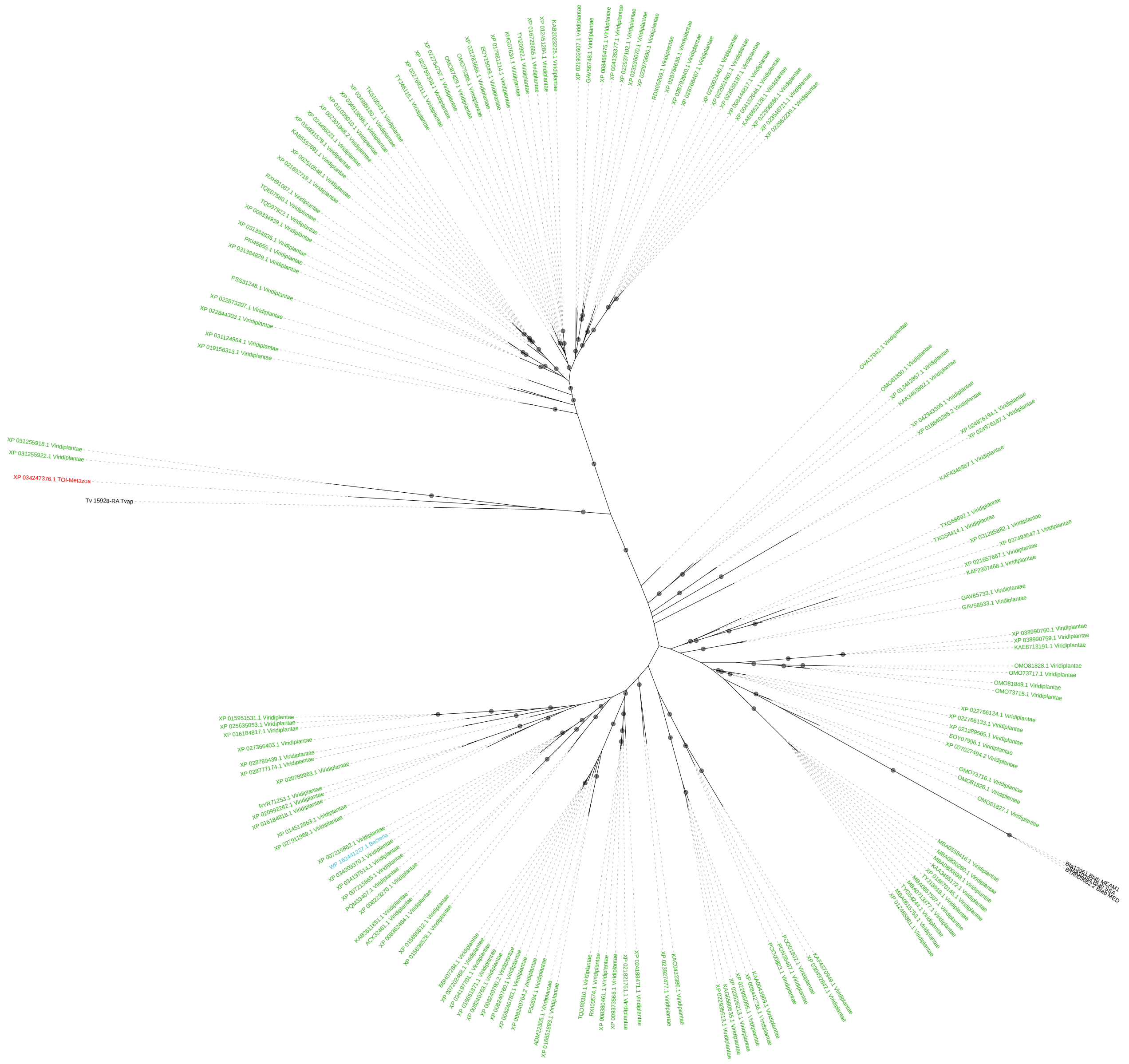

Donor: Viridiplantae
BtaV10: GT10

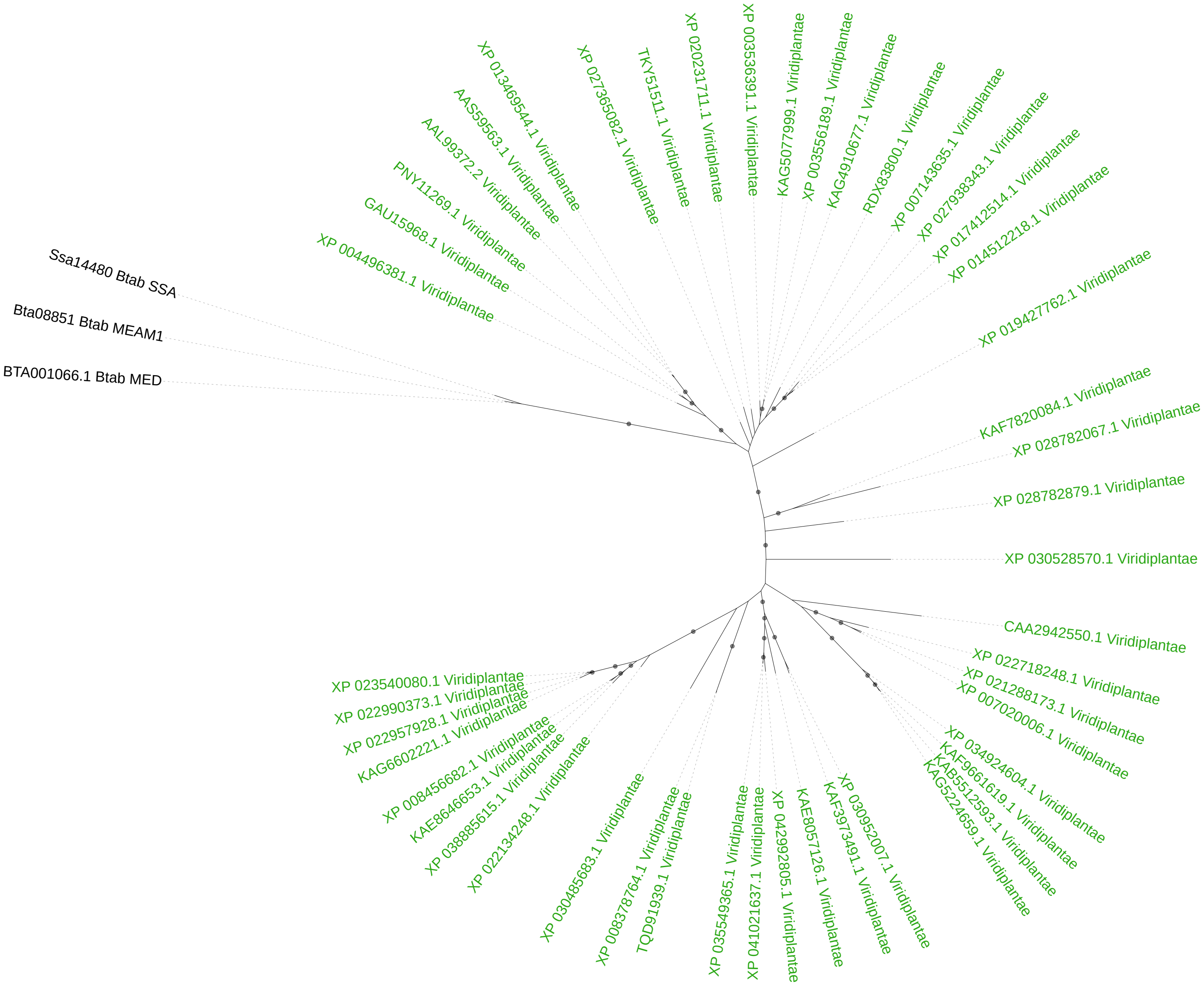

Donor: Viridiplantae
BtaV12: GH17

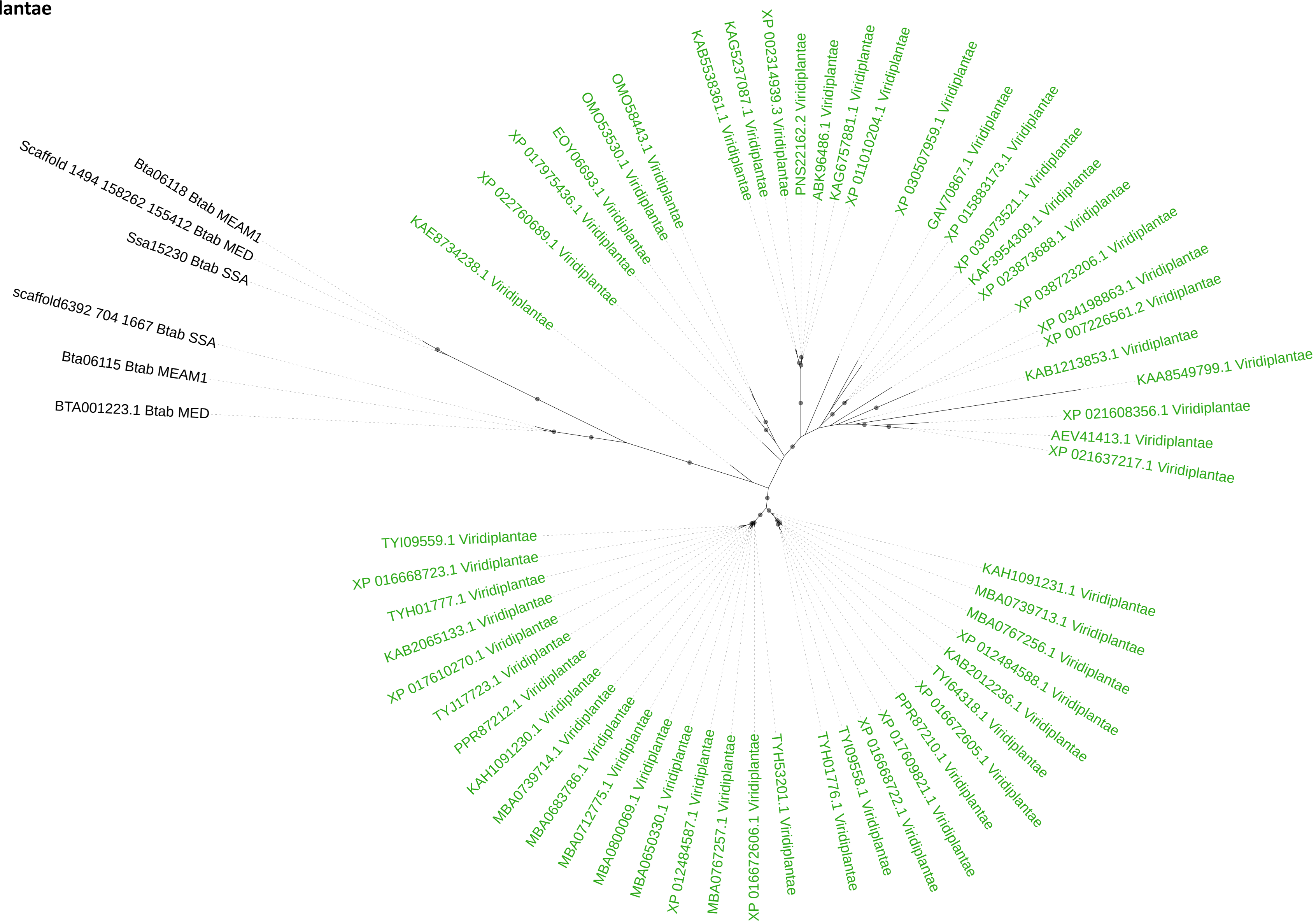

Donor: Viridiplantae
BtaV13: EXPN

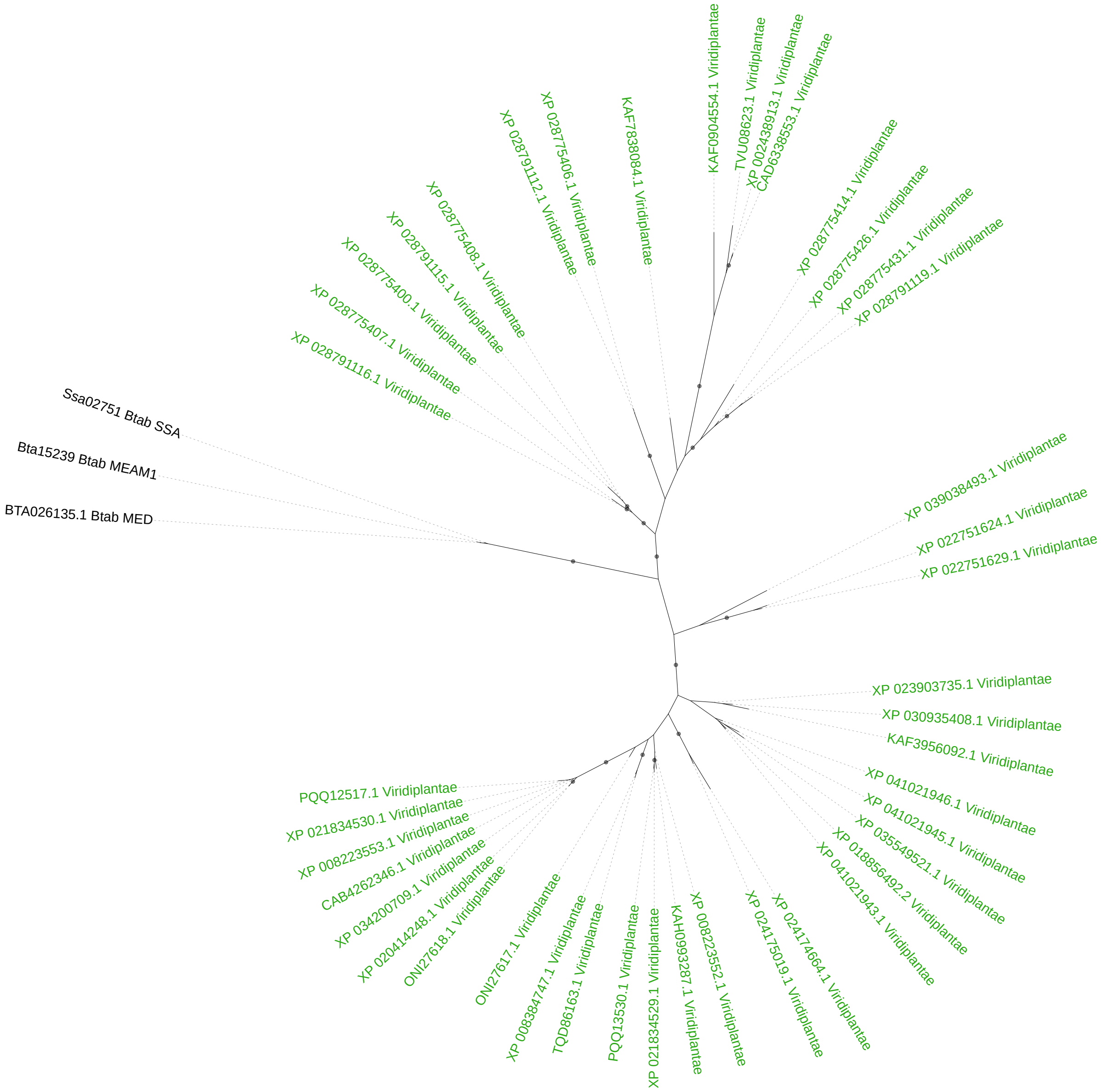

Donor: Viridiplantae
BtaV24: CE8

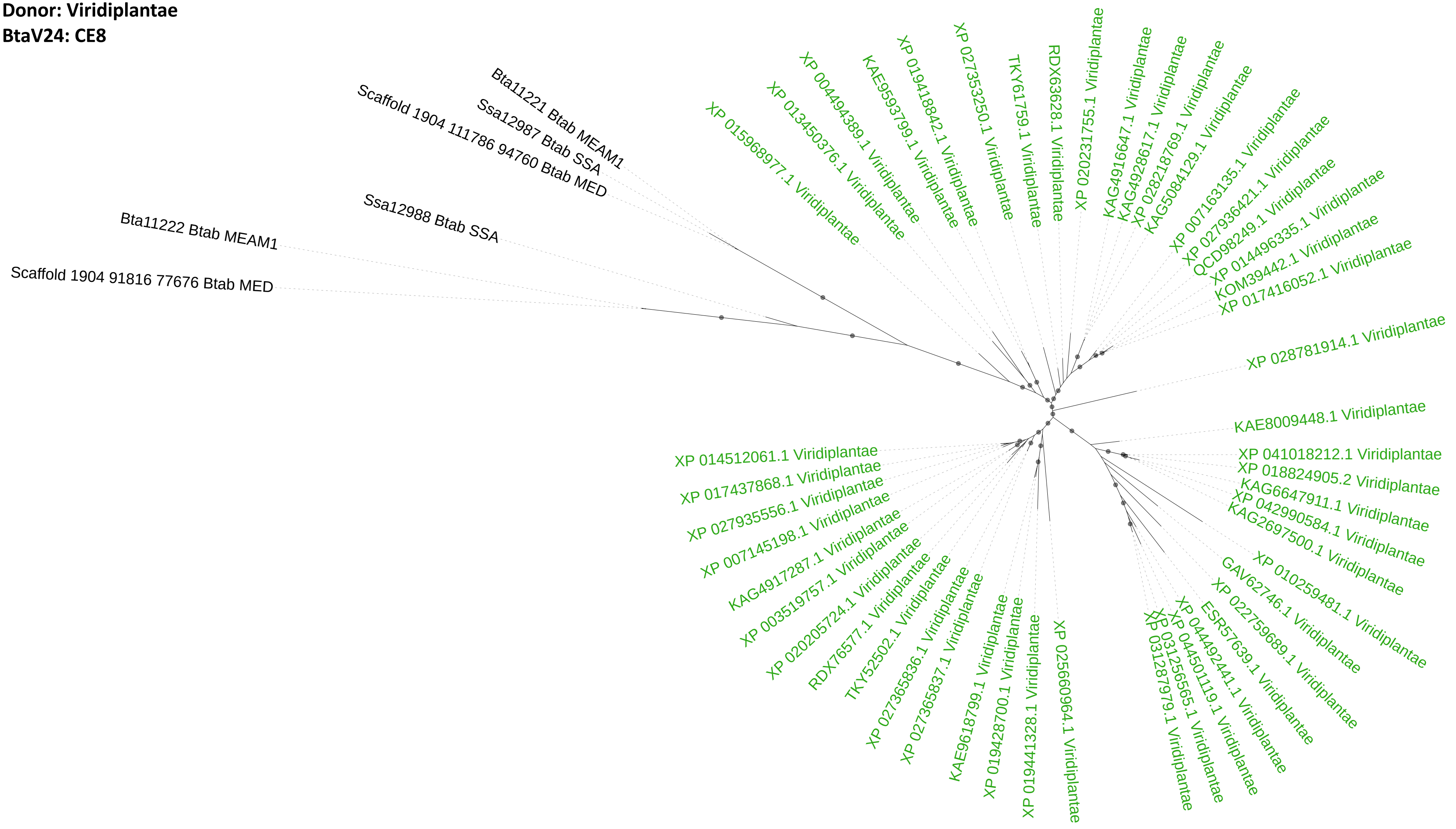

Donor: Viridiplantae
BtaV25: GT61

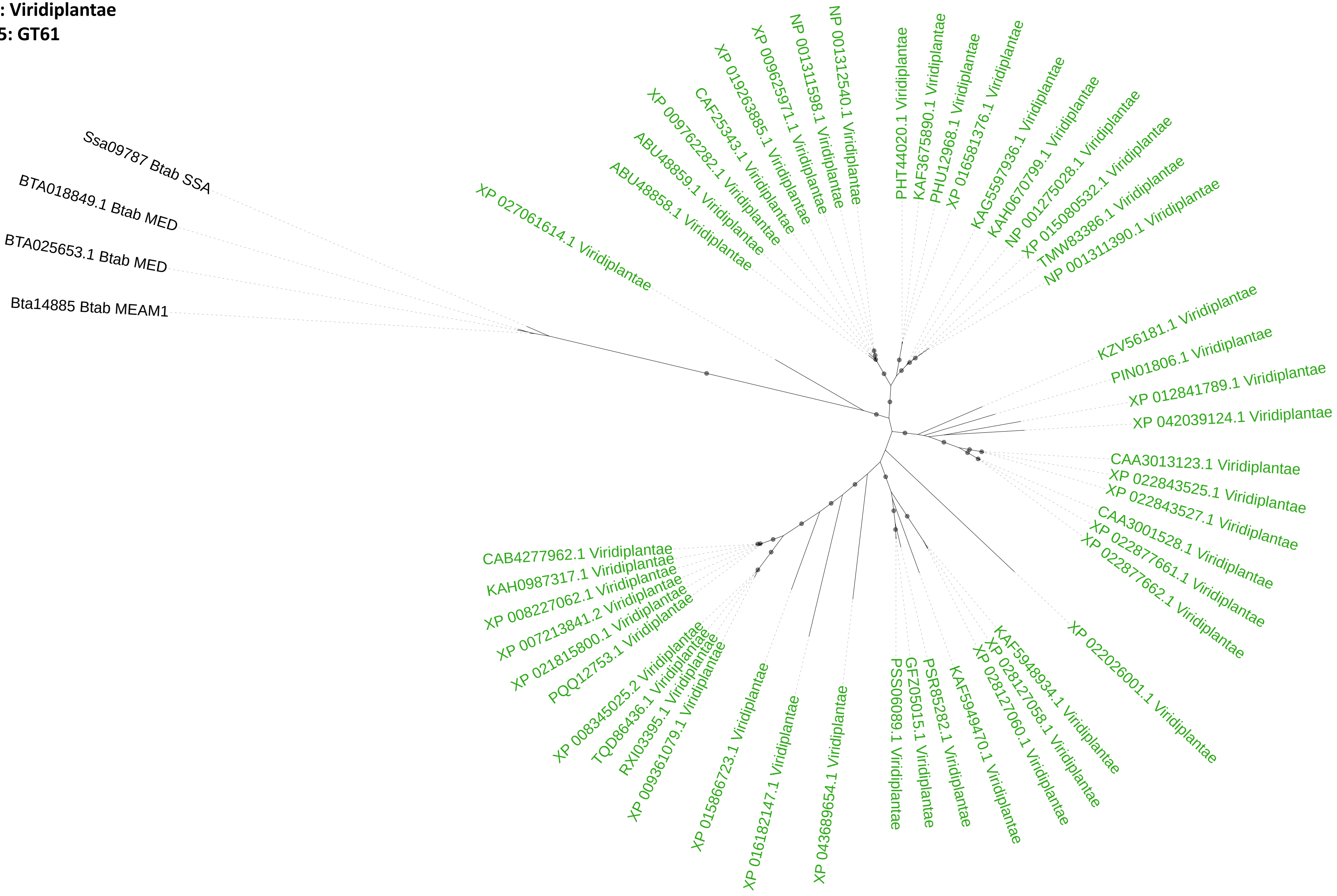

Donor: Viridiplantae
BtaV27: GT17

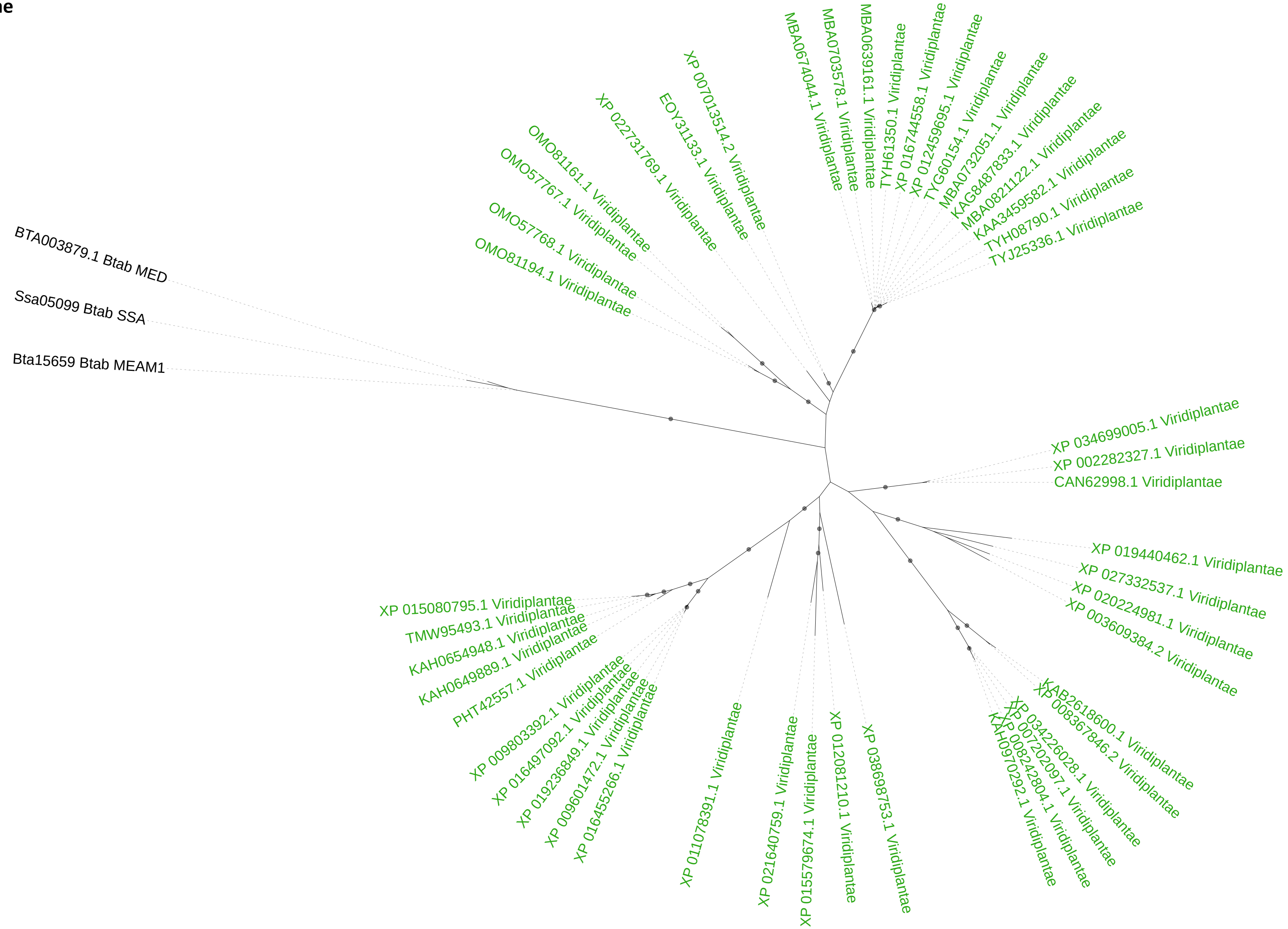

Donor: Bacteria or Fungi
BtaC01_TvaC01: PL1_4

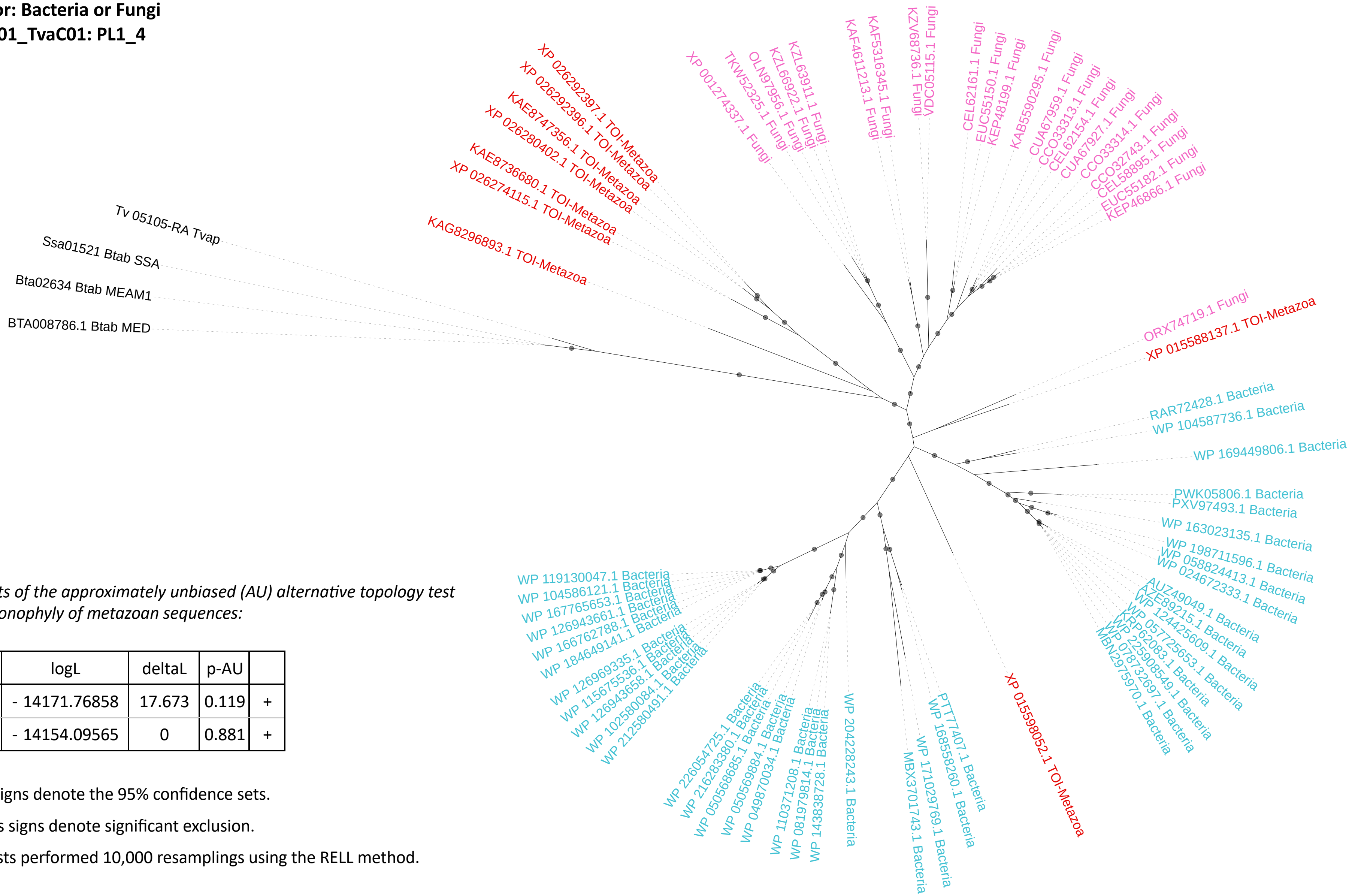

Results of the approximately unbiased (AU) alternative topology test for monophyly of metazoan sequences:

Tree	logL	deltaL	p-AU	
1	- 14171.76858	17.673	0.119	+
2	- 14154.09565	0	0.881	+

Plus signs denote the 95% confidence sets.

Minus signs denote significant exclusion.

All tests performed 10,000 resamplings using the RELL method.

Supplementary Figure 3A. Synteny analysis performed between the *B. tabaci* MEAM1, *B. tabaci* MED and *B. tabaci* SSA genomes in the region corresponding to the *B. tabaci* MEAM1 Bta06115 and Bta06118 genes. Accession numbers in green correspond to the *B. tabaci* MEAM1 Bta06115 and Bta06118 genes and their orthologs in *B. tabaci* MED and *B. tabaci* SSA.

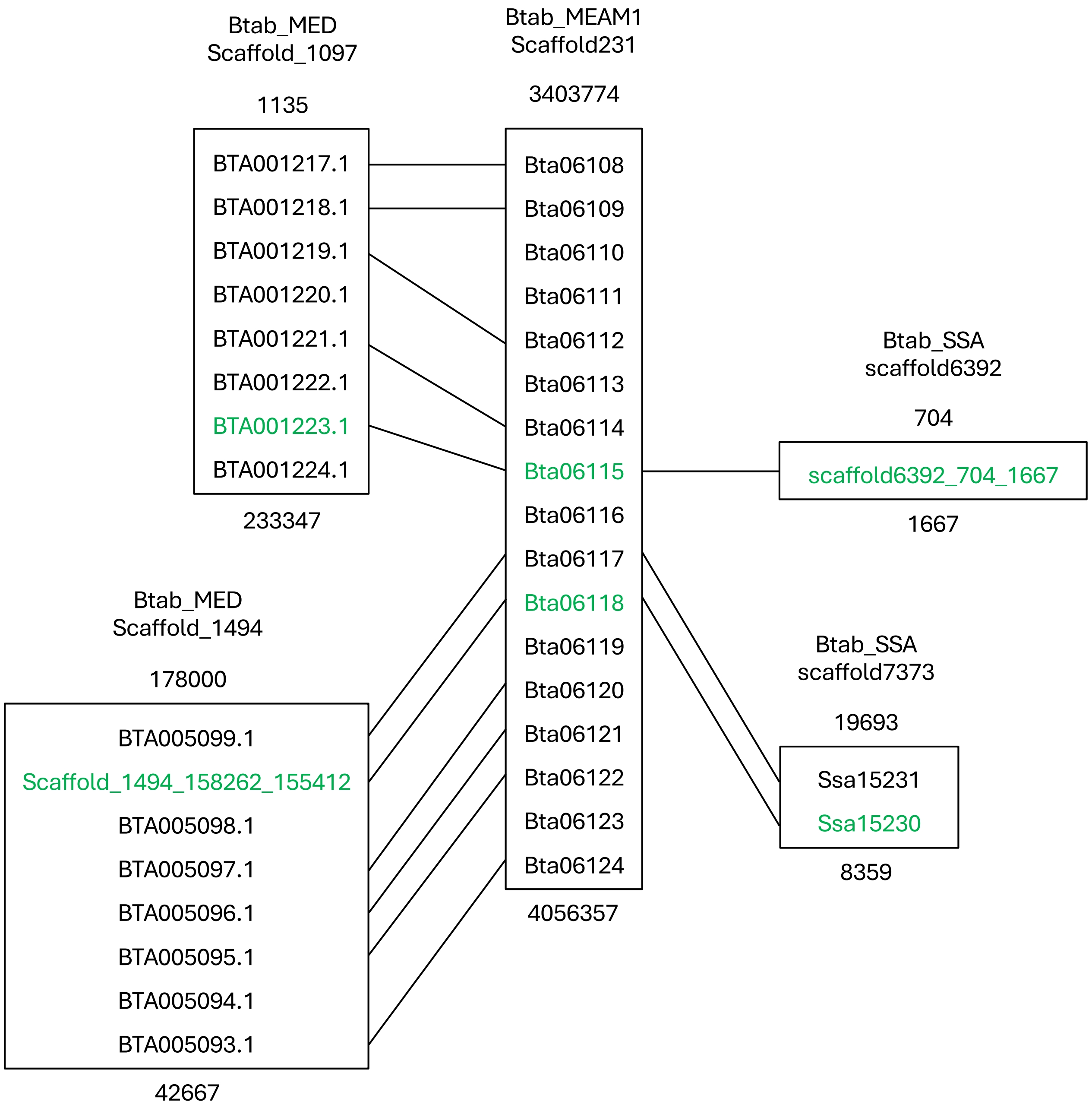

Supplementary Figure 3B. Synteny analysis performed between the *B. tabaci* MEAM1, *B. tabaci* MED and *B. tabaci* SSA genomes in the region corresponding to the *B. tabaci* MEAM1 Bta13961 gene. Accession numbers in green correspond to the *B. tabaci* MEAM1 Bta13961 gene and its orthologs in *B. tabaci* MED and *B. tabaci* SSA.

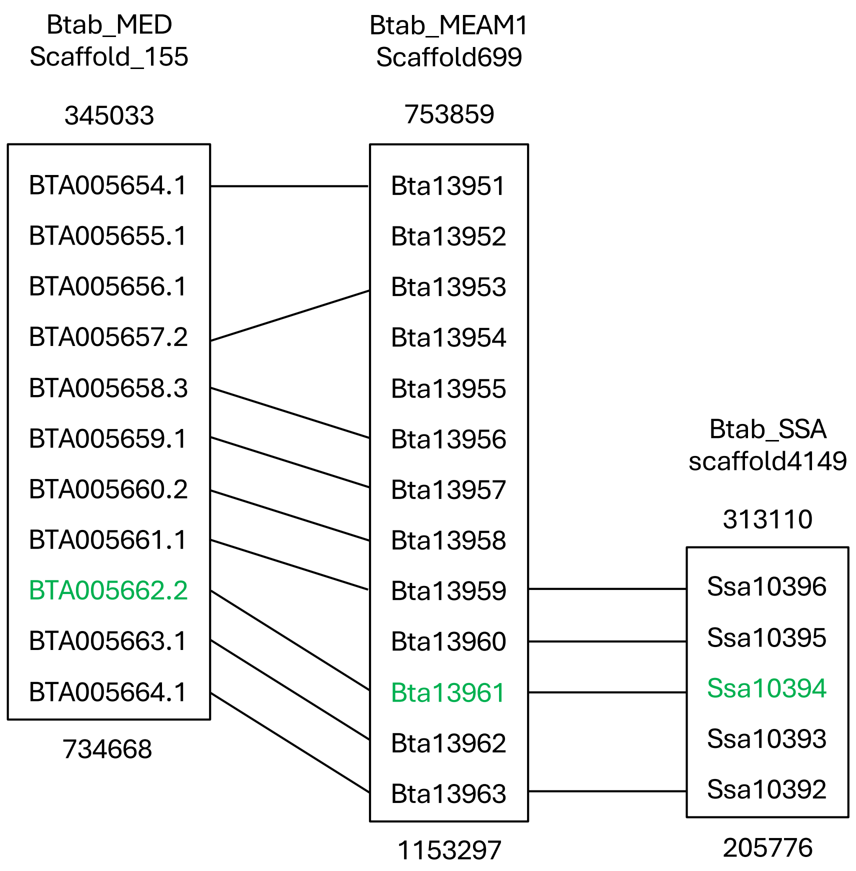

Supplementary Figure 3C. Synteny analysis performed between the *B. tabaci* MEAM1, *B. tabaci* MED and *B. tabaci* SSA genomes in the region corresponding to the *B. tabaci* MEAM1 Bta11221 and Bta11222 genes. Accession numbers in green correspond to the *B. tabaci* MEAM1 Bta11221 and Bta11222 genes and their orthologs in *B. tabaci* MED and *B. tabaci* SSA.

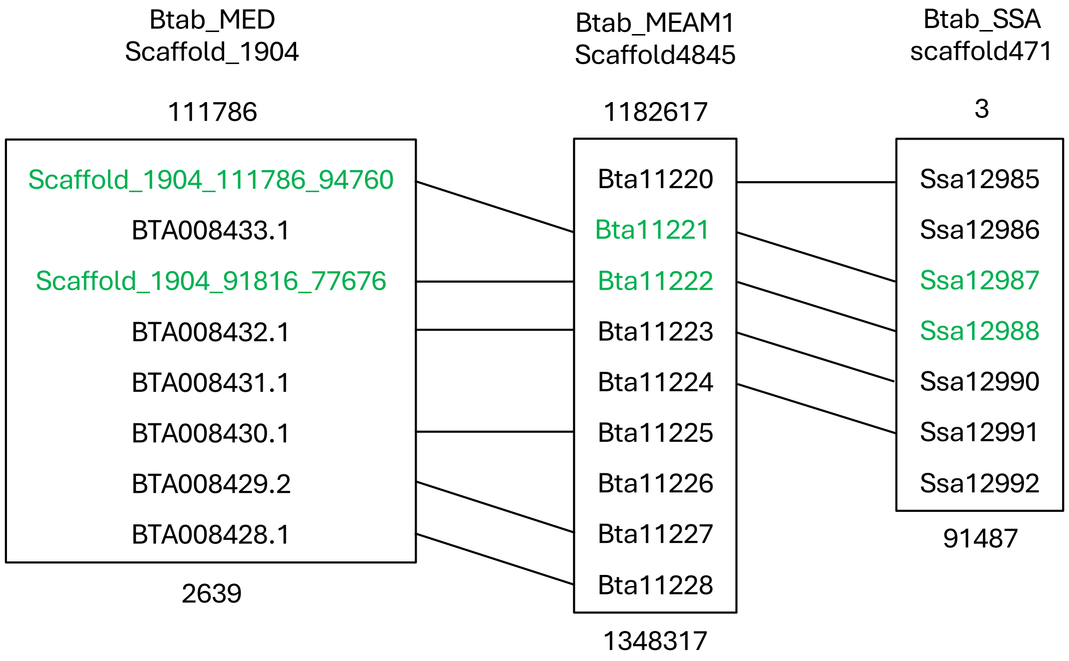

Supplementary Figure 3D. Synteny analysis performed between the *B. tabaci* MEAM1, *B. tabaci* MED and *B. tabaci* SSA genomes in the region corresponding to the *B. tabaci* MEAM1 Bta15239 gene. Accession numbers in green correspond to the *B. tabaci* MEAM1 Bta15239 gene and its orthologs in *B. tabaci* MED and *B. tabaci* SSA.

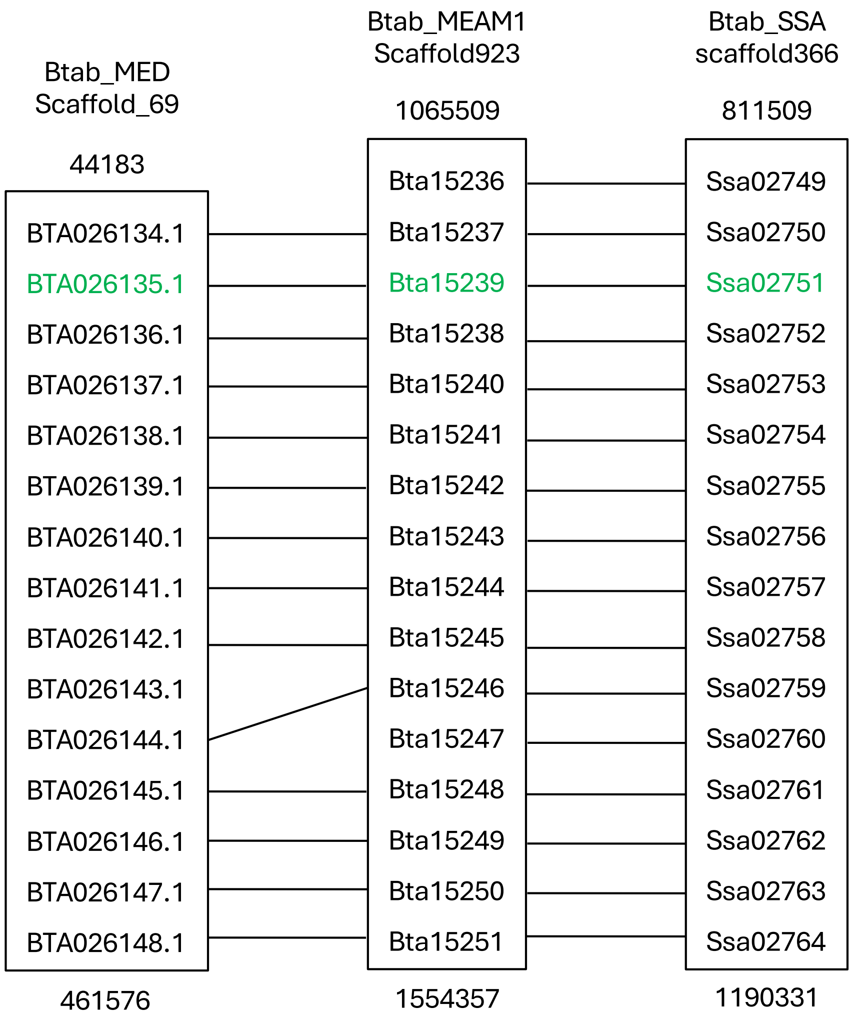

Supplementary Figure 3E. Synteny analysis performed between the *B. tabaci* MEAM1 and *T. vaporariorum* genomes in the regions corresponding to the *B. tabaci* MEAM1 Bta13961 gene and to the *T. vaporariorum* Tv_15928-RA gene, respectively. The accession number in green corresponds to the *B. tabaci* MEAM1 Bta13961 gene. The accession number in red corresponds to the *T. vaporariorum* Tv_15928-RA gene.

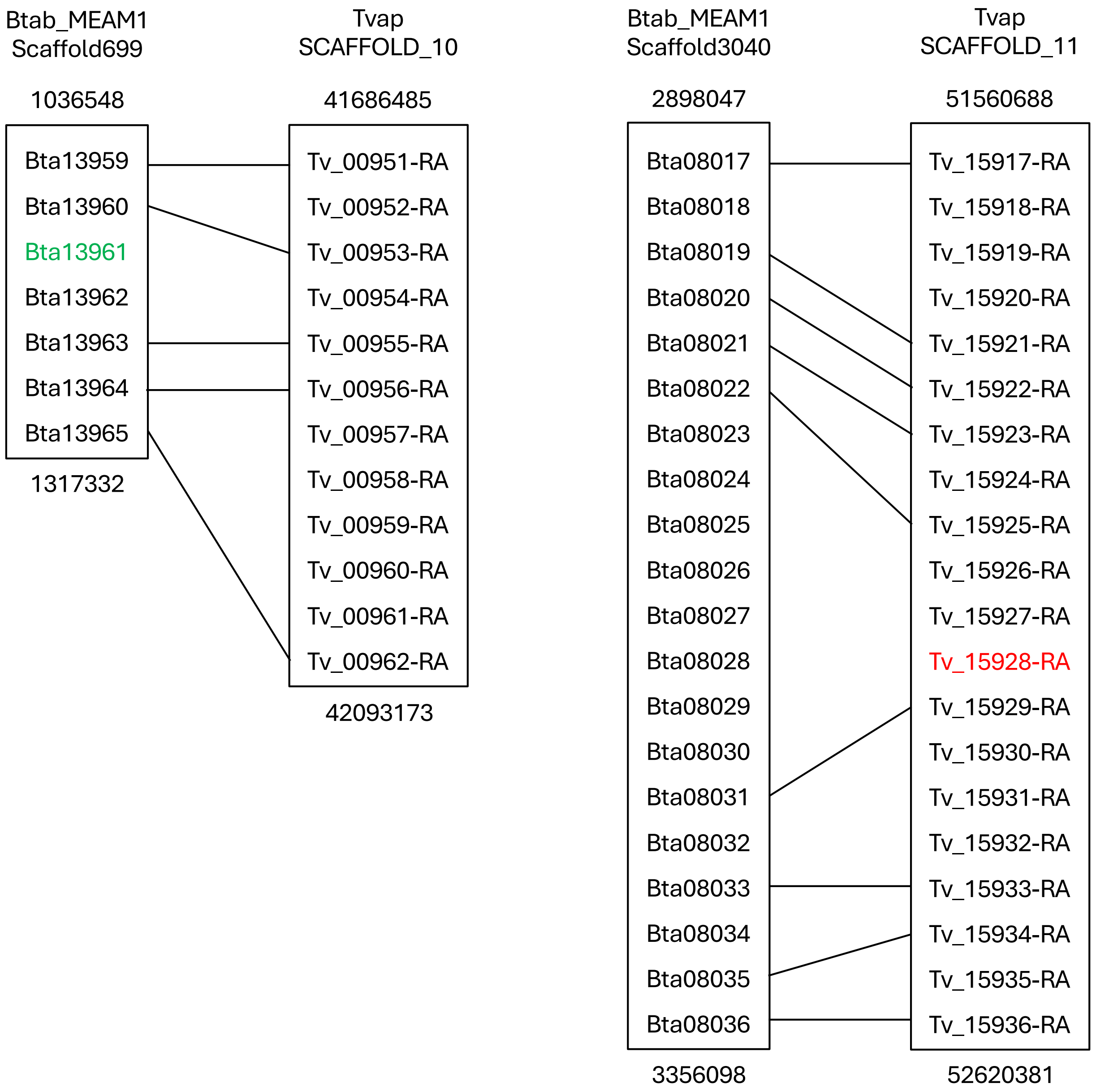

Supplementary Figure 4. Comparison of the predicted structures of Bta06115 and Bta06118 proteins with the known structures of barley (1-3)-ß-glucanase and barley (1- 3,1-4)-ß-glucanase. The α-helices are colored red, ß-sheets are yellow, and loops are green.

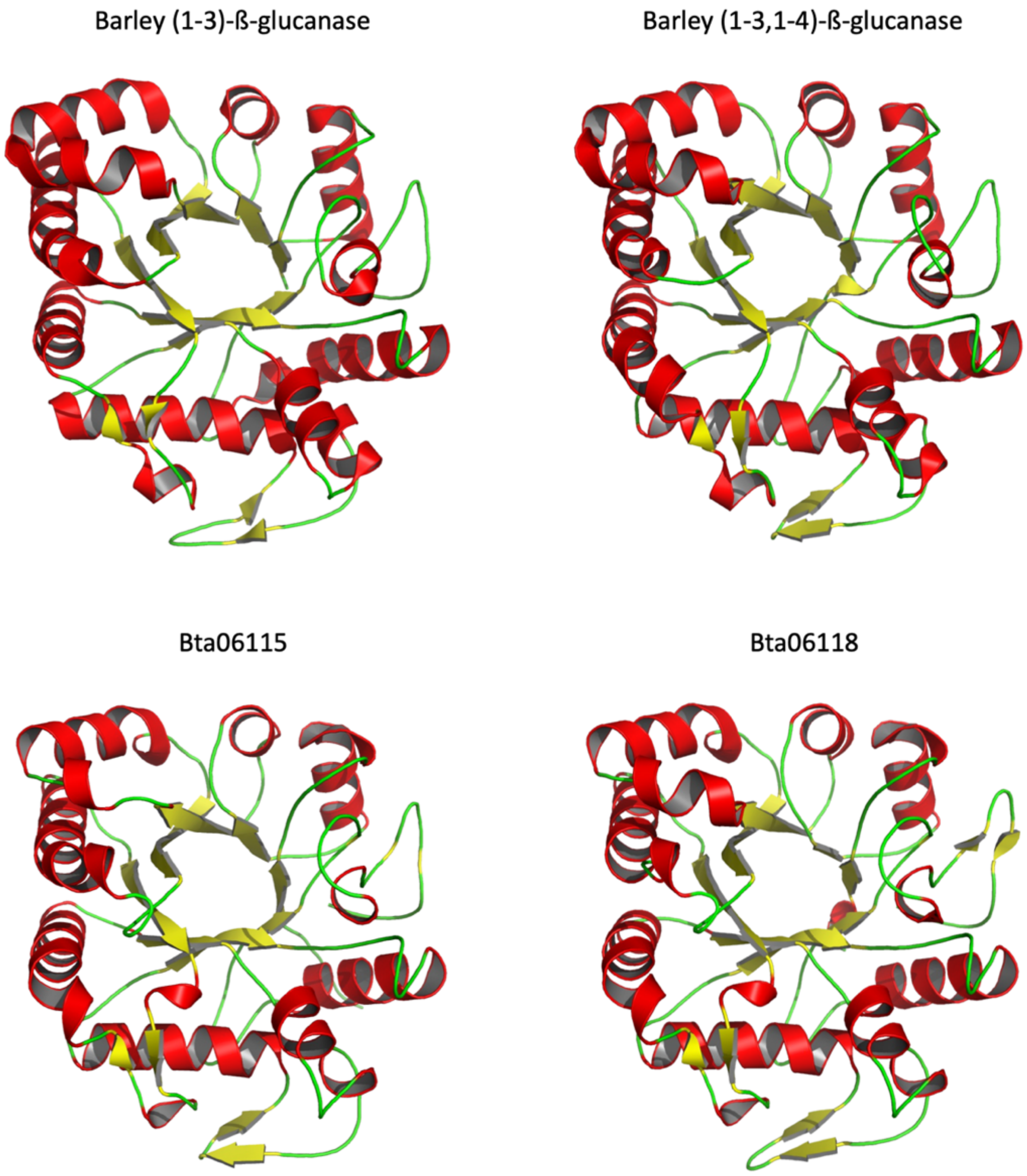

Supplementary Figure 5. Comparison of the predicted structures of the pectinesterase active part of Bta11221 and Bta11222 proteins with the known structure of carrot PME. (A) Alignment of Bta11221 (blue) and carrot PME (orange) structures. (B) Alignment of Bta11222 (green) and carrot PME (orange) structures. (C) Comparison of the active site for carrot PME, Bta11221 and Bta11222. The five catalytically important residues for carrot PME (Gln113, Gln135, Asp136, Asp157 and Arg225) and the corresponding residues in Bta11221 and Bta11222 are shown as balls and sticks.

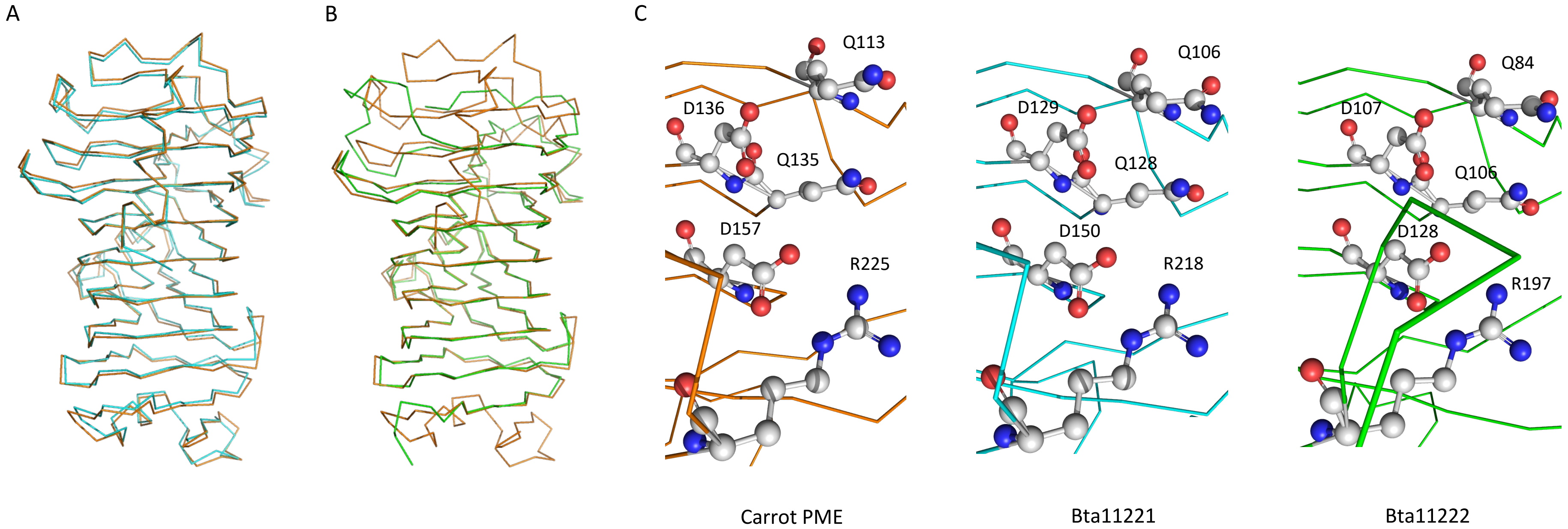

Supplementary Figure 6.

Phylogeny inferred for the 11 combined groups in which the sequences of *B. tabaci* and *T. vaporariorum* formed monophyletic clades with support values greater than or equal to 80% and 95% for SH-aLRT and UFboot, respectively.

Donor: Bacteria

TvaB06_BtaB09

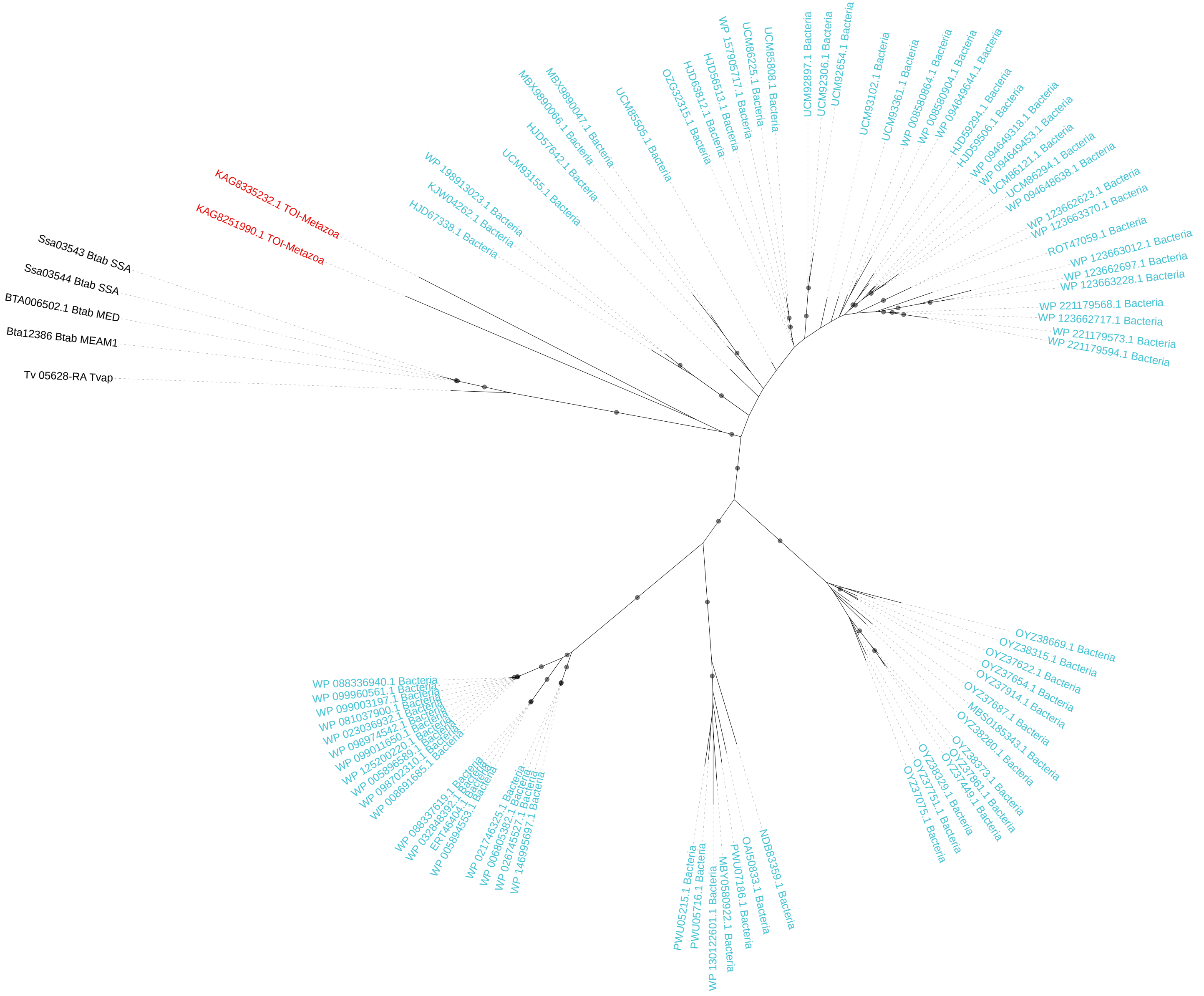

Donor: Bacteria
TvaB07_BtaB36

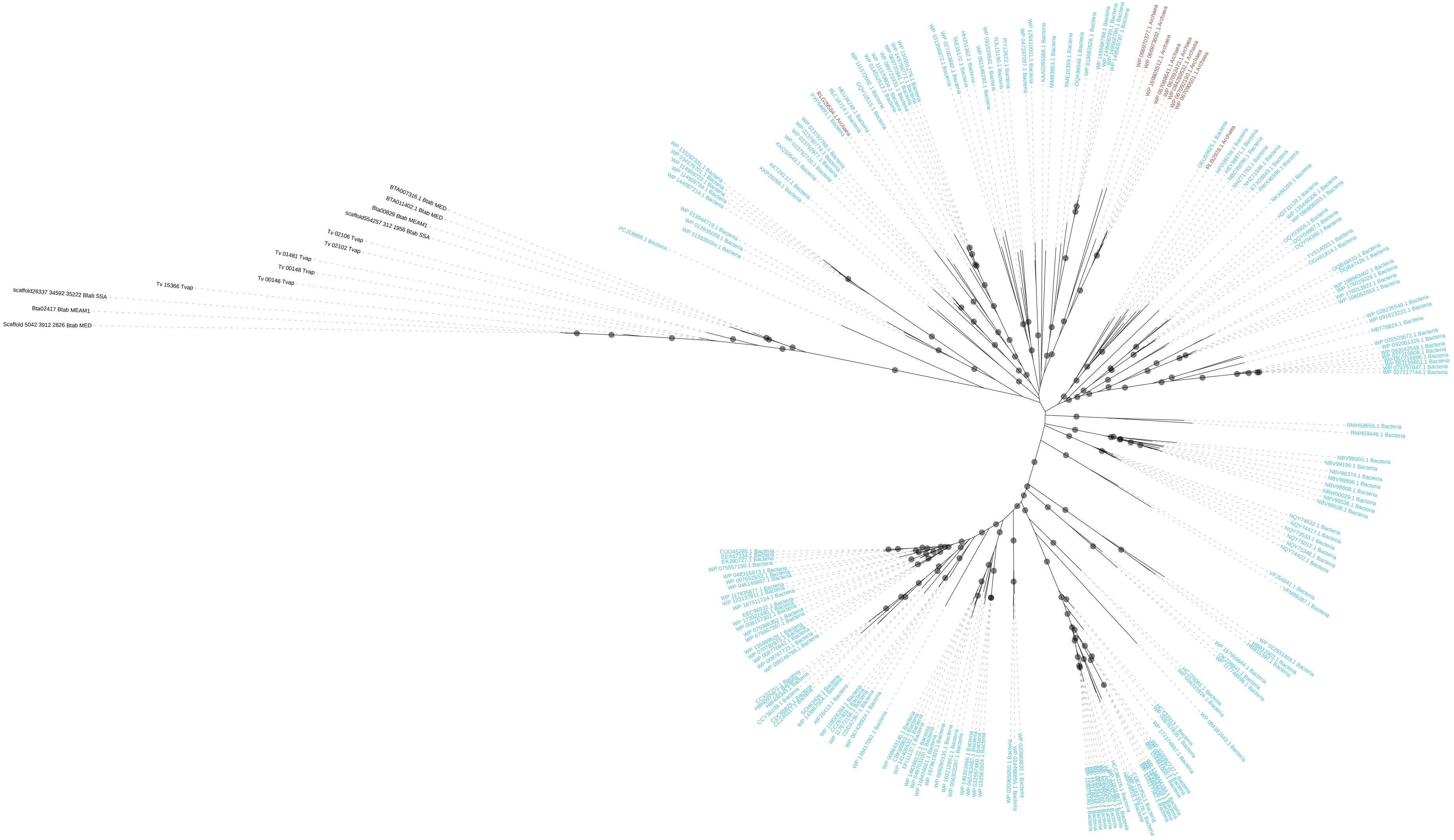

Donor: Bacteria
TvaB16_BtaB45

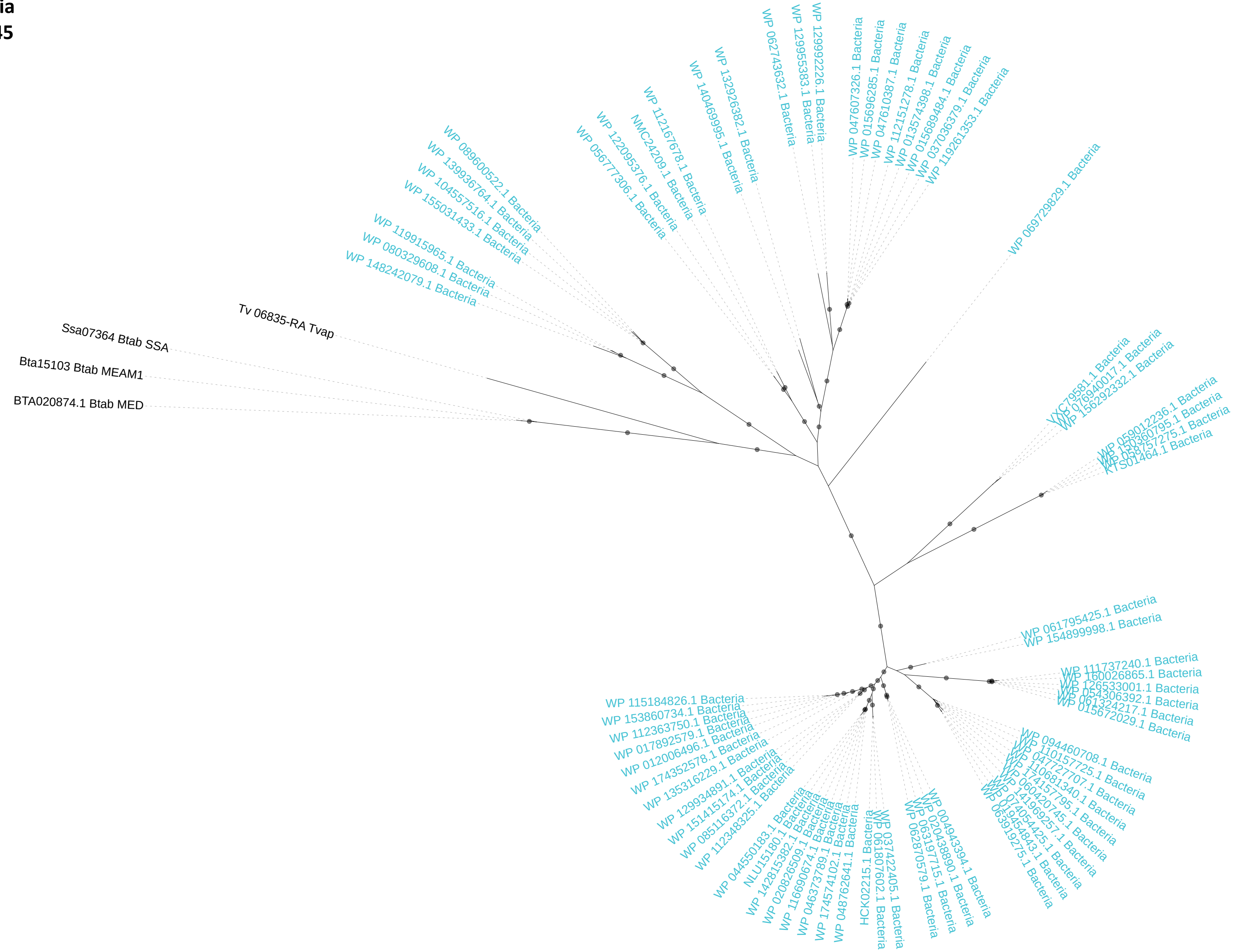

Donor: Bacteria
TvaB17_BtaB65

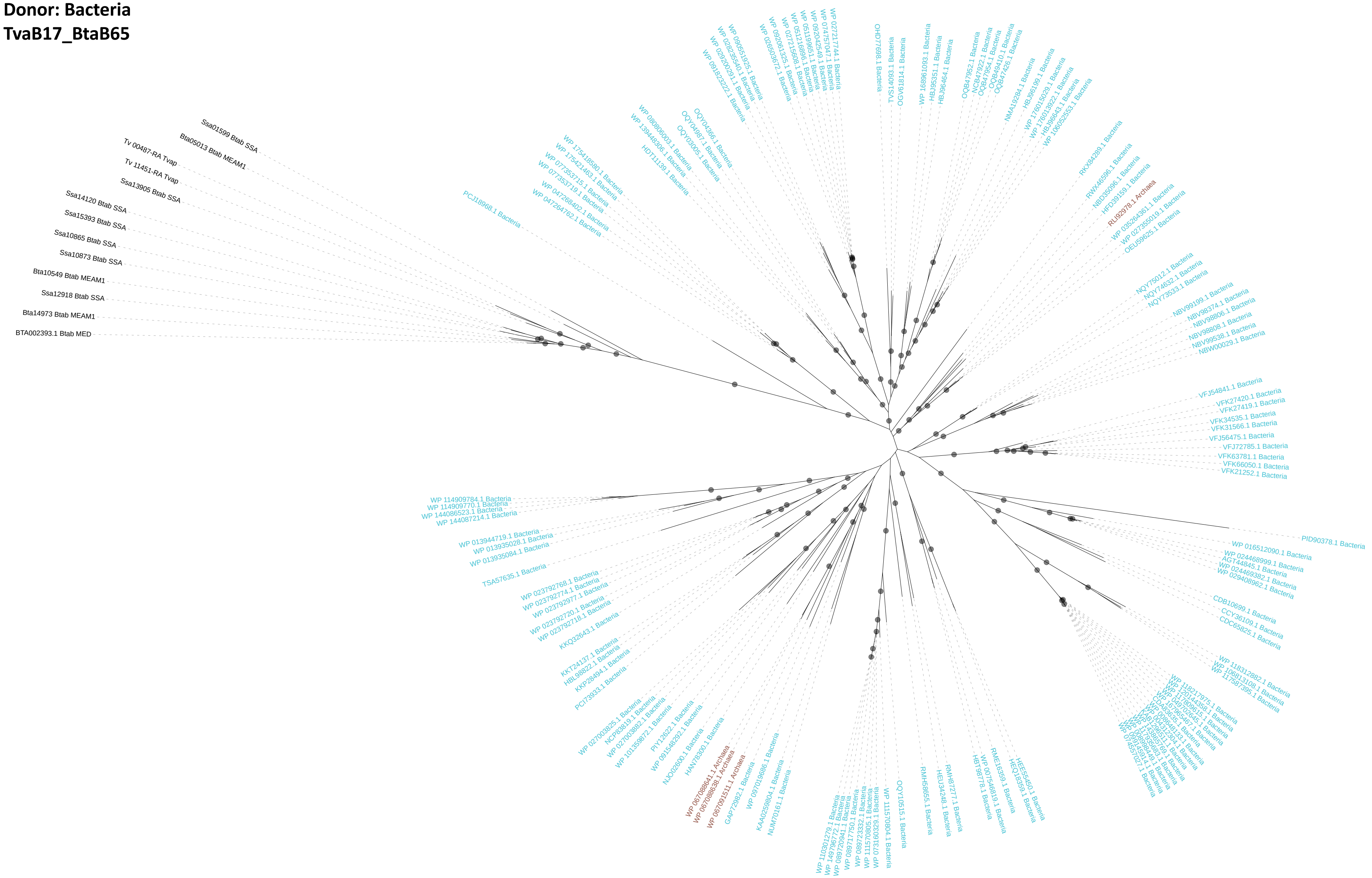

Donor: Bacteria

TvaB19_BtaB01

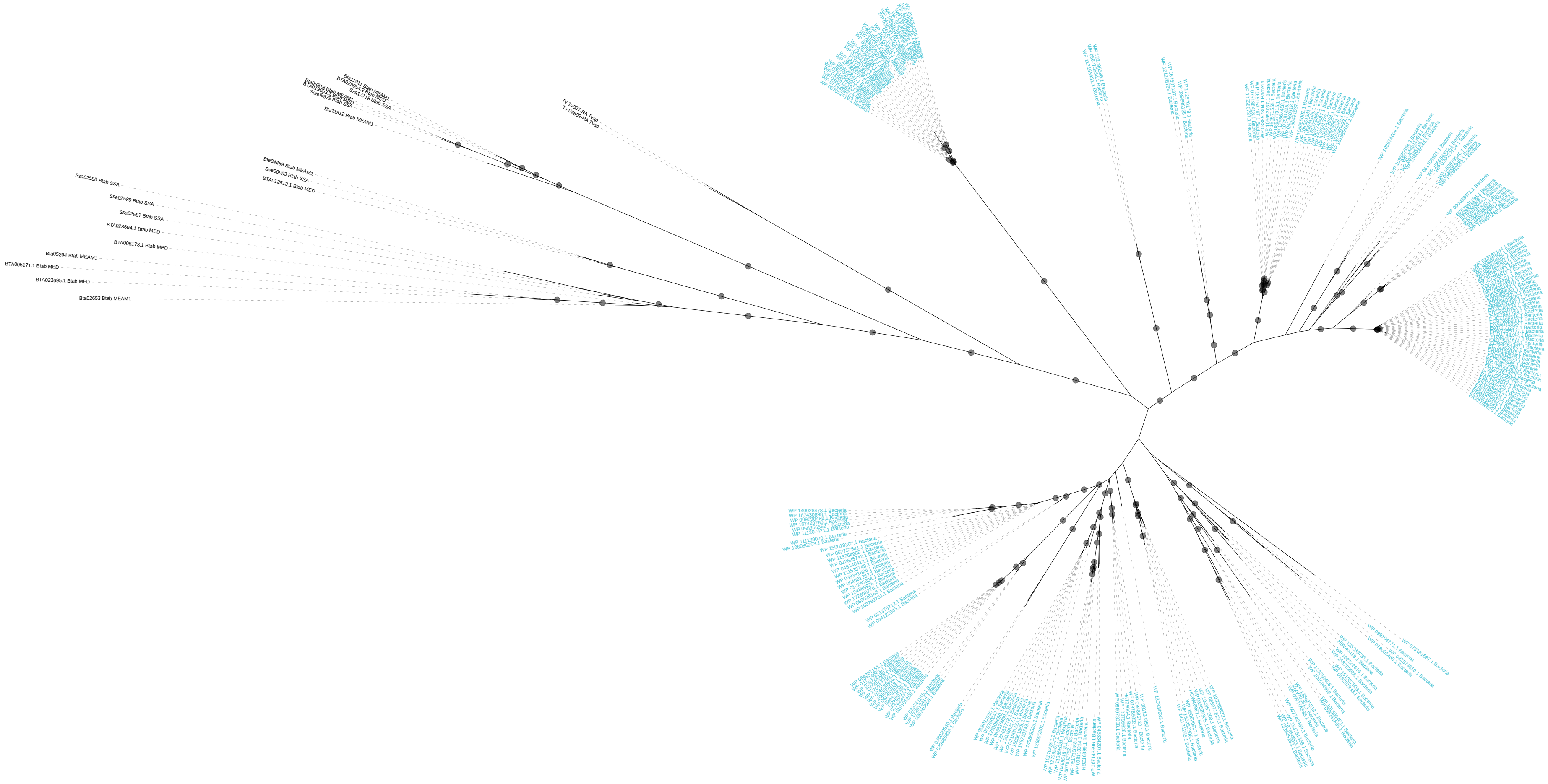

Donor: Bacteria
TvaB20_BtaB11

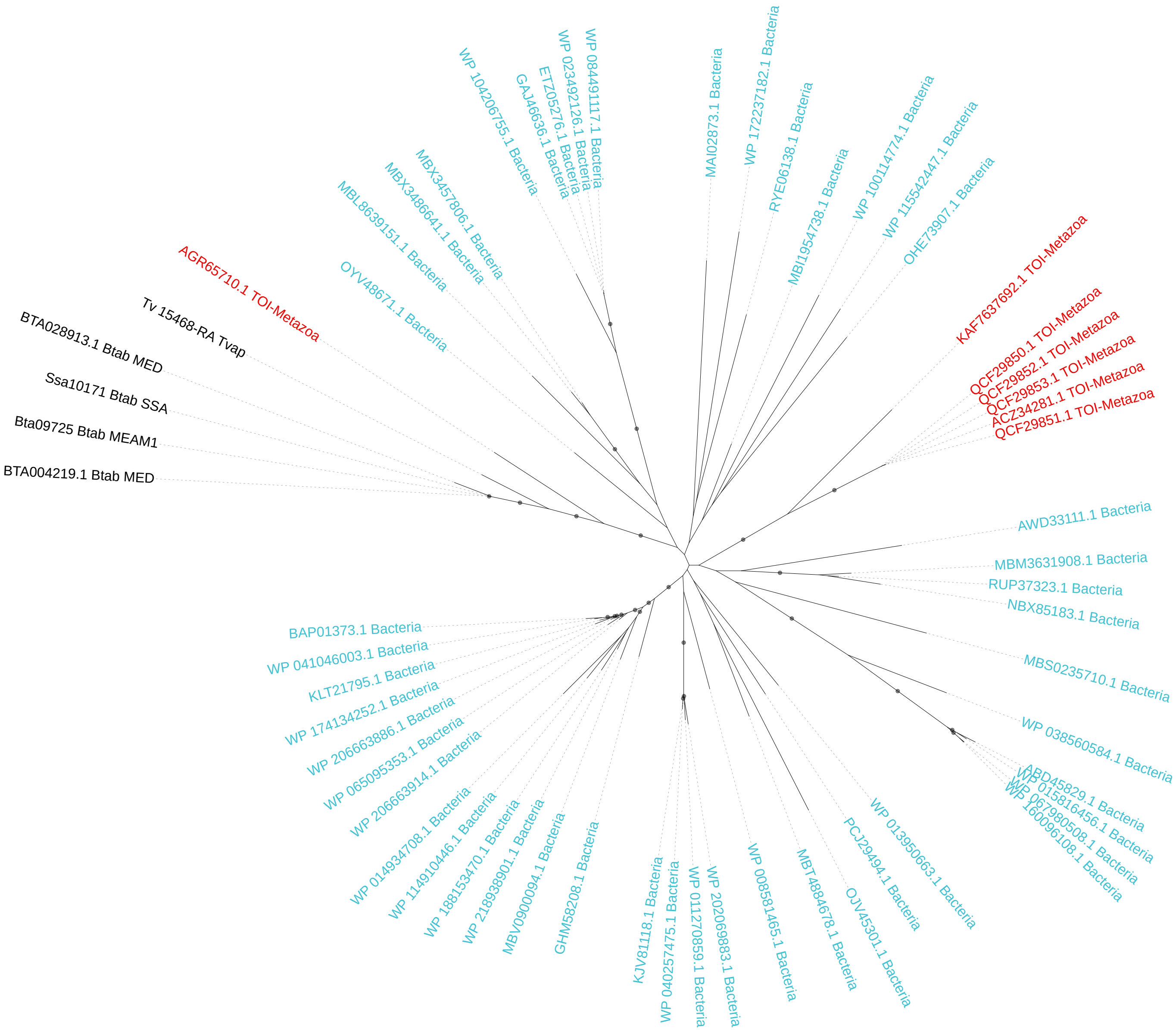

Donor: Bacteria

TvaB22_BtaB35

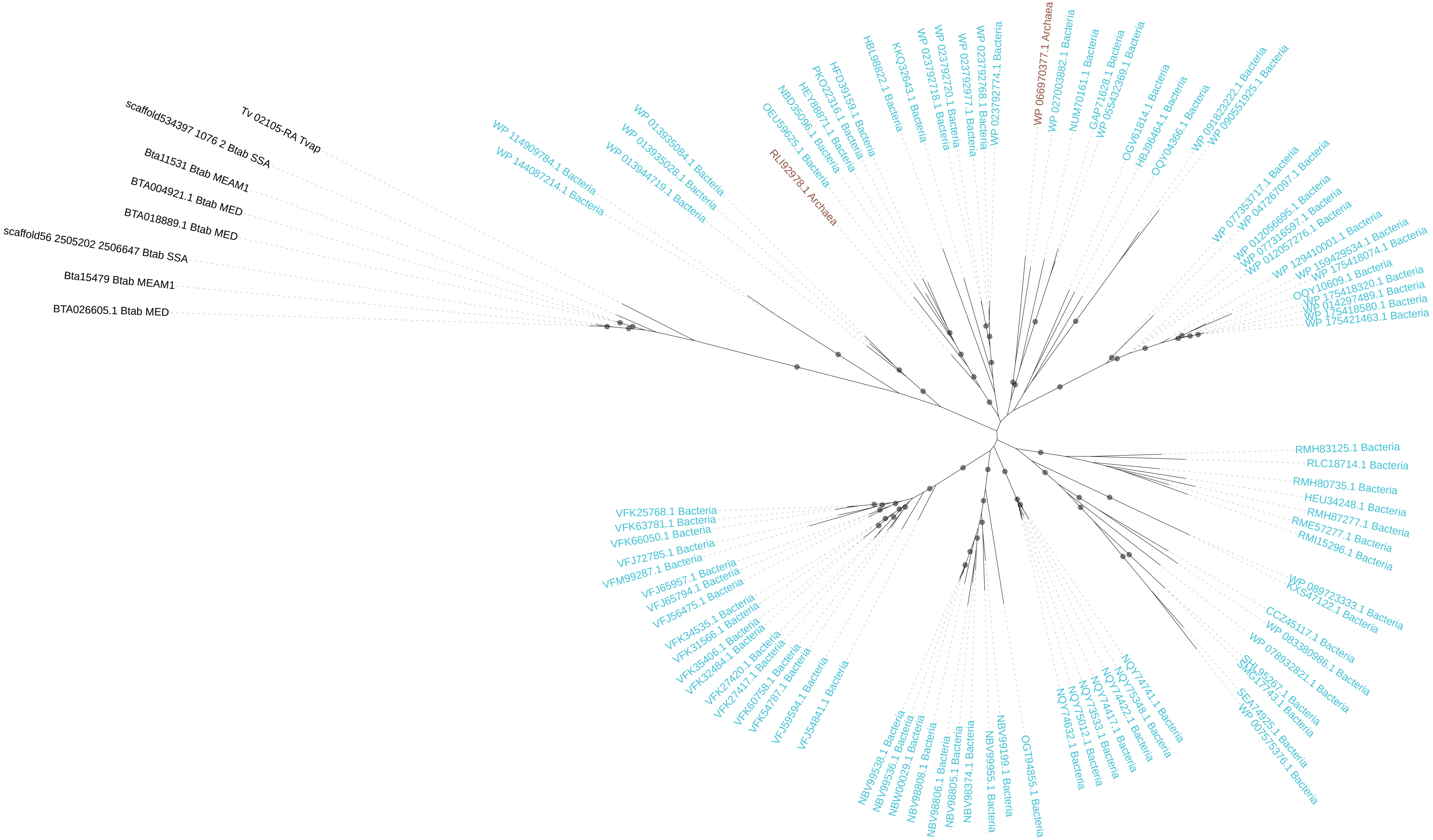

Donor: Fungi
TvaF01_BtaF22

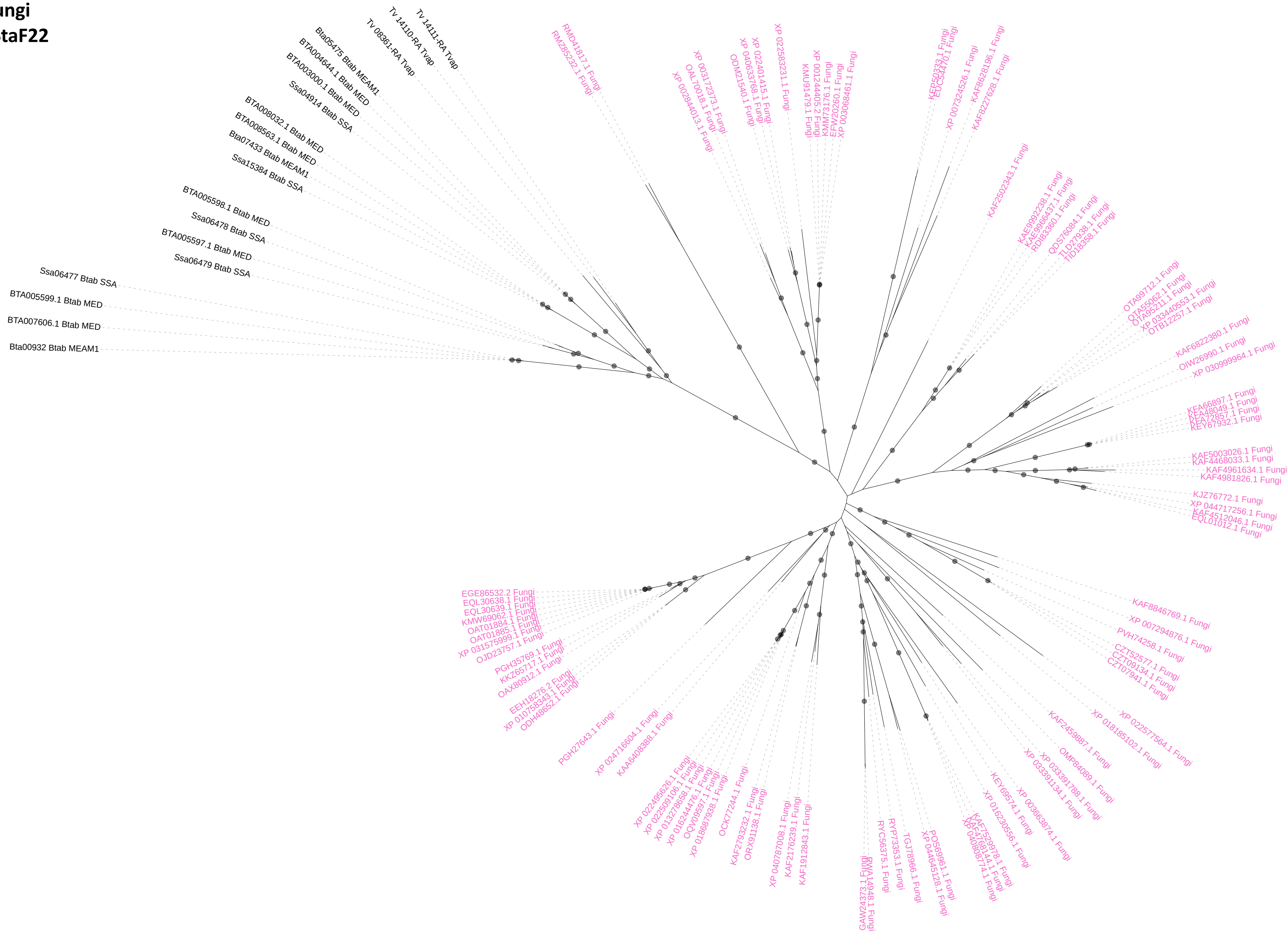

Donor: Fungi
TvaF02_BtaF37

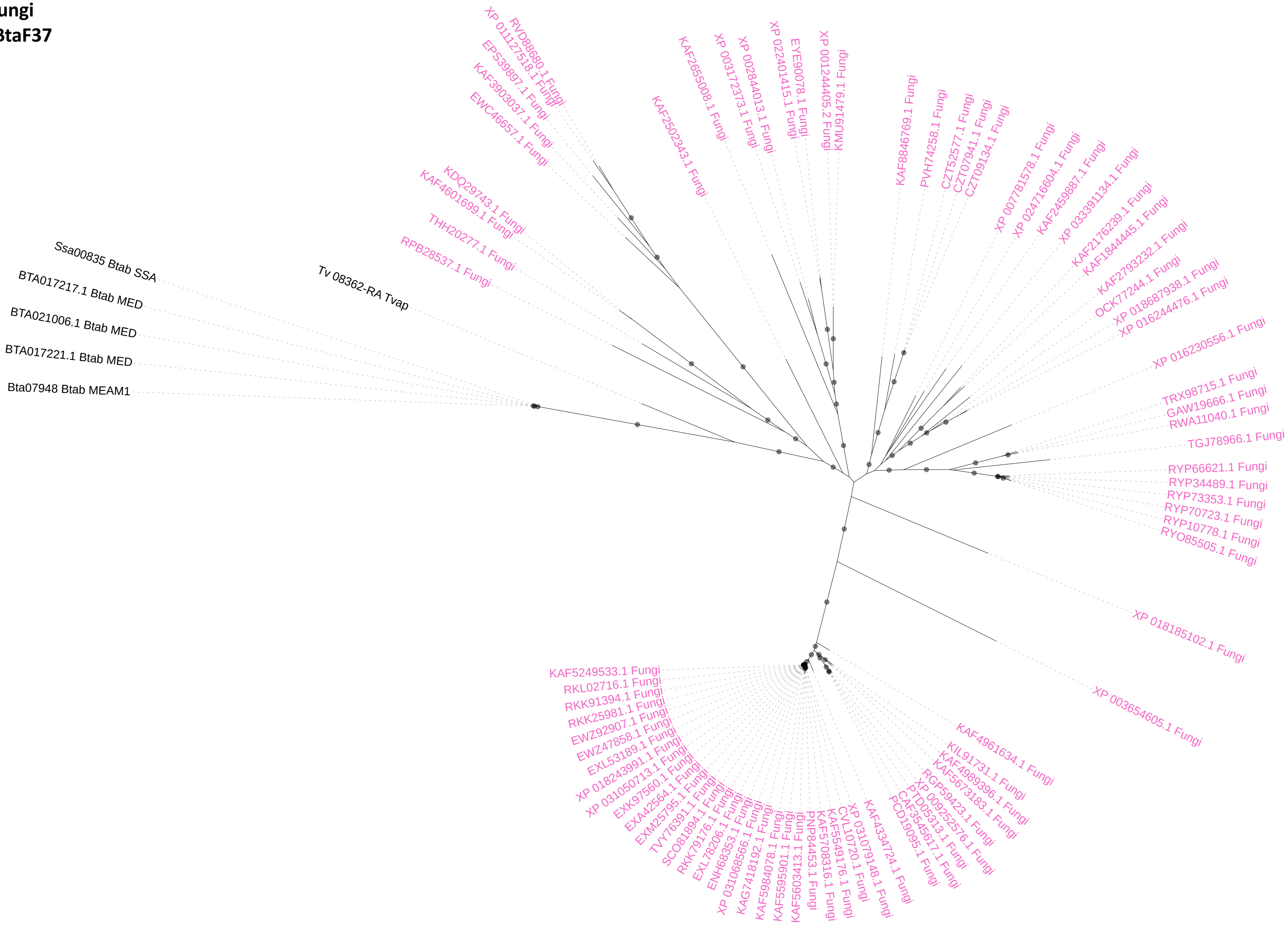

Donor: Fungi
TvaF03_BtaF26

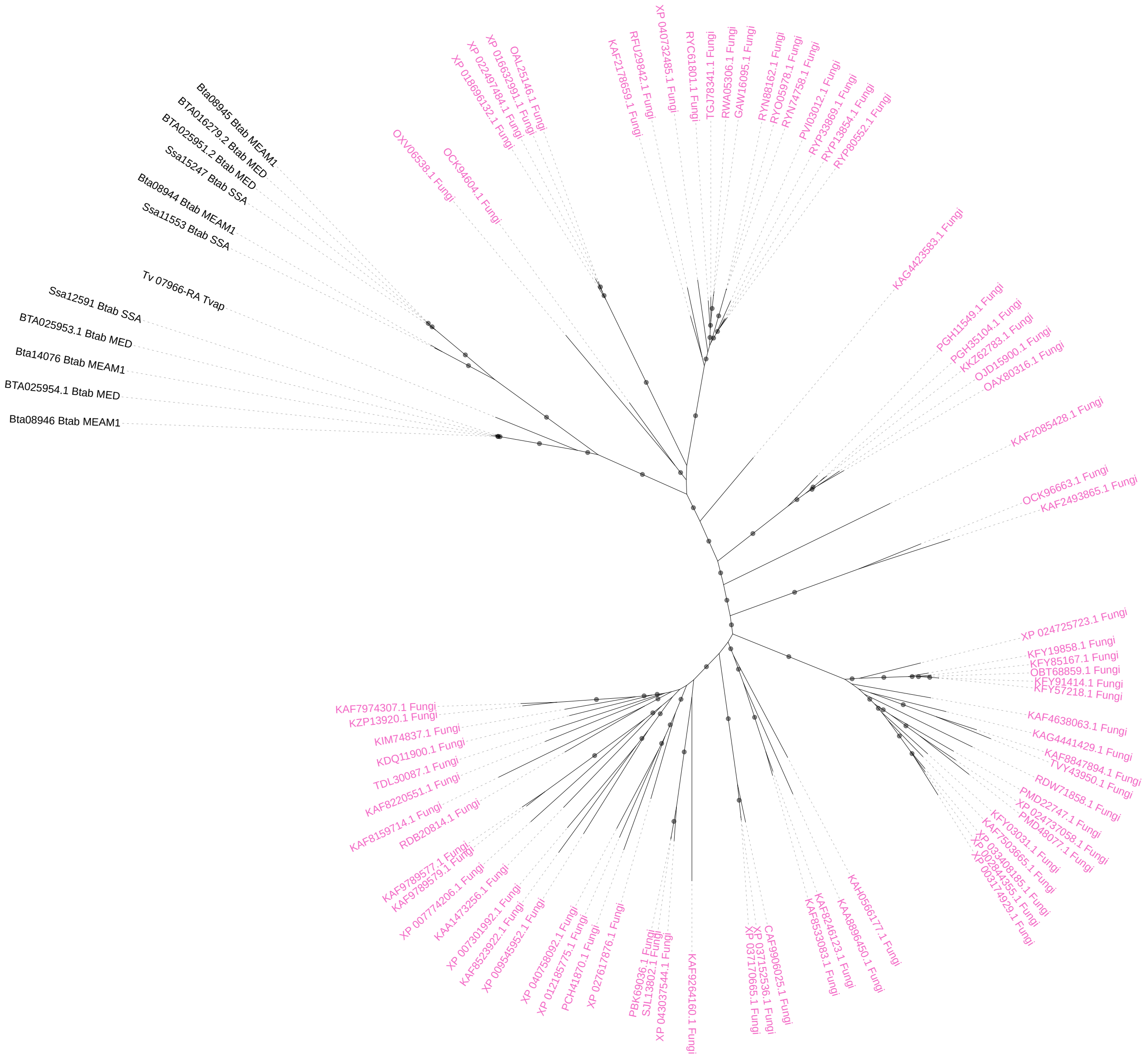

Donor: Bacteria or Fungi
TvaC01_BtaC01

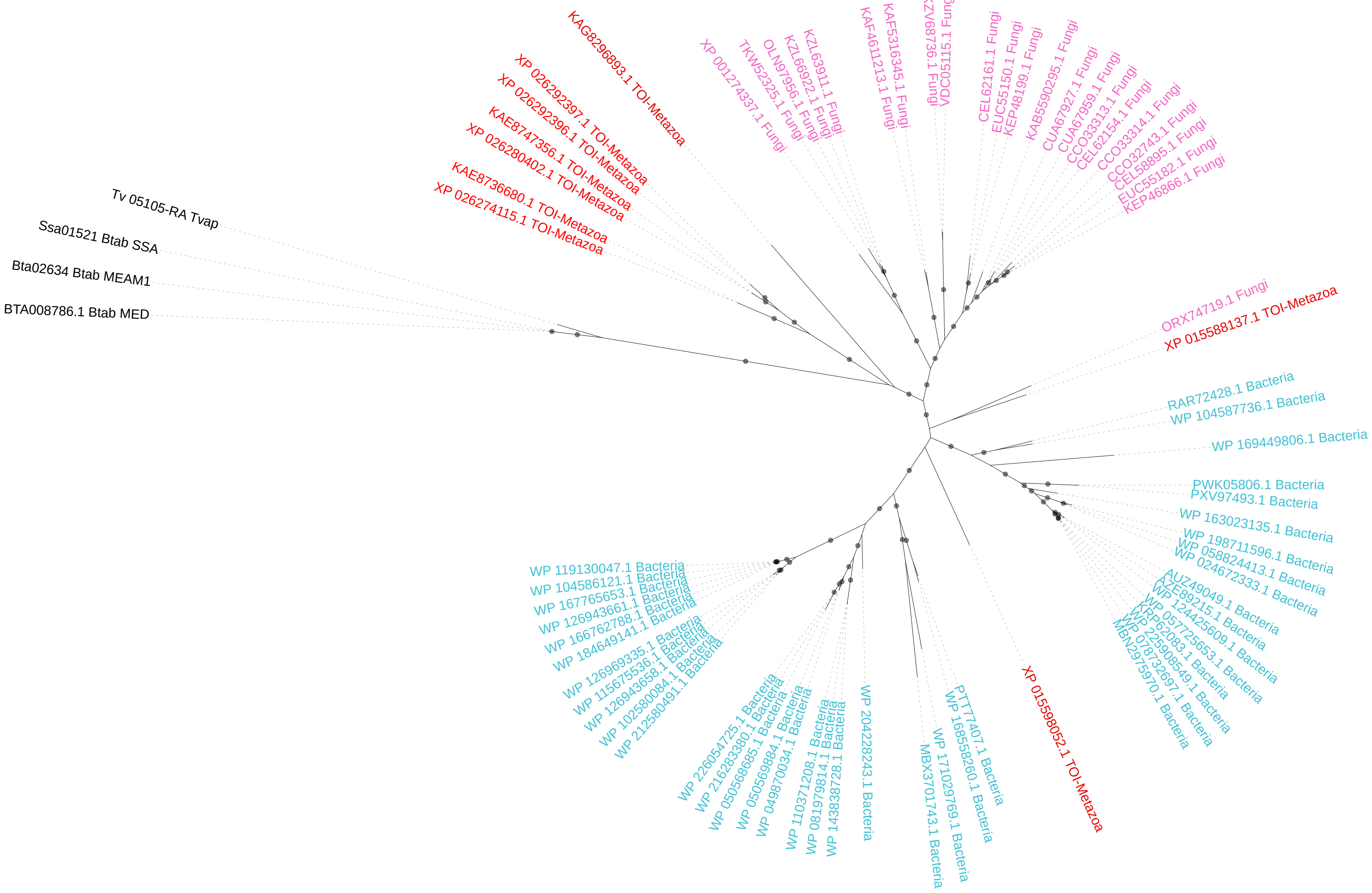

Supplementary Figure 7.

Phylogeny inferred for the 12 combined groups in which the grouping of sequences of *B. tabaci* and *T. vaporariorum* was not supported by bootstrap analysis and/or not monophyletic.

Donor: Bacteria

TvaB01_BtaB56

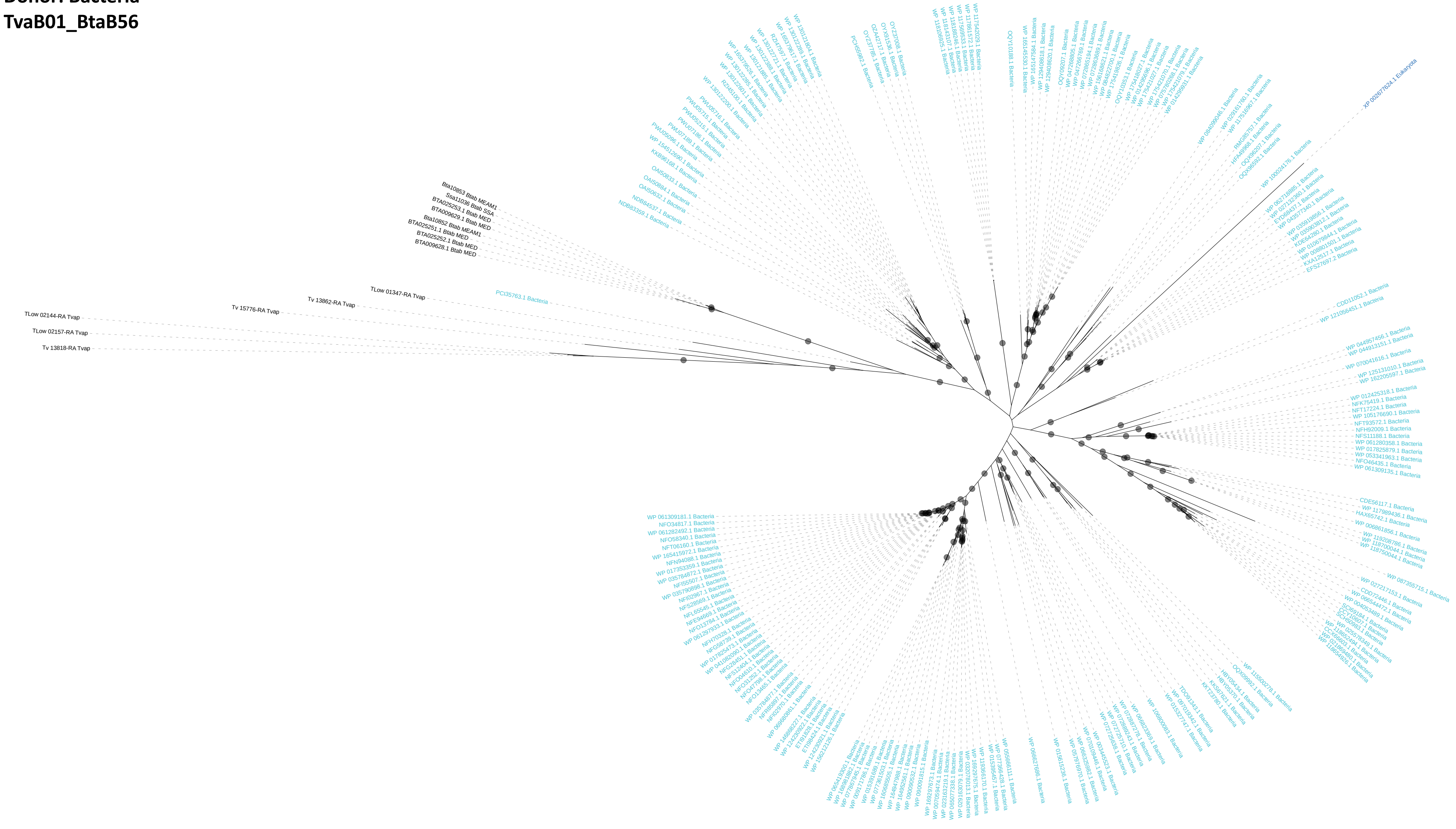

Donor: Bacteria
TvaB02_BtaB12

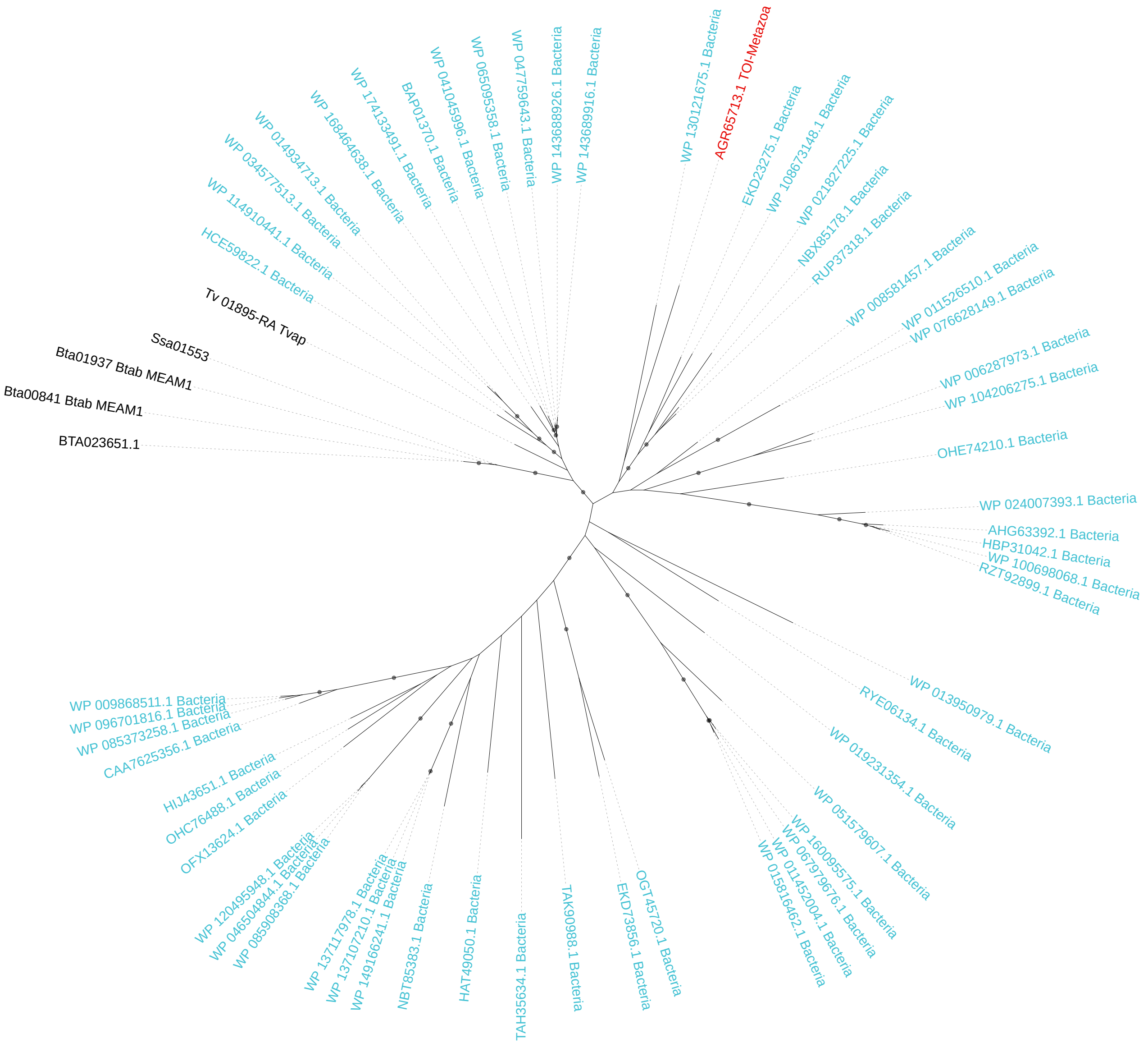

Donor: Bacteria
TvaB03_BtaB68

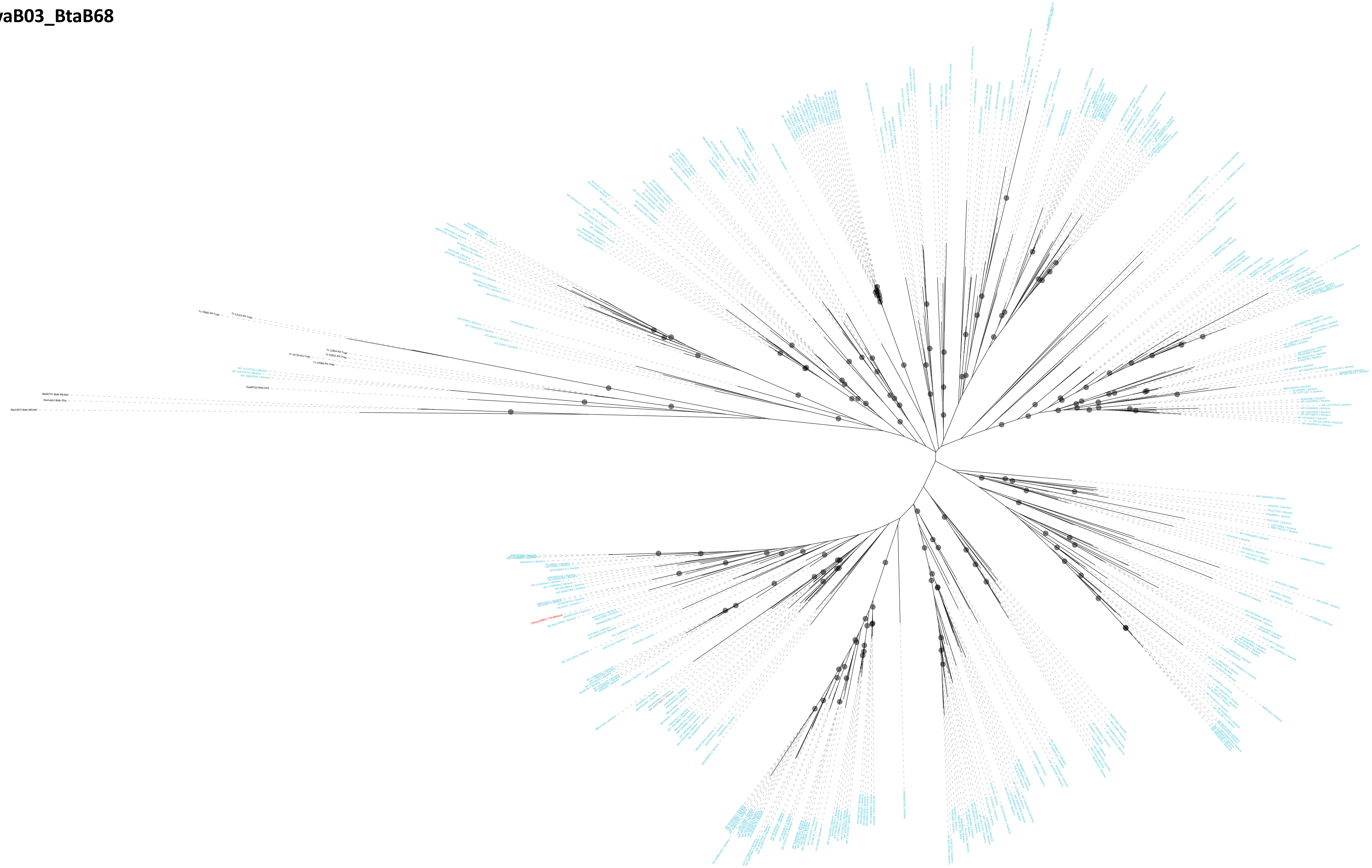

Donor: Bacteria

TvaB04_BtaB44

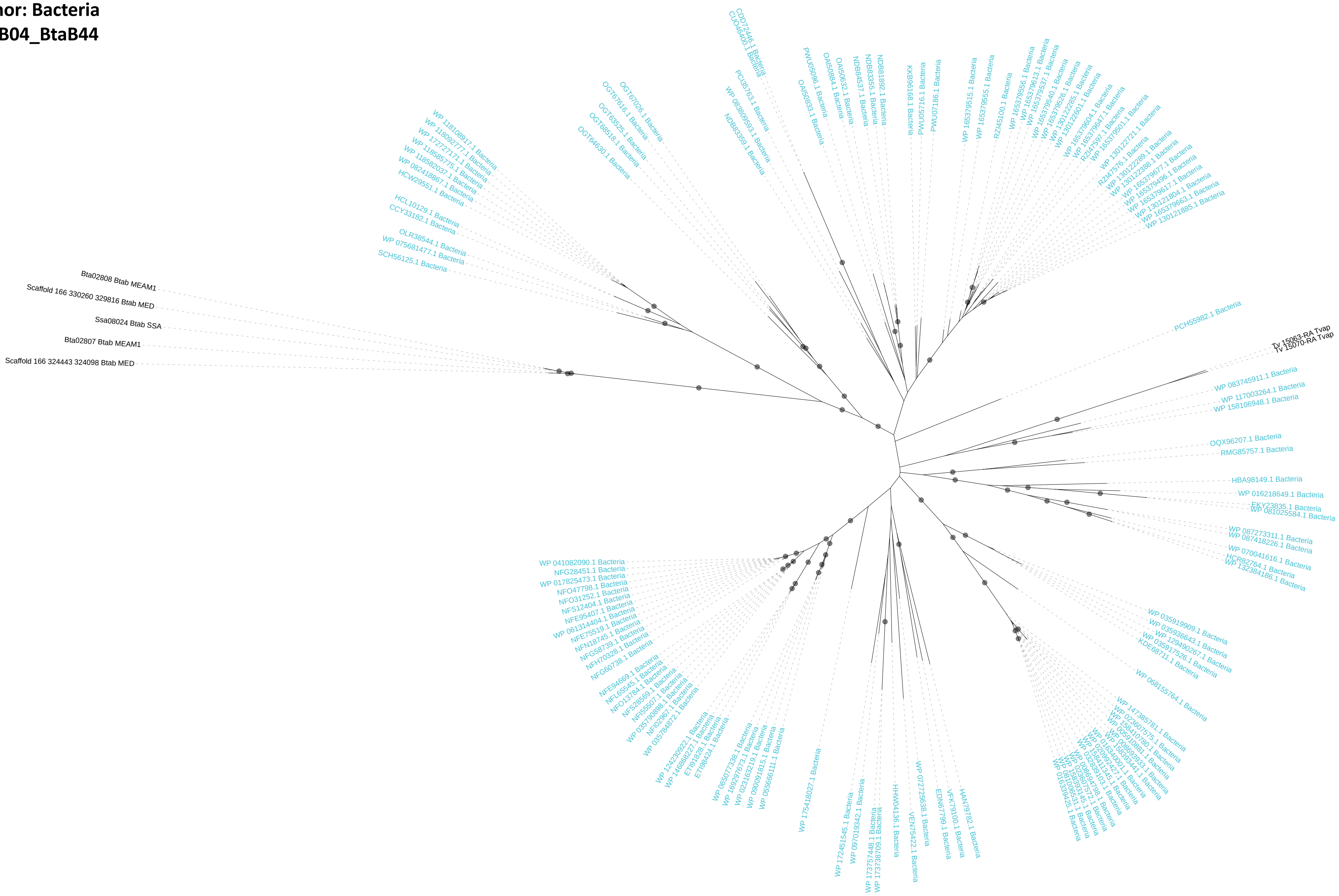

Donor: Bacteria
TvaB05_BtaB02

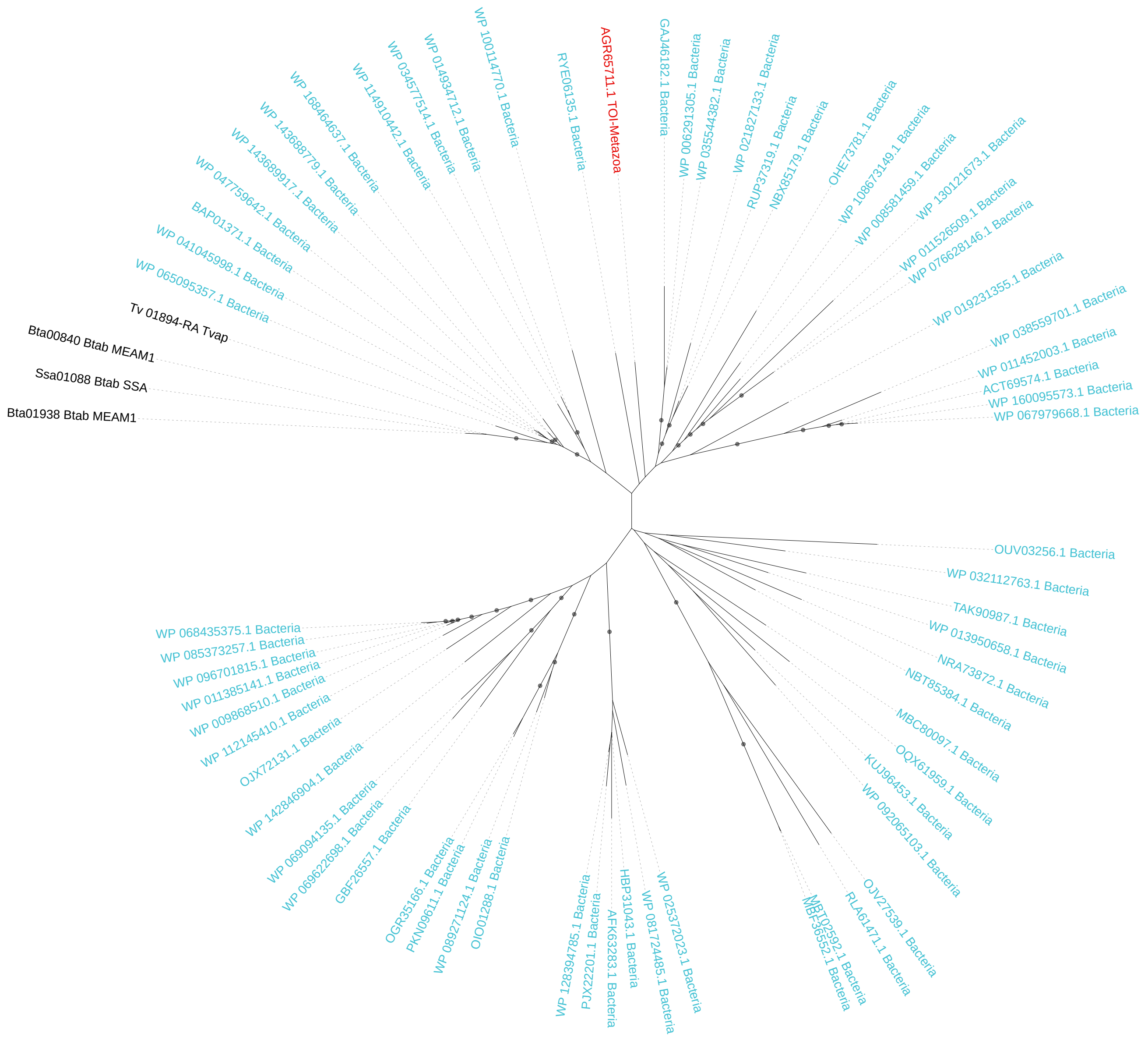

Donor: Bacteria

TvaB08_BtaB31

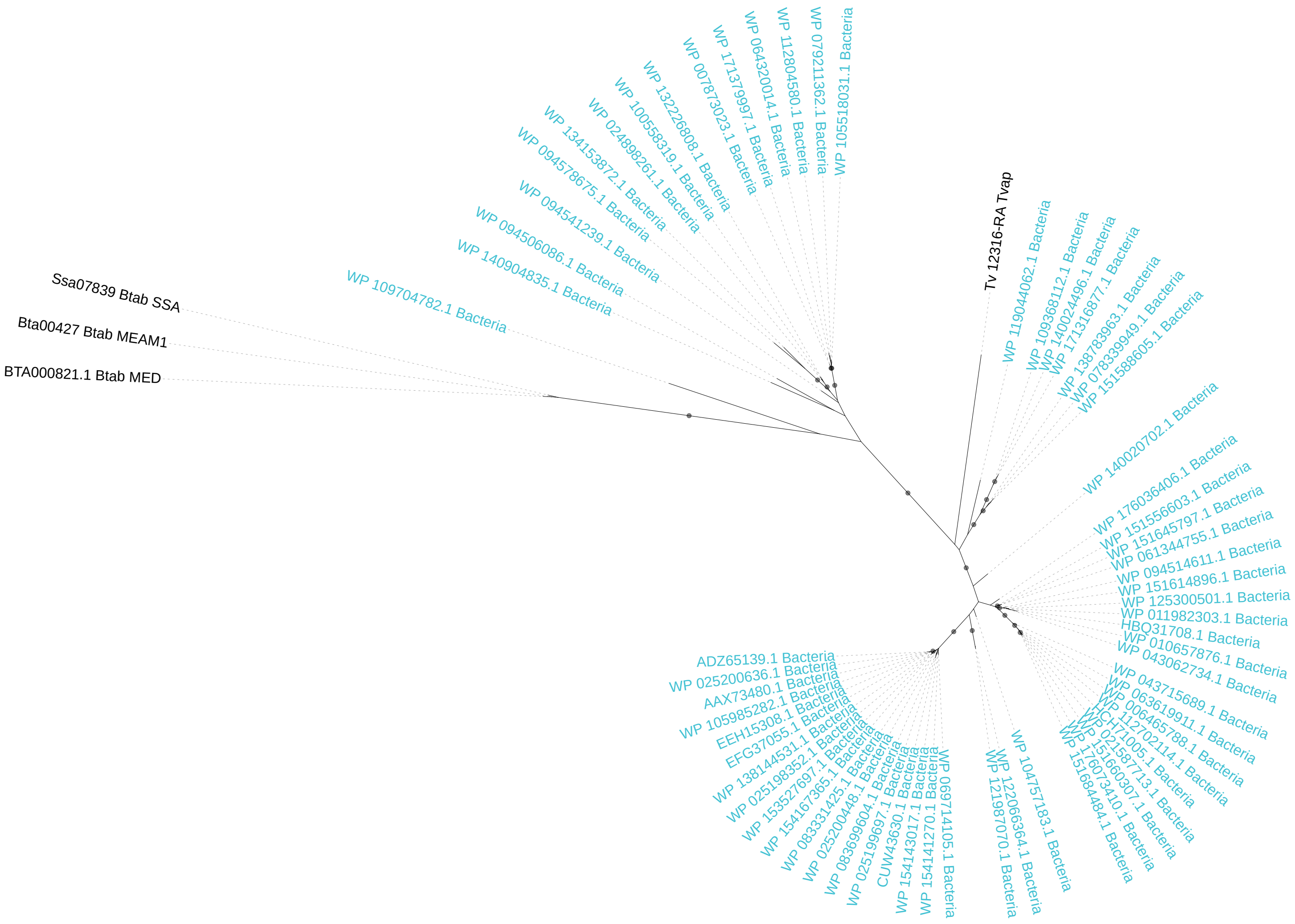

Donor: Bacteria
TvaB11_BtaB03

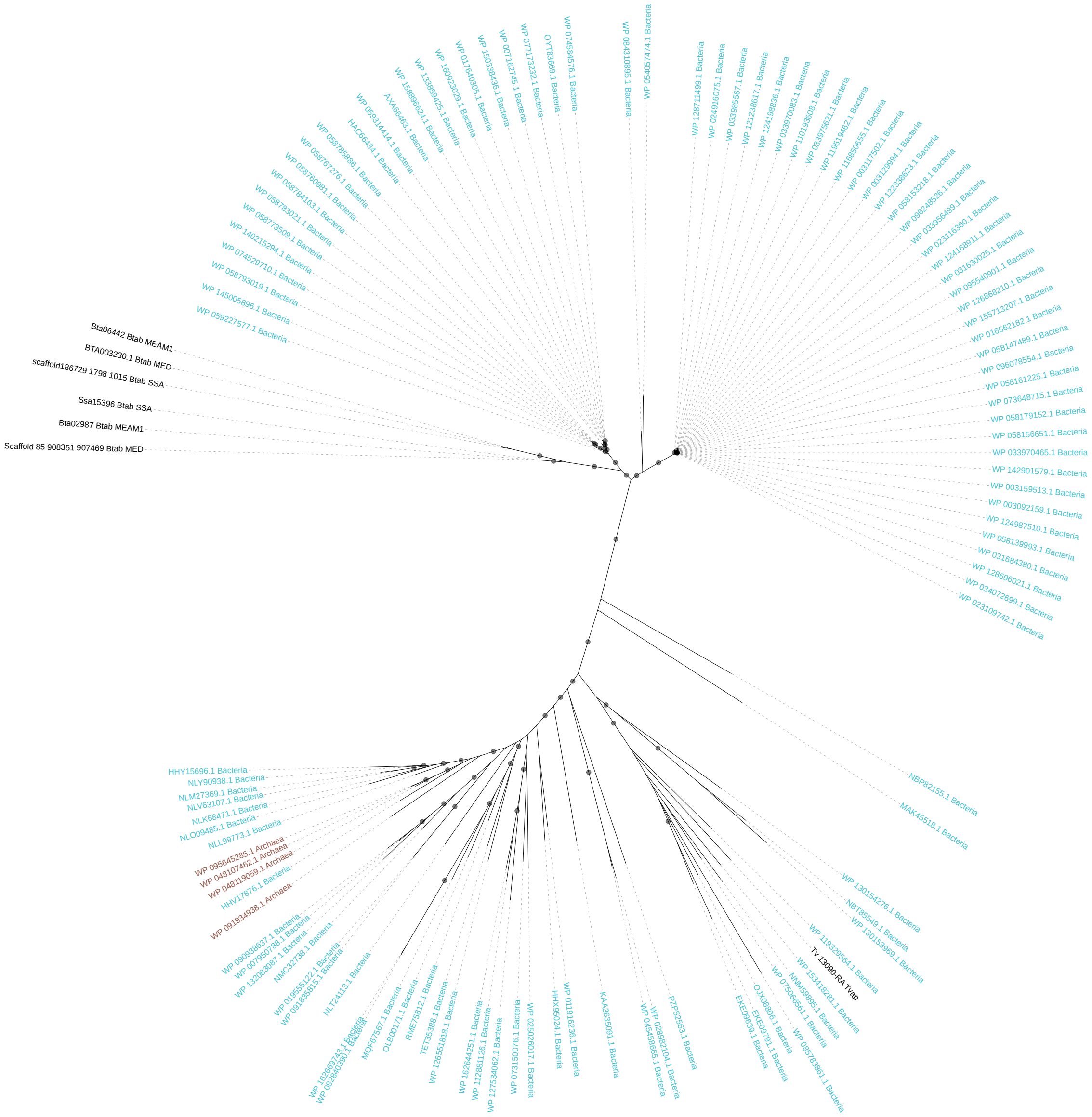

Donor: Bacteria
TvaB15_BtaB33

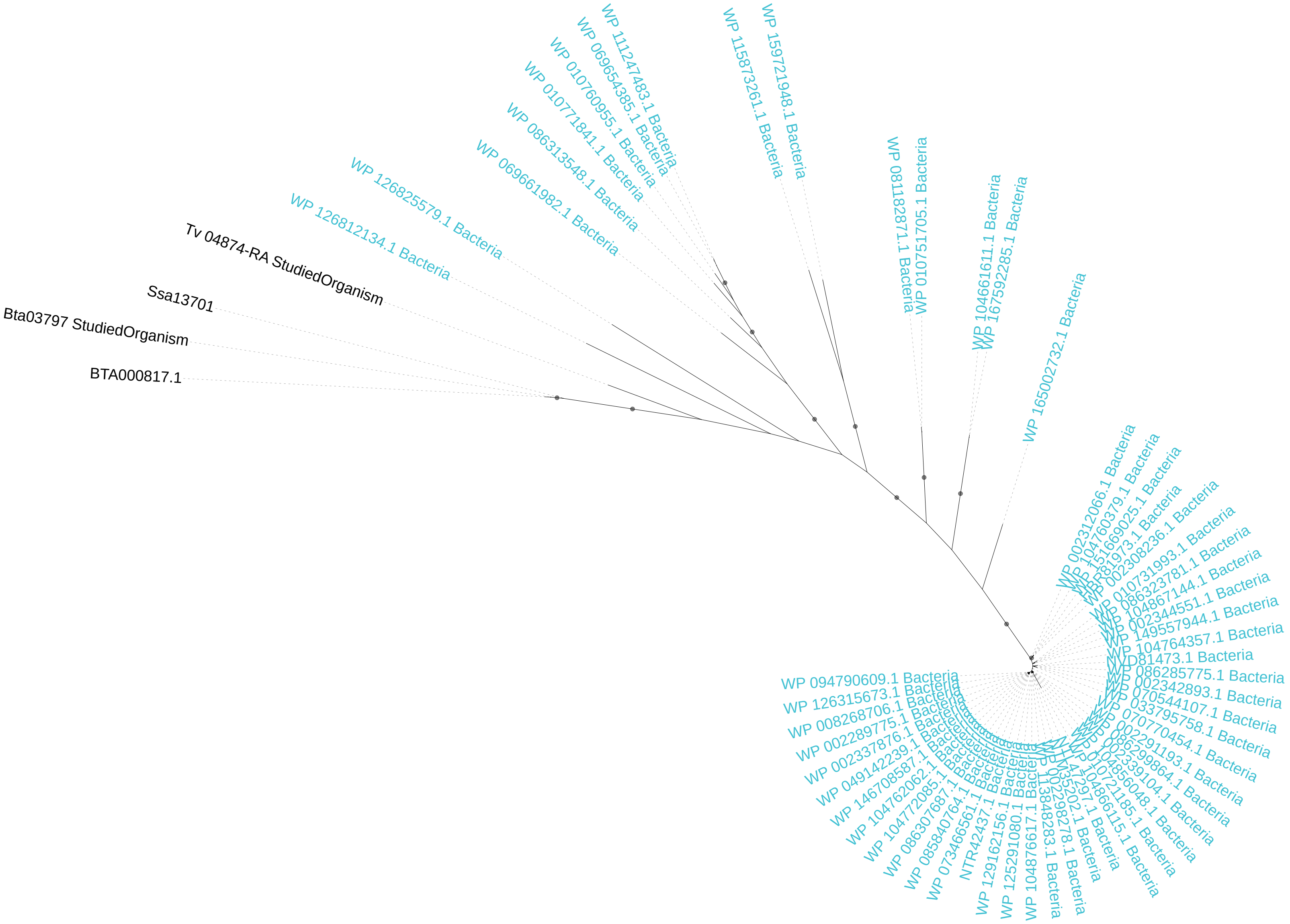

Donor: Fungi
TvaF04_BtaF10

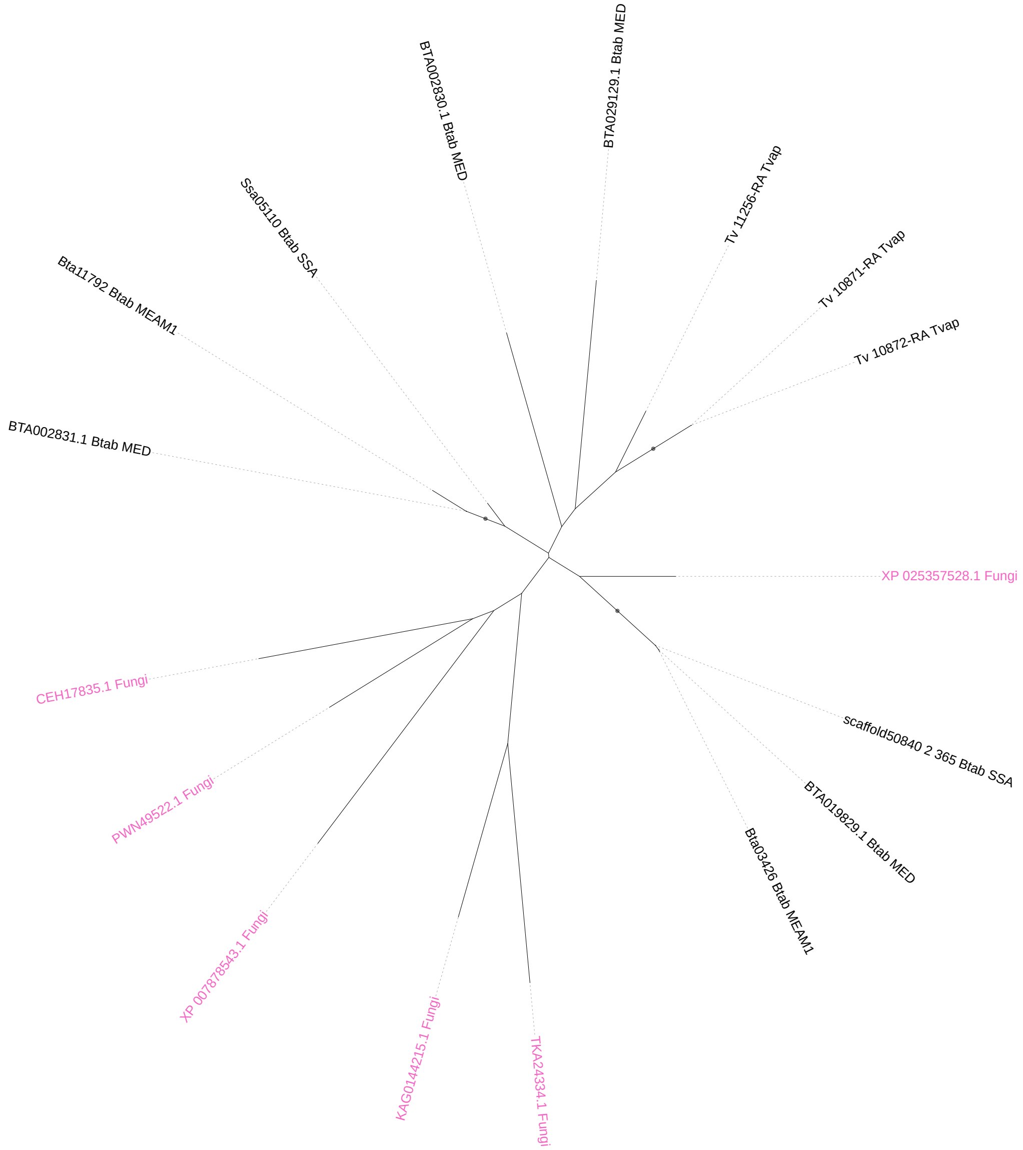

Donor: Viridiplantae
TvaV01_BtaV01

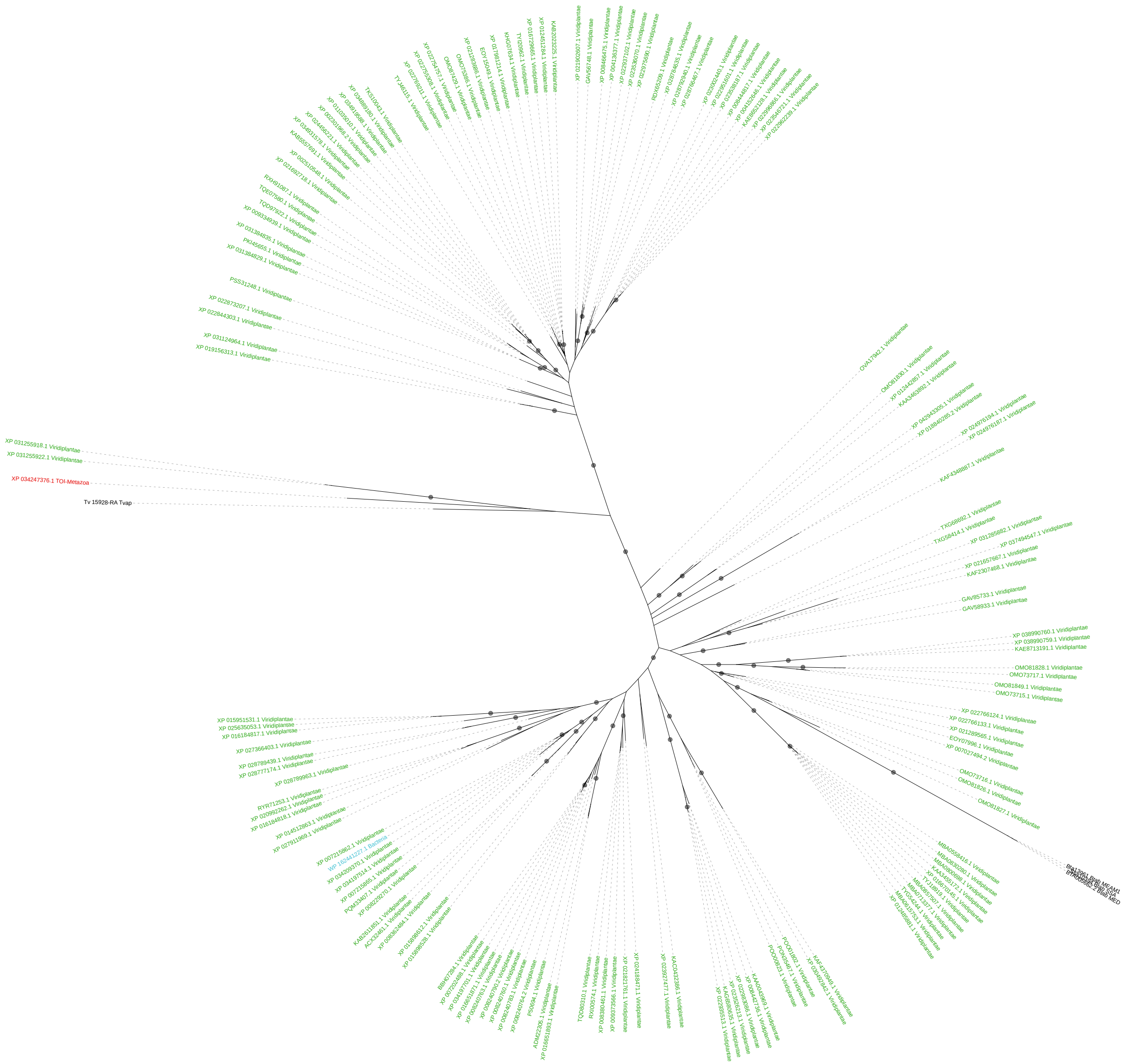

Donor: Viridiplantae

TvaV02_BtaV23

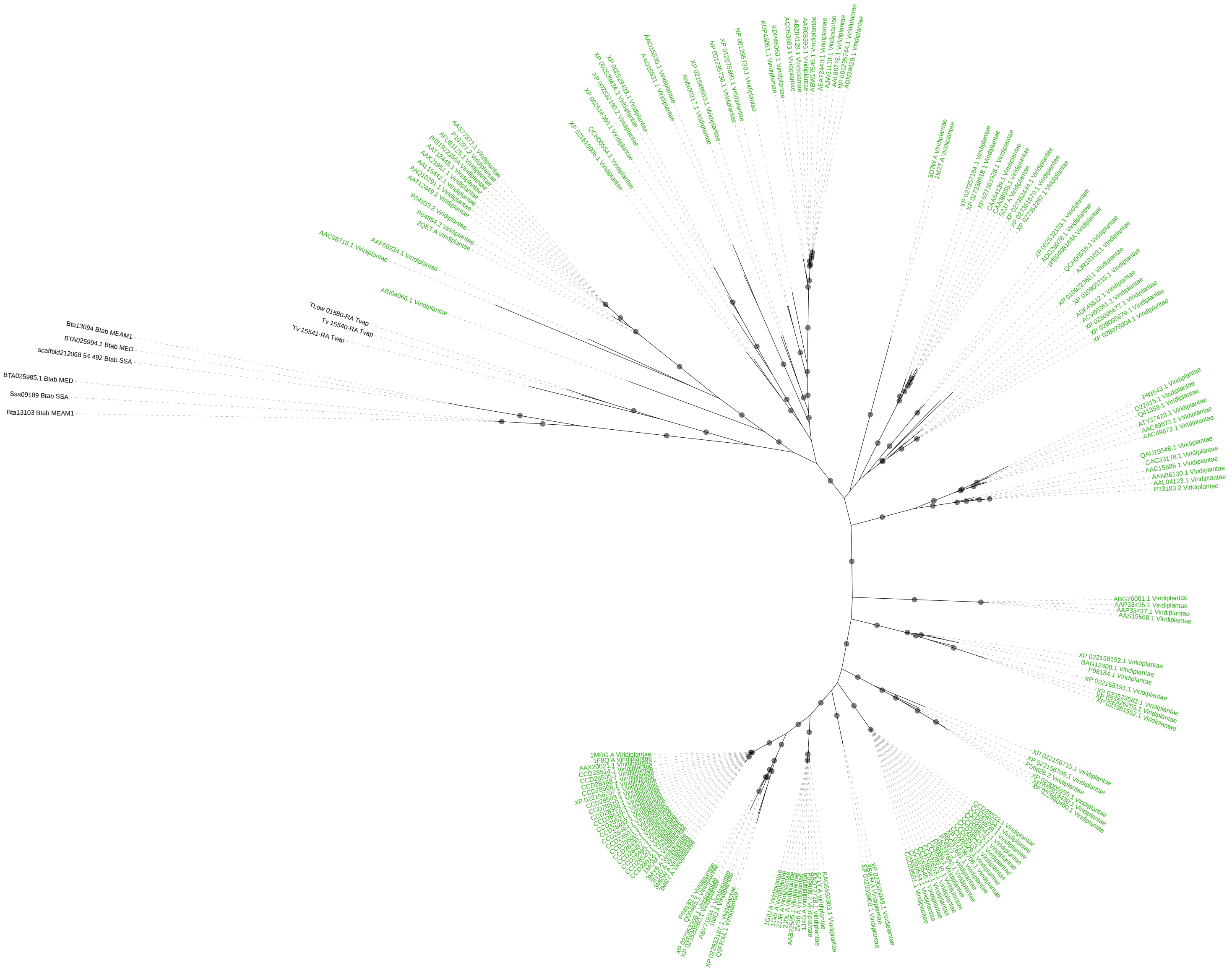

Donor: Viridiplantae
TvaV03_BtaV21

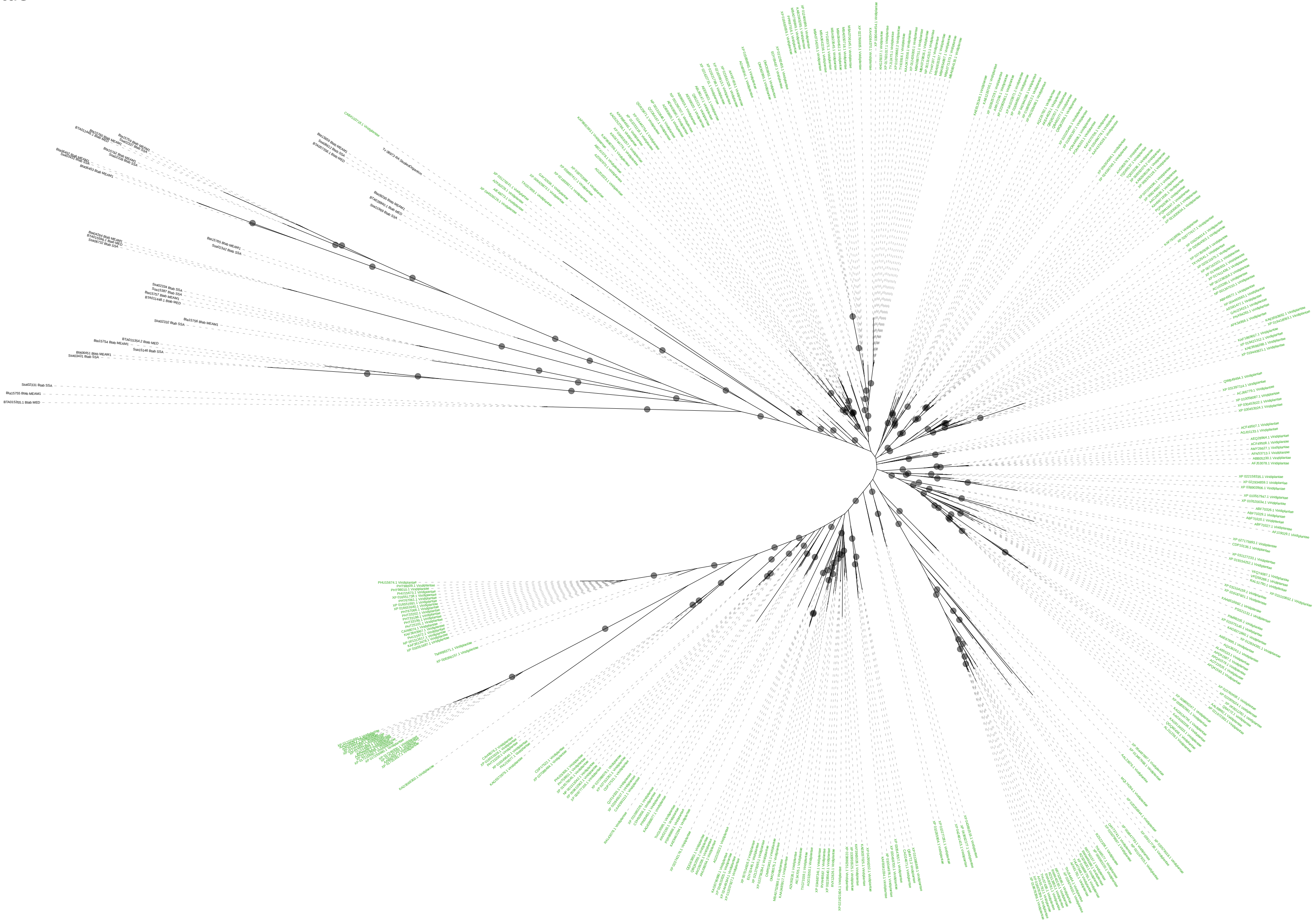

Supplementary Figure 8.

Phylogeny inferred for the HGT events corresponding to CAZymes in *F. occidentalis* and *T. palmi*. Black dots correspond to nodes with support values greater than or equal to 80% and 95% for SH-aLRT and UFboot, respectively.

FocB01_TpaB01: GH32

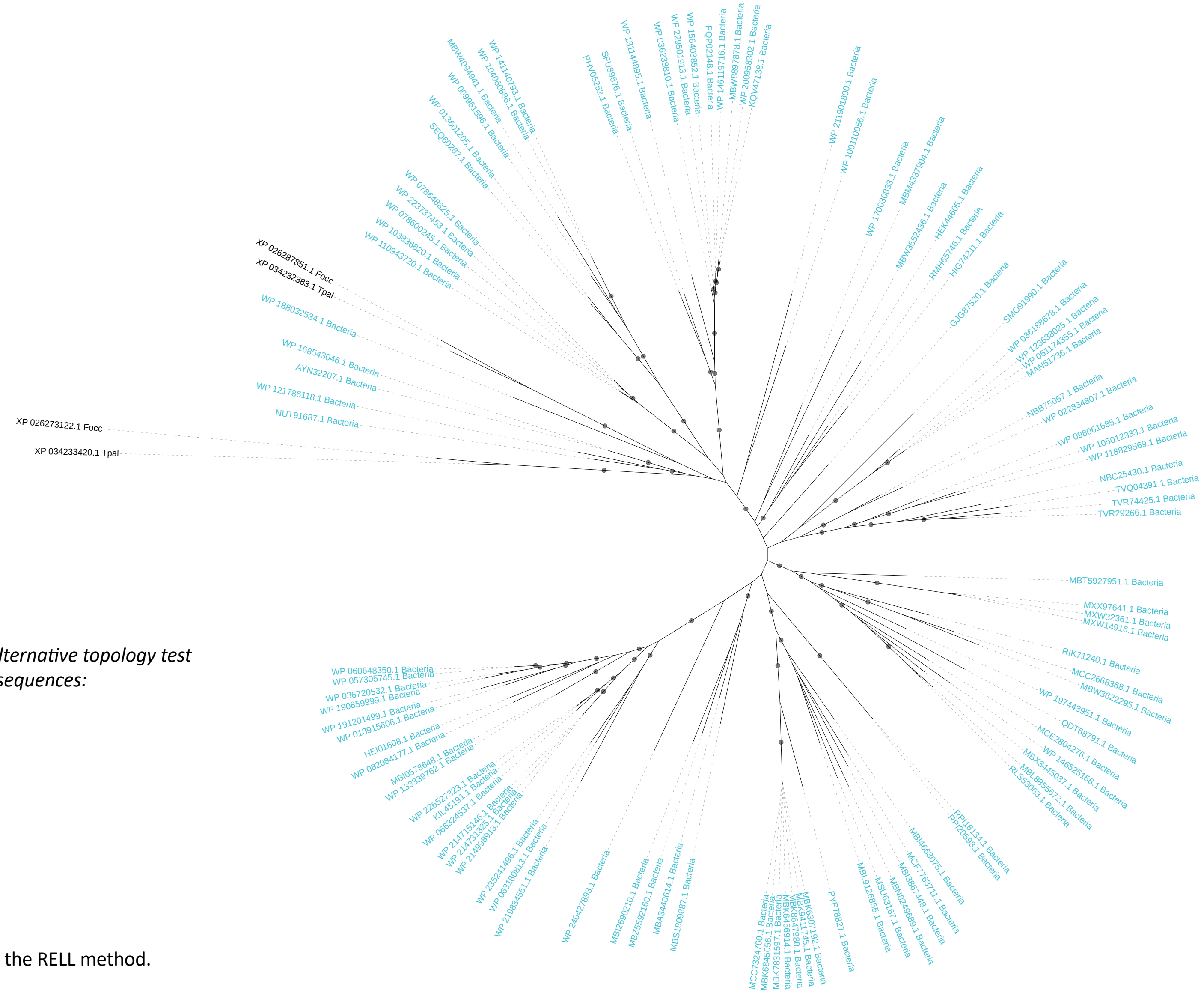

Results of the approximately unbiased (AU) alternative topology test for monophyly of F. occidentalis and T. palmi sequences:

Tree	logL	deltaL	p-AU	
1	-57479.74173	4.2003	0.427	+
2	-57475.54146	0	0.573	+

Plus signs denote the 95% confidence sets.

Minus signs denote significant exclusion.

All tests performed 10,000 resamplings using the RELL method.

FocB01_TpaB01_BtaB53: GH32

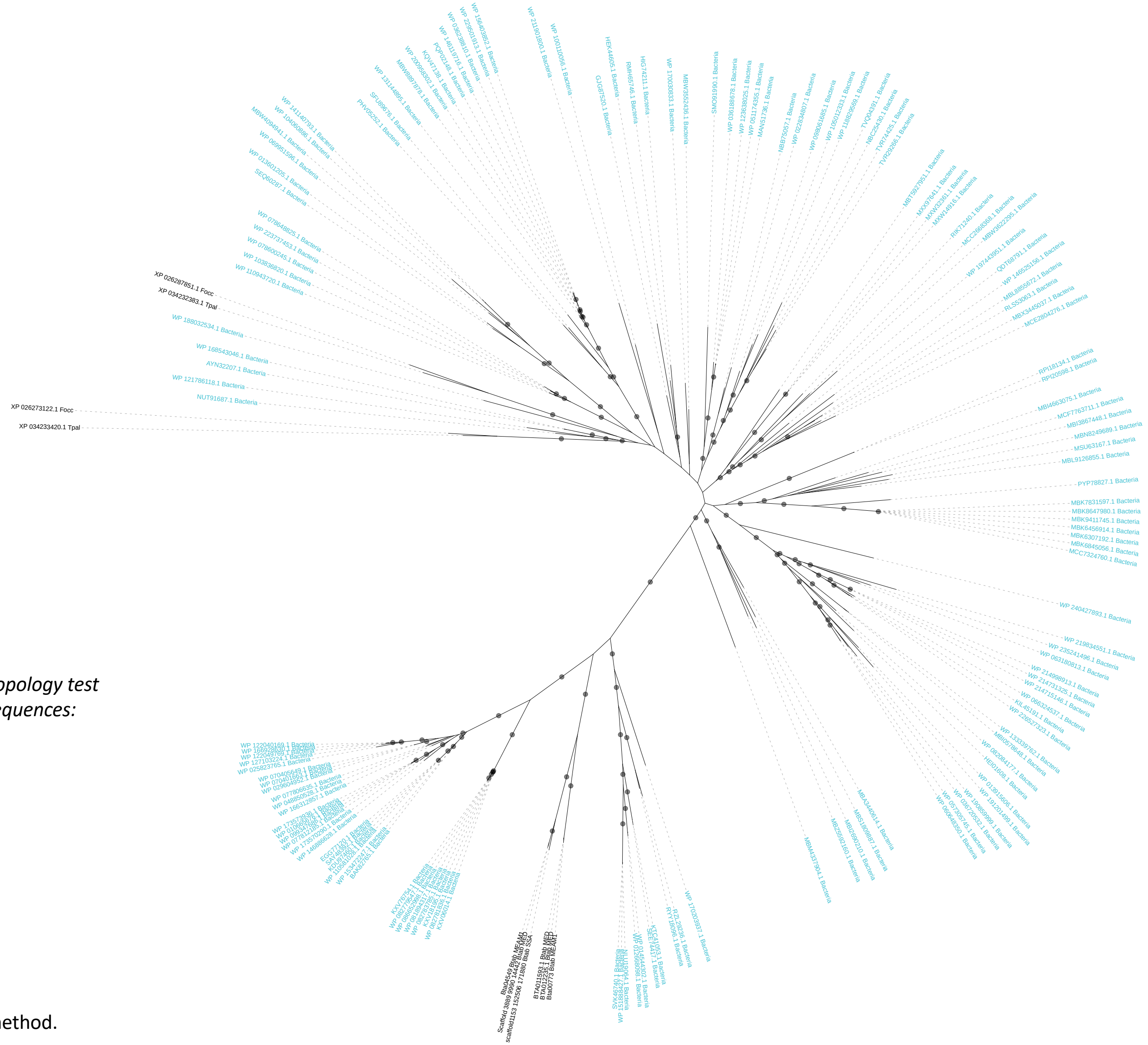

Results of the approximately unbiased (AU) alternative topology test for monophyly of F. occidentalis, T. palmi and B. tabaci sequences:

Tree	logL	deltaL	p-AU	
1	-44835.73376	178.92	2.84e-07	-
2	-44656.81805	0	1	+

Plus signs denote the 95% confidence sets.

Minus signs denote significant exclusion.

All tests performed 10,000 resamplings using the RELL method.

FocB02_TpaB02: GH5_8

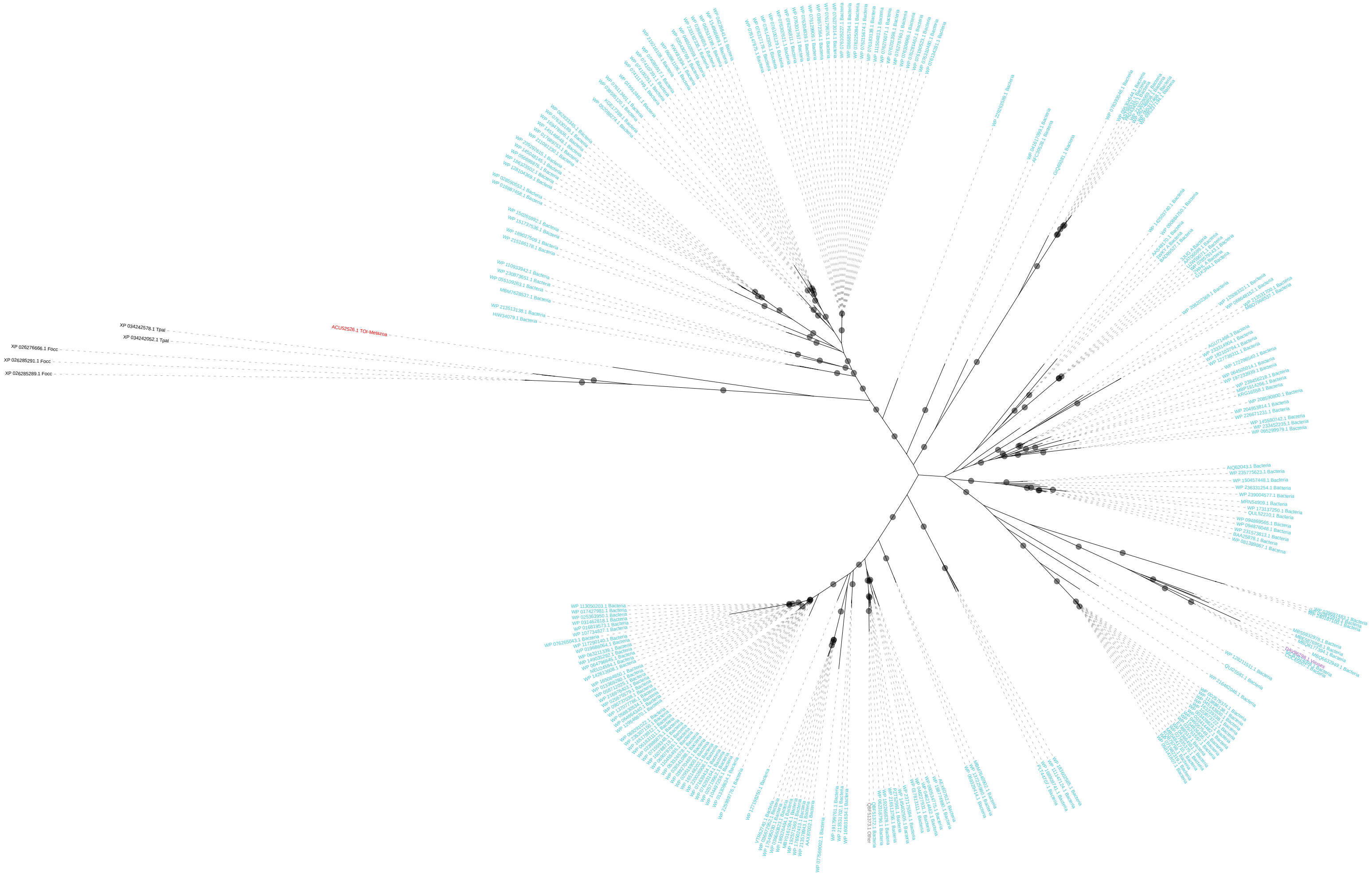

FocF01_TpaF01: GH45

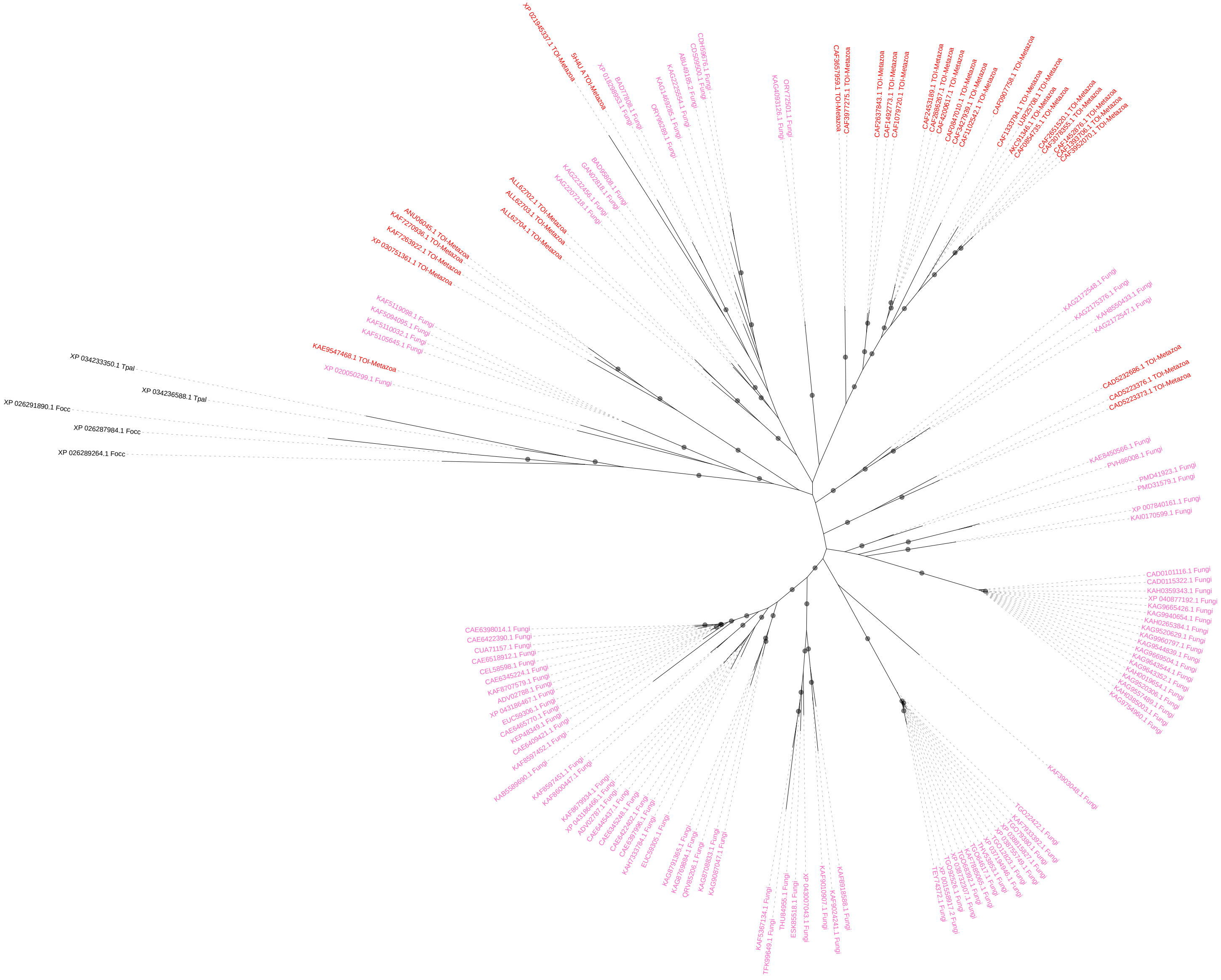

FocV01_TpaV01: GH152

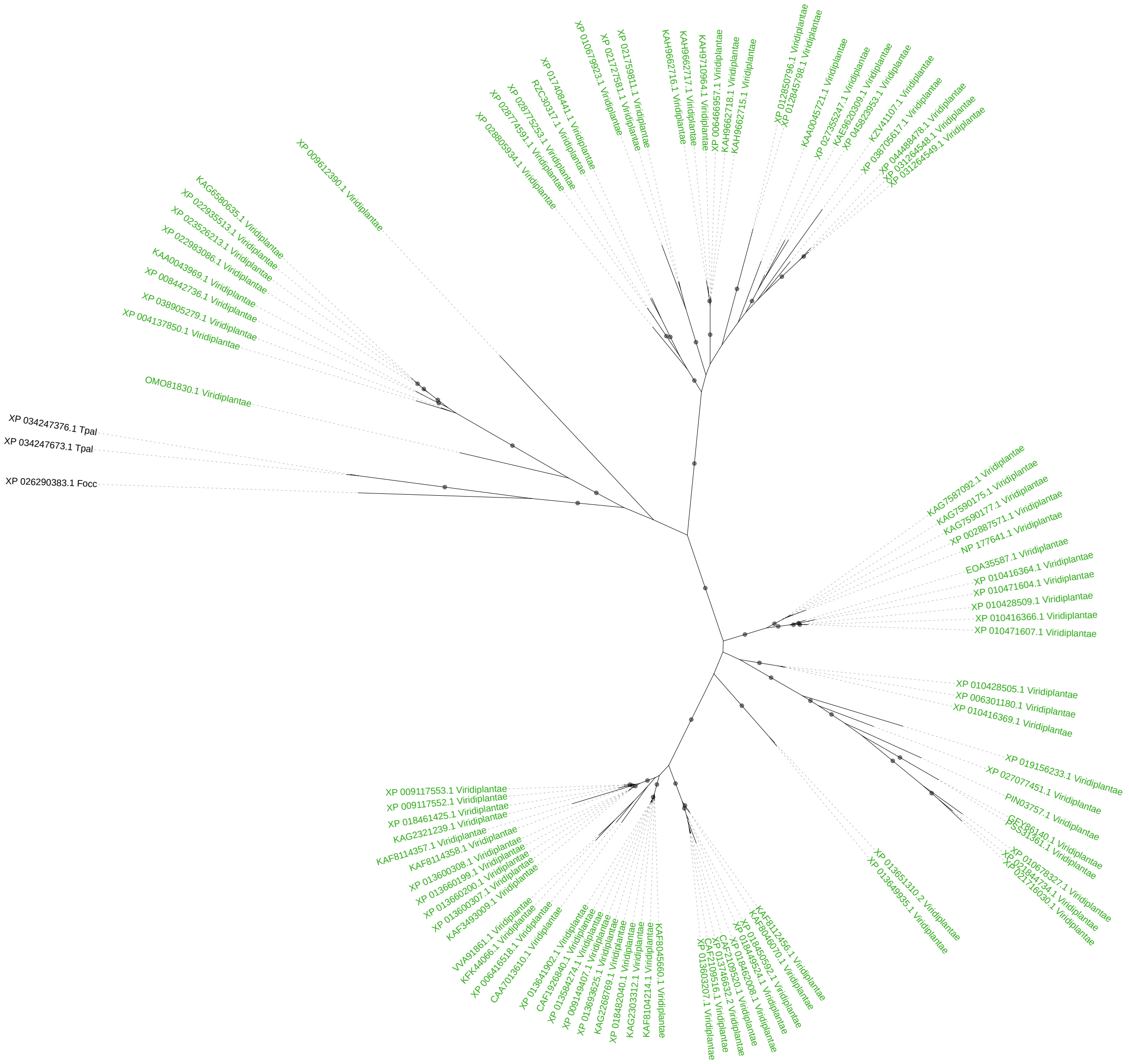

FocV01_TpaV01_BtaV01_TvaV01: GH152

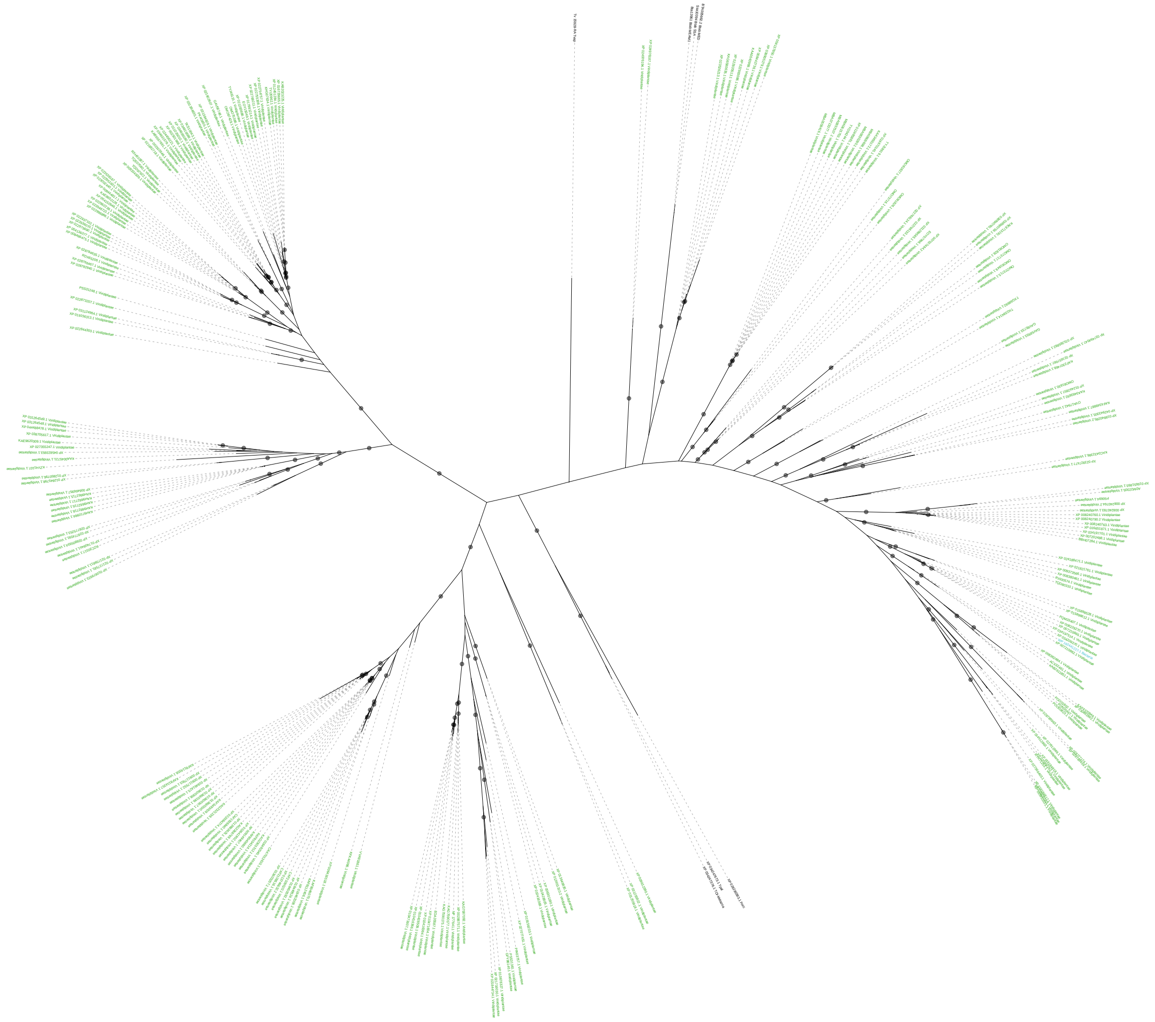

Results of the approximately unbiased (AU) alternative topology test for monophyly of Thripinae and Aleyrodinae sequences:

Tree	logL	deltaL	p-AU	
1	-22115.50057	26.954	0.206	+
2	-22088.54666	0	0.794	+

Plus signs denote the 95% confidence sets.

Minus signs denote significant exclusion.

All tests performed 10,000 resamplings using the RELL method.

FocC01_TpaC01: PL1_4

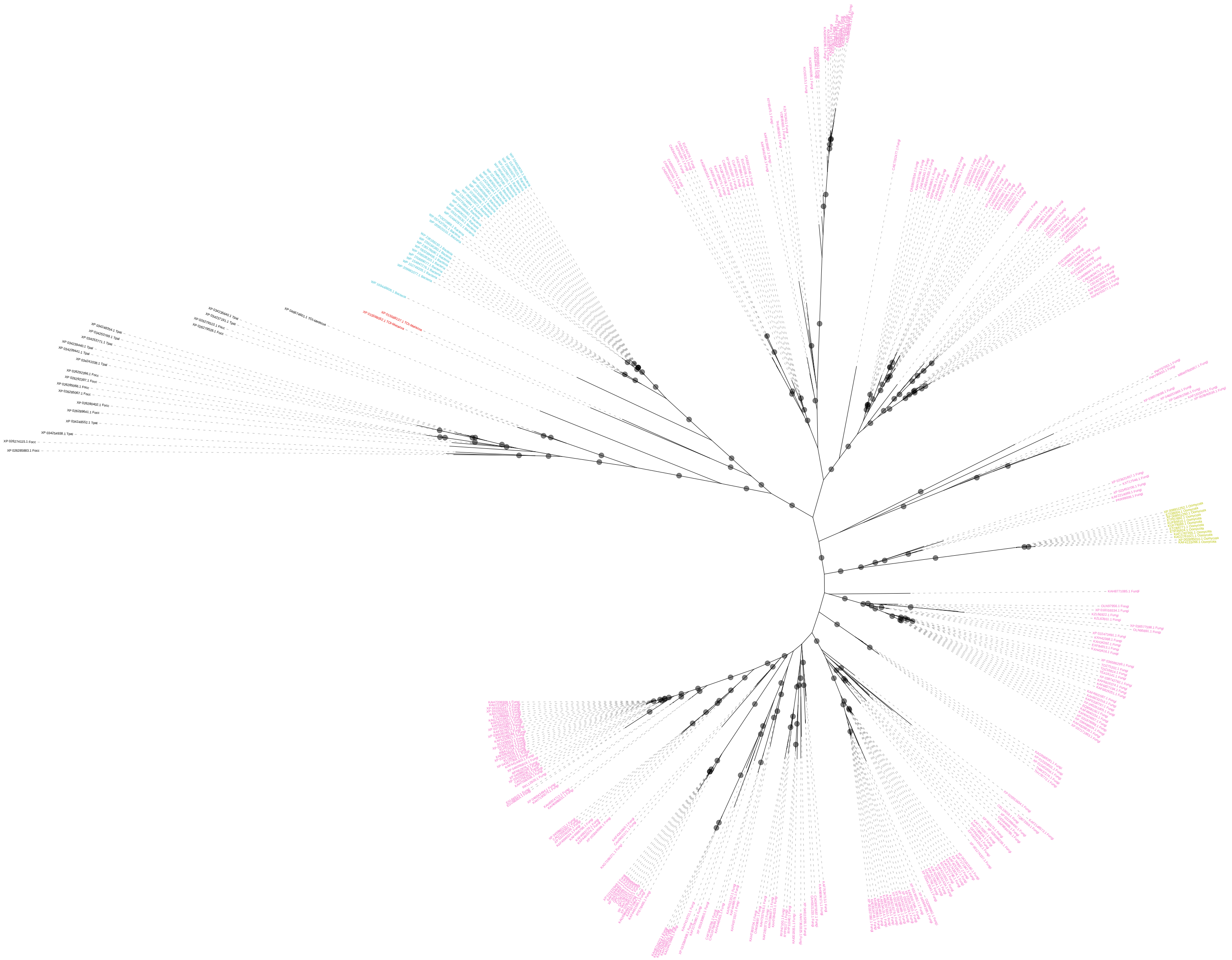

FocC01_TpaC01_BtaC01_TvaC01: PL1_4

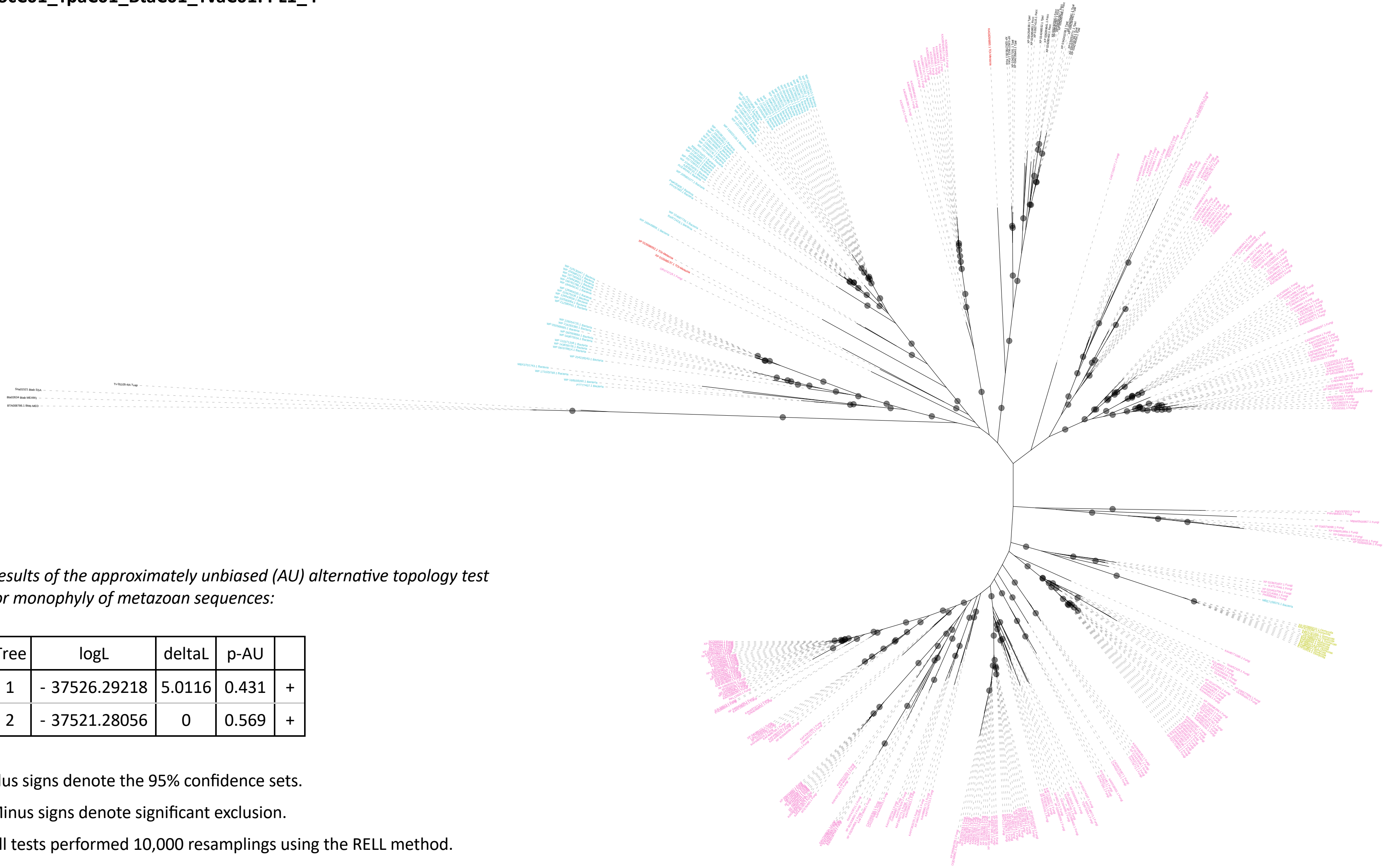

Results of the approximately unbiased (AU) alternative topology test for monophyly of metazoan sequences:

Tree	logL	deltaL	p-AU	
1	- 37526.29218	5.0116	0.431	+
2	- 37521.28056	0	0.569	+

Plus signs denote the 95% confidence sets.

Minus signs denote significant exclusion.

All tests performed 10,000 resamplings using the RELL method.

TpaV02: CBM43-CBM43-CBM43-CBM43

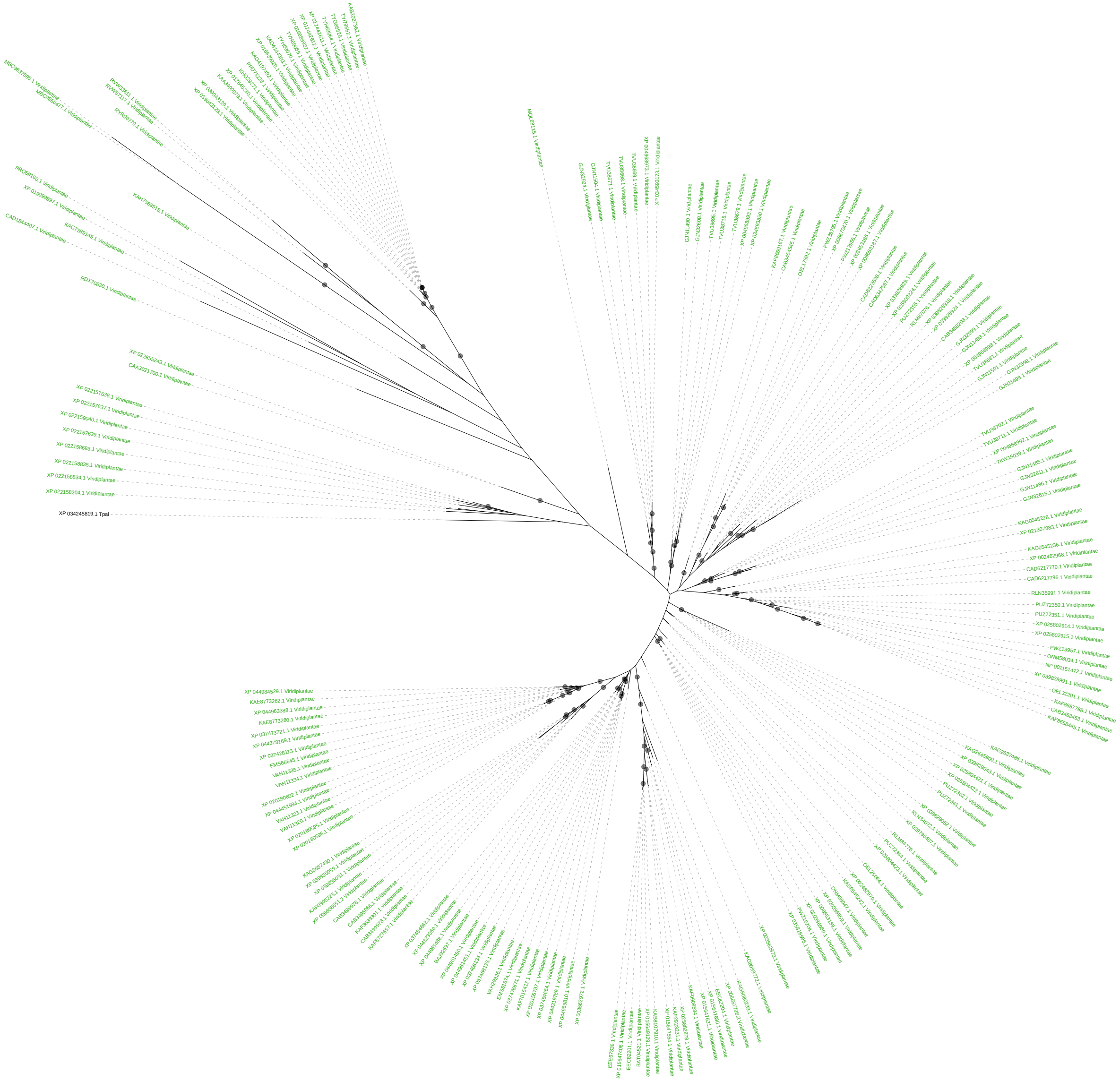

Supplement: evaf012_Supplementary_Data [file evaf012_supplementary_data.zip › Colinet 2024 GBE - Supplementary Figures.pdf]
