# Supplementary material for: Functional Carbohydrate-Active Enzymes Acquired by Horizontal Gene Transfer from Plants in the Whitefly Bemisia tabaci
Source: Genome Biol Evol. 2025 Jan 25;17(2):evaf012. doi: 10.1093/gbe/evaf012 (PMC11800479; doi:10.1093/gbe/evaf012)
Supplement: evaf012_Supplementary_Data [file evaf012_supplementary_data.zip › Colinet 2024 GBE - Supplementary Tables.pdf]

Supplementary Table 1: Estimation of proteome completeness for *Bemisia tabaci* cryptic species, *Trialeurodes vaporarorium, Frankliniella occidentalis* and *Thrips palmi* using BUSCO according to the Arthropoda Odb10 dataset

Species	Number of protein-coding genes	BUSCO Arthropoda (1013 groups searched)		
		Complete[a]	Fragmented	Missing
Bemisia tabaci MEAM1	15,662	95.6% [S:94.1%,D:1.5%]	1.5%	2.9%
Bemisia tabaci MED	20,748	86.0% [S:68.8%,D:17.2%]	3.8%	10.2%
Bemisia tabaci SSA-ECA	15,084	89.6% [S:88.0%,D:1.6%]	3.5%	6.9%
Trialeurodes vaporarorium	18,275	93.4% [S:91.6%,D:1.8%]	1.8%	4.8%
Frankliniella occidentalis	15,678	98.8%[S:96.9%,D:1.9%]	0.5%	0.7%
Thrips palmi	14,332	97.5%[S:96.3%,D:1.2%]	0.1%	2.4%

[a] S: Complete and single-copy, D: Complete and duplicated

Supplementary Table 2: Validated HGT candidates from potential bacterial, fungal or viridiplantae donors for *B. tabaci* MEAM1

Bacterial donor:

HGT event	Sequence name	Origin of donor sequences	Alternative topology Monophyly of Metazoa	Similarities with the donor sequences		Length and coverage of the alignment with the donor sequences		Local score for genomic environment	Homologs found in the two other *B. tabaci* cryptic species		Homologs found in *T. vaporarorium*	Annotation (WGD version 1.2)	CAZy	Described in literature
				max id	average id	average aln length	average coverage		*B. tabaci* MED	*B. tabaci* SSA-ECA				
BtaB01	Bta02653	Bacteria	/	66.1	64.7	375	98.5	0.67	BTA005171.1, BTA005173.1, BTA012513.1, BTA019053.1, BTA023694.1, BTA023695.1, BTA029954.2	Ssa00993, Ssa02587, Ssa02588, Ssa02589, Ssa09979, Ssa12718	Tv_08602-RA, Tv_10007-RA	Cyclopropane-fatty-acyl-phospholipid synthase family		Chen et al. 2016
	Bta04469			64.9	63.7	373	97.8	0.9						Chen et al. 2016 ; Li et al. 2022
	Bta05264			68.5	66.4	273	99.6	/						No
	Bta06818			58.1	56.4	395	98.2	0.59						Chen et al. 2016
	Bta11911			56.3	55.2	396	98.3	0.9						Chen et al. 2016 ; Li et al. 2022
	Bta11912			59.3	57.1	394	97.5	0.9						Chen et al. 2016 ; Li et al. 2022
BtaB02	Bta00840	Bacteria	1	72.1	54.2	200	84.7	0.5		Ssa01088	Tv_01894-RA	ATP-dependent dethiobiotin synthetase BioD		Chen et al. 2016 ; Li et al. 2022
	Bta01938			70.5	52.0	86	93.4	0.6						Chen et al. 2016
BtaB03	Bta02987	Bacteria	/	68.5	65.3	265	89.1	0.96	BTA003230.1, Scaffold_85_908351_907469	Ssa15396, scaffold186729_1798_1015		Phenazine biosynthesis-like domain-containing protein		Chen et al. 2016 ; Li et al. 2022
	Bta06442			64.1	61.5	260	98.6	0.8						Chen et al. 2016 ; Li et al. 2022
BtaB04	Bta20013	Bacteria	/	46.3	40.2	145	65.2	0.87	Scaffold_897_179142_179793	Ssa06560, Ssa14300, scaffold482_832064_832712_1		hypothetical protein		Chen et al. 2016
BtaB05	Bta03847	Bacteria	/	31.5	28.7	467	71.4	0.83				AAA-ATPase-like domain-containing protein		Li et al. 2022
BtaB06	Bta00063	Bacteria	/	84.9	83.4	456	98.5	0.71	BTA005853.1	scaffold95668_2976_6064		Argininosuccinate lyase		Chen et al. 2016
BtaB07	Bta20014	Bacteria	/	61.3	59.7	703	92.3	0.8	BTA005969.2	Ssa09927		methyltransferase		Chen et al. 2016
BtaB08	Bta09186	Bacteria	/	66.7	62.6	143	92.6	0.8	BTA019104.1	Ssa07340		Ribonuclease H		Chen et al. 2016
BtaB09	Bta12386	Bacteria	/	40.1	32.3	433	86.0	0.79	BTA006502.1	Ssa03543, Ssa03544	Tv_05628-RA	AAA-ATPase-like domain-containing protein		Li et al. 2022
BtaB10	Bta01157	Bacteria	/	58.1	55.3	237	96.0	0.21	BTA010421.1, BTA016630.1, scaffold153941_658_1924	Ssa01004		Uncharacterized protein		No
	Bta01158			57.7	54.9	237	67.7	0.28						Chen et al. 2016 ; Li et al. 2022
	Bta04479			59.4	56.8	240	94.8	0.76						Chen et al. 2016 ; Li et al. 2022
	Bta05970			58.5	55.4	239	96.9	/						No
BtaB11	Bta09725	Bacteria	1	70.6	66.2	309	93.8	0.94	BTA004219.1, BTA028913.1	Ssa10171	Tv_15468-RA	Biotin synthase		Chen et al. 2016 ; Li et al. 2022
BtaB12	Bta00841	Bacteria	1	70.5	55.3	273	93.8	0.7	BTA023651.1	Ssa01553	Tv_01895-RA	Adenosylmethionine-8-amino-7-oxononanoate aminotransferase BioA		Chen et al. 2016 ; Li et al. 2022
	Bta01937			73.5	59.7	426	96.7	0.63						Chen et al. 2016 ; Li et al. 2022
BtaB13	Bta04607	Bacteria	/	30.6	27.3	451	67.5	0.61	BTA010686.1	Ssa04937		AAA-ATPase-like domain-containing protein		Li et al. 2022
BtaB14	Bta14802	Bacteria	/	68.4	63.7	398	84.1	0.92	BTA007711.2	Ssa08712		Amidohydrolase		Chen et al. 2016 ; Li et al. 2022
BtaB15	Bta02416	Bacteria	/	46.7	41.2	66	53.0	0.83		Ssa01016		Unknown protein		No
BtaB16	Bta13770	Bacteria	/	32.6	29.6	101	41.6	0.91	BTA022448.2	Ssa14696		Unknown protein		No
BtaB17	Bta02167	Bacteria	/	61.5	52.0	107	10.7	0.78	BTA012693.1	Ssa02372, Ssa06822		Protein NLRC3		No
BtaB18	Bta00062	Bacteria	1	85.9	85.0	405	99.5	0.66	BTA005854.1	Ssa15432		Argininosuccinate synthase		Chen et al. 2016
BtaB19	Bta00289	Bacteria	/	30	26.7	278	27.1	0.53	BTA003732.1, BTA004712.1, BTA004713.1, BTA012459.1, BTA014511.1	Ssa06244, Ssa07626, Ssa07627, Ssa11850, Ssa13138, Ssa14803, Ssa14804, Ssa14962, Ssa15140, Ssa15337, Ssa15382		AAA-ATPase-like domain-containing protein		No
	Bta02048			31.3	29.8	471	72.6	0.9						Li et al. 2022
	Bta04448			31.7	28.3	250	73.5	0.62						No
	Bta04449			31.1	27.5	235	91.5	0.62						No
	Bta07217			30	28.7	337	98.7	/						No
	Bta10169			36.8	30.4	294	63.5	/						No
	Bta10229			30	28.7	438	67.8	0.43						No
	Bta10230			30.9	28.7	430	71.0	0.48						No
	Bta10232			31.2	29.9	418	69.6	0.55						No
	Bta10233			31.4	29.4	447	71.0	0.57						Li et al. 2022
	Bta14209			31.6	29.4	431	71.7	0.76						Li et al. 2022
	Bta15534			30.2	28.6	439	73.0	0.55						No
	Bta15536			30.3	29.2	440	66.0	0.49						No
BtaB20	Bta03200	Bacteria	/	85.9	79.6	422	96.8	0.96	Scaffold_662_290408_290801	Ssa03128		L-galactonate dehydratase		Chen et al. 2016 ; Li et al. 2022
BtaB21	Bta04921	Bacteria	/	40.7	32.6	261	47.2	0.88	BTA000806.1, BTA000807.1, BTA008325.1	Ssa01820		Unknown protein		Chen et al. 2016 ; Li et al. 2022
BtaB22	Bta03791	Bacteria	/	67.3	64.7	281	82.2	0.86	BTA021998.1			Ribosomal RNA small subunit methyltransferase A		Chen et al. 2016 ; Li et al. 2022

BtaB23	Bta03871	Bacteria	/	68.1	50.7	111	66.1	0.86	BTA027330.2	Ssa10244		Crossover junction endodeoxyribonuclease rusA		Chen et al. 2016 ; Li et al. 2022
BtaB24	Bta06657	Bacteria	/	72.0	71.0	276	86.5	/	BTA007760.1,<br>BTA013028.1	Ssa11675		Diaminopimelate epimerase		Chen et al. 2016
BtaB25	Bta15019	Bacteria	/	68.1	66.6	182	88.3	0.88	BTA012224.1	Ssa09419		Ribosome recycling factor		Chen et al. 2016 ; Li et al. 2022
BtaB26	Bta04508	Bacteria	/	73.4	72.9	1202	99.2	0.77	BTA012654.1,<br>BTA017578.1,<br>BTA025646.1	Ssa06061		Urea amidolyase		Chen et al. 2016 ; Li et al. 2022
BtaB27	Bta02625<br>Bta04622<br>Bta07024<br>Bta07125<br>Bta07126<br>Bta07870<br>Bta11369<br>Bta11370	Bacteria	/	61.1<br>56.5<br>62.1<br>58.0<br>58.5<br>65.4<br>67.4<br>67.8	58.8<br>52.8<br>59.5<br>56.2<br>57.3<br>63.9<br>65.9<br>64.8	657<br>129<br>642<br>650<br>641<br>640<br>352<br>208	98.3<br>70.6<br>96.7<br>97.5<br>89.2<br>98.0<br>97.4<br>93.1	0.85<br>0.85<br>0.91<br>0.58<br>0.68<br>0.88<br>0.66<br>0.66	BTA012629.2,<br>BTA016705.1,<br>BTA016707.1,<br>BTA030125.1,<br>BTA009444.1	Ssa01508,<br>Ssa08300,<br>Ssa10111,<br>Ssa10113,<br>Ssa10118,<br>Ssa15216		Squalene-hopene cyclase		Chen et al. 2016 ; Li et al. 2022<br>Li et al. 2022<br>Chen et al. 2016 ; Li et al. 2022<br>Chen et al. 2016 ; Li et al. 2022<br>Chen et al. 2016 ; Li et al. 2022<br>Chen et al. 2016 ; Li et al. 2022<br>Chen et al. 2016 ; Li et al. 2022<br>Chen et al. 2016
BtaB28	Bta11811	Bacteria	/	79.7	72.9	298	97.5	0.81		Ssa05133		Plant/T7N9-9 protein		Chen et al. 2016 ; Li et al. 2022
BtaB29	Bta20020	Bacteria	/	58.9	52.4	245	97.4	0.92	Scaffold_733_232<br>89_24021	scaffold570_6229<br>85_622253		4-hydroxy-tetrahydrodipicolinate reductase		Chen et al. 2016 ; Li et al. 2022
BtaB30	Bta03589<br>Bta03593	Bacteria	/	70.1<br>69.3	60.8<br>60.3	214<br>426	83.6<br>94.7	0.86<br>0.86	BTA026180.1	Ssa05826		Diaminopimelate decarboxylase		Li et al. 2022<br>Chen et al. 2016 ; Li et al. 2022
BtaB31	Bta00427	Bacteria	/	69.3	62.5	209	72.3	0.84	BTA000821.1	Ssa07839		Pantothenate kinase-like protein		Li et al. 2022
BtaB32	Bta11965	Bacteria	/	46.3	38.9	130	73.4	0.81	BTA017096.1	Ssa00217		Unknown protein		No
BtaB33	Bta03797	Bacteria	/	62.5	58.2	103	34.4	0.9	BTA000817.1	Ssa13701	Tv_04874-RA	Methylated-DNA--[protein]-cysteine S-methyltransferase		Li et al. 2022
BtaB34	Bta08776	Bacteria	/	82.9	80.6	513	95.9	1.0	BTA025302.1,<br>BTA028148.1	Ssa15043		Histidine ammonia-lyase		Chen et al. 2016
BtaB35	Bta11531<br>Bta15479	Bacteria	/	30.6<br>33	28.6<br>30.0	526<br>439	79.1<br>90.5	0.9<br>0.92	BTA004921.1,<br>BTA018889.1,<br>BTA026605.1	Ssa12442	Tv_02105-RA	AAA-ATPase-like domain-containing protein		No<br>Li et al. 2022
BtaB36	Bta00829	Bacteria	/	33.3	30.7	526	71.4	0.45	BTA007316.1,<br>BTA011402.1	scaffold554257_3<br>12_1956	Tv_00146-RA,<br>Tv_00148-RA,<br>Tv_01481-RA,<br>Tv_02102-RA,<br>Tv_02106-RA,<br>Tv_15366-RA	AAA-ATPase-like domain-containing protein		No
BtaB37	Bta15184<br>Bta15191<br>Bta20012	Bacteria	/	68.1<br>70.8<br>78.1	66.3<br>69.3<br>75.6	255<br>256<br>255	98.9<br>99.2<br>99.0	0.61<br>0.75<br>0.57	BTA021540.1,<br>BTA021541.1,<br>BTA030079.2,<br>BTA030081.1,<br>BTA030084.1			Oxidoreductase, 2OG-Fe(II) oxygenase family protein		Chen et al. 2016 ; Li et al. 2022<br>Chen et al. 2016 ; Li et al. 2022<br>Chen et al. 2016 ; Li et al. 2022
BtaB38	Bta20015	Bacteria	/	56.3	47.1	443	88.0	0.9	BTA012131.1			hypothetical protein		Chen et al. 2016 ; Li et al. 2022
BtaB39	Bta06820	Bacteria	/	61.6	60.7	336	85.8	0.63	BTA019051.2	Ssa09977		Tryptophan-tRNA ligase		Chen et al. 2016
BtaB40	Bta02826	Bacteria	/	35.2	27.6	445	70.7	0.86	BTA005203.1	Ssa08291		AAA-ATPase-like domain-containing protein		No
BtaB41	Bta04431	Bacteria	/	75.4	70.1	183	91.2	1.0	BTA007219.2,<br>BTA020661.1	Ssa00937		Acetyltransferase		Chen et al. 2016 ; Li et al. 2022
BtaB42	Bta05813	Bacteria	/	54.9	47.1	318	45.9	0.71	BTA005145.1	Ssa12681		D-alanine--D-alanine ligase		No
BtaB43	Bta02812	Bacteria	/	70.8	69.0	264	99.2	0.68	BTA006638.1,<br>BTA006641.1	scaffold413_2442<br>29_245024		4,5 dioxygenase extradiol		Chen et al. 2016 ; Li et al. 2022
BtaB44	Bta02807<br>Bta02808	Bacteria	/	42.9<br>43.1	37.4<br>39.0	115<br>111	82.4<br>72.6	0.55<br>0.6	Scaffold_166_324<br>443_324098,<br>Scaffold_166_330<br>260_329816	Ssa08024		AAA-ATPase-like domain-containing protein		No<br>Li et al. 2022
BtaB45	Bta15103	Bacteria	/	50.6	44.6	151	71.6	0.88	BTA020874.1	Ssa07364	Tv_06835-RA	Chorismate mutase 1		Chen et al. 2016 ; Li et al. 2022
BtaB46	Bta07556<br>Bta07557	Bacteria	/<br>/	57.1<br>58.3	51.4<br>52.6	111<br>106	46.6<br>44.0	0.78<br>0.78	BTA024248.1	Ssa13644,<br>Ssa13645,<br>Ssa13646		Uncharacterized protein		No<br>Li et al. 2022
BtaB47	Bta04809	Bacteria	/	30.8	29.2	412	69.2	0.56	BTA006704.1	Ssa06266		AAA-ATPase-like domain-containing protein		Li et al. 2022
BtaB48	Bta04987<br>Bta04988	Bacteria	/	72.3<br>71.7	69.0<br>70.0	277<br>285	88.7<br>95.4	0.82<br>0.86	BTA020296.1,<br>BTA021770.1,<br>BTA021771.2	Ssa00739,<br>Ssa00740		Amidinotransferase		Chen et al. 2016 ; Li et al. 2022<br>Chen et al. 2016 ; Li et al. 2022
BtaB49	Bta13948	Bacteria	/	62.6	60.9	177	69.0	0.76	Scaffold_155_308<br>817_300779	scaffold3967_193<br>336_184613		DUF1768-domain-containing protein		No
BtaB50	Bta15764	Bacteria	/	65.0	60.3	121	93.8	0.16		Ssa02340		Uncharacterized protein		No
BtaB51	Bta05339	Bacteria	1	72.1	66.6	285	45.3	0.91	BTA017168.3	Ssa09543		3-methyl-2-oxobutanoate hydroxymethyltransferase		Chen et al. 2016 ; Li et al. 2022

BtaB52	Bta12603	Bacteria	/	31.6	28.1	475	75.5	0.96	BTA027018.1	scaffold190238_1244_716		AAA-ATPase-like domain-containing protein		Li et al. 2022
BtaB53	Bta00773<br>Bta04549	Bacteria	/	49.1<br>46.8	45.5<br>44.1	476<br>487	80.3<br>88.1	0.44<br>0.88	BTA011593.1,<br>BTA012235.1,<br>Scaffold_3889_9990_14442	scaffold1153_152506_171880		Beta-fructofuranosidase	GH32	Li et al. 2022<br>Li et al. 2022
BtaB54	Bta07115	Bacteria	/	76.4	71.8	258	72.3	0.52		scaffold333079_2023_1150		Meiotic chromosome segregation protein		Li et al. 2022
BtaB55	Bta09846	Bacteria	/	31.3	29.0	569	87.8	0.81	BTA001371.1	Ssa14624		AAA-ATPase-like domain-containing protein		Li et al. 2022
BtaB56	Bta10852<br>Bta10853	Bacteria	/	36.8<br>26.1	29.5<br>23.5	476<br>207	80.6<br>74.5	0.59<br>0.64	BTA009628.1,<br>BTA009629.1,<br>BTA025251.1,<br>BTA025252.1,<br>BTA025253.1	Ssa11036	TLow_01347-RA,<br>TLow_02144-RA,<br>TLow_02157-RA,<br>Tv_13818-RA,<br>Tv_13862-RA,<br>Tv_15776-RA	AAA-ATPase-like domain-containing protein		Li et al. 2022<br>No
BtaB57	Bta00871	Bacteria	/	53.7	50.0	226	79.2	0.95	BTA021726.1	Ssa14229		Ribonuclease 3		Chen et al. 2016
BtaB58	Bta13949	Bacteria	/	72.2	70.4	597	97.3	0.76	BTA005651.1	Ssa03692		Glutamyl-tRNA(Gln) amidotransferase subunit A, putative		Chen et al. 2016
BtaB59	Bta15712	Bacteria	/	61.7	56.8	125	50.1	0.27	BTA015916.1	Ssa02297		SCP-like extracellular		Chen et al. 2016
BtaB60	Bta13118	Bacteria	/	79.5	76.2	219	97.8	0.77	BTA014321.1			GNAT family acetyltransferase		Chen et al. 2016 ; Li et al. 2022
BtaB61	Bta07442	Bacteria	/	73.4	65.5	190	98.0	0.73	BTA023294.1	scaffold763_1615070_1615649		YJL217W-like protein		Li et al. 2022
BtaB62	Bta15714	Bacteria	/	60.0	56.1	193	87.2	0.36	BTA015913.1			SCP-like extracellular	CBM50	Chen et al. 2016 ; Li et al. 2022
BtaB63	Bta00042<br>Bta02417	Bacteria	/	62.6<br>38.0	57.7<br>27.5	405<br>202	96.2<br>94.1	0.29<br>0.83	BTA016358.1,<br>Scaffold_5042_3912_2826	Ssa10934,<br>scaffold26337_34592_35222		Ser/Thr protein phosphatase family		Li et al. 2022<br>Chen et al. 2016
BtaB64	Bta05687	Bacteria	/	50.0	44.9	145	55.8	0.48	Scaffold_2144_73225_73981	Ssa04068		Deoxyuridine 5'-triphosphate nucleotidohydrolase		No
BtaB65	Bta05013<br>Bta10549<br>Bta14973	Bacteria	/	32.1<br>32<br>32.3	30.3<br>28.9<br>29.9	511<br>546<br>516	79.9<br>73.4<br>78.9	0.67<br>0.85<br>0.82	BTA002393.1	Ssa01599,<br>Ssa10865,<br>Ssa10873,<br>Ssa12918,<br>Ssa13905,<br>Ssa14120,<br>Ssa15393	Tv_00487-RA,<br>Tv_11451-RA	AAA-ATPase-like domain-containing protein		No<br>Li et al. 2022<br>No
BtaB66	Bta15713	Bacteria	/	63.3	50.5	176	39.0	0.32	BTA015915.1	Ssa02298		SCP-like extracellular		Chen et al. 2016 ; Li et al. 2022
BtaB67	Bta04447	Bacteria	/	40.1	33.2	397	86.0	0.62	BTA008945.6	Ssa00953		AAA-ATPase-like domain-containing protein		Li et al. 2022
BtaB68	Bta00747<br>Bta13975	Bacteria	/	49.6<br>52.0	44.7<br>45.3	146<br>144	94.6<br>95.7	/<br>0.91		Ssa08703,<br>Ssa14910	Tv_02831-RA,<br>Tv_11854-RA,<br>Tv_12101-RA,<br>Tv_15086-RA,<br>Tv_15720-RA,<br>Tv_15881-RA	Deoxyuridine 5'-triphosphate nucleotidohydrolase		No<br>No
BtaB69	Bta00002	Bacteria	/	53.7	52.5	713	99.1	0.78	BTA004354.1,<br>BTA008424.1	Ssa09173		ATP-dependent DNA helicase Q-like 3		Chen et al. 2016 ; Li et al. 2022
BtaB70	Bta05382	Bacteria	0	46.8	43.8	137	20.4	0.76	BTA000114.1	Ssa05150		Extracellular dioxygenase		Li et al. 2022

Fungal donor:

HGT event	Sequence name	Origin of donor sequences	Alternative topology	Similarities with the donor sequences		Length and coverage of the alignment with the donor sequences		Local score for genomic environment	Homologs found in the two other *B. tabaci* cryptic species		Homologs found in *T. vaporarorium*	Annotation (WGD version 1.2)	CAZy	Described in literature
				max id	average id	average aln length	average coverage		*B. tabaci* MED	*B. tabaci* SSA-ECA				
BtaF01	Bta00659	Fungi	/	54.1	49.3	377	86.7	0.55	BTA010566.1,<br>BTA010567.1,<br>BTA010568.1	Ssa03926		Aromatic peroxygenase		Chen et al. 2016 ; Li et al. 2022
	Bta00660			49.6	45.6	404	91.5	0.49						Chen et al. 2016 ; Li et al. 2022
BtaF02	Bta12907	Fungi	/	46.9	43.0	343	68.8	0.82	BTA009603.1,<br>BTA017757.1,<br>BTA009604.1,<br>BTA017756.1	Ssa10521,<br>Ssa10522,<br>Ssa10524		Late sexual development protein		Chen et al. 2016 ; Li et al. 2022
	Bta12908			47.8	43.0	348	92.4	0.82						Chen et al. 2016 ; Li et al. 2022
BtaF03	Bta02178	Fungi	/	43.5	40.3	438	91.9	0.92	BTA007752.1	Ssa06839		Aminotransferase family protein (LolT), putative		Chen et al. 2016 ; Li et al. 2022
BtaF04	Bta10926	Fungi	/	30.8	28.8	106	38.8	0.92	BTA000754.1,<br>BTA011323.1,<br>BTA016968.1,<br>BTA023763.1,<br>BTA025826.1,<br>BTA026082.1	Ssa02288		Replication factor-a protein 1		No
	Bta13511			32.4	29.3	106	45.3	0.91						No
BtaF05	Bta04178	Fungi	/	46.1	27.9	911	84.7	1.0	BTA015808.1,<br>BTA015810.1,<br>BTA026116.1,<br>BTA026115.2	Ssa02954,<br>Ssa02955,<br>Ssa04524,<br>Ssa04525,<br>Ssa04526		2OG-Fe(II) oxygenase superfamily protein		Chen et al. 2016 ; Li et al. 2022
	Bta14373			43.9	28.4	914	60.8	0.5						Chen et al. 2016 ; Li et al. 2022
	Bta14374			48.8	36.9	515	73.2	0.54						Chen et al. 2016 ; Li et al. 2022
BtaF06	Bta02658	Fungi	/	66.5	60.6	461	95.0	0.72		Ssa02594		Glucosylceramidase, putative	GH30	Chen et al. 2016 ; Li et al. 2022
BtaF07	Bta02250	Fungi	1	63.8	51.6	580	92.6	0.77	BTA018568.2	Ssa14166		Dextranase	GH49	Chen et al. 2016 ; Li et al. 2022
BtaF08	Bta14185	Fungi	/	42.3	39.9	677	98.8	0.92		Ssa14298		Uracil phosphoribosyltransferase		Chen et al. 2016 ; Li et al. 2022
BtaF09	Bta13117	Fungi	1	59.0	48.3	402	94.5	0.74	BTA014319.1	scaffold461923_670_139		Serine/threonine dehydratase serine racemase		Chen et al. 2016
BtaF10	Bta01809	Fungi	/	45.1	41.2	400	92.7	0.96	BTA021351.1,<br>BTA016059.1,<br>BTA024446.1,<br>BTA028865.1,<br>BTA020831.1,<br>BTA021213.1,<br>BTA029208.1,<br>BTA029214.1,<br>BTA029213.1,<br>BTA029215.1,<br>BTA029216.1,<br>BTA001574.1,<br>BTA001575.1,<br>BTA022986.1,<br>BTA022987.1,<br>BTA022989.1,<br>BTA022990.1,<br>BTA022991.1	Ssa10324,<br>Ssa01851,<br>Ssa09478,<br>Ssa09479,<br>Ssa09482,<br>Ssa06288,<br>Ssa00368,<br>Ssa12703,<br>Ssa10743,<br>Ssa10745,<br>Ssa00340,<br>Ssa00341,<br>Ssa09259,<br>Ssa09258,<br>Ssa09257,<br>Ssa10800		Major royal jelly-related protein		Chen et al. 2016
	Bta01821			47.0	45.1	405	77.3	0.82						Chen et al. 2016
	Bta04681			50.6	48.0	365	70.5	0.65						Chen et al. 2016 ; Li et al. 2022
	Bta05463			40.4	38.2	403	86.2	0.8						Chen et al. 2016 ; Li et al. 2022
	Bta05465			41.9	40.7	405	87.9	0.7						Li et al. 2022
	Bta05467			37.1	35.6	397	82.2	0.7						Li et al. 2022
	Bta06167			44.3	41.1	248	46.9	0.95						Chen et al. 2016
	Bta07455			43.8	41.4	411	95.1	0.84						Chen et al. 2016 ; Li et al. 2022
	Bta10729			44.5	40.7	382	52.4	0.5						Chen et al. 2016
	Bta10733			38.6	36.9	405	87.5	0.47						Chen et al. 2016 ; Li et al. 2022
	Bta10734			39.9	35.3	213	84.5	0.44						No
	Bta10735			39.9	34.8	157	92.3	0.41						No
	Bta10736			38.7	37.5	402	88.5	0.37						Chen et al. 2016
	Bta10737			40.0	38.6	402	80.8	0.32						Chen et al. 2016 ; Li et al. 2022
	Bta10760			53.1	50.2	398	54.7	0.66						Chen et al. 2016 ; Li et al. 2022
	Bta10761			47.0	43.6	416	91.2	0.66						Chen et al. 2016 ; Li et al. 2022
	Bta14699			41.1	37.9	416	96.0	0.76						Chen et al. 2016
	Bta14700			41.9	38.9	406	93.6	0.76						Chen et al. 2016
	Bta14701			48.6	46.2	410	83.7	0.76						Chen et al. 2016
	Bta14896			48.8	45.3	408	94.6	0.92						Chen et al. 2016
BtaF11	Bta11792	Fungi	/	47.9	38.5	140	50.5	0.92	BTA002831.1,<br>BTA002830.1,<br>BTA019829.1,<br>BTA029129.1	Ssa05110,<br>scaffold50840_2_365	Tv_10871-RA,<br>Tv_10872-RA,<br>Tv_11256-RA	Unknown protein		No
	Bta03426			54.3	40.3	189	74.9	0.82						No
BtaF12	Bta03315	Fungi	/	65.9	56.2	342	96.1	0.88	Scaffold_934_82522_83563	Ssa11275		Adenosine deaminase		Chen et al. 2016
BtaF13	Bta12326	Fungi	/	35.3	31.4	257	89.2	0.91	BTA009468.1	scaffold5114_691867_692650		Activating signal cointegrator 1 complex subunit-like protein		Chen et al. 2016
	Bta20017			33.7	29.6	413	88.4	0.96						Chen et al. 2016
BtaF14	Bta11043	Fungi	/	62.3	59.7	400	92.4	1.0	BTA013802.1,<br>BTA015522.1	Ssa00130,<br>Ssa00131		Squalene synthase		Chen et al. 2016
BtaF15	Bta03557	Fungi	/	72.9	67.6	608	99.4	0.74	BTA001108.1,<br>BTA022973.1,<br>BTA025701.1,<br>BTA027649.1	Ssa05748,<br>Ssa13062		Gamma-glutamyltranspeptidase		Chen et al. 2016
	Bta05545			67.4	63.2	609	99.6	0.84						Chen et al. 2016
BtaF16	Bta02866	Fungi	/	52.7	42.3	426	94.8	1.0	BTA013774.1	scaffold8_3079009_3077710		Alpha-1,3-glucanase, putative	GH71	Chen et al. 2016 ; Li et al. 2022
BtaF17	Bta14111	Fungi	0	41.0	36.5	216	93.2	1.0	BTA014094.1	Ssa09898		Transcription factor Rba50, putative		Chen et al. 2016 ; Li et al. 2022

BtaF18	Bta20021	Fungi	/	60.1	56.0	590	98.5	0.76	BTA012080.1	scaffold894_3378<br>74_339566		cryptochrome		Chen et al. 2016
BtaF19	Bta01072<br>Bta03567<br>Bta05090<br>Bta05091<br>Bta05092<br>Bta05093<br>Bta07691<br>Bta09066<br>Bta12453	Fungi	1	58.2<br>46.2<br>53.3<br>55.1<br>56.2<br>55.3<br>46.2<br>55.8<br>51.3	56.0<br>44.5<br>52.3<br>53.5<br>50.6<br>53.8<br>45.6<br>54.8<br>47.2	655<br>651<br>644<br>636<br>375<br>637<br>644<br>650<br>127	95.4<br>93.9<br>87.9<br>89.7<br>99.0<br>89.8<br>92.3<br>93.8<br>74.8	0.71<br>0.78<br>0.33<br>0.38<br>0.43<br>0.42<br>0.92<br>1.0<br>/	BTA004602.1,<br>BTA010570.1,<br>BTA009422.2,<br>BTA010036.3,<br>BTA001712.1	Ssa03924,<br>Ssa14575,<br>Ssa02112,<br>Ssa02113,<br>Ssa07603,<br>Ssa07877		Galactose oxidase	AA5+CBM3<br>2	Chen et al. 2016 ; Li et al. 2022<br>Chen et al. 2016 ; Li et al. 2022<br>Li et al. 2022<br>Chen et al. 2016 ; Li et al. 2022<br>Chen et al. 2016<br>Chen et al. 2016 ; Li et al. 2022<br>Chen et al. 2016 ; Li et al. 2022<br>Chen et al. 2016 ; Li et al. 2022<br>No
BtaF20	Bta15600<br>Bta15601	Fungi	/	45.7<br>46.9	43.0<br>43.1	175<br>233	90.6<br>76.3	/<br>/	BTA008707.1	Ssa12367		Aromatic peroxygenase		Chen et al. 2016<br>Chen et al. 2016 ; Li et al. 2022
BtaF21	Bta01073<br>Bta07721	Fungi	/	55.9<br>54.5	52.9<br>51.0	358<br>389	54.4<br>93.2	0.66<br>0.77	BTA004600.1,<br>BTA004601.1,<br>BTA010569.1,<br>BTA017481.1	Ssa03925,<br>Ssa07646		Aromatic peroxygenase		Chen et al. 2016 ; Li et al. 2022<br>Chen et al. 2016 ; Li et al. 2022
BtaF22	Bta00932<br>Bta05475<br>Bta07433	Fungi	/	33.0<br>32.1<br>32.1	30.7<br>30.4<br>30.3	1247<br>1257<br>1254	99.3<br>99.4<br>98.4	0.93<br>0.76<br>0.45	BTA005597.1,<br>BTA005598.1,<br>BTA005599.1,<br>BTA007606.1,<br>BTA003000.1,<br>BTA004644.1,<br>BTA008032.1,<br>BTA008563.1	sa06477,<br>Ssa06478,<br>Ssa06479,<br>Ssa04914,<br>Ssa15384	Tv_08361-RA,<br>Tv_14110-RA,<br>Tv_14111-RA	MYND finger family protein		Chen et al. 2016<br>Chen et al. 2016 ; Li et al. 2022<br>Chen et al. 2016 ; Li et al. 2022
BtaF23	Bta05635	Fungi	/	64.7	60.7	243	84.3	0.91	Scaffold_31_8000<br>23_801601	scaffold47637_45<br>_1696		Duf1275 domain protein		Chen et al. 2016 ; Li et al. 2022
BtaF24	Bta10569	Fungi	/	59.0	49.3	611	93.5	0.84	BTA015701.1	scaffold299226_1<br>298_59		Tyrosyl-DNA phosphodiesterase<br>domain protein		Chen et al. 2016 ; Li et al. 2022
BtaF25	Bta20016	Fungi	/	78.4	70.5	164	90.9	0.9	BTA012132.1,<br>BTA021974.1	scaffold532168_5<br>9820_60357		cyanate hydratase		Chen et al. 2016 ; Li et al. 2022
BtaF26	Bta08944<br>Bta08945<br>Bta08946<br>Bta14076	Fungi	/	49.0<br>46.8<br>53.0<br>55.5	43.6<br>41.9<br>48.9<br>49.5	1181<br>1244<br>2102<br>1606	94.4<br>88.5<br>99.7<br>99.8	0.68<br>0.71<br>0.73<br>/	BTA016279.2,<br>BTA025951.2,<br>BTA025953.1,<br>BTA025954.1	Ssa11553,<br>Ssa12591,<br>Ssa15247	Tv_07966-RA	NFX1-type zinc finger-containing<br>protein 1		Chen et al. 2016<br>Chen et al. 2016<br>Chen et al. 2016<br>No
BtaF27	Bta10703	Fungi	/	41.4	35.5	351	82.7	0.88	BTA013842.1	Ssa02002		Zinc finger MYND-type protein		Chen et al. 2016
BtaF28	Bta00658	Fungi	/	53.6	50.0	387	87.9	0.6	BTA010565.1	Ssa03927		Aromatic peroxygenase		Chen et al. 2016 ; Li et al. 2022
BtaF29	Bta00041	Fungi	/	54.8	52.1	388	87.5	0.34	BTA016362.1	Ssa10936		Aromatic peroxygenase		Chen et al. 2016 ; Li et al. 2022
BtaF30	Bta08225	Fungi		44.9	41.1	270	64.6	0.9	BTA009130.1	Ssa05079		Aromatic peroxygenase		Chen et al. 2016
BtaF31	Bta08226	Fungi	/	56.0	46.7	111	25.8	0.9	Scaffold_520_329<br>177_399713	scaffold1911_304<br>567_303956		Aromatic peroxygenase		Chen et al. 2016
BtaF32	Bta05277<br>Bta20018<br>Bta20019	Fungi	/	48.4<br>43.6<br>54.5	34.0<br>33.8<br>47.4	329<br>338<br>218	86.9<br>86.5<br>78.0	0.56<br>0.8<br>0.8	BTA017099.3	Ssa13377,<br>Ssa13378		Virginiamycin B lyase protein		No<br>Chen et al. 2016<br>Chen et al. 2016
BtaF33	Bta01735	Fungi	/	64.4	60.9	479	90.9	0.87	BTA009203.1	Ssa04872		Glutamyl-tRNA(Gln)<br>amidotransferase subunit A		Li et al. 2022
BtaF34	Bta11373	Fungi	/	45.9	43.7	377	76.8	0.76	Scaffold_424_428<br>395_446441	Ssa15364		Aromatic peroxygenase		Chen et al. 2016
BtaF35	Bta02217<br>Bta05754	Fungi		66.4<br>57.4	59.6<br>55.1	145<br>194	83.2<br>90.6	0.66<br>0.75	BTA008151.1	scaffold1412_248<br>109_245904		Aromatic peroxygenase		Chen et al. 2016 ; Li et al. 2022<br>Chen et al. 2016
BtaF36	Bta04808	Fungi	/	43.9	41.3	369	79.1	0.51	BTA006705.1	Ssa06267		Aromatic peroxygenase		Chen et al. 2016 ; Li et al. 2022
BtaF37	Bta07948	Fungi	/	30.8	29.7	1319	99.8	0.92	BTA017217.1,<br>BTA017221.1,<br>BTA021006.1	Ssa00835	Tv_08362-RA	MYND finger family protein		Chen et al. 2016 ; Li et al. 2022
BtaF38	Bta00038<br>Bta00039<br>Bta05684<br>Bta05753<br>Bta05981<br>Bta15602	Fungi	/	56.4<br>55.3<br>48.7<br>57.1<br>40.0<br>48.1	53.2<br>52.1<br>46.0<br>49.7<br>37.7<br>45.5	386<br>388<br>379<br>155<br>371<br>369	74.6<br>75.9<br>92.4<br>78.3<br>79.7<br>89.2	0.44<br>0.41<br>0.3<br>0.75<br>0.82<br>/	BTA016364.1,<br>BTA022954.1,<br>BTA008707.1,<br>BTA006705.1,<br>BTA008708.1	Ssa05078,<br>Ssa10939,<br>Ssa10938,<br>Ssa10588,<br>Ssa10589,<br>Ssa12367,<br>Ssa06267,<br>Ssa12366		Aromatic peroxygenase		Chen et al. 2016 ; Li et al. 2022<br>Chen et al. 2016 ; Li et al. 2022<br>Chen et al. 2016 ; Li et al. 2022<br>Chen et al. 2016<br>Chen et al. 2016 ; Li et al. 2022<br>Chen et al. 2016 ; Li et al. 2022

Viridiplantae donor:

HGT event	Sequence name	Origin of donor sequences	Alternative topology	Similarities with the donor sequences		Length and coverage of the alignment with the donor sequences		Local score for genomic environment	Homologs found in the two other *B. tabaci* cryptic species		Homologs found in *T. vaporarorium*	Annotation (WGD version 1.2)	CAZy	Described in literature
				max id	average id	average aln length	average coverage		*B. tabaci* MED	*B. tabaci* SSA-ECA				
BtaV01	Bta06115<br>Bta06118	Viridiplantae	/	71.2<br>58.8	66.4<br>56.8	318<br>318	91.8<br>87.4	0.82<br>0.81	BTA001223.1, Scaffold_1494_158262_155412	Ssa15230, scaffold6392_704_1667		Glucan endo-1,3-beta-glucosidase	GH17	Gilbert and Maumus 2022<br>Gilbert and Maumus 2022 ; Li et al. 2022
BtaV02	Bta13961	Viridiplantae	/	59.0	54.7	232	93.6	0.96	BTA005662.2	Ssa10394	Tv_15928-RA	Thaumatin-like protein 1a	GH152	Gilbert and Maumus 2022
BtaV03	Bta02746	Viridiplantae	/	50.0	47.7	636	98.1	0.83	BTA017301.1, BTA020608.1	Ssa06068		Plant/T31B5-30 protein		Gilbert and Maumus 2022
BtaV04	Bta04871	Viridiplantae	/	66.8	60.3	302	86.1	0.96	BTA009091.1	Ssa10894		Nicotianamine synthase, putative		Gilbert and Maumus 2022
BtaV05	Bta11576	Viridiplantae	/	59.3	57.3	268	96.2	0.96	Scaffold_1194_116759_114636	scaffold89828_1484_481		Sterol desaturase, putative		Gilbert and Maumus 2022 ; Li et al. 2022
BtaV06	Bta14387	Viridiplantae	/	82.1	76.2	233	95.0	0.92	BTA010079.1	Ssa02799 , Ssa10266		Pathogen-related protein, related		Gilbert and Maumus 2022
BtaV07	Bta11842	Viridiplantae	/	79.5	70.3	179	74.9	0.96				Transferase, transferring glycosyl groups, putative		Gilbert and Maumus 2022 ; Li et al. 2022
BtaV08	Bta02650<br>Bta07786	Viridiplantae	/	42.5<br>39.7	39.6<br>37.5	455<br>465	98.5<br>97.6	0.59<br>0.96	BTA005164.2, BTA023005.1	Ssa02576, Ssa02578		Anthocyanin 5-aromatic		Xia et al. 2021 ; Gilbert and Maumus<br>Xia et al. 2021 ; Gilbert and Maumus 2022 ; Li et al. 2022
BtaV09	Bta05651<br>Bta05662<br>Bta01560<br>Bta01561	Viridiplantae	/	63.8<br>66.0<br>57.8<br>57.8	57.7<br>55.9<br>53.5<br>53.0	243<br>148<br>465<br>318	97.3<br>67.4<br>91.8<br>93.5	0.44<br>0.82<br>0.8<br>0.8	BTA018079.1, BTA025205.1	Ssa00364, Ssa03783 Ssa14466		HXXXD-type acyl-transferase family		Gilbert and Maumus 2022<br>Gilbert and Maumus 2022<br>Gilbert and Maumus 2022 ; Li et al. 2022<br>Gilbert and Maumus 2022
BtaV10	Bta04575<br>Bta05675<br>Bta05676	Viridiplantae	1	59.0<br>61.8<br>61.4	54.9<br>58.9<br>58.6	174<br>392<br>394	97.5<br>94.4<br>97.6	0.88<br>0.67<br>0.67	BTA022947.1, Scaffold_44_980879_981397	Ssa09262		Ornithine decarboxylase		Gilbert and Maumus 2022<br>Gilbert and Maumus 2022<br>Gilbert and Maumus 2022
BtaV11	Bta08851	Viridiplantae	/	73.5	69.1	401	97.6	1.0	BTA001066.1	Ssa14480		Alpha-(1,4)-fucosyltransferase-like protein	GT10	Gilbert and Maumus 2022
BtaV12	Bta08104	Viridiplantae	/	65.4	59.3	92	71.2	0.52	BTA002345.1	Ssa14505		Subtilisin-like protease		Gilbert and Maumus 2022 ; Li et al. 2022
BtaV13	Bta01681<br>Bta13782<br>Bta14307	Viridiplantae	/	64.5<br>73.6<br>69.5	60.3<br>66.3<br>62.8	261<br>260<br>222	98.8<br>99.1<br>96.4	0.77<br>1.0<br>0.96	BTA015001.1, BTA022461.1	Ssa04825, Ssa10246, Ssa12462, Ssa13979		S-adenosylmethionine-dependent methyltransferase, putative		Gilbert and Maumus 2022 ; Li et al. 2022<br>Gilbert and Maumus 2022 ; Li et al. 2022<br>Gilbert and Maumus 2022
BtaV14	Bta02345<br>Bta09738	Viridiplantae	/	59.9<br>70.3	57.7<br>67.5	698<br>697	97.8<br>95.1	0.82<br>0.89	BTA019832.1	Ssa01449, Ssa06609		Phenylalanine ammonia-lyase		Li et al. 2022<br>Li et al. 2022
BtaV15	Bta08293	Viridiplantae	/	61.9	55.4	387	97.5	0.65	BTA010343.2	Ssa07619		Chalcone synthase		Gilbert and Maumus 2022 ; Li et al. 2022
BtaV16	Bta14404	Viridiplantae	/	78.1	75.6	355	86.6	0.87	Scaffold_51_1080200_1078090	Ssa04498		Glutelin type-A 1		Gilbert and Maumus 2022 ; Li et al. 2022
BtaV17	Bta07485	Viridiplantae	/	67.6	64.8	365	85.5	0.81	BTA008512.1, BTA019386.1, BTA019387.1	Ssa04263		Purple acid phosphatase 29		Gilbert and Maumus 2022 ; Li et al. 2022
BtaV18	Bta05648	Viridiplantae	/	68.1	64.5	120	17.1	0.56	Scaffold_1460_66224_62822	Ssa11172		HXXXD-type acyl-transferase family protein		No
BtaV19	Bta07668	Viridiplantae	/	69.6	64.4	464	65.0	0.92	BTA014198.1	scaffold31037_32915_31767		Proteasome-associated ATPase		Gilbert and Maumus 2022
BtaV20	Bta04264<br>Bta06451<br>Bta06452<br>Bta06453<br>Bta09295<br>Bta13659<br>Bta15754<br>Bta15755<br>Bta15756<br>Bta15757<br>Bta15759<br>Bta15760<br>Bta15762<br>Bta15765	Viridiplantae	/	52.5<br>52.3<br>49.5<br>50.6<br>76.0<br>62.9<br>57.1<br>65.6<br>60.1<br>57.1<br>55.9<br>54.6<br>51.6<br>65.7	49.6<br>51.2<br>47.2<br>47.9<br>73.8<br>61.6<br>53.9<br>63.2<br>58.4<br>55.2<br>52.6<br>52.3<br>49.9<br>63.5	370<br>366<br>364<br>364<br>383<br>393<br>212<br>380<br>362<br>363<br>166<br>145<br>368<br>363	96.0<br>61.1<br>96.1<br>88.4<br>99.4<br>97.3<br>81.3<br>98.9<br>94.3<br>95.9<br>93.4<br>83.8<br>93.5<br>81.8	0.88<br>0.45<br>0.39<br>0.49<br>1.0<br>0.87<br>0.1<br>0.2<br>0.2<br>0.2<br>0.2<br>0.2<br>0.16<br>0.16	BTA015596.1, BTA007356.1, BTA011448.1, BTA015354.2, BTA015355.1, BTA028840.1, BTA011446.1	Ssa06732, Ssa02331, Ssa02332, Ssa02334, Ssa02342, Ssa03431, Ssa08612, Ssa11969, Ssa15146, Ssa15387, Ssa02337, Ssa02338, Ssa03432	Tv_08972-RA	Omega-6 fatty acid desaturase		Gilbert and Maumus 2022<br>Gilbert and Maumus 2022<br>Gilbert and Maumus 2022<br>Gilbert and Maumus 2022<br>Gilbert and Maumus 2022<br>Gilbert and Maumus 2022<br>Gilbert and Maumus 2022<br>Gilbert and Maumus 2022<br>Gilbert and Maumus 2022<br>Gilbert and Maumus 2022<br>Gilbert and Maumus 2022<br>Gilbert and Maumus 2022<br>Gilbert and Maumus 2022<br>Gilbert and Maumus 2022

BtaV21	Bta14371<br>Bta14372	Viridiplantae	/	58.2<br>59.9	55.9<br>55.1	122<br>488	97.5<br>54.3	0.39<br>0.45	Scaffold_688_163<br>109_167894,<br>Scaffold_688_157<br>148_164111	Ssa04520,<br>Ssa04521,<br>Ssa04522,<br>Ssa04523		HXXXD-type acyl-transferase family		Gilbert and Maumus 2022<br>Gilbert and Maumus 2022
BtaV22	Bta13094<br>Bta13103	Viridiplantae	/	33.0<br>35.6	30.7<br>32.9	185<br>197	95.8<br>69.6	0.86<br>0.82	BTA025985.1,<br>BTA025994.1	Ssa09189 ,<br>scaffold212068_5<br>4_492	TLow_01580-RA,<br>Tv_15540-RA,<br>Tv_15541-RA	rRNA N-glycosidase		Lapadula et al. 2020<br>Lapadula et al. 2020 ; Gilbert and Maumus 2022 ; Li et al. 2022
BtaV23	Bta15659	Viridiplantae	/	71.8	67.7	364	96.2	0.96	BTA003879.1	Ssa05099		Beta-1,4-N-acetylglucosaminyltransferase family protein	GT17	Gilbert and Maumus 2022
BtaV24	Bta14885	Viridiplantae	/	60.3	55.9	507	98.1	0.94	BTA018849.1,<br>BTA025653.1	Ssa09787		Beta-1,2-xylosyltransferase	GT61	Gilbert and Maumus 2022 ; Li et al. 2022
BtaV25	Bta11221<br>Bta11222	Viridiplantae	/	59.4<br>53.5	54.8<br>50.8	549<br>446	66.0<br>86.3	0.86<br>0.86	Scaffold_1904_91<br>816_77676,<br>Scaffold_1904_11	Ssa12987,<br>scaffold471_3996<br>9_63131		Pectinesterase	CE8	Gilbert and Maumus 2022<br>Gilbert and Maumus 2022
BtaV26	Bta07117	Viridiplantae	/	62.0	59.1	185	95.1	0.38	Scaffold_3373_12<br>48_9134	scaffold168257_1<br>4_362		Ureidoglycolate hydrolase		Gilbert and Maumus 2022 ; Li et al. 2022
BtaV27	Bta15239	Viridiplantae	/	69.8	64.1	119	87.6	1.0	BTA026135.1	Ssa02751		Expansin-like EG45 domain-containing protein		Li et al. 2022

Complex: Bacterial or Fungal donor

HGT event	Sequence name	Origin of donor sequences	Alternative topology	Similarities with the donor sequences		Length and coverage of the alignment with the donor sequences		Local score for genomic environment	Homologs found in the two other *B. tabaci* cryptic species		Homologs found in *T. vaporarorium*	Annotation (WGD version 1.2)	CAZy	Described in literature
				max id	average id	average aln length	average coverage		*B. tabaci* MED	*B. tabaci* SSA-ECA				
BtaC01	Bta02634	Bacteria or Fungi	0	34.4	31.1	350	83.9	0.6	BTA008786.1	Ssa01521	Tv_05105-RA	Pectin lyase	PL1_4	No

Supplementary Table 3: HGT candidates described in the literature for *B. tabaci* MEAM1 and not found in our study

Sequence name	Literature	Reason why it was not found in our study
Bta00456	Bacteria (Chen et al. 2016)	No longer found in the 1.2 version of the proteome.
Bta02353	Bacteria (Li et al. 2022)	Identity between the donor sequences and the HGT candidate sequence in the DIAMOND homology search results was less than 30%.
Bta02480	Bacteria (Chen et al. 2016 ; Li et al. 2022)	AHS ≤ 0
Bta07164	Bacteria (Chen et al. 2016)	AHS ≤ 0
Bta11397	Bacteria (Chen et al. 2016)	AHS ≤ 0
Bta12968	Bacteria (Li et al. 2022)	Classified as non-HGT due to the presence of other metazoan sequences in the sister branch and in the ancestral sister branch. The alternative topology constructed by grouping all metazoan sequences was not significantly different from the unconstrained topology, confirming the classification as non-HGT.
Bta13226	Bacteria (Chen et al. 2016)	AHS ≤ 0
Bta13776	Bacteria (Chen et al. 2016)	AHS ≤ 0
Bta13944	Bacteria (Li et al. 2022)	AHS ≤ 0
Bta14500	Bacteria (Chen et al. 2016 ; Li et al. 2022)	AHS ≤ 0
Bta14724	Bacteria (Li et al. 2022)	AHS ≤ 0
Bta02218	Fungi (Chen et al. 2016)	No similarity found with NR in the Diamond result. The lack of similarity was confirmed by BLASTP against NR at NCBI.
Bta08969	Fungi (Chen et al. 2016)	HGT_COMPLEX: Unclear whether donor origin was bacterial or fungal.
Bta13068	Viridiplantae (Gilbert and Maumus 2022)	No similarity found with NR in the Diamond result. The lack of similarity was confirmed by BLASTP against NR at NCBI.

Supplementary Table 4: Overrepresented GO terms among validated HGT candidates from potential bacterial, fungal or viridiplantae donors for *B. tabaci* MEAM1

root_node_name	node_id	node_name	raw_p_overrep	FWER_overrep	refined_p_overrep	FDR_overrep	nb_genes_refset_root_node	nb_genes_candidate_in_root	nb_gene_refset_node	nb_genes_candidate_in_node	name_genes_candidate_in_node
HGT Bacteria											
molecular_function	GO:0000287	magnesium ion binding	4.78709249865742e-08	0	4.78709249865742e-08	1.66776125759678e-07	6169	44	34	6	Bta00747|Bta00840|Bta01938|Bta03871|
molecular_function	GO:0016866	intramolecular transferase activity	2.37339959024691e-11	0	2.37339959024691e-11	1.60204472341666e-10	6169	44	21	7	Bta02625|Bta07024|Bta07125|Bta07126|Bta07870|Bta11369|Bta11370
molecular_function	GO:0016879	ligase activity, forming carbon-nitrogen bonds	2.27534974684758e-07	0	0.0002757252188581	6.30096852973176e-07	6169	44	23	5	Bta00062|Bta00840|Bta01938|Bta05339|Bta05813
molecular_function	GO:0004170	dUTP diphosphatase activity	2.2224899964394e-06	0.001	2.2224899964394e-06	5.33397599145455e-06	6169	44	5	3	Bta00747|Bta05687|Bta13975
molecular_function	GO:0004141	dethiobiotin synthase activity	3.77712517891934e-05	0.013	3.77712517891934e-05	5.16366480156062e-05	6169	44	2	2	Bta00840|Bta01938
molecular_function	GO:0008836	diaminopimelate decarboxylase activity	3.77712517891934e-05	0.013	3.77712517891934e-05	5.16366480156062e-05	6169	44	2	2	Bta03589|Bta03593
cellular_component	GO:0005811	lipid droplet	8.69071715427881e-18	0	8.69071715427881e-18	9.38597452662112e-16	2247	10	8	7	Bta02625|Bta07024|Bta07125|Bta07126|
biological_process	GO:0008610	lipid biosynthetic process	2.19831675341128e-12	0	0.000189397237390966	1.8262939182186e-11	4287	43	102	13	Bta02625|Bta02653|Bta04469|Bta05264|Bta06818|Bta07024|Bta07125|Bta07126|
biological_process	GO:0009089	lysine biosynthetic process via diaminopimelate	5.97004604987519e-09	0	5.97004604987519e-09	2.47986528225585e-08	4287	43	4	4	Bta03589|Bta03593|Bta06657|Bta20020
biological_process	GO:0009102	biotin biosynthetic process	5.97004604987519e-09	0	5.97004604987519e-09	2.47986528225585e-08	4287	43	4	4	Bta00840|Bta01937|Bta01938|Bta09725
biological_process	GO:0016104	triterpenoid biosynthetic process	3.12108961795685e-15	0	3.12108961795685e-15	4.81539541056199e-14	4287	43	7	7	Bta02625|Bta07024|Bta07125|Bta07126|Bta07870|Bta11369|Bta11370
biological_process	GO:0006226	dUMP biosynthetic process	6.95241478593945e-06	0.002	6.95241478593945e-06	1.31729964365169e-05	4287	43	5	3	Bta00747|Bta05687|Bta13975
biological_process	GO:0046081	dUTP catabolic process	6.95241478593945e-06	0.002	6.95241478593945e-06	1.31729964365169e-05	4287	43	5	3	Bta00747|Bta05687|Bta13975
biological_process	GO:0006526	arginine biosynthetic process	8.10478963448566e-05	0.029	8.10478963448566e-05	0.000104204438157673	4287	43	2	2	Bta00062|Bta00063
HGT Fungi											
molecular_function	GO:0004601	peroxidase activity	3.66430096659827e-31	0	3.66430096659827e-31	2.19858057995896e-30	6169	45	49	19	Bta00038|Bta00039|Bta00041|Bta00658|Bta00659|Bta00660|Bta01073|Bta02217|Bta04808|Bta05684|Bta05753|Bta05754|
HGT Viridiplantae											
molecular_function	GO:0016717	oxidoreductase activity, acting on paired donors, with oxidation of a pair of donors resulting in the reduction of molecular oxygen to two molecules of water	1.26787439093126e-10	0	1.26787439093126e-10	2.09199274503657e-09	6169	29	20	6	Bta06452|Bta09295|Bta13659|Bta15755|Bta15756|Bta15765
molecular_function	GO:0030598	rRNA N-glycosylase activity	1.73683874506334e-05	0.003	1.73683874506334e-05	4.77630654892417e-05	6169	29	2	2	Bta13094|Bta13103
molecular_function	GO:0030599	pectinesterase activity	1.73683874506334e-05	0.003	1.73683874506334e-05	4.77630654892417e-05	6169	29	2	2	Bta11221|Bta11222
molecular_function	GO:0016841	ammonia-lyase activity	5.19677076179776e-05	0.005	5.19677076179776e-05	0.000107183396962079	6169	29	3	2	Bta02345|Bta09738
biological_process	GO:0006596	polyamine biosynthetic process	4.5738072745559e-06	0	4.5738072745559e-06	1.67706266733716e-05	4408	26	6	3	Bta04575|Bta05675|Bta05676
biological_process	GO:0006629	lipid metabolic process	1.99651433804412e-12	0	1.99651433804412e-12	6.58849731554559e-11	4408	26	240	15	Bta04264|Bta06451|Bta06452|Bta06453|Bta09295|Bta11576|Bta13659|Bta15754|
biological_process	GO:0042545	cell wall modification	3.90430065448644e-05	0.011	3.90430065448644e-05	8.58946143987016e-05	4408	26	2	2	Bta11221|Bta11222

HGT Bacteria + Fungi + Viridiplantae										
molecular_function	GO:0004601	peroxidase activity	5.45718553136594e-22	0	5.45718553136594e-22	3.41074095710371e-20	6673	118	49	19	Bta00038|Bta00039|Bta00041|Bta00658|Bta00659|Bta00660|Bta01073|Bta02217|Bta04808|Bta05684|Bta05753|Bta05754|Bta05981|Bta07721|Bta08225|Bta08226|Bta11373|Bta15601|Bta15602
molecular_function	GO:0016717	oxidoreductase activity, acting on paired donors, with oxidation of a pair of donors resulting in the reduction of molecular oxygen to two molecules of water	6.3644593518552e-07	0	6.3644593518552e-07	2.20988171939417e-06	6673	118	20	6	Bta06452|Bta09295|Bta13659|Bta15755|Bta15756|Bta15765
molecular_function	GO:0016866	intramolecular transferase activity	3.04349973779946e-08	0	3.04349973779946e-08	1.40902765638864e-07	6673	118	21	7	Bta02625|Bta07024|Bta07125|Bta07126|Bta07870|Bta11369|Bta11370
molecular_function	GO:0016841	ammonia-lyase activity	4.63518691969453e-06	0.001	4.63518691969453e-06	1.25956166296047e-05	6673	118	3	3	Bta02345|Bta08776|Bta09738
molecular_function	GO:0000287	magnesium ion binding	1.82189865098747e-05	0.007	1.82189865098747e-05	4.46543787006732e-05	6673	118	34	6	Bta00747|Bta00840|Bta01938|Bta03871|Bta05687|Bta13975
molecular_function	GO:0016798	hydrolase activity, acting on glycosyl bonds	1.95323653630582e-05	0.007	1.95323653630582e-05	4.69528013535052e-05	6673	118	91	9	Bta00773|Bta02250|Bta02658|Bta02866|Bta04549|Bta06115|Bta06118|Bta13094|Bta13103
molecular_function	GO:0016879	ligase activity, forming carbon-nitrogen bonds	3.26094908050568e-05	0.007	3.26094908050568e-05	7.02790750108982e-05	6673	118	23	5	Bta00062|Bta00840|Bta01938|Bta05339|Bta05813
molecular_function	GO:0004170	dUTP diphosphatase activity	4.52193208416261e-05	0.008	4.52193208416261e-05	8.07487872171894e-05	6673	118	5	3	Bta00747|Bta05687|Bta13975
cellular_component	GO:0005811	lipid droplet	4.6521100294467e-16	0	4.6521100294467e-16	9.69189589468063e-15	1834	9	8	7	Bta02625|Bta07024|Bta07125|Bta07126|Bta07870|Bta11369|Bta11370
biological_process	GO:0006629	lipid metabolic process	1.03172125882258e-19	0	4.11302896236752e-09	2.57930314705646e-18	4643	77	240	30	Bta02625|Bta02653|Bta02658|Bta04264|Bta04469|Bta05264|Bta06451|Bta06452|Bta06453|Bta06818|Bta07024|Bta07125|Bta07126|Bta07870|Bta09295|Bta11043|Bta11369|Bta11370|Bta11576|Bta11911|Bta11912|Bta13659|Bta15754|Bta15755|Bta15756|Bta15757|Bta15759|Bta15760|Bta15762|Bta15765
biological_process	GO:0008610	lipid biosynthetic process	5.22715083728764e-11	0	0.000147337329446341	4.27575861363102e-10	4643	77	102	15	Bta02625|Bta02653|Bta04469|Bta05264|Bta06818|Bta07024|Bta07125|Bta07126|Bta07870|Bta11043|Bta11369|Bta11370|Bta11576|Bta11911|Bta11912
biological_process	GO:0009089	lysine biosynthetic process via diaminopimelate	6.90043184238973e-08	0	6.90043184238973e-08	2.61379994029914e-07	4643	77	4	4	Bta03589|Bta03593|Bta06657|Bta20020
biological_process	GO:0009102	biotin biosynthetic process	6.90043184238973e-08	0	6.90043184238973e-08	2.61379994029914e-07	4643	77	4	4	Bta00840|Bta01937|Bta01938|Bta09725
biological_process	GO:0016053	organic acid biosynthetic process	5.98680141923098e-14	0	0.000238330962574668	9.35437721754841e-13	4643	77	43	13	Bta00062|Bta00063|Bta00840|Bta01937|Bta01938|Bta03589|Bta03593|Bta04871|Bta05339|Bta06657|Bta09725|Bta20014|Bta20020
biological_process	GO:0016104	triterpenoid biosynthetic process	2.55883320124494e-13	0	2.55883320124494e-13	2.66545125129681e-12	4643	77	7	7	Bta02625|Bta07024|Bta07125|Bta07126|Bta07870|Bta11369|Bta11370
biological_process	GO:0019752	carboxylic acid metabolic process	7.50404068388319e-11	0	0.000211410856127128	5.21113936380777e-10	4643	77	164	18	Bta00062|Bta00063|Bta00840|Bta01937|Bta01938|Bta03200|Bta03589|Bta03593|Bta04871|Bta05339|Bta06657|Bta06820|Bta08776|Bta09725|Bta09738|Bta15103|Bta20014|Bta20020

<table>
<tr><td>biological_process</td><td>GO:0046394</td><td>carboxylic acid biosynthetic process</td><td>5.98680141923098e-14</td><td>0</td><td>0.000238330962574668</td><td>9.35437721754841e-13</td><td>4643</td><td>77</td><td>43</td><td>13</td><td>Bta00062|Bta00063|Bta00840|Bta01937|Bta01938|Bta03589|Bta03593|Bta04871|Bta05339|Bta06657|Bta09725|Bta20014|Bta20020</td></tr>
<tr><td>biological_process</td><td>GO:0006226</td><td>dUMP biosynthetic process</td><td>4.23797470126717e-05</td><td>0.01</td><td>4.23797470126717e-05</td><td>7.67749040084632e-05</td><td>4643</td><td>77</td><td>5</td><td>3</td><td>Bta00747|Bta05687|Bta13975</td></tr>
<tr><td>biological_process</td><td>GO:0046081</td><td>dUTP catabolic process</td><td>4.23797470126717e-05</td><td>0.01</td><td>4.23797470126717e-05</td><td>7.67749040084632e-05</td><td>4643</td><td>77</td><td>5</td><td>3</td><td>Bta00747|Bta05687|Bta13975</td></tr>
<tr><td>biological_process</td><td>GO:0006596</td><td>polyamine biosynthetic process</td><td>8.37502425309647e-05</td><td>0.021</td><td>8.37502425309647e-05</td><td>0.000134215132261161</td><td>4643</td><td>77</td><td>6</td><td>3</td><td>Bta04575|Bta05675|Bta05676</td></tr>
</table>

Supplementary Table 5: Predicted CAZymes in *B. tabaci* , *T. vaporariorum* , *F. occidentalis* and *T. palmi*

B. tabaci

Protein ID	Description	Model notes	Definition line	HGT	Donor
Bta00067	AA3_2		GMC oxidoreductase		
Bta00205	GT105	fragment N-term;	Glycosyltransferase Family 105 protein		
Bta00245	GH18-CBM18-CBM18-CBM18		Glycoside Hydrolase Family 18 / Carbohydrate-Binding Module Family 18 protein		
Bta00300	GT1		Glycosyltransferase Family 1 protein		
Bta00304	CBM21		Carbohydrate-Binding Module Family 21 protein		
Bta00306	AA1		Multicopper oxidase		
Bta00307	GT16		Glycosyltransferase Family 16 protein		
Bta00329	GH37		Glycoside Hydrolase Family 37 protein		
Bta00445	GT27-CBM13		Glycosyltransferase Family 27 / Carbohydrate-Binding Module Family 13 protein		
Bta00548	GT1		Glycosyltransferase Family 1 protein		
Bta00633	GT66	fragment C-term;	Glycosyltransferase Family 66 protein		
Bta00718	GT1		Glycosyltransferase Family 1 protein		
Bta00731	GT27-CBM13		Glycosyltransferase Family 27 / Carbohydrate-Binding Module Family 13 protein		
Bta00773	GH32		Glycoside Hydrolase Family 32 protein	HGT	Bacteria
Bta00822	GT1		Glycosyltransferase Family 1 protein		
Bta00951	GT23	(fragment) splicing problem;	Glycosyltransferase Family 23 protein		
Bta00966	GT31		Glycosyltransferase Family 31 protein		
Bta00998	GH89	(fragment) splicing problem;	Glycoside Hydrolase Family 89 protein		
Bta01019	GT31		Glycosyltransferase Family 31 protein		
Bta01062	GT47-GT64		Glycosyltransferase Family 47 / Glycosyltransferase Family 64 protein		
Bta01072	CBM32-AA5_2		Carbohydrate-Binding Module Family 32 / Galactose oxidase	HGT	Fungi
Bta01126	GH79	fragment N-term;	Glycoside Hydrolase Family 79 protein		
Bta01138	GT1	fragment C-term;	Glycosyltransferase Family 1 protein		
Bta01168	GT4		Glycosyltransferase Family 4 protein		
Bta01169	GT8		Glycosyltransferase Family 8 protein		
Bta01181	GT1	fragment N-term;	Glycosyltransferase Family 1 protein		
Bta01182	GT1-GT1		Glycosyltransferase Family 1 protein		
Bta01183	GT1		Glycosyltransferase Family 1 protein		
Bta01184	GT1		Glycosyltransferase Family 1 protein		
Bta01219	GH47		Glycoside Hydrolase Family 47 protein		
Bta01304	GT1		Glycosyltransferase Family 1 protein		
Bta01342	GH18-GH18-CBM14		Glycoside Hydrolase Family 18 / Carbohydrate-Binding Module Family 14 protein		
Bta01403	GT27-CBM13		Glycosyltransferase Family 27 / Carbohydrate-Binding Module Family 13 protein		
Bta01405	GH47		Glycoside Hydrolase Family 47 protein		
Bta01463	GH13_17	fragment N-term;	Glycoside Hydrolase Family 13 protein		
Bta01464	GH13	fragment N-term / C-term;	Glycoside Hydrolase Family 13 protein		
Bta01468	GH63		Glycoside Hydrolase Family 63 protein		
Bta01478	GH13_17		Glycoside Hydrolase Family 13 protein		
Bta01485	GH2		Glycoside Hydrolase Family 2 protein		
Bta01758	GT31	(fragment) splicing problem;	Glycosyltransferase Family 31 protein		
Bta01766	GT1	fragment N-term;	Glycosyltransferase Family 1 protein		
Bta01777	CBM47		Carbohydrate-Binding Module Family 47 protein		
Bta01963	GT68		Glycosyltransferase Family 68 protein		
Bta01975	CBM14-CBM14-CBM14		Carbohydrate-Binding Module Family 14 protein		

Bta01976	CBM14-CBM14-CBM14		Carbohydrate-Binding Module Family 14 protein		
Bta01977	CBM14-CBM14-CBM14		Carbohydrate-Binding Module Family 14 protein		
Bta01978	CBM14-CBM14-CBM14		Carbohydrate-Binding Module Family 14 protein		
Bta01979	CBM14-CBM14-CBM14		Carbohydrate-Binding Module Family 14 protein		
Bta01980	CBM14-CBM14-CBM14		Carbohydrate-Binding Module Family 14 protein		
Bta01982	CBM14-CBM14-CBM14	(fragment) splicing problem;	Carbohydrate-Binding Module Family 14 protein		
Bta02118	GH30_1		Glycoside Hydrolase Family 30 protein		
Bta02176	GH84		Glycoside Hydrolase Family 84 protein		
Bta02228	GT1		Glycosyltransferase Family 1 protein		
Bta02242	GT2		Glycosyltransferase Family 2 protein		
Bta02250	GH49		Glycoside Hydrolase Family 49 protein	HGT	Fungi
Bta02273	GH18		Glycoside Hydrolase Family 18 protein		
Bta02274	GH18		Glycoside Hydrolase Family 18 protein		
Bta02288	GT41		Glycosyltransferase Family 41 protein		
Bta02306	GT1		Glycosyltransferase Family 1 protein		
Bta02326	GT1		Glycosyltransferase Family 1 protein		
Bta02348	GT1		Glycosyltransferase Family 1 protein		
Bta02350	GT32		Glycosyltransferase Family 32 protein		
Bta02395	AA3_2		GMC oxidoreductase		
Bta02396	AA3_2	(fragment) splicing problem;	GMC oxidoreductase		
Bta02397	AA3_2	fragment N-term;	GMC oxidoreductase		
Bta02398	AA3_2		GMC oxidoreductase		
Bta02399	AA3_2	(fragment) splicing problem;	GMC oxidoreductase		
Bta02400	AA3_2		GMC oxidoreductase		
Bta02401	AA3_2	fragment C-term;	GMC oxidoreductase		
Bta02402	AA3_2	fragment N-term;	GMC oxidoreductase		
Bta02405	AA3_2-AA3_2	(fragment) splicing problem;	GMC oxidoreductase		
Bta02406	AA3_2		GMC oxidoreductase		
Bta02407	AA3_2		GMC oxidoreductase		
Bta02440	AA3_2		GMC oxidoreductase		
Bta02521	CBM14		Carbohydrate-Binding Module Family 14 protein		
Bta02522	GH37		Glycoside Hydrolase Family 37 protein		
Bta02601	GT1		Glycosyltransferase Family 1 protein		
Bta02602	GT1		Glycosyltransferase Family 1 protein		
Bta02603	GT1		Glycosyltransferase Family 1 protein		
Bta02604	GT1		Glycosyltransferase Family 1 protein		
Bta02622	CBM47	fragment N-term;	Carbohydrate-Binding Module Family 47 protein		
Bta02630	GH13_15		Glycoside Hydrolase Family 13 protein		
Bta02634	PL1_4	fragment C-term;	Polysaccharide Lyase Family 1 protein	HGT	Complex: Bacteria or Fungi
Bta02658	GH30_3		Glycoside Hydrolase Family 30 protein	HGT	Fungi
Bta02682	GH116		Glycoside Hydrolase Family 116 protein		
Bta02725	GT1		Glycosyltransferase Family 1 protein		
Bta02726	GT1		Glycosyltransferase Family 1 protein		
Bta02795	GT7		Glycosyltransferase Family 7 protein		
Bta02810	GH13_17		Glycoside Hydrolase Family 13 protein		
Bta02866	GH71		Glycoside Hydrolase Family 71 protein	HGT	Fungi
Bta02947	AA3_2	fragment C-term;	GMC oxidoreductase		
Bta02948	AA3_2	fragment N-term;	GMC oxidoreductase		
Bta02973	AA3_2		GMC oxidoreductase		

Bta02974	AA3_2		GMC oxidoreductase		
Bta02991	GT1		Glycosyltransferase Family 1 protein		
Bta03001	AA3_2		GMC oxidoreductase		
Bta03112	GH13_17	fragment N-term;	Glycoside Hydrolase Family 13 protein		
Bta03142	GH22		Glycoside Hydrolase Family 22 protein		
Bta03157	GT59		Glycosyltransferase Family 59 protein		
Bta03192	GT1		Glycosyltransferase Family 1 protein		
Bta03195	CBM14		Carbohydrate-Binding Module Family 14 protein		
Bta03197	GH37		Glycoside Hydrolase Family 37 protein		
Bta03198	GH37		Glycoside Hydrolase Family 37 protein		
Bta03202	GH37		Glycoside Hydrolase Family 37 protein		
Bta03221	GH38		Glycoside Hydrolase Family 38 protein		
Bta03307	GH13_17		Glycoside Hydrolase Family 13 protein		
Bta03340	GH47		Glycoside Hydrolase Family 47 protein		
Bta03431	GT1		Glycosyltransferase Family 1 protein		
Bta03439	GH13_17		Glycoside Hydrolase Family 13 protein		
Bta03440	GT1	fragment N-term;	Glycosyltransferase Family 1 protein		
Bta03567	CBM32-AA5_2		Carbohydrate-Binding Module Family 32 / Galactose oxidase	HGT	Fungi
Bta03574	GT13		Glycosyltransferase Family 13 protein		
Bta03601	GT2		Glycosyltransferase Family 2 protein		
Bta03753	CBM50		Carbohydrate-Binding Module Family 50 protein		
Bta03818	GH13_17		Glycoside Hydrolase Family 13 protein		
Bta03820	GT105	fragment C-term;	Glycosyltransferase Family 105 protein		
Bta03913	GT49		Glycosyltransferase Family 49 protein		
Bta03920	GH38		Glycoside Hydrolase Family 38 protein		
Bta03960	GT4		Glycosyltransferase Family 4 protein		
Bta03991	GH13_17		Glycoside Hydrolase Family 13 protein		
Bta03992	GH13_17		Glycoside Hydrolase Family 13 protein		
Bta03994	GH13_17		Glycoside Hydrolase Family 13 protein		
Bta03995	GH13_17		Glycoside Hydrolase Family 13 protein		
Bta04042	GH13_17	fragment C-term;	Glycoside Hydrolase Family 13 protein		
Bta04297	GH13_17		Glycoside Hydrolase Family 13 protein		
Bta04298	GH13_17		Glycoside Hydrolase Family 13 protein		
Bta04306	GH13_17		Glycoside Hydrolase Family 13 protein		
Bta04331	GH99	fragment N-term;	Glycoside Hydrolase Family 99 protein		
Bta04378	GT65		Glycosyltransferase Family 65 protein		
Bta04382	GH13_17	fragment C-term;	Glycoside Hydrolase Family 13 protein		
Bta04384	GH13	fragment N-term / C-term;	Glycoside Hydrolase Family 13 protein		
Bta04402	AA3_2		GMC oxidoreductase		
Bta04421	GH13_17		Glycoside Hydrolase Family 13 protein		
Bta04425	GH20		Glycoside Hydrolase Family 20 protein		
Bta04444	GT13		Glycosyltransferase Family 13 protein		
Bta04537	GT1	fragment N-term;	Glycosyltransferase Family 1 protein		
Bta04539	GT1	fragment N-term;	Glycosyltransferase Family 1 protein		
Bta04543	GT1	fragment C-term;	Glycosyltransferase Family 1 protein		
Bta04549	GH32		Glycoside Hydrolase Family 32 protein	HGT	Bacteria
Bta04553	GH13_15		Glycoside Hydrolase Family 13 protein		
Bta04680	GT54		Glycosyltransferase Family 54 protein		
Bta04683	GH13	fragment C-term;	Glycoside Hydrolase Family 13 protein		

Bta04691	GT25	fragment C-term;	Glycosyltransferase Family 25 protein		
Bta04694	GT54		Glycosyltransferase Family 54 protein		
Bta04731	GH13_15	fragment N-term;	Glycoside Hydrolase Family 13 protein		
Bta04732	GH13	fragment C-term;	Glycoside Hydrolase Family 13 protein		
Bta04739	GT1		Glycosyltransferase Family 1 protein		
Bta05024	GT31		Glycosyltransferase Family 31 protein		
Bta05026	GT27-CBM13		Glycosyltransferase Family 27 / Carbohydrate-Binding Module Family 13 protein		
Bta05090	CBM32-AA5_2		Carbohydrate-Binding Module Family 32 / Galactose oxidase	HGT	Fungi
Bta05091	CBM32-AA5_2		Carbohydrate-Binding Module Family 32 / Galactose oxidase	HGT	Fungi
Bta05092	AA5_2	fragment N-term;	Galactose oxidase	HGT	Fungi
Bta05093	CBM32-AA5_2		Carbohydrate-Binding Module Family 32 / Galactose oxidase	HGT	Fungi
Bta05237	GH38		Glycoside Hydrolase Family 38 protein		
Bta05251	GT8	fragment N-term / C-term;	Glycosyltransferase Family 8 protein		
Bta05309	GH35		Glycoside Hydrolase Family 35 protein		
Bta05340	GH13_17		Glycoside Hydrolase Family 13 protein		
Bta05342	GH13	fragment C-term;	Glycoside Hydrolase Family 13 protein		
Bta05343	GH13_17	fragment N-term;	Glycoside Hydrolase Family 13 protein		
Bta05386	GH13_17		Glycoside Hydrolase Family 13 protein		
Bta05396	GH13_17		Glycoside Hydrolase Family 13 protein		
Bta05397	GH13_17		Glycoside Hydrolase Family 13 protein		
Bta05466	GT105	(fragment) splicing problem;	Glycosyltransferase Family 105 protein		
Bta05518	GH13_17		Glycoside Hydrolase Family 13 protein		
Bta05547	CBM14		Carbohydrate-Binding Module Family 14 protein		
Bta05577	GT35		Glycosyltransferase Family 35 protein		
Bta05604	GT1		Glycosyltransferase Family 1 protein		
Bta05616	GT27-CBM13		Glycosyltransferase Family 27 / Carbohydrate-Binding Module Family 13 protein		
Bta05704	GH47		Glycoside Hydrolase Family 47 protein		
Bta05915	CBM50		Carbohydrate-Binding Module Family 50 protein		
Bta05951	GT1		Glycosyltransferase Family 1 protein		
Bta06017	GT1	(fragment) splicing problem;	Glycosyltransferase Family 1 protein		
Bta06059	GH13_17	fragment N-term;	Glycoside Hydrolase Family 13 protein		
Bta06072	CBM14		Carbohydrate-Binding Module Family 14 protein		
Bta06073	CBM14	(fragment) splicing problem;	Carbohydrate-Binding Module Family 14 protein		
Bta06115	GH17		Glycoside Hydrolase Family 17 protein	HGT	Viridiplantae
Bta06118	GH17		Glycoside Hydrolase Family 17 protein	HGT	Viridiplantae
Bta06208	GT92		Glycosyltransferase Family 92 protein		
Bta06327	GH13_17	(fragment) splicing problem;	Glycoside Hydrolase Family 13 protein		
Bta06329	GH18		Glycoside Hydrolase Family 18 protein		
Bta06441	GH13	fragment C-term;	Glycoside Hydrolase Family 13 protein		
Bta06445	GH13_17	(fragment) splicing problem;	Glycoside Hydrolase Family 13 protein		
Bta06458	GH13_17		Glycoside Hydrolase Family 13 protein		
Bta06497	GT90	(fragment) splicing problem;	Glycosyltransferase Family 90 protein		
Bta06513	GT31		Glycosyltransferase Family 31 protein		
Bta06581	CBM21		Carbohydrate-Binding Module Family 21 protein		
Bta06664	GT1		Glycosyltransferase Family 1 protein		
Bta06665	GT1		Glycosyltransferase Family 1 protein		
Bta06706	GH20	(fragment) splicing problem;	Glycoside Hydrolase Family 20 protein		
Bta06849	GH31		Glycoside Hydrolase Family 31 protein		
Bta06905	GT43		Glycosyltransferase Family 43 protein		

Bta06986	GH20		Glycoside Hydrolase Family 20 protein		
Bta06988	GT22		Glycosyltransferase Family 22 protein		
Bta07185	GT1		Glycosyltransferase Family 1 protein		
Bta07226	GT22		Glycosyltransferase Family 22 protein		
Bta07244	GT31		Glycosyltransferase Family 31 protein		
Bta07272	AA15		Auxilliary Activities Family 15 protein		
Bta07330	AA3_2	fragment N-term;	GMC oxidoreductase		
Bta07340	GT61		Glycosyltransferase Family 61 protein		
Bta07348	GT1		Glycosyltransferase Family 1 protein		
Bta07368	GT1		Glycosyltransferase Family 1 protein		
Bta07377	GH13	(fragment) splicing problem;	Glycoside Hydrolase Family 13 protein		
Bta07385	GT31		Glycosyltransferase Family 31 protein		
Bta07386	GT31	fragment N-term;	Glycosyltransferase Family 31 protein		
Bta07432	GT105	fragment N-term;	Glycosyltransferase Family 105 protein		
Bta07452	GH13_17	(fragment) splicing problem;	Glycoside Hydrolase Family 13 protein		
Bta07453	GH13_17		Glycoside Hydrolase Family 13 protein		
Bta07478	GT22		Glycosyltransferase Family 22 protein		
Bta07492	GT110		Glycosyltransferase Family 110 protein		
Bta07496	GT13		Glycosyltransferase Family 13 protein		
Bta07516	GH38		Glycoside Hydrolase Family 38 protein		
Bta07589	GT1	fragment N-term;	Glycosyltransferase Family 1 protein		
Bta07602	GT1		Glycosyltransferase Family 1 protein		
Bta07603	GT1	(fragment) splicing problem;	Glycosyltransferase Family 1 protein		
Bta07604	GT1	fragment N-term;	Glycosyltransferase Family 1 protein		
Bta07605	GT1		Glycosyltransferase Family 1 protein		
Bta07606	GT1-GT1	(fragment) splicing problem;	Glycosyltransferase Family 1 protein		
Bta07607	GT1	fragment C-term;	Glycosyltransferase Family 1 protein		
Bta07608	GT1		Glycosyltransferase Family 1 protein		
Bta07610	GT1	fragment N-term;	Glycosyltransferase Family 1 protein		
Bta07623	GT1		Glycosyltransferase Family 1 protein		
Bta07646	GT1	fragment N-term;	Glycosyltransferase Family 1 protein		
Bta07691	CBM32-AA5_2		Carbohydrate-Binding Module Family 32 / Galactose oxidase	HGT	Fungi
Bta07704	GT1		Glycosyltransferase Family 1 protein		
Bta07715	GT47-GT64		Glycosyltransferase Family 47 / Glycosyltransferase Family 64 protein		
Bta07764	GH13_17		Glycoside Hydrolase Family 13 protein		
Bta08022	CBM14		Carbohydrate-Binding Module Family 14 protein		
Bta08033	CBM14		Carbohydrate-Binding Module Family 14 protein		
Bta08063	GT7		Glycosyltransferase Family 7 protein		
Bta08065	GT4		Glycosyltransferase Family 4 protein		
Bta08213	GT1		Glycosyltransferase Family 1 protein		
Bta08294	GT1-GT1		Glycosyltransferase Family 1 protein		
Bta08372	GT1		Glycosyltransferase Family 1 protein		
Bta08425	GH13_17		Glycoside Hydrolase Family 13 protein		
Bta08426	GH13_17	fragment C-term;	Glycoside Hydrolase Family 13 protein		
Bta08427	GH13_17	(fragment) splicing problem;	Glycoside Hydrolase Family 13 protein		
Bta08431	GH13_17	fragment C-term;	Glycoside Hydrolase Family 13 protein		
Bta08458	GT20	(fragment) splicing problem;	Glycosyltransferase Family 20 protein		
Bta08517	GT8-GT49	fragment N-term;	Glycosyltransferase Family 8 / Glycosyltransferase Family 49 protein		
Bta08621	GH27		Glycoside Hydrolase Family 27 protein		

Bta08702	GT20		Glycosyltransferase Family 20 protein		
Bta08716	CBM20		Carbohydrate-Binding Module Family 20 protein		
Bta08743	GT1		Glycosyltransferase Family 1 protein		
Bta08748	GT27-CBM13	fragment C-term;	Glycosyltransferase Family 27 / Carbohydrate-Binding Module Family 13 protein		
Bta08851	GT10		Glycosyltransferase Family 10 protein	HGT	Viridiplantae
Bta08909	AA3_2		GMC oxidoreductase		
Bta08917	GH20		Glycoside Hydrolase Family 20 protein		
Bta08970	GH18	fragment C-term;	Glycoside Hydrolase Family 18 protein		
Bta09009	GT105		Glycosyltransferase Family 105 protein		
Bta09018	GH47		Glycoside Hydrolase Family 47 protein		
Bta09037	GH18-CBM14-CBM14		Glycoside Hydrolase Family 18 / Carbohydrate-Binding Module Family 14 protein		
Bta09066	CBM32-AA5_2		Carbohydrate-Binding Module Family 32 / Galactose oxidase	HGT	Fungi
Bta09154	GT31		Glycosyltransferase Family 31 protein		
Bta09170	GH18		Glycoside Hydrolase Family 18 protein		
Bta09254	GH16_4		Glycoside Hydrolase Family 16 protein		
Bta09256	GH16_4		Glycoside Hydrolase Family 16 protein		
Bta09257	GH16	(fragment) splicing problem;	Glycoside Hydrolase Family 16 protein		
Bta09258	GH16_4	fragment C-term;	Glycoside Hydrolase Family 16 protein		
Bta09259	GH16_4		Glycoside Hydrolase Family 16 protein		
Bta09289	GT1	fragment N-term;	Glycosyltransferase Family 1 protein		
Bta09300	GT32		Glycosyltransferase Family 32 protein		
Bta09362	AA3_2	fragment N-term;	GMC oxidoreductase		
Bta09385	GT1	fragment N-term;	Glycosyltransferase Family 1 protein		
Bta09395	CBM57		Carbohydrate-Binding Module Family 57 protein		
Bta09500	GT31-GT7	(fragment) splicing problem;	Glycosyltransferase Family 31 / Glycosyltransferase Family 7 protein		
Bta09508	AA1		Multicopper oxidase		
Bta09533	GT7	fragment N-term;	Glycosyltransferase Family 7 protein		
Bta09615	GT1		Glycosyltransferase Family 1 protein		
Bta09633	GH13_17-GH13_17	(fragment) splicing problem;	Glycoside Hydrolase Family 13 protein		
Bta09634	GH13		Glycoside Hydrolase Family 13 protein		
Bta09671	CBM14		Carbohydrate-Binding Module Family 14 protein		
Bta09696	GH13_17		Glycoside Hydrolase Family 13 protein		
Bta09701	GT1		Glycosyltransferase Family 1 protein		
Bta09742	GH85		Glycoside Hydrolase Family 85 protein		
Bta09856	GH37		Glycoside Hydrolase Family 37 protein		
Bta09882	GT49		Glycosyltransferase Family 49 protein		
Bta09947	GT1		Glycosyltransferase Family 1 protein		
Bta09948	GT1		Glycosyltransferase Family 1 protein		
Bta10017	CBM14		Carbohydrate-Binding Module Family 14 protein		
Bta10019	CBM14		Carbohydrate-Binding Module Family 14 protein		
Bta10022	GH13_17		Glycoside Hydrolase Family 13 protein		
Bta10045	CE9	(fragment) splicing problem;	Carbohydrate Esterase Family 9 protein		
Bta10088	GH1		Glycoside Hydrolase Family 1 protein		
Bta10108	CBM14		Carbohydrate-Binding Module Family 14 protein		
Bta10133	GT24		Glycosyltransferase Family 24 protein		
Bta10152	GH13_17	fragment C-term;	Glycoside Hydrolase Family 13 protein		
Bta10275	GT39		Glycosyltransferase Family 39 protein		
Bta10294	GT57		Glycosyltransferase Family 57 protein		
Bta10320	GH18		Glycoside Hydrolase Family 18 protein		

Bta10399	GT7	(fragment) splicing problem;	Glycosyltransferase Family 7 protein		
Bta10525	GT31	(fragment) splicing problem;	Glycosyltransferase Family 31 protein		
Bta10551	GH35	fragment N-term;	Glycoside Hydrolase Family 35 protein		
Bta10552	GH35	fragment C-term;	Glycoside Hydrolase Family 35 protein		
Bta10614	GH13	(fragment) splicing problem;	Glycoside Hydrolase Family 13 protein		
Bta10620	GH18		Glycoside Hydrolase Family 18 protein		
Bta10635	GH22	fragment C-term;	Glycoside Hydrolase Family 22 protein		
Bta10665	GT43		Glycosyltransferase Family 43 protein		
Bta10684	CBM39		Carbohydrate-Binding Module Family 39 protein		
Bta10764	GT49		Glycosyltransferase Family 49 protein		
Bta10765	GT49	(fragment) splicing problem;	Glycosyltransferase Family 49 protein		
Bta10773	GT23		Glycosyltransferase Family 23 protein		
Bta10805	GH18-CBM14		Glycoside Hydrolase Family 18 / Carbohydrate-Binding Module Family 14 protein		
Bta10836	GH18-CBM14-CBM14-CBM14	fragment N-term;	Glycoside Hydrolase Family 18 / Carbohydrate-Binding Module Family 14 protein		
Bta10858	GT31	(fragment) splicing problem;	Glycosyltransferase Family 31 protein		
Bta10859	GT31	fragment N-term;	Glycosyltransferase Family 31 protein		
Bta10860	GT31-GT31-GT31	fragment C-term;	Glycosyltransferase Family 31 protein		
Bta10863	GT1		Glycosyltransferase Family 1 protein		
Bta10864	GT1	(fragment) splicing problem;	Glycosyltransferase Family 1 protein		
Bta10865	GT1		Glycosyltransferase Family 1 protein		
Bta10911	AA3_2		GMC oxidoreductase		
Bta10981	CBM14		Carbohydrate-Binding Module Family 14 protein		
Bta11035	AA3_2		GMC oxidoreductase		
Bta11045	GT8		Glycosyltransferase Family 8 protein		
Bta11069	GT1	fragment N-term;	Glycosyltransferase Family 1 protein		
Bta11080	GT22		Glycosyltransferase Family 22 protein		
Bta11100	GH22		Glycoside Hydrolase Family 22 protein		
Bta11101	GT1	fragment N-term;	Glycosyltransferase Family 1 protein		
Bta11108	GT1		Glycosyltransferase Family 1 protein		
Bta11109	GT1-GT1	(fragment) splicing problem;	Glycosyltransferase Family 1 protein		
Bta11221	CE8		Carbohydrate Esterase Family 8 protein	HGT	Viridiplantae
Bta11222	CE8	fragment C-term;	Carbohydrate Esterase Family 8 protein	HGT	Viridiplantae
Bta11257	GH1		Glycoside Hydrolase Family 1 protein		
Bta11358	GH13_17		Glycoside Hydrolase Family 13 protein		
Bta11426	GT66		Glycosyltransferase Family 66 protein		
Bta11448	GH13_15		Glycoside Hydrolase Family 13 protein		
Bta11582	AA3_2		GMC oxidoreductase		
Bta11629	AA3_2		GMC oxidoreductase		
Bta11760	GH13_25-GH133		Glycoside Hydrolase Family 13 / Glycoside Hydrolase Family 133 protein		
Bta11780	GH31		Glycoside Hydrolase Family 31 protein		
Bta11831	GH31		Glycoside Hydrolase Family 31 protein		
Bta11864	GT1	(fragment) splicing problem;	Glycosyltransferase Family 1 protein		
Bta11878	AA1		Multicopper oxidase		
Bta11896	AA1		Multicopper oxidase		
Bta11905	AA15		Auxilliary Activities Family 15 protein		
Bta11975	GH13_17	fragment N-term;	Glycoside Hydrolase Family 13 protein		
Bta11977	GH13_17		Glycoside Hydrolase Family 13 protein		
Bta11978	GH13_17	(fragment) splicing problem;	Glycoside Hydrolase Family 13 protein		
Bta11979	GH13_17		Glycoside Hydrolase Family 13 protein		

Bta12019	GT2		Glycosyltransferase Family 2 protein		
Bta12077	GT1		Glycosyltransferase Family 1 protein		
Bta12101	GH13_17		Glycoside Hydrolase Family 13 protein		
Bta12131	GH20	fragment C-term;	Glycoside Hydrolase Family 20 protein		
Bta12153	CBM14		Carbohydrate-Binding Module Family 14 protein		
Bta12213	GT33		Glycosyltransferase Family 33 protein		
Bta12269	GT1		Glycosyltransferase Family 1 protein		
Bta12369	GT58	(fragment) splicing problem;	Glycosyltransferase Family 58 protein		
Bta12376	GT21		Glycosyltransferase Family 21 protein		
Bta12433	GT27-CBM13		Glycosyltransferase Family 27 / Carbohydrate-Binding Module Family 13 protein		
Bta12440	GT1		Glycosyltransferase Family 1 protein		
Bta12453	CBM32	fragment C-term;	Carbohydrate-Binding Module Family 32 protein	HGT	Fungi
Bta12456	CBM14-CBM14-CBM14-CBM14-CBM14-CBM14-CBM14		Carbohydrate-Binding Module Family 14 protein		
Bta12575	GT31		Glycosyltransferase Family 31 protein		
Bta12578	GH13_17		Glycoside Hydrolase Family 13 protein		
Bta12583	GT32	fragment C-term;	Glycosyltransferase Family 32 protein		
Bta12585	GT32		Glycosyltransferase Family 32 protein		
Bta12611	AA3_2		GMC oxidoreductase		
Bta12617	GH31		Glycoside Hydrolase Family 31 protein		
Bta12624	GH13_17	(fragment) splicing problem;	Glycoside Hydrolase Family 13 protein		
Bta12670	GH13_17		Glycoside Hydrolase Family 13 protein		
Bta12671	GH13_17		Glycoside Hydrolase Family 13 protein		
Bta12678	GH13_17		Glycoside Hydrolase Family 13 protein		
Bta12680	GH31		Glycoside Hydrolase Family 31 protein		
Bta12682	GH13_17		Glycoside Hydrolase Family 13 protein		
Bta12683	GH13_17		Glycoside Hydrolase Family 13 protein		
Bta12684	GT1	fragment C-term;	Glycosyltransferase Family 1 protein		
Bta12685	GT1	fragment N-term / C-term;	Glycosyltransferase Family 1 protein		
Bta12702	GT14		Glycosyltransferase Family 14 protein		
Bta12717	GT49		Glycosyltransferase Family 49 protein		
Bta12718	GH27		Glycoside Hydrolase Family 27 protein		
Bta12836	AA3_2		GMC oxidoreductase		
Bta12860	GH37		Glycoside Hydrolase Family 37 protein		
Bta12862	GT92		Glycosyltransferase Family 92 protein		
Bta12867	GH29	(fragment) splicing problem;	Glycoside Hydrolase Family 29 protein		
Bta12927	GT10	(fragment) splicing problem;	Glycosyltransferase Family 10 protein		
Bta13236	GH13_17		Glycoside Hydrolase Family 13 protein		
Bta13237	GH13_15	fragment N-term;	Glycoside Hydrolase Family 13 protein		
Bta13238	GH13_17	fragment C-term;	Glycoside Hydrolase Family 13 protein		
Bta13239	GH13_17	fragment N-term;	Glycoside Hydrolase Family 13 protein		
Bta13432	GT2		Glycosyltransferase Family 2 protein		
Bta13439	GH2	(fragment) splicing problem;	Glycoside Hydrolase Family 2 protein		
Bta13494	GT16		Glycosyltransferase Family 16 protein		
Bta13568	CBM48		Carbohydrate-Binding Module Family 48 protein		
Bta13611	GT7		Glycosyltransferase Family 7 protein		
Bta13629	GT105	(fragment) splicing problem;	Glycosyltransferase Family 105 protein		
Bta13631	GT90		Glycosyltransferase Family 90 protein		
Bta13660	GH13_17		Glycoside Hydrolase Family 13 protein		
Bta13669	GH18-CBM18-CBM18-CBM18		Glycoside Hydrolase Family 18 / Carbohydrate-Binding Module Family 18 protein		

Bta13679	GT3		Glycosyltransferase Family 3 protein		
Bta13694	CBM14		Carbohydrate-Binding Module Family 14 protein		
Bta13755	GT1	fragment C-term;	Glycosyltransferase Family 1 protein		
Bta13756	GT1		Glycosyltransferase Family 1 protein		
Bta13757	GT1	(fragment) splicing problem;	Glycosyltransferase Family 1 protein		
Bta13758	GT1	fragment N-term;	Glycosyltransferase Family 1 protein		
Bta13867	GT47-GT64		Glycosyltransferase Family 47 / Glycosyltransferase Family 64 protein		
Bta13871	CBM48-GH13_8		Carbohydrate-Binding Module Family 48 / Glycoside Hydrolase Family 13 protein		
Bta13913	GH13_17	fragment N-term / C-term;	Glycoside Hydrolase Family 13 protein		
Bta13914	GH13_17		Glycoside Hydrolase Family 13 protein		
Bta13961	GH152		Glycoside Hydrolase Family 152 protein	HGT	Viridiplantae
Bta13982	GH20	fragment N-term;	Glycoside Hydrolase Family 20 protein		
Bta14010	GT7	fragment N-term;	Glycosyltransferase Family 7 protein		
Bta14019	GT39		Glycosyltransferase Family 39 protein		
Bta14112	CBM14-CBM14		Carbohydrate-Binding Module Family 14 protein		
Bta14119	GT1	fragment C-term;	Glycosyltransferase Family 1 protein		
Bta14275	GT27-CBM13		Glycosyltransferase Family 27 / Carbohydrate-Binding Module Family 13 protein		
Bta14305	GT7		Glycosyltransferase Family 7 protein		
Bta14313	GH13	fragment C-term;	Glycoside Hydrolase Family 13 protein		
Bta14369	GH89	fragment N-term / C-term;	Glycoside Hydrolase Family 89 protein		
Bta14419	GH13_17		Glycoside Hydrolase Family 13 protein		
Bta14422	GH13_17		Glycoside Hydrolase Family 13 protein		
Bta14475	CBM14-CBM14	(fragment) splicing problem;	Carbohydrate-Binding Module Family 14 protein		
Bta14509	GH22-GH22		Glycoside Hydrolase Family 22 protein		
Bta14571	GT31	(fragment) splicing problem;	Glycosyltransferase Family 31 protein		
Bta14596	GH18-CBM14-CBM14-CBM14-GH18-GH18-CBM14-GH18		Glycoside Hydrolase Family 18 / Carbohydrate-Binding Module Family 14 protein		
Bta14598	GT29	(fragment) splicing problem;	Glycosyltransferase Family 29 protein		
Bta14689	GT66	(fragment) splicing problem;	Glycosyltransferase Family 66 protein		
Bta14723	GH20		Glycoside Hydrolase Family 20 protein		
Bta14774	GH20		Glycoside Hydrolase Family 20 protein		
Bta14797	GH56		Glycoside Hydrolase Family 56 protein		
Bta14885	GT61		Glycosyltransferase Family 61 protein	HGT	Viridiplantae
Bta15044	GT92		Glycosyltransferase Family 92 protein		
Bta15239	EXPN		Distantly related to plant expansins	HGT	Viridiplantae
Bta15255	GT1		Glycosyltransferase Family 1 protein		
Bta15284	AA1		Multicopper oxidase		
Bta15340	GH20	(fragment) splicing problem;	Glycoside Hydrolase Family 20 protein		
Bta15388	GT32		Glycosyltransferase Family 32 protein		
Bta15456	GH31	(fragment) splicing problem;	Glycoside Hydrolase Family 31 protein		
Bta15607	GH35	fragment C-term;	Glycoside Hydrolase Family 35 protein		
Bta15609	GH35	fragment N-term;	Glycoside Hydrolase Family 35 protein		
Bta15610	GH35		Glycoside Hydrolase Family 35 protein		
Bta15649	GH13_17		Glycoside Hydrolase Family 13 protein		
Bta15659	GT17		Glycosyltransferase Family 17 protein	HGT	Viridiplantae
Bta15703	GT20		Glycosyltransferase Family 20 protein		
Bta15714	CBM50		Carbohydrate-Binding Module Family 50 protein	HGT	Bacteria

T. vaporariorum

Protein ID	Description	Model notes	Definition line	HGT	Donor
TLow_00586-RA	GT2	fragment N-term;	Glycosyltransferase Family 2 protein		
TLow_00611-RA	GT7		Glycosyltransferase Family 7 protein		
TLow_00703-RA	CBM14		Carbohydrate-Binding Module Family 14 protein		
TLow_01318-RA	AA3_2	fragment N-term / C-term;	GMC oxidoreductase		
TLow_01399-RA	GH79	fragment C-term;	Glycoside Hydrolase Family 79 protein		
TLow_01400-RA	GH79	fragment C-term;	Glycoside Hydrolase Family 79 protein		
TLow_01636-RA	GH13	fragment N-term / C-term;	Glycoside Hydrolase Family 13 protein		
TLow_01771-RA	GT61	fragment N-term;	Glycosyltransferase Family 61 protein		
Tv_00105-RA	CBM14		Carbohydrate-Binding Module Family 14 protein		
Tv_00151-RA	AA3_2		GMC oxidoreductase		
Tv_00286-RA	GT1		Glycosyltransferase Family 1 protein		
Tv_00298-RA	GT1		Glycosyltransferase Family 1 protein		
Tv_00316-RA	GT27-CBM13		Glycosyltransferase Family 27 / Carbohydrate-Binding Module Family 13 protein		
Tv_00359-RA	GT27-CBM13	fragment N-term;	Glycosyltransferase Family 27 / Carbohydrate-Binding Module Family 13 protein		
Tv_00385-RA	GT2		Glycosyltransferase Family 2 protein		
Tv_00408-RA	GT2	fragment C-term;	Glycosyltransferase Family 2 protein		
Tv_00469-RA	GT1		Glycosyltransferase Family 1 protein		
Tv_00477-RA	GT31	(fragment) splicing problem;	Glycosyltransferase Family 31 protein		
Tv_00541-RA	GH22		Glycoside Hydrolase Family 22 protein		
Tv_00627-RA	GT1		Glycosyltransferase Family 1 protein		
Tv_00746-RA	GT105	fragment N-term / C-term;	Glycosyltransferase Family 105 protein		
Tv_00748-RA	GT105	fragment N-term;	Glycosyltransferase Family 105 protein		
Tv_00792-RA	CBM14		Carbohydrate-Binding Module Family 14 protein		
Tv_00841-RA	GH20		Glycoside Hydrolase Family 20 protein		
Tv_00902-RA	GT1	fragment C-term;	Glycosyltransferase Family 1 protein		
Tv_00903-RA	GT1-GT1-GT1	fragment N-term;	Glycosyltransferase Family 1 protein		
Tv_00904-RA	GT1-GT1		Glycosyltransferase Family 1 protein		
Tv_00906-RA	GT1	fragment N-term;	Glycosyltransferase Family 1 protein		
Tv_00938-RA	GH13_17		Glycoside Hydrolase Family 13 protein		
Tv_00942-RA	GT7		Glycosyltransferase Family 7 protein		
Tv_00954-RA	GH37		Glycoside Hydrolase Family 37 protein		
Tv_00966-RA	AA3_2		GMC oxidoreductase		
Tv_01011-RA	GT8	fragment C-term;	Glycosyltransferase Family 8 protein		
Tv_01013-RA	GT8	fragment C-term;	Glycosyltransferase Family 8 protein		
Tv_01066-RA	GH31		Glycoside Hydrolase Family 31 protein		
Tv_01108-RA	GH18-CBM14-CBM14-CBM14-GH18-GH18-CBM14		Glycoside Hydrolase Family 18 / Carbohydrate-Binding Module Family 14 protein		
Tv_01131-RA	CBM14-CBM14-CBM14		Carbohydrate-Binding Module Family 14 protein		
Tv_01133-RA	CBM14-CBM14-CBM14		Carbohydrate-Binding Module Family 14 protein		
Tv_01134-RA	CBM14-CBM14-CBM14		Carbohydrate-Binding Module Family 14 protein		
Tv_01135-RA	CBM14-CBM14-CBM14		Carbohydrate-Binding Module Family 14 protein		
Tv_01140-RA	CBM14		Carbohydrate-Binding Module Family 14 protein		
Tv_01194-RA	GT27	fragment C-term;	Glycosyltransferase Family 27 protein		
Tv_01195-RA	CBM13		Carbohydrate-Binding Module Family 13 protein		
Tv_01250-RA	CE9	(fragment) splicing problem;	Carbohydrate Esterase Family 9 protein		
Tv_01347-RA	GT1		Glycosyltransferase Family 1 protein		
Tv_01461-RA	CBM47	(fragment) splicing problem;	Carbohydrate-Binding Module Family 47 protein		

Tv_01486-RA	GT29		Glycosyltransferase Family 29 protein		
Tv_01510-RA	GH84		Glycoside Hydrolase Family 84 protein		
Tv_01518-RA	GH37	fragment N-term;	Glycoside Hydrolase Family 37 protein		
Tv_01563-RA	GT22		Glycosyltransferase Family 22 protein		
Tv_01595-RA	GT31	(fragment) splicing problem;	Glycosyltransferase Family 31 protein		
Tv_01864-RA	GH27		Glycoside Hydrolase Family 27 protein		
Tv_01872-RA	GH20		Glycoside Hydrolase Family 20 protein		
Tv_01873-RA	GH20		Glycoside Hydrolase Family 20 protein		
Tv_02025-RA	CBM14-CBM14	(fragment) splicing problem;	Carbohydrate-Binding Module Family 14 protein		
Tv_02051-RA	GT1	(fragment) splicing problem;	Glycosyltransferase Family 1 protein		
Tv_02090-RA	GT1		Glycosyltransferase Family 1 protein		
Tv_02098-RA	CBM13		Carbohydrate-Binding Module Family 13 protein		
Tv_02099-RA	CBM13	(fragment) splicing problem;	Carbohydrate-Binding Module Family 13 protein		
Tv_02104-RA	GT27	fragment N-term / C-term;	Glycosyltransferase Family 27 protein		
Tv_02109-RA	GH47	fragment N-term;	Glycoside Hydrolase Family 47 protein		
Tv_02146-RA	CBM14	fragment N-term;	Carbohydrate-Binding Module Family 14 protein		
Tv_02147-RA	CBM14	fragment C-term;	Carbohydrate-Binding Module Family 14 protein		
Tv_02163-RA	GT105		Glycosyltransferase Family 105 protein		
Tv_02254-RA	GT4		Glycosyltransferase Family 4 protein		
Tv_02275-RA	GT20	fragment C-term;	Glycosyltransferase Family 20 protein		
Tv_02277-RA	GT1	fragment N-term;	Glycosyltransferase Family 1 protein		
Tv_02342-RA	GH13		Glycoside Hydrolase Family 13 protein		
Tv_02343-RA	GH13		Glycoside Hydrolase Family 13 protein		
Tv_02344-RA	GH13		Glycoside Hydrolase Family 13 protein		
Tv_02358-RA	GT2		Glycosyltransferase Family 2 protein		
Tv_02425-RA	CBM14		Carbohydrate-Binding Module Family 14 protein		
Tv_02426-RA	CBM14		Carbohydrate-Binding Module Family 14 protein		
Tv_02441-RA	GT35		Glycosyltransferase Family 35 protein		
Tv_02581-RA	GT4		Glycosyltransferase Family 4 protein		
Tv_02586-RA	GT7		Glycosyltransferase Family 7 protein		
Tv_03404-RA	GT8-GT49	(fragment) splicing problem;	Glycosyltransferase Family 8 / Glycosyltransferase Family 49 protein		
Tv_03505-RA	GH27		Glycoside Hydrolase Family 27 protein		
Tv_03530-RA	GH38		Glycoside Hydrolase Family 38 protein		
Tv_03544-RA	GT20	fragment N-term;	Glycosyltransferase Family 20 protein		
Tv_03582-RA	GT27-CBM13		Glycosyltransferase Family 27 / Carbohydrate-Binding Module Family 13 protein		
Tv_03624-RA	GT22	fragment C-term;	Glycosyltransferase Family 22 protein		
Tv_03625-RA	GT22	(fragment) splicing problem;	Glycosyltransferase Family 22 protein		
Tv_03746-RA	GH13_17		Glycoside Hydrolase Family 13 protein		
Tv_03782-RA	GT43	fragment C-term;	Glycosyltransferase Family 43 protein		
Tv_03807-RA	GT47-GT64	fragment N-term;	Glycosyltransferase Family 47 / Glycosyltransferase Family 64 protein		
Tv_03973-RA	GT27-CBM13	(fragment) splicing problem;	Glycosyltransferase Family 27 / Carbohydrate-Binding Module Family 13 protein		
Tv_03990-RA	GT23		Glycosyltransferase Family 23 protein		
Tv_04069-RA	GH13_17		Glycoside Hydrolase Family 13 protein		
Tv_04071-RA	GH13_17		Glycoside Hydrolase Family 13 protein		
Tv_04072-RA	GH13_17		Glycoside Hydrolase Family 13 protein		
Tv_04073-RA	GH13_17-GH13_17		Glycoside Hydrolase Family 13 protein		
Tv_04075-RA	GH13_17		Glycoside Hydrolase Family 13 protein		
Tv_04077-RA	GH13_17		Glycoside Hydrolase Family 13 protein		
Tv_04078-RA	GH13_17		Glycoside Hydrolase Family 13 protein		

Tv_04079-RA	GH13	fragment C-term;	Glycoside Hydrolase Family 13 protein	HGT	Bacteria
Tv_04113-RA	GT1		Glycosyltransferase Family 1 protein		
Tv_04122-RA	GH13_17		Glycoside Hydrolase Family 13 protein		
Tv_04124-RA	GH13_17	fragment N-term / C-term;	Glycoside Hydrolase Family 13 protein		
Tv_04131-RA	GH13_17	(fragment) splicing problem;	Glycoside Hydrolase Family 13 protein		
Tv_04132-RA	GH13_17		Glycoside Hydrolase Family 13 protein		
Tv_04133-RA	GH13_17		Glycoside Hydrolase Family 13 protein		
Tv_04134-RA	GH13_17		Glycoside Hydrolase Family 13 protein		
Tv_04172-RA	GT49		Glycosyltransferase Family 49 protein		
Tv_04197-RA	GH18		Glycoside Hydrolase Family 18 protein		
Tv_04246-RA	GT1-GT1		Glycosyltransferase Family 1 protein		
Tv_04295-RA	GH18-CBM14		Glycoside Hydrolase Family 18 / Carbohydrate-Binding Module Family 14 protein		
Tv_04305-RA	GT24	(fragment) splicing problem;	Glycosyltransferase Family 24 protein		
Tv_04489-RA	GH13_17	(fragment) splicing problem;	Glycoside Hydrolase Family 13 protein		
Tv_04500-RA	GH38		Glycoside Hydrolase Family 38 protein		
Tv_04564-RA	GT4		Glycosyltransferase Family 4 protein		
Tv_04576-RA	GH13_17	(fragment) splicing problem;	Glycoside Hydrolase Family 13 protein		
Tv_04643-RA	GT61		Glycosyltransferase Family 61 protein		
Tv_04727-RA	GT1		Glycosyltransferase Family 1 protein		
Tv_04751-RA	GH13_17	(fragment) splicing problem;	Glycoside Hydrolase Family 13 protein		
Tv_04752-RA	GH13_17	(fragment) splicing problem;	Glycoside Hydrolase Family 13 protein		
Tv_04852-RA	CBM14		Carbohydrate-Binding Module Family 14 protein		
Tv_04857-RA	GT31		Glycosyltransferase Family 31 protein		
Tv_04859-RA	GT1-GT1	fragment N-term;	Glycosyltransferase Family 1 protein		
Tv_04891-RA	GT65	(fragment) splicing problem;	Glycosyltransferase Family 65 protein		
Tv_04916-RA	GT1	fragment N-term;	Glycosyltransferase Family 1 protein		
Tv_04931-RA	GT1		Glycosyltransferase Family 1 protein		
Tv_04932-RA	GT1		Glycosyltransferase Family 1 protein		
Tv_04934-RA	GT27-CBM13	(fragment) splicing problem;	Glycosyltransferase Family 27 / Carbohydrate-Binding Module Family 13 protein		
Tv_04957-RA	GT20		Glycosyltransferase Family 20 protein		
Tv_04960-RA	GH13_17	fragment C-term;	Glycoside Hydrolase Family 13 protein		
Tv_04997-RA	GH13_17	fragment N-term;	Glycoside Hydrolase Family 13 protein		
Tv_04998-RA	GH13	fragment C-term;	Glycoside Hydrolase Family 13 protein		
Tv_04999-RA	GH13_17	(fragment) splicing problem;	Glycoside Hydrolase Family 13 protein		
Tv_05000-RA	GH13_17-GH13	fragment C-term;	Glycoside Hydrolase Family 13 protein		
Tv_05001-RA	GH13_17	(fragment) splicing problem;	Glycoside Hydrolase Family 13 protein		
Tv_05069-RA	GT49	(fragment) splicing problem;	Glycosyltransferase Family 49 protein		
Tv_05077-RA	GT21	fragment N-term;	Glycosyltransferase Family 21 protein		
Tv_05083-RA	GT1		Glycosyltransferase Family 1 protein		
Tv_05090-RA	GT58		Glycosyltransferase Family 58 protein		
Tv_05105-RA	PL1_4	fragment C-term;	Polysaccharide Lyase Family 1 protein	HGT	Complex: Bacteria or Fungi
Tv_05119-RA	GT1		Glycosyltransferase Family 1 protein		
Tv_05121-RA	GT1		Glycosyltransferase Family 1 protein		
Tv_05122-RA	GT1		Glycosyltransferase Family 1 protein		
Tv_05145-RA	GT1		Glycosyltransferase Family 1 protein		
Tv_05165-RA	AA3_2		GMC oxidoreductase		
Tv_05314-RA	GT49		Glycosyltransferase Family 49 protein		
Tv_05317-RA	GH27		Glycoside Hydrolase Family 27 protein		
Tv_05323-RA	GT90		Glycosyltransferase Family 90 protein		

Tv_05391-RA	GT1		Glycosyltransferase Family 1 protein		
Tv_05430-RA	GT1	fragment C-term;	Glycosyltransferase Family 1 protein		
Tv_05441-RA	GT1	(fragment) splicing problem;	Glycosyltransferase Family 1 protein		
Tv_05533-RA	GT54	(fragment) splicing problem;	Glycosyltransferase Family 54 protein		
Tv_05535-RA	GT54	fragment C-term;	Glycosyltransferase Family 54 protein		
Tv_05541-RA	GT25	fragment C-term;	Glycosyltransferase Family 25 protein		
Tv_05548-RA	GT25	fragment N-term;	Glycosyltransferase Family 25 protein		
Tv_05549-RA	GT54		Glycosyltransferase Family 54 protein		
Tv_05587-RA	GT66		Glycosyltransferase Family 66 protein		
Tv_05613-RA	AA3_2		GMC oxidoreductase		
Tv_05624-RA	AA3_2		GMC oxidoreductase		
Tv_05656-RA	GH20		Glycoside Hydrolase Family 20 protein		
Tv_05701-RA	CBM47		Carbohydrate-Binding Module Family 47 protein		
Tv_05908-RA	AA3_2		GMC oxidoreductase		
Tv_05909-RA	GH13_17		Glycoside Hydrolase Family 13 protein		
Tv_05910-RA	GH13_17		Glycoside Hydrolase Family 13 protein		
Tv_05917-RA	GT1-GT1		Glycosyltransferase Family 1 protein		
Tv_05937-RA	GH13	(fragment) splicing problem;	Glycoside Hydrolase Family 13 protein		
Tv_05979-RA	GT1	fragment N-term;	Glycosyltransferase Family 1 protein		
Tv_05987-RA	GT1	fragment N-term;	Glycosyltransferase Family 1 protein		
Tv_06020-RA	GH89	fragment C-term;	Glycoside Hydrolase Family 89 protein		
Tv_06021-RA	GH89	fragment N-term;	Glycoside Hydrolase Family 89 protein		
Tv_06050-RA	GH13_17	(fragment) splicing problem;	Glycoside Hydrolase Family 13 protein		
Tv_06132-RA	GT10	fragment N-term;	Glycosyltransferase Family 10 protein		
Tv_06277-RA	GH20		Glycoside Hydrolase Family 20 protein		
Tv_06371-RA	GT10		Glycosyltransferase Family 10 protein		
Tv_06393-RA	GT1		Glycosyltransferase Family 1 protein		
Tv_06471-RA	CBM14		Carbohydrate-Binding Module Family 14 protein		
Tv_06484-RA	GH18-CBM14-CBM14		Glycoside Hydrolase Family 18 / Carbohydrate-Binding Module Family 14 protein		
Tv_06504-RA	GT66	fragment C-term;	Glycosyltransferase Family 66 protein		
Tv_06589-RA	CBM50		Carbohydrate-Binding Module Family 50 protein		
Tv_06597-RA	GH20		Glycoside Hydrolase Family 20 protein		
Tv_06654-RA	GT22		Glycosyltransferase Family 22 protein		
Tv_06726-RA	AA1		Multicopper oxidase		
Tv_06734-RA	GT31-GT7		Glycosyltransferase Family 31 / Glycosyltransferase Family 7 protein		
Tv_06794-RA	GT110		Glycosyltransferase Family 110 protein		
Tv_06971-RA	GT47-GT64		Glycosyltransferase Family 47 / Glycosyltransferase Family 64 protein		
Tv_07030-RA	CBM14		Carbohydrate-Binding Module Family 14 protein		
Tv_07082-RA	GH85		Glycoside Hydrolase Family 85 protein		
Tv_07120-RA	AA3_2		GMC oxidoreductase		
Tv_07170-RA	GT13		Glycosyltransferase Family 13 protein		
Tv_07242-RA	GT8	(fragment) splicing problem;	Glycosyltransferase Family 8 protein		
Tv_07372-RA	GH2		Glycoside Hydrolase Family 2 protein		
Tv_07391-RA	GT92		Glycosyltransferase Family 92 protein		
Tv_07457-RA	AA3_2		GMC oxidoreductase		
Tv_07510-RA	GH47		Glycoside Hydrolase Family 47 protein		
Tv_07550-RA	GT31	(fragment) splicing problem;	Glycosyltransferase Family 31 protein		
Tv_07557-RA	AA1		Multicopper oxidase		
Tv_07565-RA	GT31		Glycosyltransferase Family 31 protein		

Tv_07716-RA	GT41		Glycosyltransferase Family 41 protein		
Tv_07809-RA	GH18	(fragment) splicing problem;	Glycoside Hydrolase Family 18 protein		
Tv_07810-RA	GH18		Glycoside Hydrolase Family 18 protein		
Tv_07905-RA	GH13_17		Glycoside Hydrolase Family 13 protein		
Tv_07947-RA	GT39		Glycosyltransferase Family 39 protein		
Tv_08013-RA	GH56		Glycoside Hydrolase Family 56 protein		
Tv_08063-RA	GT3		Glycosyltransferase Family 3 protein		
Tv_08077-RA	CBM14-CBM14-CBM14-CBM14-CBM14-CBM14-CBM14		Carbohydrate-Binding Module Family 14 protein		
Tv_08081-RA	GT43		Glycosyltransferase Family 43 protein		
Tv_08177-RA	GH31		Glycoside Hydrolase Family 31 protein		
Tv_08210-RA	GH20		Glycoside Hydrolase Family 20 protein		
Tv_08263-RA	CBM14		Carbohydrate-Binding Module Family 14 protein		
Tv_08317-RA	GH30_1		Glycoside Hydrolase Family 30 protein		
Tv_08448-RA	GH47	(fragment) splicing problem;	Glycoside Hydrolase Family 47 protein		
Tv_08655-RA	GT31	fragment C-term;	Glycosyltransferase Family 31 protein		
Tv_08671-RA	GT1		Glycosyltransferase Family 1 protein		
Tv_08676-RA	GH13-GH13-GH13		Glycoside Hydrolase Family 13 protein		
Tv_08678-RA	GH13_17		Glycoside Hydrolase Family 13 protein		
Tv_08680-RA	GH13_17		Glycoside Hydrolase Family 13 protein		
Tv_08691-RA	GH116		Glycoside Hydrolase Family 116 protein		
Tv_08765-RA	GT1		Glycosyltransferase Family 1 protein		
Tv_08960-RA	GT59	fragment C-term;	Glycosyltransferase Family 59 protein		
Tv_08968-RA	CBM14		Carbohydrate-Binding Module Family 14 protein		
Tv_08969-RA	CBM14		Carbohydrate-Binding Module Family 14 protein		
Tv_08979-RA	GH13_17		Glycoside Hydrolase Family 13 protein		
Tv_08981-RA	GH13_17		Glycoside Hydrolase Family 13 protein		
Tv_09051-RA	GT31		Glycosyltransferase Family 31 protein		
Tv_09054-RA	GH18		Glycoside Hydrolase Family 18 protein		
Tv_09074-RA	GT105		Glycosyltransferase Family 105 protein		
Tv_09101-RA	CBM39		Carbohydrate-Binding Module Family 39 protein		
Tv_09204-RA	GH22		Glycoside Hydrolase Family 22 protein		
Tv_09245-RA	GT57		Glycosyltransferase Family 57 protein		
Tv_09445-RA	GT66	(fragment) splicing problem;	Glycosyltransferase Family 66 protein		
Tv_09527-RA	GT39		Glycosyltransferase Family 39 protein		
Tv_09545-RA	GT31		Glycosyltransferase Family 31 protein		
Tv_09550-RA	GT31	fragment N-term;	Glycosyltransferase Family 31 protein		
Tv_09566-RA	GH1	(fragment) splicing problem;	Glycoside Hydrolase Family 1 protein		
Tv_09568-RA	GH22		Glycoside Hydrolase Family 22 protein		
Tv_09581-RA	GH13_17		Glycoside Hydrolase Family 13 protein		
Tv_09582-RA	GH13_17	fragment C-term;	Glycoside Hydrolase Family 13 protein		
Tv_09589-RA	GH13-GH13	(fragment) splicing problem;	Glycoside Hydrolase Family 13 protein		
Tv_09675-RA	GH13_17		Glycoside Hydrolase Family 13 protein		
Tv_09676-RA	GH13_17		Glycoside Hydrolase Family 13 protein		
Tv_09677-RA	GH13_17		Glycoside Hydrolase Family 13 protein		
Tv_09686-RA	GH13_17		Glycoside Hydrolase Family 13 protein		
Tv_09694-RA	GH39		Glycoside Hydrolase Family 39 protein		
Tv_09747-RA	GH38	fragment C-term;	Glycoside Hydrolase Family 38 protein		
Tv_09748-RA	GH38	fragment N-term;	Glycoside Hydrolase Family 38 protein		
Tv_09765-RA	GH13	(fragment) splicing problem;	Glycoside Hydrolase Family 13 protein		

Tv_09794-RA	GH37	fragment C-term;	Glycoside Hydrolase Family 37 protein		
Tv_09799-RA	GH37-GH37	fragment N-term / C-term;	Glycoside Hydrolase Family 37 protein		
Tv_09800-RA	GH37	fragment C-term;	Glycoside Hydrolase Family 37 protein		
Tv_09802-RA	GH37	fragment N-term;	Glycoside Hydrolase Family 37 protein		
Tv_09868-RA	GT105	fragment N-term;	Glycosyltransferase Family 105 protein		
Tv_09920-RA	GT1	fragment N-term;	Glycosyltransferase Family 1 protein		
Tv_10002-RA	GH13_17	(fragment) splicing problem;	Glycoside Hydrolase Family 13 protein		
Tv_10139-RA	GT105	fragment N-term / C-term;	Glycosyltransferase Family 105 protein		
Tv_10141-RA	GT105	fragment N-term;	Glycosyltransferase Family 105 protein		
Tv_10332-RA	GH31		Glycoside Hydrolase Family 31 protein		
Tv_10623-RA	GH31	fragment C-term;	Glycoside Hydrolase Family 31 protein		
Tv_10709-RA	GT1-GT1-GT1		Glycosyltransferase Family 1 protein		
Tv_10792-RA	AA3_2	fragment C-term;	GMC oxidoreductase		
Tv_10793-RA	AA3	fragment N-term;	GMC oxidoreductase		
Tv_10808-RA	GT1		Glycosyltransferase Family 1 protein		
Tv_10938-RA	GT31-GT7	fragment C-term;	Glycosyltransferase Family 31 / Glycosyltransferase Family 7 protein		
Tv_11122-RA	GH13_17	fragment N-term / C-term;	Glycoside Hydrolase Family 13 protein		
Tv_11123-RA	GH13	fragment C-term;	Glycoside Hydrolase Family 13 protein		
Tv_11124-RA	GH13_17		Glycoside Hydrolase Family 13 protein		
Tv_11131-RA	CBM21		Carbohydrate-Binding Module Family 21 protein		
Tv_11211-RA	GH13_25-GH133	(fragment) splicing problem;	Glycoside Hydrolase Family 13 / Glycoside Hydrolase Family 133 protein		
Tv_11223-RA	GH38	fragment N-term;	Glycoside Hydrolase Family 38 protein		
Tv_11224-RA	GH38	fragment N-term / C-term;	Glycoside Hydrolase Family 38 protein		
Tv_11262-RA	CBM57		Carbohydrate-Binding Module Family 57 protein		
Tv_11360-RA	AA15		Auxilliary Activities Family 15 protein		
Tv_11375-RA	AA1		Multicopper oxidase		
Tv_11376-RA	AA1		Multicopper oxidase		
Tv_11629-RA	GT13		Glycosyltransferase Family 13 protein		
Tv_11649-RA	GT27-CBM13		Glycosyltransferase Family 27 / Carbohydrate-Binding Module Family 13 protein		
Tv_11809-RA	GH18		Glycoside Hydrolase Family 18 protein		
Tv_11816-RA	CBM48-GH13_8		Carbohydrate-Binding Module Family 48 / Glycoside Hydrolase Family 13 protein		
Tv_11876-RA	GH31		Glycoside Hydrolase Family 31 protein		
Tv_11940-RA	GT1		Glycosyltransferase Family 1 protein		
Tv_12008-RA	GT47-GT64		Glycosyltransferase Family 47 / Glycosyltransferase Family 64 protein		
Tv_12259-RA	GT1		Glycosyltransferase Family 1 protein		
Tv_12473-RA	GT31	(fragment) splicing problem;	Glycosyltransferase Family 31 protein		
Tv_12479-RA	GT2		Glycosyltransferase Family 2 protein		
Tv_12497-RA	GT1		Glycosyltransferase Family 1 protein		
Tv_12648-RA	AA3	fragment N-term;	GMC oxidoreductase		
Tv_12650-RA	AA3_2		GMC oxidoreductase		
Tv_12651-RA	AA3_2		GMC oxidoreductase		
Tv_12652-RA	AA3_2	fragment N-term;	GMC oxidoreductase		
Tv_12653-RA	AA3_2	fragment C-term;	GMC oxidoreductase		
Tv_12655-RA	AA3_2	fragment N-term;	GMC oxidoreductase		
Tv_12660-RA	AA3_2	fragment C-term;	GMC oxidoreductase		
Tv_12661-RA	AA3_2		GMC oxidoreductase		
Tv_12662-RA	AA3_2		GMC oxidoreductase		
Tv_12663-RA	AA3_2	fragment N-term;	GMC oxidoreductase		
Tv_12664-RA	AA3_2	fragment C-term;	GMC oxidoreductase		

Tv_12665-RA	AA3_2		GMC oxidoreductase		
Tv_12666-RA	AA3_2		GMC oxidoreductase		
Tv_12766-RA	GT22		Glycosyltransferase Family 22 protein		
Tv_12853-RA	CBM14		Carbohydrate-Binding Module Family 14 protein		
Tv_12865-RA	GT1		Glycosyltransferase Family 1 protein		
Tv_12868-RA	GT1	fragment N-term;	Glycosyltransferase Family 1 protein		
Tv_12880-RA	GH1-GH1	(fragment) splicing problem;	Glycoside Hydrolase Family 1 protein		
Tv_12890-RA	GH63		Glycoside Hydrolase Family 63 protein		
Tv_12935-RA	GT1		Glycosyltransferase Family 1 protein		
Tv_13034-RA	GT90		Glycosyltransferase Family 90 protein		
Tv_13120-RA	GH13_17		Glycoside Hydrolase Family 13 protein		
Tv_13161-RA	GT33		Glycosyltransferase Family 33 protein		
Tv_13175-RA	GH79	fragment N-term;	Glycoside Hydrolase Family 79 protein		
Tv_13281-RA	GT1-GT1	(fragment) splicing problem;	Glycosyltransferase Family 1 protein		
Tv_13379-RA	GH20	fragment C-term;	Glycoside Hydrolase Family 20 protein		
Tv_13434-RA	AA15		Auxilliary Activities Family 15 protein		
Tv_13463-RA	GT32		Glycosyltransferase Family 32 protein		
Tv_13587-RA	AA3_2	(fragment) splicing problem;	GMC oxidoreductase		
Tv_13711-RA	GH35		Glycoside Hydrolase Family 35 protein		
Tv_13737-RA	GH18-CBM18-CBM18-CBM18	fragment N-term;	Glycoside Hydrolase Family 18 / Carbohydrate-Binding Module Family 18 protein		
Tv_13767-RA	GH18-CBM14-CBM14		Glycoside Hydrolase Family 18 / Carbohydrate-Binding Module Family 14 protein		
Tv_13768-RA	AA3_2		GMC oxidoreductase		
Tv_13841-RA	GT8		Glycosyltransferase Family 8 protein		
Tv_13954-RA	GT1-GT1	fragment N-term;	Glycosyltransferase Family 1 protein		
Tv_14001-RA	CBM21		Carbohydrate-Binding Module Family 21 protein		
Tv_14029-RA	GH22		Glycoside Hydrolase Family 22 protein		
Tv_14032-RA	GT1		Glycosyltransferase Family 1 protein		
Tv_14034-RA	GT1	fragment N-term;	Glycosyltransferase Family 1 protein		
Tv_14056-RA	GH16_4		Glycoside Hydrolase Family 16 protein		
Tv_14057-RA	GH16_4		Glycoside Hydrolase Family 16 protein		
Tv_14093-RA	GT14		Glycosyltransferase Family 14 protein		
Tv_14174-RA	AA1	fragment N-term;	Multicopper oxidase		
Tv_14176-RA	GT16		Glycosyltransferase Family 16 protein		
Tv_14208-RA	GH37	fragment N-term;	Glycoside Hydrolase Family 37 protein		
Tv_14213-RA	GH37		Glycoside Hydrolase Family 37 protein		
Tv_14255-RA	GT105	fragment N-term;	Glycosyltransferase Family 105 protein		
Tv_14305-RA	CBM14		Carbohydrate-Binding Module Family 14 protein		
Tv_14306-RA	GH37	fragment N-term;	Glycoside Hydrolase Family 37 protein		
Tv_14307-RA	GH37	fragment C-term;	Glycoside Hydrolase Family 37 protein		
Tv_14448-RA	GT1	fragment C-term;	Glycosyltransferase Family 1 protein		
Tv_14482-RA	CBM48		Carbohydrate-Binding Module Family 48 protein		
Tv_14568-RA	GT1	fragment N-term;	Glycosyltransferase Family 1 protein		
Tv_14587-RA	GT1		Glycosyltransferase Family 1 protein		
Tv_14760-RA	GH35	fragment N-term;	Glycoside Hydrolase Family 35 protein		
Tv_14790-RA	GT1		Glycosyltransferase Family 1 protein		
Tv_14792-RA	GT1	fragment N-term;	Glycosyltransferase Family 1 protein		
Tv_14824-RA	GH13_17		Glycoside Hydrolase Family 13 protein		
Tv_14833-RA	GT1		Glycosyltransferase Family 1 protein		
Tv_14944-RA	GH37		Glycoside Hydrolase Family 37 protein		

Tv_15005-RA	GH47		Glycoside Hydrolase Family 47 protein		
Tv_15020-RA	GT1	fragment C-term;	Glycosyltransferase Family 1 protein		
Tv_15045-RA	GT31		Glycosyltransferase Family 31 protein		
Tv_15081-RA	GT54	fragment C-term;	Glycosyltransferase Family 54 protein		
Tv_15088-RA	GT54	fragment N-term;	Glycosyltransferase Family 54 protein		
Tv_15133-RA	GT92		Glycosyltransferase Family 92 protein		
Tv_15139-RA	GH29		Glycoside Hydrolase Family 29 protein		
Tv_15167-RA	GT1		Glycosyltransferase Family 1 protein		
Tv_15503-RA	GT49		Glycosyltransferase Family 49 protein		
Tv_15512-RA	GT4		Glycosyltransferase Family 4 protein		
Tv_15739-RA	GH13_17	(fragment) splicing problem;	Glycoside Hydrolase Family 13 protein		
Tv_15755-RA	GH47		Glycoside Hydrolase Family 47 protein		
Tv_15844-RA	GH13	fragment C-term;	Glycoside Hydrolase Family 13 protein		
Tv_15845-RA	GH13_17	fragment N-term;	Glycoside Hydrolase Family 13 protein		
Tv_15891-RA	CBM14-CBM14-CBM14		Carbohydrate-Binding Module Family 14 protein		
Tv_15898-RA	GH99		Glycoside Hydrolase Family 99 protein		
Tv_15925-RA	CBM14		Carbohydrate-Binding Module Family 14 protein		
Tv_15928-RA	GH152	(fragment) splicing problem;	Glycoside Hydrolase Family 152 protein	HGT	Viridiplantae
Tv_15933-RA	CBM14		Carbohydrate-Binding Module Family 14 protein		
Tv_15955-RA	GH18	fragment C-term;	Glycoside Hydrolase Family 18 protein		
Tv_15956-RA	GH18-GH18	fragment N-term / C-term;	Glycoside Hydrolase Family 18 protein		
Tv_15957-RA	GH18-CBM14	fragment N-term;	Glycoside Hydrolase Family 18 / Carbohydrate-Binding Module Family 14 protein		
Tv_16156-RA	GT47	fragment C-term;	Glycosyltransferase Family 47 protein		

F. occidentalis

**Protein ID**	**Description**	**Model notes**	**Definition line**	**HGT**	**Donor**
XP_026271392.1	GT1		Glycosyltransferase Family 1 protein		
XP_026271551.1	AA3_2		GMC oxidoreductase		
XP_026271901.1	GH38	fragment C-term;	Glycoside Hydrolase Family 38 protein		
XP_026272000.1	GT105		Glycosyltransferase Family 105 protein		
XP_026272052.1	GH16_4		Glycoside Hydrolase Family 16 protein		
XP_026272092.1	GT22		Glycosyltransferase Family 22 protein		
XP_026272095.1	CBM14		Carbohydrate-Binding Module Family 14 protein		
XP_026272203.1	GH1		Glycoside Hydrolase Family 1 protein		
XP_026272209.1	GT10		Glycosyltransferase Family 10 protein		
XP_026272388.1	GH1	fragment N-term / C-term;	Glycoside Hydrolase Family 1 protein		
XP_026272402.1	GH16_4		Glycoside Hydrolase Family 16 protein		
XP_026272427.1	GT58		Glycosyltransferase Family 58 protein		
XP_026272503.1	GT31		Glycosyltransferase Family 31 protein		
XP_026272515.1	GT7		Glycosyltransferase Family 7 protein		
XP_026272538.1	GH37		Glycoside Hydrolase Family 37 protein		
XP_026272610.1	GT31		Glycosyltransferase Family 31 protein		
XP_026272668.1	GH22		Glycoside Hydrolase Family 22 protein		
XP_026272669.1	CBM21		Carbohydrate-Binding Module Family 21 protein		
XP_026272679.1	GH22		Glycoside Hydrolase Family 22 protein		
XP_026272691.1	GT20		Glycosyltransferase Family 20 protein		
XP_026272815.1	GT105		Glycosyltransferase Family 105 protein		
XP_026272828.1	GH18-CBM14		Glycoside Hydrolase Family 18 / Carbohydrate-Binding Module Family 14 protein		

XP_026272949.1	GT39		Glycosyltransferase Family 39 protein		
XP_026272953.1	GT31-GT7	(fragment) splicing problem;	Glycosyltransferase Family 31 / Glycosyltransferase Family 7 protein		
XP_026272958.1	GT8-GT49		Glycosyltransferase Family 8 / Glycosyltransferase Family 49 protein		
XP_026273012.1	GT49		Glycosyltransferase Family 49 protein		
XP_026273055.1	GH22		Glycoside Hydrolase Family 22 protein		
XP_026273122.1	GH32		Glycoside Hydrolase Family 32 protein	HGT	Bacteria
XP_026273138.1	AA1	fragment C-term;	Multicopper oxidase		
XP_026273151.1	GH35		Glycoside Hydrolase Family 35 protein		
XP_026273242.1	GT2		Glycosyltransferase Family 2 protein		
XP_026273431.1	GT47-GT64		Glycosyltransferase Family 47 / Glycosyltransferase Family 64 protein		
XP_026273575.1	GT14		Glycosyltransferase Family 14 protein		
XP_026273591.1	GH13_17		Glycoside Hydrolase Family 13 protein		
XP_026273891.1	GH30_1		Glycoside Hydrolase Family 30 protein		
XP_026273905.1	GT4		Glycosyltransferase Family 4 protein		
XP_026273921.1	GH89		Glycoside Hydrolase Family 89 protein		
XP_026273946.1	CBM14-CBM14-GH1		Carbohydrate-Binding Module Family 14 / Glycoside Hydrolase Family 1 protein		
XP_026273952.1	GT54		Glycosyltransferase Family 54 protein		
XP_026273993.1	GT25	fragment C-term;	Glycosyltransferase Family 25 protein		
XP_026274115.1	PL1_4	fragment C-term;	Polysaccharide Lyase Family 1 protein	HGT	Complex: Bacteria or Fungi
XP_026274121.1	GH1		Glycoside Hydrolase Family 1 protein		
XP_026274148.1	GT25	fragment N-term;	Glycosyltransferase Family 25 protein		
XP_026274343.1	GT66	(fragment) splicing problem;	Glycosyltransferase Family 66 protein		
XP_026274619.1	CBM14		Carbohydrate-Binding Module Family 14 protein		
XP_026274622.1	CBM14		Carbohydrate-Binding Module Family 14 protein		
XP_026274677.1	CBM14		Carbohydrate-Binding Module Family 14 protein		
XP_026274686.1	CBM14		Carbohydrate-Binding Module Family 14 protein		
XP_026274851.1	CBM14		Carbohydrate-Binding Module Family 14 protein		
XP_026274864.1	GT43		Glycosyltransferase Family 43 protein		
XP_026274870.1	GT27-CBM13		Glycosyltransferase Family 27 / Carbohydrate-Binding Module Family 13 protein		
XP_026274904.1	GT1		Glycosyltransferase Family 1 protein		
XP_026274905.1	GT1		Glycosyltransferase Family 1 protein		
XP_026274906.1	GT1		Glycosyltransferase Family 1 protein		
XP_026274911.1	GT1		Glycosyltransferase Family 1 protein		
XP_026274916.1	GT1-GT1-GT1		Glycosyltransferase Family 1 protein		
XP_026275035.1	CBM14		Carbohydrate-Binding Module Family 14 protein		
XP_026275069.1	CBM14		Carbohydrate-Binding Module Family 14 protein		
XP_026275176.1	GT16		Glycosyltransferase Family 16 protein		
XP_026275221.1	GT1		Glycosyltransferase Family 1 protein		
XP_026275317.1	GH31		Glycoside Hydrolase Family 31 protein		
XP_026275448.1	CBM14		Carbohydrate-Binding Module Family 14 protein		
XP_026275456.1	GT22		Glycosyltransferase Family 22 protein		
XP_026275497.1	GT1		Glycosyltransferase Family 1 protein		
XP_026275702.1	GT43		Glycosyltransferase Family 43 protein		
XP_026275815.1	GT61		Glycosyltransferase Family 61 protein		
XP_026275888.1	AA3_2		GMC oxidoreductase		
XP_026276037.1	GH30_1		Glycoside Hydrolase Family 30 protein		
XP_026276202.1	GT1		Glycosyltransferase Family 1 protein		
XP_026276203.1	GT1		Glycosyltransferase Family 1 protein		
XP_026276262.1	GT1		Glycosyltransferase Family 1 protein		

XP_026276325.1	GH30_1		Glycoside Hydrolase Family 30 protein		
XP_026276440.1	GH35		Glycoside Hydrolase Family 35 protein		
XP_026276449.1	GT49		Glycosyltransferase Family 49 protein		
XP_026276465.1	GH1		Glycoside Hydrolase Family 1 protein		
XP_026276466.1	GH1		Glycoside Hydrolase Family 1 protein		
XP_026276495.1	GT8		Glycosyltransferase Family 8 protein		
XP_026276517.1	GH20		Glycoside Hydrolase Family 20 protein		
XP_026276583.1	GH18	fragment C-term;	Glycoside Hydrolase Family 18 protein		
XP_026276646.1	CBM50		Carbohydrate-Binding Module Family 50 protein		
XP_026276666.1	GH5_8		Glycoside Hydrolase Family 5 protein	HGT	Bacteria
XP_026276732.1	AA3_2		GMC oxidoreductase		
XP_026276765.1	GT92		Glycosyltransferase Family 92 protein		
XP_026276966.1	GH56		Glycoside Hydrolase Family 56 protein		
XP_026277104.1	GH1		Glycoside Hydrolase Family 1 protein		
XP_026277116.1	GH1		Glycoside Hydrolase Family 1 protein		
XP_026277129.1	GH1		Glycoside Hydrolase Family 1 protein		
XP_026277178.1	GH1		Glycoside Hydrolase Family 1 protein		
XP_026277263.1	GT90		Glycosyltransferase Family 90 protein		
XP_026277346.1	GH1		Glycoside Hydrolase Family 1 protein		
XP_026277353.1	GH1		Glycoside Hydrolase Family 1 protein		
XP_026277354.1	GH1		Glycoside Hydrolase Family 1 protein		
XP_026277504.1	GT2		Glycosyltransferase Family 2 protein		
XP_026277582.1	GH13_25-GH133		Glycoside Hydrolase Family 13 / Glycoside Hydrolase Family 133 protein		
XP_026277756.1	GT2		Glycosyltransferase Family 2 protein		
XP_026277826.1	GT92		Glycosyltransferase Family 92 protein		
XP_026277834.1	GT57		Glycosyltransferase Family 57 protein		
XP_026277861.1	AA3_2		GMC oxidoreductase		
XP_026277927.1	GT65		Glycosyltransferase Family 65 protein		
XP_026277980.1	CE9		Carbohydrate Esterase Family 9 protein		
XP_026277983.1	GH1		Glycoside Hydrolase Family 1 protein		
XP_026278042.1	GT41		Glycosyltransferase Family 41 protein		
XP_026278216.1	GH1		Glycoside Hydrolase Family 1 protein		
XP_026278238.1	GT90		Glycosyltransferase Family 90 protein		
XP_026278264.1	GT31		Glycosyltransferase Family 31 protein		
XP_026278300.1	CBM21		Carbohydrate-Binding Module Family 21 protein		
XP_026278401.1	GT24		Glycosyltransferase Family 24 protein		
XP_026278450.1	GT27-CBM13		Glycosyltransferase Family 27 / Carbohydrate-Binding Module Family 13 protein		
XP_026278473.1	GT35		Glycosyltransferase Family 35 protein		
XP_026278527.1	GT10		Glycosyltransferase Family 10 protein		
XP_026278599.1	GH22		Glycoside Hydrolase Family 22 protein		
XP_026278633.1	GH2		Glycoside Hydrolase Family 2 protein		
XP_026278638.1	GH20	(fragment) splicing problem;	Glycoside Hydrolase Family 20 protein		
XP_026278691.1	GH20		Glycoside Hydrolase Family 20 protein		
XP_026278843.1	CBM14		Carbohydrate-Binding Module Family 14 protein		
XP_026278853.1	GT57		Glycosyltransferase Family 57 protein		
XP_026278876.1	AA15		Auxilliary Activities Family 15 protein		
XP_026278917.1	AA3_2		GMC oxidoreductase		
XP_026278952.1	GH9		Glycoside Hydrolase Family 9 protein		
XP_026279015.1	CBM14-CBM14-CBM14-CBM14-CBM14-CBM14-CBM14		Carbohydrate-Binding Module Family 14 protein		

XP_026279096.1	GT2		Glycosyltransferase Family 2 protein		
XP_026279162.1	GH84		Glycoside Hydrolase Family 84 protein		
XP_026279182.1	GH31		Glycoside Hydrolase Family 31 protein		
XP_026279195.1	AA1		Multicopper oxidase		
XP_026279277.1	GH1	fragment N-term;	Glycoside Hydrolase Family 1 protein		
XP_026279283.1	CBM14-CBM14-GH1		Carbohydrate-Binding Module Family 14 / Glycoside Hydrolase Family 1 protein		
XP_026279337.1	GT54	(fragment) splicing problem;	Glycosyltransferase Family 54 protein		
XP_026279359.1	GH18-GH18-CBM14		Glycoside Hydrolase Family 18 / Carbohydrate-Binding Module Family 14 protein		
XP_026279375.1	GT27-CBM13		Glycosyltransferase Family 27 / Carbohydrate-Binding Module Family 13 protein		
XP_026279513.1	PL1_4	(fragment) splicing problem;	Polysaccharide Lyase Family 1 protein	HGT	Complex: Bacteria or Fungi
XP_026279528.1	PL1_4	(fragment) splicing problem;	Polysaccharide Lyase Family 1 protein	HGT	Complex: Bacteria or Fungi
XP_026279697.1	GT1		Glycosyltransferase Family 1 protein		
XP_026279860.1	GT1		Glycosyltransferase Family 1 protein		
XP_026280023.1	GT31		Glycosyltransferase Family 31 protein		
XP_026280167.1	GT8	(fragment) splicing problem;	Glycosyltransferase Family 8 protein		
XP_026280209.1	GT23		Glycosyltransferase Family 23 protein		
XP_026280340.1	GH13_17		Glycoside Hydrolase Family 13 protein		
XP_026280345.1	GH13_17		Glycoside Hydrolase Family 13 protein		
XP_026280363.1	AA3_2		GMC oxidoreductase		
XP_026280366.1	AA3_2		GMC oxidoreductase		
XP_026280374.1	CBM14-CBM14		Carbohydrate-Binding Module Family 14 protein		
XP_026280376.1	GT66	(fragment) splicing problem;	Glycosyltransferase Family 66 protein		
XP_026280402.1	PL1_4	(fragment) splicing problem;	Polysaccharide Lyase Family 1 protein	HGT	Complex: Bacteria or Fungi
XP_026280504.1	GH20		Glycoside Hydrolase Family 20 protein		
XP_026280640.1	CBM47		Carbohydrate-Binding Module Family 47 protein		
XP_026280652.1	GH20		Glycoside Hydrolase Family 20 protein		
XP_026280712.1	GH79		Glycoside Hydrolase Family 79 protein		
XP_026280824.1	GH31	(fragment) splicing problem;	Glycoside Hydrolase Family 31 protein		
XP_026280852.1	AA1		Multicopper oxidase		
XP_026280854.1	GT7		Glycosyltransferase Family 7 protein		
XP_026280905.1	GT98		Glycosyltransferase Family 98 protein		
XP_026280965.1	GH47		Glycoside Hydrolase Family 47 protein		
XP_026280995.1	GT31	(fragment) splicing problem;	Glycosyltransferase Family 31 protein		
XP_026281016.1	GT68		Glycosyltransferase Family 68 protein		
XP_026281037.1	GT21		Glycosyltransferase Family 21 protein		
XP_026281231.1	CBM14		Carbohydrate-Binding Module Family 14 protein		
XP_026281265.1	GH1	fragment C-term;	Glycoside Hydrolase Family 1 protein		
XP_026281402.1	GH20		Glycoside Hydrolase Family 20 protein		
XP_026281449.1	GT33		Glycosyltransferase Family 33 protein		
XP_026281495.1	GT13		Glycosyltransferase Family 13 protein		
XP_026281552.1	AA3_2		GMC oxidoreductase		
XP_026281601.1	CBM48		Carbohydrate-Binding Module Family 48 protein		
XP_026281774.1	GT4		Glycosyltransferase Family 4 protein		
XP_026281838.1	AA3_2		GMC oxidoreductase		
XP_026281850.1	GT31		Glycosyltransferase Family 31 protein		
XP_026281861.1	GT3		Glycosyltransferase Family 3 protein		
XP_026282077.1	GT105		Glycosyltransferase Family 105 protein		
XP_026282345.1	GT59		Glycosyltransferase Family 59 protein		
XP_026282369.1	GH18-CBM14-CBM14-GH18-CBM14-CBM14-CBM14-GH18-GH18-CBM14-GH18		Glycoside Hydrolase Family 18 / Carbohydrate-Binding Module Family 14 protein		

XP_026282505.1	GH79		Glycoside Hydrolase Family 79 protein		
XP_026282691.1	GH1		Glycoside Hydrolase Family 1 protein		
XP_026282723.1	GT27-CBM13	fragment C-term;	Glycosyltransferase Family 27 / Carbohydrate-Binding Module Family 13 protein		
XP_026282959.1	GT8		Glycosyltransferase Family 8 protein		
XP_026283009.1	GT49	(fragment) splicing problem;	Glycosyltransferase Family 49 protein		
XP_026283169.1	GH27		Glycoside Hydrolase Family 27 protein		
XP_026283285.1	GH22		Glycoside Hydrolase Family 22 protein		
XP_026283358.1	GH1		Glycoside Hydrolase Family 1 protein		
XP_026283506.1	CBM14		Carbohydrate-Binding Module Family 14 protein		
XP_026283507.1	CBM14		Carbohydrate-Binding Module Family 14 protein		
XP_026283531.1	GH47		Glycoside Hydrolase Family 47 protein		
XP_026283562.1	GH18-CBM14-CBM14	(fragment) splicing problem;	Glycoside Hydrolase Family 18 / Carbohydrate-Binding Module Family 14 protein		
XP_026283629.1	GT76		Glycosyltransferase Family 76 protein		
XP_026283655.1	GH47		Glycoside Hydrolase Family 47 protein		
XP_026283824.1	GT39		Glycosyltransferase Family 39 protein		
XP_026283909.1	GT1		Glycosyltransferase Family 1 protein		
XP_026283914.1	GH1		Glycoside Hydrolase Family 1 protein		
XP_026284071.1	GH1		Glycoside Hydrolase Family 1 protein		
XP_026284119.1	GT31	(fragment) splicing problem;	Glycosyltransferase Family 31 protein		
XP_026284142.1	CBM20		Carbohydrate-Binding Module Family 20 protein		
XP_026284159.1	GH13_15		Glycoside Hydrolase Family 13 protein		
XP_026284171.1	GT31	(fragment) splicing problem;	Glycosyltransferase Family 31 protein		
XP_026284183.1	CBM47		Carbohydrate-Binding Module Family 47 protein		
XP_026284196.1	AA3_2		GMC oxidoreductase		
XP_026284470.1	AA3_2		GMC oxidoreductase		
XP_026284490.1	GH1		Glycoside Hydrolase Family 1 protein		
XP_026284494.1	GH1		Glycoside Hydrolase Family 1 protein		
XP_026284527.1	GT22		Glycosyltransferase Family 22 protein		
XP_026284547.1	GT4		Glycosyltransferase Family 4 protein		
XP_026284668.1	GH22		Glycoside Hydrolase Family 22 protein		
XP_026284670.1	AA3_2		GMC oxidoreductase		
XP_026284729.1	AA3_2		GMC oxidoreductase		
XP_026284910.1	CBM14		Carbohydrate-Binding Module Family 14 protein		
XP_026284999.1	GH2		Glycoside Hydrolase Family 2 protein		
XP_026285006.1	GH16_4		Glycoside Hydrolase Family 16 protein		
XP_026285007.1	CBM57		Carbohydrate-Binding Module Family 57 protein		
XP_026285066.1	PL1_4	(fragment) splicing problem;	Polysaccharide Lyase Family 1 protein	HGT	Complex: Bacteria or Fungi
XP_026285067.1	PL1_4	(fragment) splicing problem;	Polysaccharide Lyase Family 1 protein	HGT	Complex: Bacteria or Fungi
XP_026285092.1	AA3_2		GMC oxidoreductase		
XP_026285262.1	GH1		Glycoside Hydrolase Family 1 protein		
XP_026285289.1	GH5_8		Glycoside Hydrolase Family 5 protein	HGT	Bacteria
XP_026285291.1	GH5_8		Glycoside Hydrolase Family 5 protein	HGT	Bacteria
XP_026285315.1	GT47-GT64		Glycosyltransferase Family 47 / Glycosyltransferase Family 64 protein		
XP_026285319.1	GH99		Glycoside Hydrolase Family 99 protein		
XP_026285326.1	GH18		Glycoside Hydrolase Family 18 protein		
XP_026285426.1	GT27-CBM13		Glycosyltransferase Family 27 / Carbohydrate-Binding Module Family 13 protein		
XP_026285441.1	GT27-CBM13		Glycosyltransferase Family 27 / Carbohydrate-Binding Module Family 13 protein		
XP_026285502.1	GH1		Glycoside Hydrolase Family 1 protein		
XP_026285581.1	GH1		Glycoside Hydrolase Family 1 protein		

XP_026285608.1	GT110		Glycosyltransferase Family 110 protein		
XP_026285692.1	GH1		Glycoside Hydrolase Family 1 protein		
XP_026285787.1	GH1		Glycoside Hydrolase Family 1 protein		
XP_026285857.1	GH85	(fragment) splicing problem;	Glycoside Hydrolase Family 85 protein		
XP_026285883.1	PL1_4	(fragment) splicing problem;	Polysaccharide Lyase Family 1 protein	HGT	Complex: Bacteria or Fungi
XP_026285908.1	CBM14-CBM14-CBM14		Carbohydrate-Binding Module Family 14 protein		
XP_026285962.1	GT105		Glycosyltransferase Family 105 protein		
XP_026285994.1	GH47	(fragment) splicing problem;	Glycoside Hydrolase Family 47 protein		
XP_026286003.1	AA1		Multicopper oxidase		
XP_026286031.1	GH2		Glycoside Hydrolase Family 2 protein		
XP_026286161.1	GT2		Glycosyltransferase Family 2 protein		
XP_026286293.1	GT31		Glycosyltransferase Family 31 protein		
XP_026286356.1	AA3_2		GMC oxidoreductase		
XP_026286505.1	GT92		Glycosyltransferase Family 92 protein		
XP_026286599.1	GH1		Glycoside Hydrolase Family 1 protein		
XP_026286611.1	AA3_2	(fragment) splicing problem;	GMC oxidoreductase		
XP_026286629.1	GH1	fragment C-term;	Glycoside Hydrolase Family 1 protein		
XP_026286630.1	GH1	fragment N-term;	Glycoside Hydrolase Family 1 protein		
XP_026286632.1	GH1	fragment N-term / C-term;	Glycoside Hydrolase Family 1 protein		
XP_026286772.1	CBM14	fragment N-term;	Carbohydrate-Binding Module Family 14 protein		
XP_026286922.1	GH18-CBM14-CBM14-CBM14	(fragment) splicing problem;	Glycoside Hydrolase Family 18 / Carbohydrate-Binding Module Family 14 protein		
XP_026286949.1	GH16_4		Glycoside Hydrolase Family 16 protein		
XP_026286975.1	CBM14		Carbohydrate-Binding Module Family 14 protein		
XP_026287231.1	GH1	(fragment) splicing problem;	Glycoside Hydrolase Family 1 protein		
XP_026287312.1	GT7		Glycosyltransferase Family 7 protein		
XP_026287319.1	GH22		Glycoside Hydrolase Family 22 protein		
XP_026287364.1	GH1		Glycoside Hydrolase Family 1 protein		
XP_026287407.1	GH22		Glycoside Hydrolase Family 22 protein		
XP_026287599.1	GH1	fragment C-term;	Glycoside Hydrolase Family 1 protein		
XP_026287603.1	GH22-GH22		Glycoside Hydrolase Family 22 protein		
XP_026287675.1	CBM14		Carbohydrate-Binding Module Family 14 protein		
XP_026287748.1	GT13		Glycosyltransferase Family 13 protein		
XP_026287789.1	AA3_2	(fragment) splicing problem;	GMC oxidoreductase		
XP_026287851.1	GH32		Glycoside Hydrolase Family 32 protein	HGT	Bacteria
XP_026287983.1	GH1		Glycoside Hydrolase Family 1 protein		
XP_026287984.1	GH45		Glycoside Hydrolase Family 45 protein	HGT	Fungi
XP_026287990.1	GT105		Glycosyltransferase Family 105 protein		
XP_026288136.1	GH1		Glycoside Hydrolase Family 1 protein		
XP_026288193.1	GH1		Glycoside Hydrolase Family 1 protein		
XP_026288229.1	GH39		Glycoside Hydrolase Family 39 protein		
XP_026288262.1	CBM14		Carbohydrate-Binding Module Family 14 protein		
XP_026288499.1	GH1		Glycoside Hydrolase Family 1 protein		
XP_026288501.1	GH37		Glycoside Hydrolase Family 37 protein		
XP_026288506.1	GH37		Glycoside Hydrolase Family 37 protein		
XP_026288543.1	AA3_2	fragment N-term;	GMC oxidoreductase		
XP_026288627.1	CBM14		Carbohydrate-Binding Module Family 14 protein		
XP_026288628.1	CBM14-CBM14-CBM14-CBM14-CBM14-CBM14		Carbohydrate-Binding Module Family 14 protein		
XP_026288642.1	CBM14-CBM14-CBM14		Carbohydrate-Binding Module Family 14 protein		
XP_026288646.1	CBM14-CBM14-CBM14		Carbohydrate-Binding Module Family 14 protein		

XP_026288705.1	CBM14-CBM14-CBM14		Carbohydrate-Binding Module Family 14 protein		
XP_026288706.1	CBM14-CBM14-CBM14		Carbohydrate-Binding Module Family 14 protein		
XP_026288707.1	CBM14-CBM14-CBM14		Carbohydrate-Binding Module Family 14 protein		
XP_026288737.1	GT32		Glycosyltransferase Family 32 protein		
XP_026288839.1	CBM14-CBM14-GH1		Carbohydrate-Binding Module Family 14 / Glycoside Hydrolase Family 1 protein		
XP_026288857.1	GT22		Glycosyltransferase Family 22 protein		
XP_026288943.1	GH1	(fragment) splicing problem;	Glycoside Hydrolase Family 1 protein		
XP_026288944.1	GH1	fragment N-term;	Glycoside Hydrolase Family 1 protein		
XP_026288982.1	GH1		Glycoside Hydrolase Family 1 protein		
XP_026289055.1	GH20		Glycoside Hydrolase Family 20 protein		
XP_026289145.1	GH22	fragment N-term;	Glycoside Hydrolase Family 22 protein		
XP_026289264.1	GH45		Glycoside Hydrolase Family 45 protein	HGT	Fungi
XP_026289265.1	GH22		Glycoside Hydrolase Family 22 protein		
XP_026289306.1	AA3_2		GMC oxidoreductase		
XP_026289307.1	AA3_2		GMC oxidoreductase		
XP_026289309.1	AA3_2		GMC oxidoreductase		
XP_026289310.1	AA3_2		GMC oxidoreductase		
XP_026289312.1	AA3_2		GMC oxidoreductase		
XP_026289319.1	AA3_2-AA3_2		GMC oxidoreductase		
XP_026289325.1	AA3_2-AA3_2-AA3_2-AA3_2		GMC oxidoreductase		
XP_026289345.1	GT61		Glycosyltransferase Family 61 protein		
XP_026289476.1	AA3_2		GMC oxidoreductase		
XP_026289490.1	AA3_2	fragment C-term;	GMC oxidoreductase		
XP_026289493.1	AA3_2		GMC oxidoreductase		
XP_026289521.1	GH1	fragment N-term / C-term;	Glycoside Hydrolase Family 1 protein		
XP_026289532.1	GT31		Glycosyltransferase Family 31 protein		
XP_026289562.1	CBM14		Carbohydrate-Binding Module Family 14 protein		
XP_026289622.1	GH1		Glycoside Hydrolase Family 1 protein		
XP_026289641.1	PL1_4	(fragment) splicing problem;	Polysaccharide Lyase Family 1 protein	HGT	Complex: Bacteria or Fungi
XP_026289642.1	GH1	fragment N-term;	Glycoside Hydrolase Family 1 protein		
XP_026289643.1	AA3_2		GMC oxidoreductase		
XP_026289679.1	CBM39-GH16_4	(fragment) splicing problem;	Carbohydrate-Binding Module Family 39 / Glycoside Hydrolase Family 16 protein		
XP_026289810.1	GH1		Glycoside Hydrolase Family 1 protein		
XP_026289832.1	GH1	fragment N-term / C-term;	Glycoside Hydrolase Family 1 protein		
XP_026289894.1	GT1	fragment N-term / C-term;	Glycosyltransferase Family 1 protein		
XP_026289900.1	GH1	fragment N-term;	Glycoside Hydrolase Family 1 protein		
XP_026290001.1	CBM48-GH13_8		Carbohydrate-Binding Module Family 48 / Glycoside Hydrolase Family 13 protein		
XP_026290004.1	AA3_2	fragment C-term;	GMC oxidoreductase		
XP_026290091.1	GT31-GT7		Glycosyltransferase Family 31 / Glycosyltransferase Family 7 protein		
XP_026290108.1	GH38	fragment N-term / C-term;	Glycoside Hydrolase Family 38 protein		
XP_026290110.1	GT1		Glycosyltransferase Family 1 protein		
XP_026290116.1	GH1		Glycoside Hydrolase Family 1 protein		
XP_026290124.1	GH1	fragment C-term;	Glycoside Hydrolase Family 1 protein		
XP_026290280.1	GT1		Glycosyltransferase Family 1 protein		
XP_026290293.1	GH1		Glycoside Hydrolase Family 1 protein		
XP_026290315.1	GT50		Glycosyltransferase Family 50 protein		
XP_026290324.1	GT1		Glycosyltransferase Family 1 protein		
XP_026290338.1	AA3_2		GMC oxidoreductase		
XP_026290383.1	GH152		Glycoside Hydrolase Family 152 protein	HGT	Viridiplantae

XP_026290470.1	AA3_2		GMC oxidoreductase		
XP_026290513.1	AA1		Multicopper oxidase		
XP_026290541.1	CBM14-CBM14		Carbohydrate-Binding Module Family 14 protein		
XP_026290559.1	AA15		Auxilliary Activities Family 15 protein		
XP_026290580.1	GT1	fragment N-term;	Glycosyltransferase Family 1 protein		
XP_026290752.1	GH63		Glycoside Hydrolase Family 63 protein		
XP_026290776.1	GH1		Glycoside Hydrolase Family 1 protein		
XP_026290795.1	GH29		Glycoside Hydrolase Family 29 protein		
XP_026290983.1	GH1		Glycoside Hydrolase Family 1 protein		
XP_026291029.1	GH1	fragment C-term;	Glycoside Hydrolase Family 1 protein		
XP_026291031.1	GH22		Glycoside Hydrolase Family 22 protein		
XP_026291034.1	GH1	fragment N-term;	Glycoside Hydrolase Family 1 protein		
XP_026291102.1	CBM14		Carbohydrate-Binding Module Family 14 protein		
XP_026291188.1	GH1	fragment C-term;	Glycoside Hydrolase Family 1 protein		
XP_026291249.1	GT1		Glycosyltransferase Family 1 protein		
XP_026291611.1	GT1		Glycosyltransferase Family 1 protein		
XP_026291613.1	GH1		Glycoside Hydrolase Family 1 protein		
XP_026291692.1	GH18		Glycoside Hydrolase Family 18 protein		
XP_026291724.1	GT16	fragment N-term;	Glycosyltransferase Family 16 protein		
XP_026291832.1	AA3_2	fragment N-term;	GMC oxidoreductase		
XP_026291890.1	GH45	(fragment) splicing problem;	Glycoside Hydrolase Family 45 protein	HGT	Fungi
XP_026291892.1	GH18	fragment N-term;	Glycoside Hydrolase Family 18 protein		
XP_026291972.1	GH31		Glycoside Hydrolase Family 31 protein		
XP_026291995.1	GT64		Glycosyltransferase Family 64 protein		
XP_026292004.1	CBM14		Carbohydrate-Binding Module Family 14 protein		
XP_026292060.1	GH1	fragment N-term / C-term;	Glycoside Hydrolase Family 1 protein		
XP_026292146.1	GT1	fragment C-term;	Glycosyltransferase Family 1 protein		
XP_026292176.1	GT1		Glycosyltransferase Family 1 protein		
XP_026292251.1	GT1		Glycosyltransferase Family 1 protein		
XP_026292396.1	PL1_4	(fragment) splicing problem;	Polysaccharide Lyase Family 1 protein	HGT	Complex: Bacteria or Fungi
XP_026292397.1	PL1_4	(fragment) splicing problem;	Polysaccharide Lyase Family 1 protein	HGT	Complex: Bacteria or Fungi
XP_026292409.1	GT1	fragment C-term;	Glycosyltransferase Family 1 protein		
XP_026292436.1	AA3_2	fragment C-term;	GMC oxidoreductase		
XP_026292490.1	CBM39	fragment N-term;	Carbohydrate-Binding Module Family 39 protein		
XP_026292503.1	AA1	fragment N-term;	Multicopper oxidase		
XP_026292528.1	GH1	fragment N-term;	Glycoside Hydrolase Family 1 protein		
XP_026292714.1	CBM14		Carbohydrate-Binding Module Family 14 protein		
XP_026292767.1	GT43	fragment N-term / C-term;	Glycosyltransferase Family 43 protein		
XP_026292919.1	GH116		Glycoside Hydrolase Family 116 protein		
XP_026292938.1	GH1		Glycoside Hydrolase Family 1 protein		
XP_026292945.1	GH22		Glycoside Hydrolase Family 22 protein		
XP_026293029.1	GT31		Glycosyltransferase Family 31 protein		
XP_026293209.1	AA1		Multicopper oxidase		
XP_026293310.1	AA3_2	fragment N-term;	GMC oxidoreductase		
XP_026293399.1	CE13		Carbohydrate Esterase Family 13 protein		
XP_026293405.1	GH38	(fragment) splicing problem;	Glycoside Hydrolase Family 38 protein		
XP_026293426.1	GH18		Glycoside Hydrolase Family 18 protein		
XP_026293490.1	CBM14-CBM14-CBM14-CBM14		Carbohydrate-Binding Module Family 14 protein		
XP_026293740.1	GH1		Glycoside Hydrolase Family 1 protein		

XP_026293776.1	CBM14		Carbohydrate-Binding Module Family 14 protein		
XP_026293885.1	AA3_2		GMC oxidoreductase		
XP_026293897.1	GH47		Glycoside Hydrolase Family 47 protein		
XP_026294185.1	GT1		Glycosyltransferase Family 1 protein		
XP_026294279.1	GT29		Glycosyltransferase Family 29 protein		
XP_026294292.1	GT31		Glycosyltransferase Family 31 protein		
XP_026294370.1	AA1		Multicopper oxidase		
XP_026294395.1	GT1		Glycosyltransferase Family 1 protein		
XP_026294449.1	GT27-CBM13	(fragment) splicing problem;	Glycosyltransferase Family 27 / Carbohydrate-Binding Module Family 13 protein		
XP_026294573.1	GT31	fragment N-term;	Glycosyltransferase Family 31 protein		
XP_026294605.1	GT49		Glycosyltransferase Family 49 protein		

T. palmi

Protein ID	Description	Model notes	Definition line	HGT	Donor
XP_034229989.1	GH1	fragment N-term;	Glycoside Hydrolase Family 1 protein		
XP_034230049.1	CBM14		Carbohydrate-Binding Module Family 14 protein		
XP_034230158.1	GT1		Glycosyltransferase Family 1 protein		
XP_034230228.1	AA3_2		GMC oxidoreductase		
XP_034230230.1	AA3_2		GMC oxidoreductase		
XP_034230315.1	GT105		Glycosyltransferase Family 105 protein		
XP_034230380.1	CBM14-CBM14		Carbohydrate-Binding Module Family 14 protein		
XP_034230391.1	GT13		Glycosyltransferase Family 13 protein		
XP_034230419.1	GT2		Glycosyltransferase Family 2 protein		
XP_034230455.1	CBM57		Carbohydrate-Binding Module Family 57 protein		
XP_034230479.1	GT61		Glycosyltransferase Family 61 protein		
XP_034230536.1	AA15		Auxilliary Activities Family 15 protein		
XP_034230692.1	GH2		Glycoside Hydrolase Family 2 protein		
XP_034230730.1	GH1		Glycoside Hydrolase Family 1 protein		
XP_034230753.1	GH22		Glycoside Hydrolase Family 22 protein		
XP_034230762.1	GT2		Glycosyltransferase Family 2 protein		
XP_034230845.1	AA3_2		GMC oxidoreductase		
XP_034230866.1	GT43		Glycosyltransferase Family 43 protein		
XP_034230892.1	GT65		Glycosyltransferase Family 65 protein		
XP_034230949.1	AA3_2		GMC oxidoreductase		
XP_034230950.1	AA3_2		GMC oxidoreductase		
XP_034231003.1	GT1		Glycosyltransferase Family 1 protein		
XP_034231019.1	GT92		Glycosyltransferase Family 92 protein		
XP_034231040.1	AA3_2-AA3_2		GMC oxidoreductase		
XP_034231041.1	AA3_2		GMC oxidoreductase		
XP_034231075.1	GH22		Glycoside Hydrolase Family 22 protein		
XP_034231185.1	CBM14		Carbohydrate-Binding Module Family 14 protein		
XP_034231187.1	CBM14		Carbohydrate-Binding Module Family 14 protein		
XP_034231281.1	AA1		Multicopper oxidase		
XP_034231304.1	GT1		Glycosyltransferase Family 1 protein		
XP_034231365.1	GH37		Glycoside Hydrolase Family 37 protein		
XP_034231424.1	AA3_2-AA3_2		GMC oxidoreductase		
XP_034231426.1	GT13		Glycosyltransferase Family 13 protein		
XP_034231448.1	GH16_4		Glycoside Hydrolase Family 16 protein		

XP_034231449.1	AA1		Multicopper oxidase		
XP_034231457.1	GT31		Glycosyltransferase Family 31 protein		
XP_034231710.1	CBM47		Carbohydrate-Binding Module Family 47 protein		
XP_034231727.1	GT41		Glycosyltransferase Family 41 protein		
XP_034231998.1	GT105		Glycosyltransferase Family 105 protein		
XP_034232030.1	GH20		Glycoside Hydrolase Family 20 protein		
XP_034232055.1	CBM47		Carbohydrate-Binding Module Family 47 protein		
XP_034232152.1	GH1		Glycoside Hydrolase Family 1 protein		
XP_034232312.1	GT31		Glycosyltransferase Family 31 protein		
XP_034232383.1	GH32		Glycoside Hydrolase Family 32 protein	HGT	Bacteria
XP_034232398.1	GH1		Glycoside Hydrolase Family 1 protein		
XP_034232426.1	AA3_2		GMC oxidoreductase		
XP_034232579.1	AA3_2	fragment N-term;	GMC oxidoreductase		
XP_034232815.1	GT1		Glycosyltransferase Family 1 protein		
XP_034232830.1	GH1	fragment N-term;	Glycoside Hydrolase Family 1 protein		
XP_034232847.1	GT49		Glycosyltransferase Family 49 protein		
XP_034232873.1	GT31		Glycosyltransferase Family 31 protein		
XP_034232917.1	AA1		Multicopper oxidase		
XP_034232935.1	GH22-GH22		Glycoside Hydrolase Family 22 protein		
XP_034233104.1	GH13_15		Glycoside Hydrolase Family 13 protein		
XP_034233350.1	GH45	(fragment) splicing problem;	Glycoside Hydrolase Family 45 protein	HGT	Fungi
XP_034233351.1	AA3_2	fragment N-term;	GMC oxidoreductase		
XP_034233420.1	GH32		Glycoside Hydrolase Family 32 protein	HGT	Bacteria
XP_034233461.1	GH35		Glycoside Hydrolase Family 35 protein		
XP_034233657.1	GT8	(fragment) splicing problem;	Glycosyltransferase Family 8 protein		
XP_034233979.1	CBM14-CBM14-CBM14		Carbohydrate-Binding Module Family 14 protein		
XP_034233988.1	GH1		Glycoside Hydrolase Family 1 protein		
XP_034234045.1	CBM14-CBM14		Carbohydrate-Binding Module Family 14 protein		
XP_034234061.1	CBM14		Carbohydrate-Binding Module Family 14 protein		
XP_034234070.1	GT49		Glycosyltransferase Family 49 protein		
XP_034234073.1	GH39	fragment N-term;	Glycoside Hydrolase Family 39 protein		
XP_034234087.1	GH18		Glycoside Hydrolase Family 18 protein		
XP_034234108.1	GH20		Glycoside Hydrolase Family 20 protein		
XP_034234142.1	CBM14-CBM14-CBM14		Carbohydrate-Binding Module Family 14 protein		
XP_034234196.1	AA3_2		GMC oxidoreductase		
XP_034234238.1	CBM14-CBM14-CBM14		Carbohydrate-Binding Module Family 14 protein		
XP_034234307.1	GH1		Glycoside Hydrolase Family 1 protein		
XP_034234389.1	GH35		Glycoside Hydrolase Family 35 protein		
XP_034234464.1	GH20		Glycoside Hydrolase Family 20 protein		
XP_034234651.1	GT43		Glycosyltransferase Family 43 protein		
XP_034234691.1	CBM14-CBM14-CBM14		Carbohydrate-Binding Module Family 14 protein		
XP_034234754.1	GT1		Glycosyltransferase Family 1 protein		
XP_034234765.1	CBM14-CBM14-CBM14		Carbohydrate-Binding Module Family 14 protein		
XP_034234918.1	CBM14-CBM14-CBM14		Carbohydrate-Binding Module Family 14 protein		
XP_034234920.1	CBM14-CBM14-CBM14		Carbohydrate-Binding Module Family 14 protein		
XP_034234999.1	AA3_2	fragment N-term;	GMC oxidoreductase		
XP_034235276.1	CBM14-CBM14		Carbohydrate-Binding Module Family 14 protein		
XP_034235278.1	GH56		Glycoside Hydrolase Family 56 protein		
XP_034235375.1	GT16		Glycosyltransferase Family 16 protein		

XP_034235409.1	GH20	(fragment) splicing problem;	Glycoside Hydrolase Family 20 protein		
XP_034235640.1	PL1_4	(fragment) splicing problem;	Polysaccharide Lyase Family 1 protein	HGT	Complex: Bacteria or Fungi
XP_034235648.1	GH1		Glycoside Hydrolase Family 1 protein		
XP_034235670.1	GH1	fragment C-term;	Glycoside Hydrolase Family 1 protein		
XP_034235714.1	GT1		Glycosyltransferase Family 1 protein		
XP_034235752.1	GH22		Glycoside Hydrolase Family 22 protein		
XP_034235817.1	GT2		Glycosyltransferase Family 2 protein		
XP_034235838.1	GT90		Glycosyltransferase Family 90 protein		
XP_034235996.1	CBM14		Carbohydrate-Binding Module Family 14 protein		
XP_034236588.1	GH45		Glycoside Hydrolase Family 45 protein	HGT	Fungi
XP_034236620.1	AA3_2		GMC oxidoreductase		
XP_034236632.1	GH1		Glycoside Hydrolase Family 1 protein		
XP_034236643.1	GH9		Glycoside Hydrolase Family 9 protein		
XP_034236904.1	GT1		Glycosyltransferase Family 1 protein		
XP_034237109.1	GT61		Glycosyltransferase Family 61 protein		
XP_034237121.1	GH1		Glycoside Hydrolase Family 1 protein		
XP_034237190.1	GT57		Glycosyltransferase Family 57 protein		
XP_034237191.1	PL1_4	(fragment) splicing problem;	Polysaccharide Lyase Family 1 protein	HGT	Complex: Bacteria or Fungi
XP_034237329.1	AA3_2	fragment C-term;	GMC oxidoreductase		
XP_034237346.1	AA3_2		GMC oxidoreductase		
XP_034237429.1	AA15		Auxilliary Activities Family 15 protein		
XP_034237478.1	CBM14		Carbohydrate-Binding Module Family 14 protein		
XP_034237553.1	GH20		Glycoside Hydrolase Family 20 protein		
XP_034237725.1	GH1		Glycoside Hydrolase Family 1 protein		
XP_034237726.1	GH1	fragment N-term;	Glycoside Hydrolase Family 1 protein		
XP_034237728.1	GH1	fragment C-term;	Glycoside Hydrolase Family 1 protein		
XP_034237737.1	CBM20		Carbohydrate-Binding Module Family 20 protein		
XP_034237899.1	GH1		Glycoside Hydrolase Family 1 protein		
XP_034238314.1	GH13_17		Glycoside Hydrolase Family 13 protein		
XP_034238335.1	GT29		Glycosyltransferase Family 29 protein		
XP_034238381.1	GT31		Glycosyltransferase Family 31 protein		
XP_034238486.1	GT66		Glycosyltransferase Family 66 protein		
XP_034238644.1	GH1		Glycoside Hydrolase Family 1 protein		
XP_034238785.1	GT24		Glycosyltransferase Family 24 protein		
XP_034238855.1	AA3_2		GMC oxidoreductase		
XP_034238883.1	GT22		Glycosyltransferase Family 22 protein		
XP_034238963.1	GH18		Glycoside Hydrolase Family 18 protein		
XP_034238972.1	GT10		Glycosyltransferase Family 10 protein		
XP_034239010.1	GH38		Glycoside Hydrolase Family 38 protein		
XP_034239232.1	GT22		Glycosyltransferase Family 22 protein		
XP_034239330.1	GT23		Glycosyltransferase Family 23 protein		
XP_034239395.1	GH30_1	fragment N-term;	Glycoside Hydrolase Family 30 protein		
XP_034239440.1	PL1_4	fragment N-term / C-term;	Polysaccharide Lyase Family 1 protein	HGT	Complex: Bacteria or Fungi
XP_034239465.1	CBM39-GH16_4		Carbohydrate-Binding Module Family 39 / Glycoside Hydrolase Family 16 protein		
XP_034239544.1	CBM39-GH16_4	(fragment) splicing problem;	Carbohydrate-Binding Module Family 39 / Glycoside Hydrolase Family 16 protein		
XP_034239569.1	GH18-CBM14		Glycoside Hydrolase Family 18 / Carbohydrate-Binding Module Family 14 protein		
XP_034239606.1	GT16		Glycosyltransferase Family 16 protein		
XP_034239611.1	GH22		Glycoside Hydrolase Family 22 protein		
XP_034239672.1	GT21	(fragment) splicing problem;	Glycosyltransferase Family 21 protein		

XP_034239705.1	GH27		Glycoside Hydrolase Family 27 protein		
XP_034239717.1	CE9	(fragment) splicing problem;	Carbohydrate Esterase Family 9 protein		
XP_034239735.1	GT1		Glycosyltransferase Family 1 protein		
XP_034239858.1	GH2		Glycoside Hydrolase Family 2 protein		
XP_034239875.1	GT57		Glycosyltransferase Family 57 protein		
XP_034239912.1	GH31		Glycoside Hydrolase Family 31 protein		
XP_034240246.1	AA1		Multicopper oxidase		
XP_034240255.1	CBM14		Carbohydrate-Binding Module Family 14 protein		
XP_034240262.1	AA3_2	(fragment) splicing problem;	GMC oxidoreductase		
XP_034240278.1	AA3_2	(fragment) splicing problem;	GMC oxidoreductase		
XP_034240369.1	GH30_1		Glycoside Hydrolase Family 30 protein		
XP_034240415.1	GH16_4		Glycoside Hydrolase Family 16 protein		
XP_034240427.1	CBM14		Carbohydrate-Binding Module Family 14 protein		
XP_034240608.1	GH1		Glycoside Hydrolase Family 1 protein		
XP_034240623.1	GH1		Glycoside Hydrolase Family 1 protein		
XP_034240627.1	GT22		Glycosyltransferase Family 22 protein		
XP_034240632.1	GT8		Glycosyltransferase Family 8 protein		
XP_034240678.1	GH1	fragment C-term;	Glycoside Hydrolase Family 1 protein		
XP_034240731.1	GH1	fragment N-term;	Glycoside Hydrolase Family 1 protein		
XP_034240850.1	GH1		Glycoside Hydrolase Family 1 protein		
XP_034240865.1	GT1		Glycosyltransferase Family 1 protein		
XP_034240903.1	GH16_4		Glycoside Hydrolase Family 16 protein		
XP_034240904.1	GH16_4		Glycoside Hydrolase Family 16 protein		
XP_034241038.1	PL1_4	(fragment) splicing problem;	Polysaccharide Lyase Family 1 protein	HGT	Complex: Bacteria or Fungi
XP_034241133.1	GT105	(fragment) splicing problem;	Glycosyltransferase Family 105 protein		
XP_034241502.1	GT1		Glycosyltransferase Family 1 protein		
XP_034241515.1	GT31		Glycosyltransferase Family 31 protein		
XP_034241559.1	GT20		Glycosyltransferase Family 20 protein		
XP_034241740.1	GH13_25-GH133		Glycoside Hydrolase Family 13 / Glycoside Hydrolase Family 133 protein		
XP_034241770.1	CBM14		Carbohydrate-Binding Module Family 14 protein		
XP_034241806.1	GT31		Glycosyltransferase Family 31 protein		
XP_034241934.1	CBM14		Carbohydrate-Binding Module Family 14 protein		
XP_034242052.1	GH5_8		Glycoside Hydrolase Family 5 protein	HGT	Bacteria
XP_034242065.1	GH1		Glycoside Hydrolase Family 1 protein		
XP_034242067.1	GH1	fragment N-term;	Glycoside Hydrolase Family 1 protein		
XP_034242068.1	GH1		Glycoside Hydrolase Family 1 protein		
XP_034242079.1	CBM14-CBM14-GH1		Carbohydrate-Binding Module Family 14 / Glycoside Hydrolase Family 1 protein		
XP_034242082.1	GT1		Glycosyltransferase Family 1 protein		
XP_034242145.1	GH30_1		Glycoside Hydrolase Family 30 protein		
XP_034242173.1	GT1-GT1		Glycosyltransferase Family 1 protein		
XP_034242309.1	GH1		Glycoside Hydrolase Family 1 protein		
XP_034242338.1	GT54		Glycosyltransferase Family 54 protein		
XP_034242400.1	CBM14-CBM14-CBM14-CBM14-CBM14-CBM14-CBM14		Carbohydrate-Binding Module Family 14 protein		
XP_034242473.1	GT8		Glycosyltransferase Family 8 protein		
XP_034242578.1	GH5_8		Glycoside Hydrolase Family 5 protein	HGT	Bacteria
XP_034242622.1	GH47		Glycoside Hydrolase Family 47 protein		
XP_034242715.1	AA3_2		GMC oxidoreductase		
XP_034242717.1	AA3_2		GMC oxidoreductase		
XP_034242730.1	GH1		Glycoside Hydrolase Family 1 protein		

XP_034242821.1	GT43		Glycosyltransferase Family 43 protein		
XP_034242876.1	GT2		Glycosyltransferase Family 2 protein		
XP_034242958.1	GH18-CBM14-CBM14-GH18-CBM14-CBM14-CBM14-GH18-GH18-CBM14-GH18		Glycoside Hydrolase Family 18 / Carbohydrate-Binding Module Family 14 protein		
XP_034243006.1	GT1		Glycosyltransferase Family 1 protein		
XP_034243155.1	AA3_2		GMC oxidoreductase		
XP_034243232.1	GH20		Glycoside Hydrolase Family 20 protein		
XP_034243548.1	AA3_2	fragment C-term;	GMC oxidoreductase		
XP_034243618.1	GT76		Glycosyltransferase Family 76 protein		
XP_034243778.1	GT31		Glycosyltransferase Family 31 protein		
XP_034243895.1	GH2		Glycoside Hydrolase Family 2 protein		
XP_034243932.1	GT1		Glycosyltransferase Family 1 protein		
XP_034244054.1	GT39		Glycosyltransferase Family 39 protein		
XP_034244075.1	GH63		Glycoside Hydrolase Family 63 protein		
XP_034244218.1	GH47		Glycoside Hydrolase Family 47 protein		
XP_034244463.1	GT27-CBM13		Glycosyltransferase Family 27 / Carbohydrate-Binding Module Family 13 protein		
XP_034244583.1	AA3_2		GMC oxidoreductase		
XP_034244609.1	GT27-CBM13		Glycosyltransferase Family 27 / Carbohydrate-Binding Module Family 13 protein		
XP_034244814.1	GH31		Glycoside Hydrolase Family 31 protein		
XP_034244844.1	GT50		Glycosyltransferase Family 50 protein		
XP_034244861.1	CBM21		Carbohydrate-Binding Module Family 21 protein		
XP_034244864.1	CBM14-CBM14		Carbohydrate-Binding Module Family 14 protein		
XP_034244872.1	CBM50		Carbohydrate-Binding Module Family 50 protein		
XP_034244875.1	GH22		Glycoside Hydrolase Family 22 protein		
XP_034244880.1	GH22		Glycoside Hydrolase Family 22 protein		
XP_034244905.1	GH1		Glycoside Hydrolase Family 1 protein		
XP_034244999.1	GH1		Glycoside Hydrolase Family 1 protein		
XP_034245055.1	AA3_2		GMC oxidoreductase		
XP_034245074.1	AA3_2		GMC oxidoreductase		
XP_034245312.1	AA1		Multicopper oxidase		
XP_034245357.1	GH1		Glycoside Hydrolase Family 1 protein		
XP_034245606.1	GH30_1		Glycoside Hydrolase Family 30 protein		
XP_034245684.1	GH1	fragment N-term / C-term;	Glycoside Hydrolase Family 1 protein		
XP_034245696.1	GH1		Glycoside Hydrolase Family 1 protein		
XP_034245819.1	CBM43-CBM43-CBM43-CBM43		Carbohydrate-Binding Module Family 43 protein	HGT	Viridiplantae
XP_034246068.1	GT27-CBM13		Glycosyltransferase Family 27 / Carbohydrate-Binding Module Family 13 protein		
XP_034246129.1	CBM14-CBM14-GH1		Carbohydrate-Binding Module Family 14 / Glycoside Hydrolase Family 1 protein		
XP_034246266.1	GT49		Glycosyltransferase Family 49 protein		
XP_034246495.1	GH18		Glycoside Hydrolase Family 18 protein		
XP_034246754.1	GT2		Glycosyltransferase Family 2 protein		
XP_034246764.1	CBM14-CBM14-GH1	(fragment) splicing problem;	Carbohydrate-Binding Module Family 14 / Glycoside Hydrolase Family 1 protein		
XP_034246835.1	GT105	fragment N-term;	Glycosyltransferase Family 105 protein		
XP_034246846.1	GH47		Glycoside Hydrolase Family 47 protein		
XP_034246957.1	GH116		Glycoside Hydrolase Family 116 protein		
XP_034247376.1	GH152		Glycoside Hydrolase Family 152 protein	HGT	Viridiplantae
XP_034247478.1	GH38	fragment C-term;	Glycoside Hydrolase Family 38 protein		
XP_034247610.1	GT105		Glycosyltransferase Family 105 protein		
XP_034247673.1	GH152		Glycoside Hydrolase Family 152 protein	HGT	Viridiplantae
XP_034247731.1	GT31		Glycosyltransferase Family 31 protein		
XP_034247819.1	AA3_2		GMC oxidoreductase		

XP_034247857.1	AA3_2		GMC oxidoreductase		
XP_034247887.1	GT27-CBM13		Glycosyltransferase Family 27 / Carbohydrate-Binding Module Family 13 protein		
XP_034247964.1	GH1		Glycoside Hydrolase Family 1 protein		
XP_034248138.1	GH85	(fragment) splicing problem;	Glycoside Hydrolase Family 85 protein		
XP_034248270.1	GH18-GH18-CBM14		Glycoside Hydrolase Family 18 / Carbohydrate-Binding Module Family 14 protein		
XP_034248354.1	PL1_4	fragment C-term;	Polysaccharide Lyase Family 1 protein	HGT	Complex: Bacteria or Fungi
XP_034248372.1	GH18-CBM14-CBM14-CBM14	(fragment) splicing problem;	Glycoside Hydrolase Family 18 / Carbohydrate-Binding Module Family 14 protein		
XP_034248377.1	GT92		Glycosyltransferase Family 92 protein		
XP_034248438.1	CBM14		Carbohydrate-Binding Module Family 14 protein		
XP_034248531.1	AA15		Auxilliary Activities Family 15 protein		
XP_034248552.1	PL1_4	fragment C-term;	Polysaccharide Lyase Family 1 protein	HGT	Complex: Bacteria or Fungi
XP_034248577.1	GT3		Glycosyltransferase Family 3 protein		
XP_034248646.1	GH1	fragment C-term;	Glycoside Hydrolase Family 1 protein		
XP_034248773.1	CBM14-CBM14-GH1		Carbohydrate-Binding Module Family 14 / Glycoside Hydrolase Family 1 protein		
XP_034248903.1	GT10	(fragment) splicing problem;	Glycosyltransferase Family 10 protein		
XP_034248925.1	GH37		Glycoside Hydrolase Family 37 protein		
XP_034248976.1	GH1		Glycoside Hydrolase Family 1 protein		
XP_034248983.1	CBM14		Carbohydrate-Binding Module Family 14 protein		
XP_034249033.1	GT4		Glycosyltransferase Family 4 protein		
XP_034249124.1	GH37		Glycoside Hydrolase Family 37 protein		
XP_034249179.1	GH1		Glycoside Hydrolase Family 1 protein		
XP_034249322.1	GH1		Glycoside Hydrolase Family 1 protein		
XP_034249356.1	GH1		Glycoside Hydrolase Family 1 protein		
XP_034249360.1	GT39		Glycosyltransferase Family 39 protein		
XP_034249440.1	GH38		Glycoside Hydrolase Family 38 protein		
XP_034249611.1	CBM13	(fragment) splicing problem;	Carbohydrate-Binding Module Family 13 protein		
XP_034249645.1	GT31-GT7		Glycosyltransferase Family 31 / Glycosyltransferase Family 7 protein		
XP_034249763.1	GT49		Glycosyltransferase Family 49 protein		
XP_034249890.1	GH1	(fragment) splicing problem;	Glycoside Hydrolase Family 1 protein		
XP_034249940.1	GH13		Glycoside Hydrolase Family 13 protein		
XP_034249956.1	GT8-GT49	(fragment) splicing problem;	Glycosyltransferase Family 8 / Glycosyltransferase Family 49 protein		
XP_034250000.1	GT14		Glycosyltransferase Family 14 protein		
XP_034250014.1	GH31		Glycoside Hydrolase Family 31 protein		
XP_034250019.1	GH18-CBM14		Glycoside Hydrolase Family 18 / Carbohydrate-Binding Module Family 14 protein		
XP_034250029.1	GT47-GT64		Glycosyltransferase Family 47 / Glycosyltransferase Family 64 protein		
XP_034250033.1	GH1	(fragment) splicing problem;	Glycoside Hydrolase Family 1 protein		
XP_034250131.1	GT1		Glycosyltransferase Family 1 protein		
XP_034250265.1	GT1	fragment N-term;	Glycosyltransferase Family 1 protein		
XP_034250391.1	AA3_2		GMC oxidoreductase		
XP_034250679.1	GT66		Glycosyltransferase Family 66 protein		
XP_034250782.1	GH13		Glycoside Hydrolase Family 13 protein		
XP_034250870.1	GH29		Glycoside Hydrolase Family 29 protein		
XP_034250949.1	GH2		Glycoside Hydrolase Family 2 protein		
XP_034251003.1	CBM21		Carbohydrate-Binding Module Family 21 protein		
XP_034251442.1	CBM48-GH13_8		Carbohydrate-Binding Module Family 48 / Glycoside Hydrolase Family 13 protein		
XP_034251591.1	GT59		Glycosyltransferase Family 59 protein		
XP_034251595.1	GT31		Glycosyltransferase Family 31 protein		
XP_034251951.1	GT7		Glycosyltransferase Family 7 protein		
XP_034252032.1	GH1		Glycoside Hydrolase Family 1 protein		

XP_034252322.1	GH1		Glycoside Hydrolase Family 1 protein		
XP_034252555.1	GH1	fragment N-term;	Glycoside Hydrolase Family 1 protein		
XP_034252647.1	GT31		Glycosyltransferase Family 31 protein		
XP_034252780.1	GT47-GT64		Glycosyltransferase Family 47 / Glycosyltransferase Family 64 protein		
XP_034252867.1	CBM14		Carbohydrate-Binding Module Family 14 protein		
XP_034252888.1	GT22		Glycosyltransferase Family 22 protein		
XP_034252975.1	CBM14		Carbohydrate-Binding Module Family 14 protein		
XP_034252989.1	GH18		Glycoside Hydrolase Family 18 protein		
XP_034253001.1	GH99		Glycoside Hydrolase Family 99 protein		
XP_034253060.1	CBM14		Carbohydrate-Binding Module Family 14 protein		
XP_034253076.1	GH1	fragment N-term;	Glycoside Hydrolase Family 1 protein		
XP_034253121.1	GT27-CBM13		Glycosyltransferase Family 27 / Carbohydrate-Binding Module Family 13 protein		
XP_034253302.1	GT47-GT64		Glycosyltransferase Family 47 / Glycosyltransferase Family 64 protein		
XP_034253455.1	GT1		Glycosyltransferase Family 1 protein		
XP_034253511.1	CBM14		Carbohydrate-Binding Module Family 14 protein		
XP_034253547.1	CBM14		Carbohydrate-Binding Module Family 14 protein		
XP_034253708.1	GT4		Glycosyltransferase Family 4 protein		
XP_034253709.1	CBM14	(fragment) splicing problem;	Carbohydrate-Binding Module Family 14 protein		
XP_034253736.1	CBM14		Carbohydrate-Binding Module Family 14 protein		
XP_034253757.1	CBM14		Carbohydrate-Binding Module Family 14 protein		
XP_034253769.1	PL1_4	fragment C-term;	Polysaccharide Lyase Family 1 protein	HGT	Complex: Bacteria or Fungi
XP_034253771.1	PL1_4	fragment C-term;	Polysaccharide Lyase Family 1 protein	HGT	Complex: Bacteria or Fungi
XP_034254025.1	GT4		Glycosyltransferase Family 4 protein		
XP_034254051.1	GH47		Glycoside Hydrolase Family 47 protein		
XP_034254370.1	GT27-CBM13		Glycosyltransferase Family 27 / Carbohydrate-Binding Module Family 13 protein		
XP_034254403.1	GT27-CBM13		Glycosyltransferase Family 27 / Carbohydrate-Binding Module Family 13 protein		
XP_034254538.1	GH31		Glycoside Hydrolase Family 31 protein		
XP_034254592.1	AA3_2		GMC oxidoreductase		
XP_034254627.1	GH84		Glycoside Hydrolase Family 84 protein		
XP_034254640.1	GH79		Glycoside Hydrolase Family 79 protein		
XP_034254683.1	GH89	fragment N-term;	Glycoside Hydrolase Family 89 protein		
XP_034254706.1	GH79		Glycoside Hydrolase Family 79 protein		
XP_034254735.1	GT7		Glycosyltransferase Family 7 protein		
XP_034254881.1	GT68		Glycosyltransferase Family 68 protein		
XP_034254926.1	GH47		Glycoside Hydrolase Family 47 protein		
XP_034254936.1	CBM14-CBM14-GH1		Carbohydrate-Binding Module Family 14 / Glycoside Hydrolase Family 1 protein		
XP_034254938.1	PL1_4	fragment C-term;	Polysaccharide Lyase Family 1 protein	HGT	Complex: Bacteria or Fungi
XP_034255023.1	AA3_2		GMC oxidoreductase		
XP_034255257.1	GT7		Glycosyltransferase Family 7 protein		
XP_034255289.1	GT25		Glycosyltransferase Family 25 protein		
XP_034255299.1	AA3_2		GMC oxidoreductase		
XP_034255300.1	GH22		Glycoside Hydrolase Family 22 protein		
XP_034255301.1	GH22		Glycoside Hydrolase Family 22 protein		
XP_034255332.1	GH1		Glycoside Hydrolase Family 1 protein		
XP_034255409.1	GT54		Glycosyltransferase Family 54 protein		
XP_034255447.1	GT1		Glycosyltransferase Family 1 protein		
XP_034255541.1	GH22		Glycoside Hydrolase Family 22 protein		
XP_034255542.1	GH22		Glycoside Hydrolase Family 22 protein		
XP_034255655.1	AA3_2		GMC oxidoreductase		

XP_034255658.1	AA3_2		GMC oxidoreductase			
XP_034255772.1	GT58		Glycosyltransferase Family 58 protein			
XP_034255852.1	GT35		Glycosyltransferase Family 35 protein			
XP_034255893.1	GH20		Glycoside Hydrolase Family 20 protein			
XP_034255982.1	GT33		Glycosyltransferase Family 33 protein			
XP_034256147.1	AA1		Multicopper oxidase			
XP_034256234.1	CBM48		Carbohydrate-Binding Module Family 48 protein			
XP_034256246.1	AA3_2		GMC oxidoreductase			
XP_034256272.1	GT31-GT7		Glycosyltransferase Family 31 / Glycosyltransferase Family 7 protein			
XP_034256300.1	GT31	(fragment) splicing problem;	Glycosyltransferase Family 31 protein			
XP_034256316.1	GT98		Glycosyltransferase Family 98 protein			
XP_034256384.1	CBM14		Carbohydrate-Binding Module Family 14 protein			
XP_034256564.1	AA3_2		GMC oxidoreductase			
XP_034256691.1	GH1		Glycoside Hydrolase Family 1 protein			
XP_034256971.1	CBM14		Carbohydrate-Binding Module Family 14 protein			
XP_034256998.1	GT105		Glycosyltransferase Family 105 protein			
XP_034257021.1	CBM14		Carbohydrate-Binding Module Family 14 protein			
XP_034257023.1	AA1		Multicopper oxidase			
XP_034257033.1	AA3_2		GMC oxidoreductase			

Supplementary Table 6: Validated HGT candidates from potential bacterial, fungal or viridiplantae donors for *T. vaporariorum*

Bacterial donor:

HGT event	Sequence name	Origin of donor sequences	Alternative topology Monophyly of Metazoa	Similarities with the donor sequences		Length and coverage of the alignment with the donor sequences		Local score for genomic environment	Homologs found in the *B. tabaci* cryptic species			Annotation (BLAST NR)	CAZy
				max id	average id	average aln length	average coverage		Btab_MEAM1	Btab_MED	Btab_SSA		
TvaB01	TLow_01347-RA<br>TLow_02144-RA<br>TLow_02157-RA<br>Tv_13818-RA<br>Tv_13862-RA<br>Tv_15776-RA	Bacteria	/	34.2<br>30.8<br>31.4<br>30.2<br>45.3<br>31.2	28.2<br>28.6<br>27.5<br>27.9<br>34.6<br>26.0	430<br>446<br>353<br>449<br>110<br>438	81.7<br>76.7<br>71.7<br>76.6<br>47.5<br>67.1	0.92<br>0.86<br>0.88<br>0.85<br>1.0<br>0.83	Bta10852,<br>Bta10853	BTA009628.1,<br>BTA009629.1,<br>BTA025251.1,<br>BTA025252.1,<br>BTA025253.1	Ssa11036	AAA family ATPase	
TvaB02	Tv_01895-RA	Bacteria	1	77.7	60.6	419	49.2	0.64	Bta00841,<br>Bta01937	BTA023651.1	Ssa01553	adenosylmethionine--8-amino-7-oxononanoate transaminase	
TvaB03	Tv_02831-RA<br>Tv_11854-RA<br>Tv_12101-RA<br>Tv_15086-RA<br>Tv_15720-RA<br>Tv_15881-RA	Bacteria	/	53.9<br>53.9<br>49.0<br>60.8<br>48.1<br>52.0	50.5<br>50.5<br>43.6<br>55.7<br>44.1<br>43.8	151<br>151<br>121<br>78<br>153<br>125	95.6<br>95.8<br>30.2<br>89.0<br>33.7<br>70.0	0.87<br>0.71<br>0.85<br>0.84<br>0.85<br>0.76	Bta00747,<br>Bta13975		Ssa08703,<br>Ssa14910	dUTP diphosphatase	
TvaB04	Tv_15063-RA<br>Tv_15070-RA	Bacteria	/	46.3<br>34.0	38.6<br>32.9	135<br>405	64.5<br>89.1	0.86<br>0.86				AAA family ATPase	
TvaB05	Tv_01894-RA	Bacteria	1	76.2	55.8	202	93.9	0.74	Bta00840,<br>Bta01938		Ssa01088	dethiobiotin synthase	
TvaB06	Tv_05628-RA	Bacteria	0	40.1	32.3	433	86.0	0.79	Bta12386	BTA006502.1	Ssa03543,<br>Ssa03544		
TvaB07	Tv_00146-RA<br>Tv_00148-RA<br>Tv_02102-RA<br>Tv_02106-RA<br>Tv_01481-RA<br>Tv_15366-RA	Bacteria	/	32<br>30.8<br>32<br>28.4<br>30.7<br>36.7	30.1<br>28.6<br>28.9<br>25.5<br>28.8<br>31.2	512<br>546<br>498<br>213<br>550<br>399	72.0<br>74.5<br>67.1<br>72.9<br>71.2<br>80.2	0.9<br>0.9<br>0.62<br>0.62<br>0.96<br>0.95	Bta00829	BTA007316.1,<br>BTA011402.1	scaffold554257_3<br>12_1956	AAA family ATPase	
TvaB08	Tv_12316-RA	Bacteria	/	69.4	67.8	216	99.5	0.88				nucleoside/nucleotide kinase family protein	
TvaB09	TLow_01825-RA<br>Tv_14938-RA	Bacteria	1	33.1<br>33.7	30.2<br>28.2	262<br>165	78.4<br>20.8	0.96<br>0.88				Rpn family recombination-promoting nuclease/putative	
TvaB10	Tv_04079-RA	Bacteria	/	65.4	60.0	85	72.0	0.92					GH13
TvaB11	Tv_13090-RA	Bacteria	/	56.8	47.6	259	95.9	1.0				PhzF family phenazine biosynthesis isomerase	
TvaB12	TLow_01333-RA	Bacteria	0	31.2	29.5	279	82.6	0.96				Rpn family recombination-promoting nuclease/putative transposase	
TvaB13	Tv_05804-RA	Bacteria	/	32.1	28.9	481	78.5	0.8				AAA family ATPase	
TvaB14	Tv_01789-RA<br>Tv_08710-RA	Bacteria	/	34.3<br>31.0	31.3<br>29.0	463<br>516	74.8<br>85.0	0.92<br>0.96				AAA family ATPase	
TvaB15	Tv_04874-RA	Bacteria	/	54.8	52.4	155	84.0	0.96	Bta03797	BTA000817.1	Ssa13701	methylated-DNA--[protein]-cysteine S-methyltransferase	
TvaB16	Tv_06835-RA	Bacteria	/	45.0	41.1	158	80.0	0.88	Bta15103	BTA020874.1	Ssa07364	chorismate mutase	
TvaB17	Tv_00487-RA<br>Tv_11451-RA	Bacteria	/	37.6<br>31.6	32.1<br>29.4	129<br>554	82.5<br>73.0	0.77<br>1.0	Bta05013,<br>Bta10549,<br>Bta14973	BTA002393.1	Ssa01599,<br>Ssa10865,<br>Ssa10873,<br>Ssa12918,<br>Ssa13905,<br>Ssa14120,<br>Ssa15393	AAA family ATPase	

TvaB18	Tv_09126-RA<br>Tv_13900-RA<br>Tv_14921-RA<br>Tv_15127-RA<br>Tv_15631-RA	Bacteria	/	34.3<br>32.9<br>31.3<br>35.4<br>33.3	30.9<br>29.8<br>28.7<br>31.7<br>31.3	462<br>408<br>539<br>559<br>558	72.1<br>90.2<br>95.1<br>91.6<br>89.2	0.88<br>0.86<br>0.64<br>0.84<br>0.78				AAA family ATPase	
TvaB19	Tv_08602-RA<br>Tv_10007-RA	Bacteria	/	68.2<br>68.4	66.9<br>67.0	371<br>380	65.4<br>86.1	0.8<br>0.9	Bta02653,<br>Bta04469,<br>Bta05264,<br>Bta06818,<br>Bta11911,<br>Bta11912	BTA005171.1,<br>BTA005173.1,<br>BTA012513.1,<br>BTA019053.1,<br>BTA023694.1,<br>BTA023695.1,<br>BTA029954.2	Ssa00993,<br>Ssa02587,<br>Ssa02588,<br>Ssa02589,<br>Ssa09979,<br>Ssa12718	cyclopropane fatty acyl phospholipid	
TvaB20	Tv_15468-RA	Bacteria	1	69.8	64.1	308	90.8	0.92	Bta09725	BTA004219.1,<br>BTA028913.1	Ssa10171	Biotin synthase	
TvaB21	Tv_04577-RA<br>Tv_08891-RA<br>Tv_08906-RA<br>Tv_08957-RA<br>Tv_08958-RA	Bacteria	/	36.6<br>35.1<br>35.9<br>36.1<br>34.4	31.2<br>30.3<br>31.0<br>30.7<br>31.4	416<br>441<br>389<br>428<br>393	66.9<br>70.4<br>83.0<br>73.8<br>84.6	0.91<br>0.87<br>0.83<br>0.86<br>0.86				AAA family ATPase	
TvaB22	Tv_02105-RA	Bacteria	/	39.2	35.6	251	95.3	0.62	Bta11531,<br>Bta15479	BTA004921.1,<br>BTA018889.1,<br>BTA026605.1	Ssa12442	AAA family ATPase	
TvaB23	TLow_02263-RA<br>Tv_09587-RA<br>Tv_13579-RA<br>Tv_14715-RA<br>Tv_14844-RA	Bacteria	/	35.4<br>35.8<br>34.2<br>33.4<br>34.9	33.2<br>30.5<br>31.5<br>31.1<br>32.1	287<br>463<br>482<br>302<br>409	94.6<br>71.9<br>81.4<br>73.6<br>94.1	0.81<br>0.96<br>0.88<br>0.92<br>0.78				AAA family ATPase	
TvaB24	TLow_01084-RA<br>Tv_01996-RA<br>Tv_11525-RA<br>Tv_15653-RA	Bacteria	/	60.3<br>55.9<br>57.1<br>57.1	54.3<br>46.9<br>50.7<br>50.8	66<br>79<br>143<br>143	82.8<br>52.2<br>94.8<br>94.7	0.96<br>0.76<br>0.76<br>0.91				dUTP diphosphatase	

Fungal donor:

HGT event	Sequence name	Origin of donor sequences	Alternative topology	Similarities with the donor sequences		Length and coverage of the alignment with the donor sequences		Local score for genomic environment	Homologs found in the *B. tabaci* cryptic species			Annotation (BLAST NR)	CAZy
				max id	average id	average aln length	average coverage		Btab_MEAM1	Btab_MED	Btab_SSA		
TvaF01	Tv_08361-RA<br>Tv_14110-RA<br>Tv_14111-RA	Fungi	/	32,0<br>34,0<br>34,3	30,2<br>31,3<br>31,6	910<br>681<br>1274	95,3<br>95,0<br>99,0	0.7<br>0.68<br>0.68	Bta00932,<br>Bta05475,<br>Bta07433	BTA005597.1,<br>BTA005598.1,<br>BTA005599.1,<br>BTA007606.1,<br>BTA003000.1,<br>BTA004644.1,<br>BTA008032.1,<br>BTA008563.1	sa06477,<br>Ssa06478,<br>Ssa06479,<br>Ssa04914,<br>Ssa15384	MYND finger family protein	
TvaF02	Tv_08362-RA	Fungi	/	35,7	32,8	331	96,5	0.66	Bta07948	BTA017217.1,<br>BTA017221.1,<br>BTA021006.1	Ssa00835	MYND finger family protein	
TvaF03	Tv_07966-RA	Fungi	/	49,9	45,9	2392	97,7	0.78	Bta08944,<br>Bta08945,<br>Bta08946,<br>Bta14076	BTA016279.2,<br>BTA025951.2,<br>BTA025953.1,<br>BTA025954.1	Ssa11553,<br>Ssa12591,<br>Ssa15247	NFX1-type zinc finger-containing protein 1	
TvaF04	Tv_10871-RA<br>Tv_10872-RA<br>Tv_11256-RA	Fungi	/	54,8<br>55,7<br>51,6	36,9<br>37,1<br>36,8	216<br>213<br>212	73,9<br>73,1<br>68,7	0.64<br>0.64<br>0.91	Bta03426	BTA002830.1,<br>BTA019829.1,<br>BTA029129.1,<br>BTA002831.1	Ssa05110,<br>scaffold50840_2_<br>365	Unknown protein	

Viridiplantae donor:

HGT event	Sequence name	Origin of donor sequences	Alternative topology	Similarities with the donor sequences		Length and coverage of the alignment with the donor sequences		Local score for genomic environment	Homologs found in the *B. tabaci* cryptic species			Annotation (BLAST NR)	CAZy
				max id	average id	average aln length	average coverage		Btab_MEAM1	Btab_MED	Btab_SSA		
TvaV01	Tv_15928-RA	Viridiplantae	0	60,2	57,7	202	91,7	1.0	Bta13961	BTA005662.2	Ssa10394	Thaumatin-like protein	GH152
TvaV02	TLow_01580-RA<br>Tv_15540-RA<br>Tv_15541-RA	Viridiplantae	/	39,4<br>38,1<br>31,5	34,6<br>34,1<br>27,7	220<br>223<br>239	83,5<br>74,2<br>82,5	0.61<br>0.71<br>0.61	Bta13094,<br>Bta13103	BTA025985.1,<br>BTA025994.1	Ssa09189,<br>scaffold212068_54_492	rRNA N-glycosidase	
TvaV03	Tv_08972-RA	Viridiplantae	/	71,0	68,4	379	96,9	0.96	Bta04264,<br>Bta06451,<br>Bta06452,<br>Bta06453,<br>Bta09295,<br>Bta13659,<br>Bta15754,<br>Bta15755,<br>Bta15756,<br>Bta15757,<br>Bta15759,<br>Bta15760,<br>Bta15762,<br>Bta15765	BTA007356.1,<br>BTA011448.1,<br>BTA015354.2,<br>BTA015355.1,<br>BTA028840.1,<br>BTA015596.1,<br>BTA011446.1	Ssa02331,<br>Ssa02332,<br>Ssa02334,<br>Ssa02342,<br>Ssa03431,<br>Ssa08612,<br>Ssa11969,<br>Ssa15146,<br>Ssa15387,<br>Ssa06732,<br>Ssa02337,<br>Ssa02338,<br>Ssa03432	delta(12)-fatty-acid desaturase FAD2	

Complex: Bacterial or Fungal donor

HGT event	Sequence name	Origin of donor sequences	Alternative topology	Similarities with the donor sequences		Length and coverage of the alignment with the donor sequences		Local score for genomic environment	Homologs found in the *B. tabaci* cryptic species			Annotation (BLAST NR)	CAZy
				max id	average id	average aln length	average coverage		Btab_MEAM1	Btab_MED	Btab_SSA		
TvaC01	Tv_05105-RA	Bacteria or Fungi	0	34.2	30.5	351	80.6	0.76	Bta02634	BTA005662.2	Ssa10394	Pectin lyase	PL1_4

Supplementary Table 7: Analysis of the 11 combined groups for *B. tabaci* and *T. vaporariorum* in which the grouping of sequences lacked bootstrap support and/or was not monphyletic

Results of bootstrap analysis for the combined groups for which the grouping of sequences lacked bootstrap support:

Combined group	Ultrafast bootstrap support value
TvaB05_BtaB02	70%
TvaV02_BtaV23	91%

Support values were based on an ultrafast bootstrap approximation with 1,000 replicates.
Only support values greater than or equal to 95% were considered.

Results of the approximately unbiased (AU) alternative topology test for monophyly of Aleyrodinae sequences for the combined groups in which the grouping of sequences was not monophyletic:

Combined group	Tree	logL	deltaL	p-AU	
TvaB01_BtaB56	1	-54869.4139	3.9658	0.434	+
	2	-54865.44813	0	0.566	+
TvaB02_BtaB12	1	-15665.71625	2.8104	0.379	+
	2	-15662.90584	0	0.621	+
TvaB03_BtaB68	1	-25152.92208	0	1	+
	2	-25661.69097	508.77	6.13e-06	-
TvaB04_BtaB44	1	-15393.59309	18.658	0.129	+
	2	-15374.93497	0	0.871	+
TvaB08_BtaB31	1	-4046.24064	18.874	0.12	+
	2	-4027.366596	0	0.88	+
TvaB11_BtaB03	1	-18272.4574	103.52	8.67e-07	-
	2	-18168.93243	0	1	+
TvaF04_BtaF10	1	-3488.191761	2.077	0.428	+
	2	-3486.114778	0	0.572	+
TvaV01_BtaV01	1	-11952.22823	25.552	0.177	+
	2	-11926.67646	0	0.823	+
TvaV03_BtaV21	1	-44046.69688	5.2108	0.46	+
	2	-44041.48611	0	0.54	+

Plus signs denote the 95% confidence sets.
Minus signs denote significant exclusion.
All tests performed 10,000 resamplings using the RELL method.

Supplementary Table 8: Overrepresented GO terms among validated HGT candidates from potential bacterial, fungal or viridiplantae donors for *T. vaporariorum*

<table>
<tr><th>root_node_name</th><th>node_id</th><th>node_name</th><th>raw_p_overrep</th><th>FWER_overrep</th><th>refined_p_overrep</th><th>FDR_overrep</th><th>nb_genes_refset_root_node</th><th>nb_genes_candidate_in_root_node</th><th>nb_gene_refset_node</th><th>nb_genes_candidate_in_node</th><th>name_genes_candidate_in_node</th></tr>
<tr><td>HGT Bacteria</td><td></td><td></td><td></td><td></td><td></td><td></td><td></td><td></td><td></td><td></td><td></td></tr>
<tr><td>molecular_function</td><td>GO:0000287</td><td>magnesium ion binding</td><td>2.39324290613495e-22</td><td>0</td><td>2.39324290613495e-22</td><td>8.46182313240573e-22</td><td>6896</td><td>17</td><td>42</td><td>11</td><td>TLow_01084-RA|Tv_01894-RA|Tv_02831-RA|Tv_07802-RA|Tv_09064-RA|Tv_11525-RA|Tv_11854-RA|Tv_12101-RA|Tv_15653-RA|Tv_15720-RA|Tv_15881-RA</td></tr>
<tr><td>molecular_function</td><td>GO:0004170</td><td>dUTP diphosphatase activity</td><td>6.51336021681486e-26</td><td>0</td><td>6.51336021681486e-26</td><td>6.44822661464671e-24</td><td>6896</td><td>17</td><td>14</td><td>10</td><td>TLow_01084-RA|Tv_02831-RA|Tv_07802-RA|Tv_09064-RA|Tv_11525-RA|Tv_11854-RA|Tv_12101-RA|Tv_15653-RA|Tv_15720-RA|Tv_15881-RA</td></tr>
<tr><td>biological_process</td><td>GO:0006226</td><td>dUMP biosynthetic process</td><td>8.68032002463571e-24</td><td>0</td><td>8.68032002463571e-24</td><td>7.16126402032446e-23</td><td>3410</td><td>17</td><td>14</td><td>10</td><td>TLow_01084-RA|Tv_02831-RA|Tv_07802-RA|Tv_09064-RA|Tv_11525-RA|Tv_11854-RA|Tv_12101-RA|Tv_15653-RA|Tv_15720-RA|Tv_15881-RA</td></tr>
<tr><td>biological_process</td><td>GO:0009102</td><td>biotin biosynthetic process</td><td>3.24251943712581e-08</td><td>0</td><td>3.24251943712581e-08</td><td>4.58584891822079e-08</td><td>3410</td><td>17</td><td>3</td><td>3</td><td>Tv_01894-RA|Tv_01895-RA|Tv_15468-RA</td></tr>
<tr><td>biological_process</td><td>GO:0046081</td><td>dUTP catabolic process</td><td>8.68032002463571e-24</td><td>0</td><td>8.68032002463571e-24</td><td>7.16126402032446e-23</td><td>3410</td><td>17</td><td>14</td><td>10</td><td>TLow_01084-RA|Tv_02831-RA|Tv_07802-RA|Tv_09064-RA|Tv_11525-RA|Tv_11854-RA|Tv_12101-RA|Tv_15653-RA|Tv_15720-RA|Tv_15881-RA</td></tr>
<tr><td>HGT Viridiplantae</td><td></td><td></td><td></td><td></td><td></td><td></td><td></td><td></td><td></td><td></td><td></td></tr>
<tr><td>molecular_function</td><td>GO:0030598</td><td>rRNA N-glycosylase activity</td><td>4.67171528940521e-11</td><td>0</td><td>4.67171528940521e-11</td><td>7.47474446304833e-10</td><td>5381</td><td>3</td><td>3</td><td>3</td><td>TLow_01580-RA|Tv_15540-RA|Tv_15541-RA</td></tr>
<tr><td>biological_process</td><td>GO:0017148</td><td>negative regulation of translation</td><td>8.89476306015845e-09</td><td>0</td><td>8.89476306015845e-09</td><td>4.06617739892958e-08</td><td>445</td><td>3</td><td>8</td><td>3</td><td>TLow_01580-RA|Tv_15540-RA|Tv_15541-RA</td></tr>
<tr><td>HGT Bacteria + Fungi + Viridiplantae</td><td></td><td></td><td></td><td></td><td></td><td></td><td></td><td></td><td></td><td></td><td></td></tr>
<tr><td>molecular_function</td><td>GO:0000287</td><td>magnesium ion binding</td><td>1.34007184990538e-20</td><td>0</td><td>1.34007184990538e-20</td><td>4.79832178514505e-20</td><td>6896</td><td>22</td><td>42</td><td>11</td><td>TLow_01084-RA|Tv_01894-RA|Tv_02831-RA|Tv_07802-RA|Tv_09064-RA|Tv_11525-RA|Tv_11854-RA|Tv_12101-RA|Tv_15653-RA|Tv_15720-RA|Tv_15881-RA</td></tr>
<tr><td>molecular_function</td><td>GO:0004170</td><td>dUTP diphosphatase activity</td><td>2.16076981295708e-24</td><td>0</td><td>2.16076981295708e-24</td><td>2.39845449238236e-22</td><td>6896</td><td>22</td><td>14</td><td>10</td><td>TLow_01084-RA|Tv_02831-RA|Tv_07802-RA|Tv_09064-RA|Tv_11525-RA|Tv_11854-RA|Tv_12101-RA|Tv_15653-RA|Tv_15720-RA|Tv_15881-RA</td></tr>
<tr><td>molecular_function</td><td>GO:0030598</td><td>rRNA N-glycosylase activity</td><td>1.79861038642101e-08</td><td>0</td><td>1.79861038642101e-08</td><td>2.77285767906572e-08</td><td>6896</td><td>22</td><td>3</td><td>3</td><td>TLow_01580-RA|Tv_15540-RA|Tv_15541-RA</td></tr>
<tr><td>biological_process</td><td>GO:0006226</td><td>dUMP biosynthetic process</td><td>1.27924660789978e-22</td><td>0</td><td>1.27924660789978e-22</td><td>1.1833031123073e-21</td><td>3654</td><td>22</td><td>14</td><td>10</td><td>TLow_01084-RA|Tv_02831-RA|Tv_07802-RA|Tv_09064-RA|Tv_11525-RA|Tv_11854-RA|Tv_12101-RA|Tv_15653-RA|Tv_15720-RA|Tv_15881-RA</td></tr>
<tr><td>biological_process</td><td>GO:0009102</td><td>biotin biosynthetic process</td><td>6.11946070241879e-08</td><td>0</td><td>6.11946070241879e-08</td><td>8.93763339432218e-08</td><td>3654</td><td>22</td><td>3</td><td>3</td><td>Tv_01894-RA|Tv_01895-RA|Tv_15468-RA</td></tr>
<tr><td>biological_process</td><td>GO:0046081</td><td>dUTP catabolic process</td><td>1.27924660789978e-22</td><td>0</td><td>1.27924660789978e-22</td><td>1.1833031123073e-21</td><td>3654</td><td>22</td><td>14</td><td>10</td><td>TLow_01084-RA|Tv_02831-RA|Tv_07802-RA|Tv_09064-RA|Tv_11525-RA|Tv_11854-RA|Tv_12101-RA|Tv_15653-RA|Tv_15720-RA|Tv_15881-RA</td></tr>
<tr><td>biological_process</td><td>GO:0017148</td><td>negative regulation of translation</td><td>3.38127547060968e-06</td><td>0.001</td><td>3.38127547060968e-06</td><td>4.57709240533749e-06</td><td>3654</td><td>22</td><td>8</td><td>3</td><td>TLow_01580-RA|Tv_15540-RA|Tv_15541-RA</td></tr>
</table>

Supplementary Table 9: Validated HGT candidates from potential bacterial, fungal or viridiplantae donors for *F. occidentalis*

Bacterial donor:

HGT event	Sequence name	Origin of donor sequences	Alternative topology Monophyly of Metazoa	Similarities with the donor sequences		Length and coverage of the alignment with the donor sequences		Local score for genomic environment	Homologs found in *T. palmi*	Annotation (BLAST NR)	CAZy	Described in literature
				max id	average id	average aln length	average coverage					
FocB01	XP_026273122.1	Bacteria	/	53.1	47.9	494	76.6	0.92	XP_034232383.1,	Levanase-like	GH32	Rotenberg et al. 2020;
	XP_026287851.1			47.6	44.2	493	90.1	/	XP_034233420.1			Li et al. 2022
FocB02	XP_026276666.1	Bacteria	0	55	52.8	308	95.6	0.9	XP_034242052.1,	Mannan endo-1,4-beta-	GH5_8	Rotenberg et al. 2020;
	XP_026285289.1			55.6	52.8	311	94.2	/	XP_034242578.1	mannosidase-like		Li et al. 2022
	XP_026285291.1			56	53.7	306	95	/				
FocB03	XP_026272963.1	Bacteria	0	54.4	50	143	43.8	0.84	XP_034249514.1	Riboflavin biosynthesis protein		No
FocB04	XP_026284437.1	Bacteria	/	47.5	41.7	117	83.1	0.62	XP_034246168.1	Unknown protein		No
FocB05	XP_026277179.1	Bacteria	1	52.1	46.6	220	92	0.8		O-methyltransferase		Rotenberg et al. 2020;
	XP_026279074.1			52.4	46.3	214	84	0.42				Li et al. 2022
	XP_026279079.1			50.5	45.2	212	93.1	0.5				

Fungal donor:

HGT event	Sequence name	Origin of donor sequences	Alternative topology Monophyly of Metazoa	Similarities with the donor sequences		Length and coverage of the alignment with the donor sequences		Local score for genomic environment	Homologs found in *T. palmi*	Annotation (BLAST NR)	CAZy	Described in literature
				max id	average id	average aln length	average coverage					
FocF01	XP_026287984.1	Fungi	/	57.8	54.2	212	89.8	0.32	XP_034233350.1,	Endoglucanase	GH45	No
	XP_026289264.1			56.2	52	213	89.8	0.68	XP_034236588.1			
	XP_026291890.1			62.8	57.8	133	51.4	/				

Viridiplantae donor:

HGT event	Sequence name	Origin of donor sequences	Alternative topology Monophyly of Metazoa	Similarities with the donor sequences		Length and coverage of the alignment with the donor sequences		Local score for genomic environment	Homologs found in *T. palmi*	Annotation (BLAST NR)	CAZy	Described in literature
				max id	average id	average aln length	average coverage					
FocV01	XP_026290383.1	Viridiplantae	/	58.1	55.1	227	93	0.66	XP_034247376.1,<br>XP_034247673.1	Pathogenesis-related protein 5-like	GH152	No

Complex (Bacterial or Fungal donor):

HGT event	Sequence name	Origin of donor sequences	Alternative topology Monophyly of Metazoa	Similarities with the donor sequences		Length and coverage of the alignment with the donor sequences		Local score for genomic environment	Homologs found in *T. palmi*	Annotation (BLAST NR)	CAZy	Described in literature
				max id	average id	average aln length	average coverage					
FocC01	XP_026274115.1	Bacteria or	0	42.8	39.8	336	88.2	0.56	XP_034235640.1,	Pectin lyase	PL1_4	No
	XP_026279513.1	Fungi		42.4	38.7	351	87.4	0.56	XP_034237191.1,			Li et al. 2022
	XP_026279528.1			41.9	40.4	325	81.0	0.62	XP_034239440.1,			Li et al. 2022
	XP_026280402.1			42.5	39.6	337	88.7	0.8	XP_034241038.1,			No
	XP_026285066.1			42.6	40.2	328	86.8	0.73	XP_034248354.1,			No
	XP_026285067.1			42.3	40.8	330	87.0	0.71	XP_034248552.1,			Li et al. 2022
	XP_026285883.1			42.9	40.9	335	86.8	0.96	XP_034253769.1,			No
	XP_026289641.1			41.2	40.0	320	84.8	0.0	XP_034253771.1,			No
	XP_026292396.1			42.6	41.3	324	85.7	0.61	XP_034254938.1			No
	XP_026292397.1			42.9	41.5	324	85.7	0.61				No

Supplementary Table 10: Validated HGT candidates from potential bacterial, fungal or viridiplantae donors for *T. palmi*

Bacterial donor:

HGT event	Sequence name	Origin of donor sequences	Alternative topology Monophyly of Metazoa	Similarities with the donor sequences		Length and coverage of the alignment with the donor sequences		Local score for genomic environment	Homologs found in *F. occidentalis*	Annotation (BLAST NR)	CAZy	Described in literature
				max id	average id	average aln length	average coverage					
TpaB01	XP_034232383.1<br>XP_034233420.1	Bacteria	/	46<br>55	43.6<br>48.9	511<br>497	91.5<br>86	0.76<br>0.68	XP_026273122.1,<br>XP_026287851.1	Levanase-like	GH32	Li et al. 2022
TpaB02	XP_034242052.1<br>XP_034242578.1	Bacteria	/	59.2<br>59.5	55.4<br>55.3	302<br>306	93.5<br>94.4	0.58<br>0.62	XP_026276666.1,<br>XP_026285289.1,<br>XP_026285291.1	Mannan endo-1,4-beta-mannosidase-like	GH5_8	Li et al. 2022
TpaB03	XP_034249514.1	Bacteria	0	57.3	49.4	145	74.8	0.72	XP_026272963.1	Riboflavin biosynthesis protein		Li et al. 2022
TpaB04	XP_034246168.1	Bacteria	/	47.8	40.3	128	77.9	0.44	XP_026284437.1	Unknown protein		No

Fungal donor:

HGT event	Sequence name	Origin of donor sequences	Alternative topology Monophyly of Metazoa	Similarities with the donor sequences		Length and coverage of the alignment with the donor sequences		Local score for genomic environment	Homologs found in *F. occidentalis*	Annotation (BLAST NR)	CAZy	Described in literature
				max id	average id	average aln length	average coverage					
TpaF01	XP_034236588.1<br>XP_034233350.1	Fungi	/	58.5<br>60	54.3<br>52	218<br>174	89.8<br>54.4	0.56<br>0.77	XP_026287984.1,<br>XP_026289264.1,<br>XP_026291890.1	Endoglucanase	GH45	No

Viridiplantae donor:

HGT event	Sequence name	Origin of donor sequences	Alternative topology Monophyly of Metazoa	Similarities with the donor sequences		Length and coverage of the alignment with the donor sequences		Local score for genomic environment	Homologs found in *F. occidentalis*	Annotation (BLAST NR)	CAZy	Described in literature
				max id	average id	average aln length	average coverage					
TpaV01	XP_034247376.1<br>XP_034247673.1	Viridiplantae	/	60.4<br>61.3	57.4<br>58	226<br>221	94<br>92	0.3<br>0.5	XP_026290383.1	Pathogenesis-related protein 5-like	GH152	No
TpaV02	XP_034245819.1	Viridiplantae	/	57	48.9	292	43.2	0.89		Glucan endo-1,3-beta-glucosidase 13-like	CBM43-CBM43-CBM43-CBM43	Li et al. 2022

Complex (Bacterial or Fungal donor):

HGT event	Sequence name	Origin of donor sequences	Alternative topology Monophyly of Metazoa	Similarities with the donor sequences		Length and coverage of the alignment with the donor sequences		Local score for genomic environment	Homologs found in *F. occidentalis*	Annotation (BLAST NR)	CAZy	Described in literature
				max id	average id	average aln length	average coverage					
TpaC01	XP_034235640.1<br>XP_034237191.1<br>XP_034239440.1<br>XP_034241038.1<br>XP_034248354.1<br>XP_034248552.1<br>XP_034253769.1<br>XP_034253771.1<br>XP_034254938.1	Bacteria or Fungi	0	43.4<br>43.7<br>53.7<br>39.8<br>40.9<br>40.3<br>40.6<br>41.5<br>40.8	40.27<br>40.3<br>47.3<br>38.1<br>38.9<br>37.6<br>38.8<br>39.3<br>39.3	325<br>326<br>136<br>334<br>339<br>339<br>343<br>334<br>336.6	81.7<br>82.0<br>83.9<br>88.2<br>89.3<br>89.0<br>90.6<br>88.6<br>89.9	0.58<br>0.8<br>0.4<br>0.36<br>0.38<br>0.48<br>0.44<br>0.36<br>0.68	XP_026274115.1,<br>XP_026279513.1,<br>XP_026279528.1,<br>XP_026280402.1,<br>XP_026285066.1,<br>XP_026285067.1,<br>XP_026285883.1,<br>XP_026289641.1,<br>XP_026292396.1,<br>XP_026292397.1	Pectin lyase	PL1_4	Li et al. 2022

Supplementary Table 11: Overrepresented GO terms among validated HGT candidates from potential bacterial, fungal or viridiplantae donors for *F. occidentalis* and *T. palmi*

F. occidentalis:

root_node_name	node_id	node_name	raw_p_overrep	FWER_overrep	refined_p_overrep	FDR_overrep	nb_genes_refset_root_node	nb_genes_candidate_in_root_node	nb_gene_refset_node	nb_genes_candidate_in_node	name_genes_candidate_in_node
HGT Bacteria											
molecular_function	GO:0004553	hydrolase activity, hydrolyzing O-glycosyl compounds	7.45253094404549e-08	0	7.45253094404549e-08	2.9810123776182e-07	5180	8	131	5	XP_026273122.1|XP_026276666.1|XP_026285289.1|XP_026285291.1|XP_026287851.1
molecular_function	GO:0008171	O-methyltransferase activity	2.64852256493027e-08	0	2.64852256493027e-08	2.11881805194422e-07	5180	8	7	3	XP_026277179.1|XP_026279074.1|XP_026279079.1
HGT Fungi											
molecular_function	GO:0008810	cellulase activity	1.353949901403e-11	0	1.353949901403e-11	8.12369940841798e-11	5113	3	3	3	XP_026287984.1|XP_026289264.1|XP_026291890.1
biological_process	GO:0005975	carbohydrate metabolic process	7.51497630059906e-05	0.001	7.51497630059906e-05	0.000112724644508986	4038	3	212	3	XP_026287984.1|XP_026289264.1|XP_026291890.1
HGT Bacteria + Fungi + Viridiplantae											
molecular_function	GO:0004553	hydrolase activity, hydrolyzing O-glycosyl compounds	9.67932473173405e-13	0	5.25368376343683e-07	1.35510546244277e-11	6668	11	131	8	XP_026273122.1|XP_026276666.1|XP_026285289.1|XP_026285291.1|XP_026287851.1|XP_026287984.1|XP_026289264.1|XP_026291890.1
molecular_function	GO:0008810	cellulase activity	2.23401733731494e-09	0	2.23401733731494e-09	1.04254142408031e-08	6668	11	3	3	XP_026287984.1|XP_026289264.1|XP_026291890.1
molecular_function	GO:0008171	O-methyltransferase activity	7.7944673051812e-08	0.001	7.7944673051812e-08	2.72806355681342e-07	6668	11	7	3	XP_026277179.1|XP_026279074.1|XP_026279079.1
biological_process	GO:0005975	carbohydrate metabolic process	6.59293839201467e-06	0	6.59293839201467e-06	1.84602274976411e-05	4569	5	212	5	XP_026273122.1|XP_026287851.1|XP_026287984.1|XP_026289264.1|XP_026291890.1

T. palmi:

root_node_name	node_id	node_name	raw_p_overrep	FWER_overrep	refined_p_overrep	FDR_overrep	nb_genes_refset_root_node	nb_genes_candidate_in_root_node	nb_gene_refset_node	nb_genes_candidate_in_node	name_genes_candidate_in_node
HGT Bacteria											
molecular_function	GO:0004553	hydrolase activity, hydrolyzing O-glycosyl compounds	6.24920835945864e-08	0	6.24920835945864e-08	2.10908540217664e-07	4850	4	116	4	XP_034232383.1|XP_034233420.1|XP_034242052.1|XP_034242578.1
HGT Fungi											
molecular_function	GO:0008810	cellulase activity	3.81287870702843e-08	0	3.81287870702843e-08	1.14386361210853e-07	116	2	2	2	XP_034233350.1|XP_034236588.1
HGT Bacteria + Fungi + Viridiplantae											
molecular_function	GO:0004553	hydrolase activity, hydrolyzing O-glycosyl compounds	1.48275313553821e-11	0	8.52471690838028e-07	1.48275313553821e-10	5461	6	116	6	XP_034232383.1|XP_034233350.1|XP_034233420.1|XP_034236588.1|XP_034242052.1|XP_034242578.1
molecular_function	GO:0008810	cellulase activity	5.71931806054265e-07	0	5.71931806054265e-07	1.90643935351422e-06	5461	6	2	2	XP_034233350.1|XP_034236588.1
biological_process	GO:0005975	carbohydrate metabolic process	3.67257482566665e-05	0.006	3.67257482566665e-05	7.34514965133331e-05	4347	4	194	4	XP_034232383.1|XP_034233350.1|XP_034233420.1|XP_034236588.1
